# Synthesis of New DltA Inhibitors and Their Application as Adjuvant Antibiotics to Re-Sensitize Methicillin-Resistant *Staphylococcus aureus*

**DOI:** 10.3390/molecules30122569

**Published:** 2025-06-12

**Authors:** David Leparfait, Alexandre Mahé, Xiao Feng, Delphine Coupri, Fabien Le Cavelier, Nicolas Verneuil, Emmanuel Pfund, Aurélie Budin-Verneuil, Thierry Lequeux

**Affiliations:** 1 ENSICAEN, Université de Caen Normandie, Université de Rouen Normandie, INSA Rouen Normandie, CNRS, Institut CARMeN UMR 6064, 14050 Caen, France; 2 CBSA UR 4312, Université de Caen Normandie, 14000 Caen, France

**Keywords:** nucleoside analogs, DltA inhibitor, antibiotic resistance

## Abstract

The synthesis of a new acyclic and cyclic series of D-Ala-AMP analogues was reported. Chemical modifications were introduced on the carbohydrate, the sulfamate linker, and/or the amino-acid *N*-terminal moiety in order to increase *in vivo* stability and cell permeability. These new compounds were evaluated *in vitro* as DltA inhibitors and also *in vivo* as adjuvant antibiotics to re-sensitize methicillin-resistant *Staphylococcus aureus*. Indeed, we showed that seven nucleosides containing either a fluorine atom, an azido group, a difluorophosphonylated allylic ether moiety onto the 2′-position, or a sulfamate and a triazole as the sulfamate linker had moderate to excellent IC_50_ values. Among all these new DltA inhibitors, two molecules functionalized by the fluorinated ether or the sulfamide linker were able to efficiently re-sensitize MRSA to imipenem. Quantification of D-alanyl esters confirmed that these two compounds reduced the level of bacterial cell wall D-alanyl residues by 50% and 80%.

## 1. Introduction

**Figure 1:**
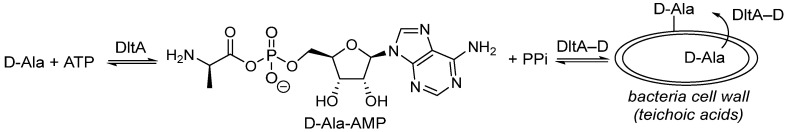
D-alanylation of cell wall teichoic acids.

Antibiotics represent one of the most significant medical discoveries of the 20th century, revolutionizing the treatment of bacterial infections and saving countless lives worldwide for several decades [1,2]. Antibacterial agents were initially designed to either kill bacteria or inhibit their growth by counteracting essential biological functions such as cell wall synthesis, DNA replication, and protein synthesis. However, these modes of action exert a selective pressure that modifies the human microbiome and induces antimicrobial resistance (AMR) [3,4,5]. As a result, inappropriate utilization and overuse of antibiotics in the past have accelerated the emergence of antibiotic-resistant bacterial strains, rendering previously effective treatments obsolete, and posing grave threats to public health [6,7]. This phenomenon was further amplified with the COVID-19 pandemic, where, in addition to the increased use of antibacterial cleaning products [8], approximately 75% of patients were treated with antibiotics although bacterial co-infection was estimated at 8.6% [9]. In 2019, 4.95 million deaths worldwide were associated with bacterial resistance to antibiotics, including 1.27 million directly attributable to AMR, positioning it as a major global burden of diseases ahead of HIV and malaria [10]. The development of new therapeutic approaches involving active compounds towards new targets remains an important challenge and is an emergency requirement for human care. In this field, adjuvant antibiotics have emerged and represent a promising way to fight AMR infections [11]. These molecules are designed to improve antibiotic actions or re-sensitize pathogens to drugs from which they have become resistant by targeting the antibiotic resistance mechanism. D-alanylation of teichoic acids serves as an effective strategy for Gram-positive bacteria to develop antibioresistance [12]. This process, catalyzed by five proteins (DltXABCD) encoded by the *dlt* operon [13,14], transfers a D-alanine residue onto the cell wall, altering its net charge and permeability, thereby reducing susceptibility to cationic antimicrobial peptides and glycopeptides such as vancomycin (Figure 1) [15].

**Figure 2:**
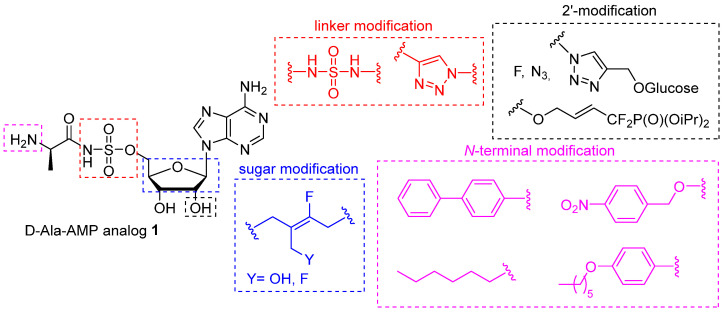
Chemical modifications envisaged for D-Ala-AMP.

Indeed, nucleoside **1**, designed as a D-Ala-AMP analog, was reported as the sole DltA inhibitor (Ki = 0.2 μM) and found to be efficient as an adjuvant to inhibit the growth of *Bacillus subtilis*, a non-pathogenic bacterium, in the presence of vancomycin [16]. We recently showed that compound **1** was also able to counteract the antibiotic resistance of methicillin-resistant *Staphylococcus aureus* (MRSA) and β-lactam-resistant *enterococci*, including vancomycin-resistant strains, when co-administered with the antibiotics [17,18,19]. However, high concentrations (500 μM to 1 mM) of inhibitor **1** were needed, suggesting that nucleoside **1** may suffer from low metabolic stability and/or weak cellular uptake. For this type of nucleoside, both the glycosidic bond and the sulfamate function were identified as being responsible for its enzymatic and chemical instability. As a result, *N*-acylsquaramides, *N*-acylsulfamides, and triazoles derived from acyclo-, morpholino-, and carba-nucleosides have already been proposed to overcome this main limitation but were never applied to **1** [20,21]. Thus, the design of new DltA inhibitors with improved stability and cell permeability still deserves to be developed for clinical use. In this context, synthesis of stable analogues of D-Ala-AMP, in which D-Alanine is attached to the nucleoside by a non-scissile bond in order to prevent its transfer and block the D-alanylation process, is of particular interest. In this paper, we report the synthesis, molecular docking, and biological studies of a new series of nucleosides derived from inhibitor **1** through chemical modifications of the carbohydrate, the sulfamate linker, and/or the amino-acid *N*-terminal moiety (Figure 2).

## 2. Results and Discussion

### 2.1. Chemistry

**Scheme 1:**
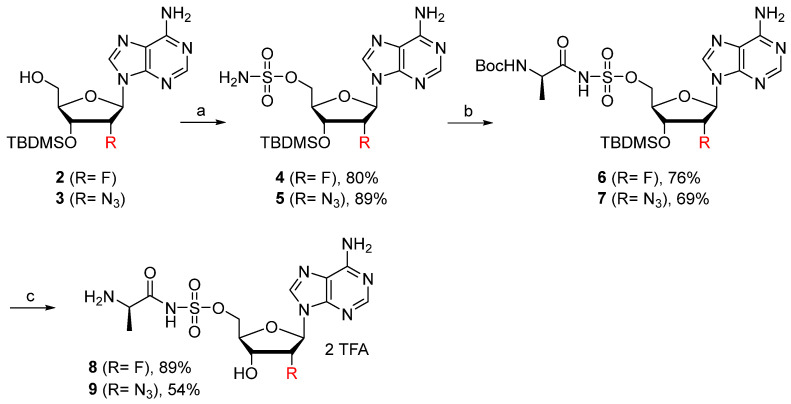
Reagents and conditions: (a) (i) NH_2_SO_2_Cl, DMA, MeCN, 20 °C, 2 h. (ii) Et_3_N, MeOH, 0 °C, 10 min. (b) Boc-D-Ala-OSu, DBU, DMF, 20 °C, 48 h for **6** and 16 h for **7**. (c) TFA, H_2_O, CH_2_Cl_2_, 20 °C, 5 h for **8** and 12 h for **9**.

The introduction of electron-withdrawing groups onto the 2′-position of nucleosides is well known to increase the *N*-glycosidic bond stability. This chemical modification was first considered. Indeed, 2′-fluoro and 2′-azido *N*-acylsulfamate adenosine **8**–**9** were prepared according to the procedure reported by Herdewijn (Scheme 1) [22]. Known compounds **2** [22] and **3** [23] were reacted with NH_2_SO_2_Cl, affording the corresponding *N*-sulfamoyl derivatives **4**–**5** in 80% and 89% yield, respectively. The desired nucleosides **8** and **9** were finally obtained in two steps involving a coupling reaction with Boc-D-Ala-Osu, followed by amine and alcohol deprotection.

**Scheme 2:**
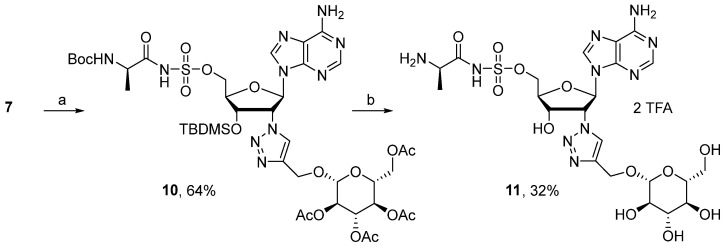
Reagents and conditions: (a) 1-*O*-propargyl-2,3,4,-tetra-*O*-acetyl-D-glucopyranose, CuSO_4_, NaAsc, *t*-BuOH, H_2_O, 20 °C, 24 h. (b) (i) TFA, H_2_O, 20 °C, 12 h. (ii) MeONa, MeOH, 20 °C, 1 h. (iii) Et_3_N.3HF, THF, 20 °C, 12 h.

Carbohydrate–drug conjugates have already been explored to enhance cellular uptake through specific glucose transporters such as GLUT proteins [24,25,26,27]. Indeed, glycoconjugates of various cytotoxic molecules were successfully developed to improve their penetration into cancer cells [28,29]. In the field of antibiotics, the use of β-cyclodextrin grafted with D-glucose and D-mannose potentiated the activity of erythromycin, rifampicin, and ciprofloxacin [30]. With respect to the structure–activity relationship of D-glucose and GLUT transporter [28] and inspired by the different linkers already used in conjugation [24,25,26,27,28,29,30,31], incorporation of a glucose moiety onto the 2′-position through a triazolyl linker was next envisaged (Scheme 2). D-glucose was introduced *via* a 1,3-dipolar cycloaddition reaction between **7** and 1-*O*-propargyl-2,3,4,-tetra-*O*-acetyl-D-glucopyranose. After 24 h of stirring at 20 °C in *t*-BuOH/H_2_O in the presence of CuSO_4_ and sodium ascorbate, triazole **10** was isolated in 64% yield. Finally, additional treatment with TFA, sodium methoxide, and Et_3_N.3HF led to the formation of the desired glucosyl-derived adenosine **11** in 32% yield over three steps.

**Scheme 3:**
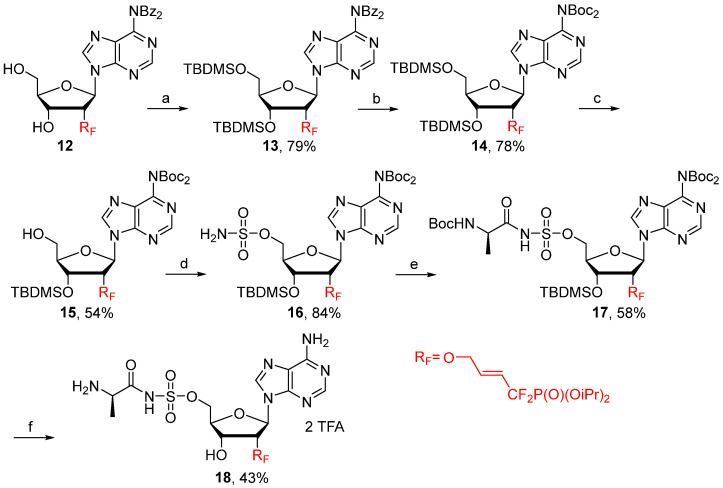
Reagents and conditions: (a) TBDMSCl, DMAP, Imidazole, DMF, 20 °C, 24 h. (b) (i) MeNH_2_, EtOH, 20 °C, 30 min. (ii) (Boc)_2_O, Et_3_N, DMAP, DMF, 20 °C, 12 h. (c) AcOH, H_2_O, THF, 20 °C, 64 h. (d) (i) NH_2_SO_2_Cl, DMAc, MeCN, 20 °C, 2 h. (ii) NEt_3_, MeOH, 0 °C, 10 min. (e) Boc-D-Ala-OSu, DBU, DMF, 20 °C, 24 h. (f) TFA, H_2_O, CH_2_Cl_2_, 20 °C, 24 h.

The last 2′-modification realized was the introduction of a difluorophosphonylated allylic ether moiety that has already been used by our group to improve the metabolic stability and lipophilicity of homouridylates [32]. The synthesis of the corresponding fluorinated *N*-acylsulfamate adenosine **18** was accomplished in six steps starting from **12** [33] (Scheme 3). Nucleoside **13**, easily obtained from **12**, was first treated with methylamine, followed by an excess of Boc_2_O to furnish **14** in 78% yield. After selective 5′-deprotection occurring in TFA/H_2_O, the corresponding alcohol **15** was sulfamoylated with NH_2_SO_2_Cl and then engaged in a coupling reaction with Boc-D-Ala-OSu in the presence of DBU to afford **17**, which was converted into the desired nucleoside **18** after a final deprotection conducted with TFA.

**Scheme 4:**
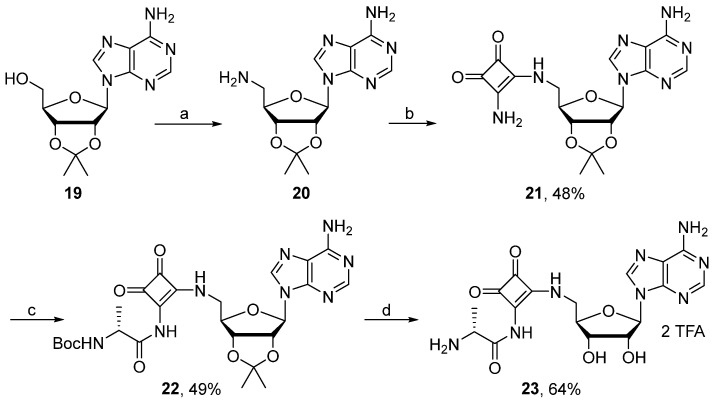
Reagents and conditions: (a) *See* ref [36,37] for reagents and conditions. (b) (i) Dimethyl squarate, MeOH, 20 °C, 4 h. (ii) NH_3_, MeOH, 20 °C, 8 h. (c) Boc-D-Ala-OSu, DBU, DMF, 60 °C, 4 h. (d) TFA, H_2_O, CH_2_Cl_2_, 20 °C, 5 h.

It was noticed that *N*-acylsulfamate adenosines could easily decompose into their corresponding cyclonucleosides *via* a nucleophilic substitution between the adenine *N*^3^-atom and the 5′-sulfamoyl group [34,35]. To avoid this competitive reaction, the bridge modification was investigated. Substitution of the acylsulfamate linkage by a stable isosteric acylphosphate mimic such as acylsquaramide was first envisaged (Scheme 4). Indeed, protected nucleoside **19** was first activated with diphenylphosphoryl azide and then reacted with sodium azide to provide **20** after hydrogenation [36,37]. Squaramide **21**, easily obtained from **20** under standard conditions [21], was treated with activated alanylester in the presence of DBU. After 4 h of stirring at 60 °C in DMF, nucleoside **22** was isolated in 49% yield. Additional treatment with TFA produced the ammonium salt **23** in good yield.

**Scheme 5:**
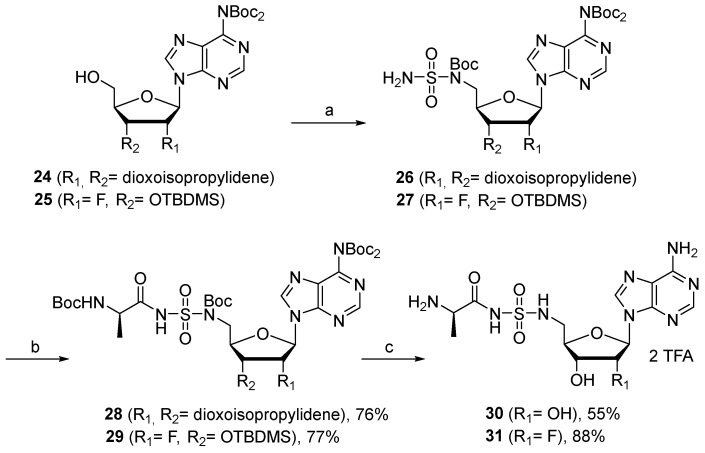
Reagents and conditions: (a) *See* ref [20] for reagents and conditions. (b) Boc-D-Ala-OSu, DBU, DMF, 20 °C, 16 h for **25** and 48 h for **26**. (c) TFA, H_2_O, CH_2_Cl_2_, 20 °C, 5 h for **27** and 10 h for **28**.

Substitution of the sulfamate oxygen with a nitrogen, leading to the more stable corresponding acylsulfamide derivatives [38], was also considered starting from **24** and **25**, easily prepared according to reported procedures [20] (Scheme 5). Compound **26** was reacted with Boc-D-Ala-OSu in the presence of DBU to produce, after 16 h of stirring in DMSO, nucleoside **28** in 76% yield. Under the same conditions, the reaction with **27** was slower and reached completion after 48 h. In this case, 2′-fluoroadenosine **29** was isolated in 77% yield. *N*-acylsulfamide adenosine **30** and 2′-fluoro *N*-acylsulfamide adenosine **31** were finally obtained after Boc and TBDMS deprotection with TFA.

**Scheme 6:**
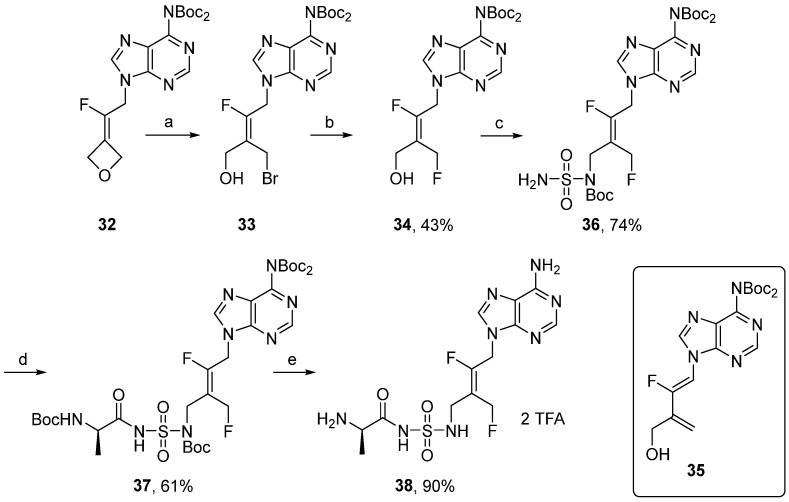
Reagents and conditions: (a) *See* ref [40] for reagents and conditions. (b) CsF, *t*BuOH, 70 °C, 4 h. (c) NH_2_SO_2_NHBoc, PPh_3_, DIAD, THF, 20 °C, 16 h. (d) Boc-D-Ala-OSu, DBU, DMF, 20 °C, 16 h. (e) TFA, H_2_O, CH_2_Cl_2_, 20 °C, 5h.

Agrofoglio showed that acyclonucleosides containing a transbutenyl motif were recognized by kinase as nucleoside mimics, and we recently reported that the fluorinated transbutenyl moiety can be used as stable nucleoside sugar surrogates [39,40]. Synthesis of *N*-acylsulfamides **38** was investigated to access a mimic of fluorinated nucleoside **8** (Scheme 6). The synthesis started from bromo-alcohol **33**, easily obtained from **32** by oxetane ring-opening reaction [40]. This later was first treated with cesium fluoride to give the corresponding acyclonucleoside **34** in 17% yield. In this case, a competitive elimination reaction occurred, leading to diene **35** as a major product. The same results were obtained with KF and TBAF with or without the presence of 18-crown-6. To overcome this limitation, *tert*-butanol was used as a solvent in order to balance the basic character of the fluoride ion and emphasize its nucleophilicity [41,42]. Under these conditions, ^19^F NMR analysis of the crude mixture revealed the presence of 92% of **34** and 8% of **35**, affording pure **34** in 43% yield. Compound **34** was next reacted with *tert*-butylsulfamoylcarbamate in the presence of PPh_3_ and DIAD. After 16 h of stirring at room temperature in THF, the corresponding fluoroalkene **36** was isolated in 74% yield. The desired acyclonucleoside **38** was finally obtained in two steps involving a coupling reaction with Boc-D-Ala-OSu, followed by a deprotection.

**Scheme 7:**
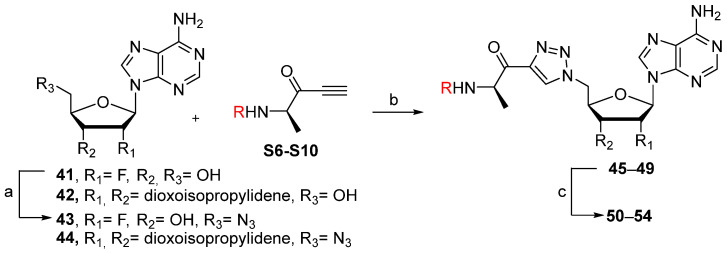
Reagents and conditions: (a) *See* ref [43,44] for reagents and conditions. (a) CuSO_4_.5H_2_O, Sodium Ascorbate, *t*-BuOH/H_2_O, 40 °C, 24 h. (b) TFA, H_2_O, CH_2_Cl_2_, 20 °C, 5 h for **44** or HCl_aq_ (3 M), MeOH, 20 °C, 20 h for **45**–**48**.

We showed that the introduction of a triazole ring to link D-alanine to adenosine or its fluorinated transbutenyl mimic was not detrimental for the inhibitory activity *in vitro* towards the DltA enzyme [40]. Indeed, nucleosides **39** and **40** were previously prepared (Scheme 7, Table 1, entry 1) and exhibited IC_50_ values in the same order of magnitude as those obtained from **1** [40]. To extend this series, analogs **50**–**54** were synthesized starting from **43** and **44**, easily prepared following reported procedures [43,44] (Table 1). Indeed, azide **43** was reacted with 4-*N*-Boc-amino-pent-1-yn-2-one in the presence of CuSO_4_ and sodium ascorbate. After 24 h of stirring at 20 °C in *t*-BuOH/H_2_O, triazole **45** was isolated in 68% yield. Additional treatment with TFA afforded **50** in 88% yield (Table 1, entry 2). Sulfamate substitution by a triazole ring removes the zwitterionic form of the 5′-chain found with compound **1** in physiological media. To restore this neutral character, we envisaged functionalizing the amino group of the triazolyl moiety with various lipophilic acyl derivatives, which are also known to improve cell permeability in several bacteria [45]. These modifications will also enrich structure-activity studies and establish if the functionalization of the amino-group of D-Ala will affect recognition by the enzyme and the activity. To rapidly access these compounds, 1,3-dipolar cycloaddition reactions between azide **44** and keto-alkynes containing either a biphenyl, heptyl, hexyloxyphenyl or a 4-nitrobenzyl chain were chosen as the key step (Table 1, entries 3–6 and *see* SI). Under the same previous conditions (24 h at 20 °C), low conversions were observed, and 24 h of stirring at 40 °C was necessary to drive the reaction until completion. Indeed, triazoles **46**–**49** were obtained in good yield and then treated with HCl or TFA to produce the corresponding deprotected nucleosides **51**–**54** in 76–83% yield.

### 2.2. Molecular Docking and Biological Studies

**Figure 3:**
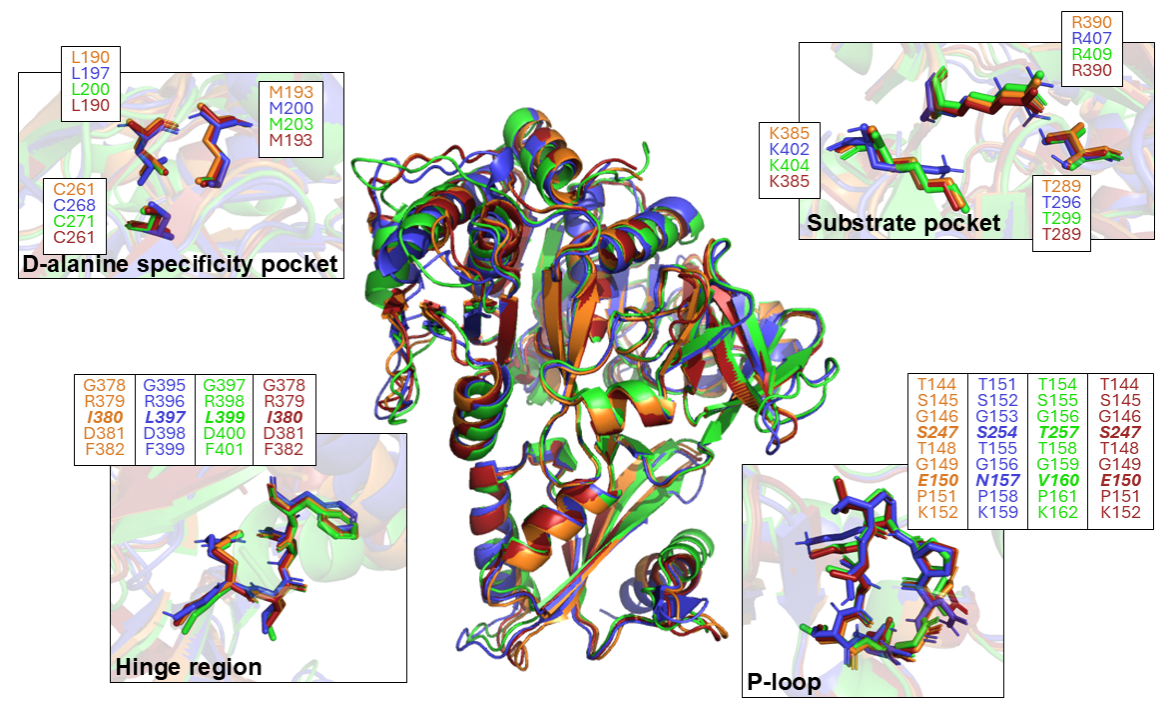
Structural superposition of DltA from *B. subtilis* MR168 (blue), *S. epidermidis* ATCC 35984 (orange), *S. aureus* Mu50 (dark red), and *E. faecalis* V583 (green). The structure of DltA from *S. epidermidis*, *S. aureus*, and *E. faecalis* was predicted using AlphaFold Server [46]. The structure of DltA from *B. subtilis* was previously determined by Yonus [47]. The aa that constitute each region are indicated in the boxes. Residues in bold italic indicate the aa differing between the four structures.

Nucleoside **1** has already been reported to effectively inhibit the growth of *Bacillus subtilis* in the presence of vancomycin by acting as a DltA inhibitor (K*i* = 0.2 μM), disrupting the D-alanylation process [16]. To validate the various proposed chemical modifications, the *in silico* binding affinity of each molecule was measured towards *B. Subtilis* DltA (PDB 3FCC) and compared to the one obtained with **1**. Docking scores listed in Table 2 are the average of nine different minimized energy states. Functionalization of the 2′-position did not disturb the binding affinity, which even seemed to increase with the size of the functional group. In fact, 2′-F and 2′-azido derivatives **8** (−8.2 kcal/mol) and **9** (−8.5 kcal/mol) showed binding affinities similar to **1** (−8.2 kcal/mol) while the introduction of bulky chains led to docking scores of −8.8 kcal/mol for **18** and −9.7 kcal/mol for **11** (Table 2, entries 1–5). Substitution of the sulfamate linkage by either a squaramide, a sulfamide or a triazole resulted in similar or slightly increased binding affinities with docking scores of −8.2 kcal/mol for **30**, −8.7 kcal/mol for **23**, and −8.9 kcal/mol for **40** (Table 2, entries 6, 7, and 11). *N*-terminal moiety functionalization of triazole **40** improved affinity, resulting in binding energies ranging from −8.9 kcal/mol to −10.5 kcal/mol (Table 2, entries 13–16). When the nucleosidic sugar pocket is replaced by the fluorinated transbutenyl moiety, the binding affinity of **38** (−7.7 kcal/mol) and **39** (−8.1 kcal/mol) was still close to **1** (Table 2, entries 1, 9, and 10). To ensure these docking results could be extended to *E. faecalis*, *S. aureus*, and *S. epidermidis*, we compared the 3D structures of DltA from these four bacteria (Figure 3). Structural superposition indicated a RMSD ranging from 2.25 Å (480 aa aligned) to 0.790 Å (387 aa aligned) (*B. subtilis* vs. *S. epidermidis*), from 4.13 Å (476 aa aligned) to 0.859 Å (368 aa aligned) (*B. subtilis* vs. *E. faecalis*), and from 3.45 Å (479 aa aligned) to 0.787 Å (377 aa aligned) (*B. subtilis* vs. *S. aureus*). This suggested that the overall structure was well conserved. Regarding the ATP and D-alanine pockets, all the structures were perfectly homologous and exhibited the same spatial distribution. These results of structural homologies supported the idea that the proposed chemical modifications should not disturb the molecular recognition of the different inhibitors by DltA from *E. faecalis*, *S. aureus*, and *S. epidermidis*.

All new compounds were then evaluated *in vitro* for enzyme inhibition against recombinant DltA from *E. faecalis* or *S. epidermis*. Half-maximal inhibitory concentrations (IC_50_) were then determined by measuring the amount of PPi released at different inhibitor concentrations (Table 2). Our results showed that the IC_50_ values of compound **1** against DltA from *E. faecalis* and *S. epidermidis* were similar, with values of 2.3 μM and 2.4 μM, respectively (Table 2, entry 1). The adenosine 2′-hydroxyl did not seem to be involved in the enzymatic recognition since its substitution by a fluorine atom, leading to compound **8**, showed the same activity as **1** (IC_50_ = 2.4 μM) with an IC_50_ value of 2.6 μM (Table 2, entries 1 and 2). We noticed the DltA enzyme tolerated the presence of various modifications on the 2′-position, but the activity was inversely related to the size of the introduced group. Indeed, compounds **9** (IC_50_ = 3.8 μM) and **18** (IC_50_ = 7.6 μM), containing either an azide or a difluorophosphonylated allylic ether, were respectively 1.6 and 3 times less active than **1** while the activity of the glucose derivative **11** (IC_50_ = 39.5 μM) dropped 16-fold (Table 2, entries 1, 3–5). Substitution of the sulfamate linkage by a squaramide drastically decreased the enzyme activity with an IC_50_ value of 28.4 μM for **23** (Table 2, entry 6). Despite a good predicted binding affinity (−8.7 Kcal/mol), we hypothesize that the lower activity could be explained by the fact that the squaramide nitrogen atom might not be under its anionic form, as is the case with sulfamate **1**. Compounds **30** (IC_50_ = 1.8 μM) and **31** (IC_50_ = 3.2 μM), both containing the more acidic sulfamide linker instead of the squaramide, were as efficient as **1** (Table 2, entries 7 and 8). Surprisingly, when this linkage was introduced onto the fluorinated transbutenyl derivative **38**, a complete loss of the activity was observed (Table 2, entry 9). This result contrasted with our previous reported work in which triazolyl transbutenyl derivative **39** (IC_50_ = 7.5 μM) was found to inhibit DltA in the same order of magnitude as its corresponding nucleosidic analogue **40** (Table 2, entries 10 and 11) [40]. In this triazolyl series, the introduction of a fluorine atom onto the 2′-position did not improve the enzymatic activity (Table 2, entries 11 and 12). Instead, fluorinated compound **50** (IC_50_ = 14.7 μM) was three times less active than non-fluorinated derivative **40** (IC_50_ = 4.5 μM). The presence of the amino group at the 5′-end seemed to be necessary since **51**–**54** were three to five times less active than the amine-free derivative **40** (Table 2, entries 11, 13–16).

All these compounds were also tested *in vivo* against MRSA clinical isolates. None of them had a significant effect on bacterial growth at concentrations up to 1 mM, thus excluding that they can act as antibiotics. The minimum inhibitory concentrations (MICs) of imipenem (IPM) were next determined in the absence or presence of 1 mM of the different DltA inhibitors to evaluate their potential to re-sensitize MRSA to this β-lactam (Table 2). In the absence of an inhibitor, the MIC value of IPM against the tested MRSA strain was 32 µg/mL, which is well above the clinical breakpoint of resistance of 2 µg/mL. Among the best *in vitro* Dlt inhibitors with IC_50_ values ranging from 1.8 to 7.6 μM, only **18** (MIC = 0.25 μg/mL) and **30** (MIC = 1 μg/mL) acted as efficient adjuvants of IPM with MICs comparable with those previously reported with **1** (MIC = 0.25 µg/mL) (Table 2, entries 1, 5 and 7) [17,18,19], while compounds **8**, **9**, **31**, **39**, and **40** were 32 to 128 times less active than **1** *in vivo* emphasizing their potential poor cell permeability or metabolic instability (Table 2, entries 2, 3, 8, 10, and 11). Compounds with moderate and weak inhibitory activity such as **11**, **23**, **38**, **50**, **51**, **53**, and **54** did not re-sensitize the bacteria to IPM since MIC values, ranging from 16 to 64 μg/mL, are above the breakpoint (Table 2, entries 4, 6, 9, 12, 13, 15, and 16). However, we observed that **52** (MIC = 8 μg/mL) slightly restored IPM antimicrobial activity even though this compound was weakly active towards DltA *in vitro* (Table 2, entry 14). This result seems to indicate that **52** was able to slightly potentiate IPM against MRSA, probably by targeting an enzyme other than DltA. To clarify this hypothesis, quantification of D-alanyl ester of the MRSA cell wall was performed as previously described in the presence of inhibitors (Table 2) [48]. At 1 mM, **40** (MIC = 8 μg/mL) did not affect D-alanylation while **52** (MIC = 8 μg/mL) and **9** (MIC = 8 μg/mL) reduced the level of bacteria cell wall D-alanyl ester residues by 16 % and 74.9%, respectively (Table 2, entries 3, 11 and 14). This clearly supports that the IPM antimicrobial activity observed with the presence of **40** was not related to D-alanylation inhibition, in contrast with **52** and **9**, which had an effect on this pathway. In addition to **9** (reduction of 74.9%), molecules exhibiting the best *in vivo* activities targeted the D-alanylation process to re-sensitize MRSA to IPM. Indeed, a reduction of 51.5% and 80.9% in D-alanyl residues was observed in the presence of compounds **18** and **30**, which were as efficient as **1** (reduction of 86.7%) (Table 2, entries 1, 5, and 7). These new series represent a promising structure entry not reported yet for the inhibition of DltA and the re-sensitization of bacteria toward known antibiotics.

## 3. Materials and Methods

### 3.1. General Information and Materials

Unless otherwise specified, all reagents were obtained from commercial suppliers and were used without purification. For anhydrous conditions, the glassware was flamed under a continuous nitrogen flow and cooled to room temperature before performing the experiment. Anhydrous solvents (THF, MeCN, Toluene, Et_2_O, and CH_2_Cl_2_) were obtained with PURE SOLV, Innovative Technology Solvent Purification System, by passing the degassed solvents (N_2_) through a column of activated alumina. Anhydrous Pyridine and DMF were distilled from CaH_2_. Flash column chromatography was performed on silica gel (40–63 µm), and thin layer chromatography plates were revealed by UV light and/or KMnO_4_ solution. ^1^H NMR, ^13^C NMR, ^31^P NMR, and ^19^F NMR spectra were recorded on 500 or 600 MHz apparatus in deuterated solvent. All NMR spectra were calibrated by the residual peaks of the deuterated solvent according to Hugo E. Gottlieb’s values [49]. ^19^F and ^31^P NMR spectral lines are with respect to the internal references CFCl_3_ and H_3_PO_4,_ respectively. All data are reported in the following order: chemical shifts (δ) in parts per million (ppm), multiplicity (s: singlet, d: doublet, t: triplet, q: quadruplet, m: multiplet, *br* s: broad signal), coupling constants (*J*) in Hertz (Hz), and number of protons. All proton and carbon assignments were realized by NMR methods involving DEPT-135, HSQC, HMBC, and COSY experiments. Mass spectra and high-resolution mass spectra (HRMS) were recorded on a Q-TOF (Quadrupole time-of-flight) micro instrument with an electrospray source in the ESI mode. Reversed phase preparative high-performance liquid chromatography was performed on Gemini^®^ 5 μm C18 110 Å, LC Column 250 × 10 mm, equipped with a diode array detector. The exact HPLC-RP method: buffer TFA 0.05%, flow rate 5 mL/min; solvent A/B (MeCN/H_2_O): 0–3 min: 5%A, 3–7 min: linear gradient 5–100%A, 7–13 min: 100%A.

### 3.2. General Procedure

General procedure A for the sulfamoylation: (Method A1) To a solution of alcohol (1.0 equiv.) in DMF (0.5 M) were added triethylamine (1.1 equiv.) and sulfamoyl chloride (2.4 equiv.) at 0 °C. The reaction mixture was stirred at 0 °C for 2 h and overnight at 20 °C. The mixture was diluted with EtOAc, then washed three times with H_2_O and once with brine. The organic layer was dried over MgSO_4_ and concentrated under reduced pressure to give the desired product. (Method A2) To a solution of alcohol (1.0 equiv.) in DMAc (0.6 M) was added a cooled solution of sulfamoyl chloride (2.4 equiv.) in MeCN (1.9 M) at 0 °C. The mixture was stirred for 2 h at 20 °C, then triethylamine (4.0 equiv.) with MeOH was added at 0 °C. The mixture was stirred for 10 min and concentrated under reduced pressure. The residue was taken up with EtOAc, and the organic layer was washed twice with an aqueous solution of NaHCO_3_ (1 M) and brine. The organic layer was dried over MgSO_4_, filtered, and concentrated under reduced pressure to give the desired product.

General procedure B for the amino acid introduction: To a solution of sulfamoyl derivative (1.0 equiv.) and activated alanine (1.0 equiv.) in DMF (0.1 M), 1,8-diazabicyclo[5.4.0]undec-7-ene (DBU) (1.5 equiv.) was added at 20 °C. The mixture was stirred for 16 h, then the solvent was evaporated under reduced pressure. The crude product was purified by column chromatography to give the desired product.

General procedure C for the nucleoside deprotection: (Method C1) To a solution of protected nucleoside analogue (1.0 equiv.) in MeOH (0.1 M), a solution of aqueous HCl (3 M) was added. The reaction mixture was stirred at 20 °C for 20 h, then the solvent was removed under reduced pressure. The residue was taken up with a small amount of MeOH and precipitated by the addition of Et_2_O. The desired product was isolated after filtration. Method C2: To a solution of protected nucleoside analogue (1.0 equiv.) in CH_2_Cl_2_/H_2_O (5:1), TFA was added, and the reaction mixture was stirred for 5 h at 20 °C. The solvent was removed under reduced pressure. The residue was taken up with a small amount of MeOH and precipitated by the addition of Et_2_O. The desired product was isolated after filtration.

General procedure D for the sulfamide introduction by Mitsunobu reaction: To a solution of alcohol (1.0 equiv.) in THF (0.05 M), *tert*-butyl sulfamoylcarbamate (3.0 equiv.) and triphenylphosphine (1.5 equiv.) were added. Then, diisopropyl azodicarboxylate (1.05 equiv.) was added dropwise. The mixture was stirred overnight at 20 °C and concentrated under reduced pressure. The crude mixture was purified by column chromatography to afford the desired product.

General procedure E for the copper-catalyzed azide-alkyne cycloaddition: To a solution of alkyl azide (1.0 equiv.) in *t*-BuOH/H_2_O (0.2 M, 1:1), alkyne (1.1 equiv.), followed by sodium ascorbate (10 mol%), was added, and CuSO_4_.5H_2_O (5 mol%) was added at 20 °C. The mixture was stirred for 24 h at 40 °C, then the solvent was removed under reduced pressure. The residue was taken up with CH_2_Cl_2_/H_2_O (1:1), and the aqueous layer was extracted three times with CH_2_Cl_2_. The combined organic layers were washed with brine, dried over MgSO_4_, filtered, and then concentrated under reduced pressure. The crude was purified by column chromatography to give the desired product.

General procedure F for the alcohol protection by silyl ether: To a solution of adenosine derivative (1.0 equiv.) in DMF (0.2 M), Imidazole (6.6 equiv.) and DMAP (0.1 equiv.) were added at 20 °C. TBDMSCl (3.3 equiv.) was then added by portion at 0 °C. The reaction mixture was stirred for 24 h at 20 °C, then quenched with an aqueous solution of NH_4_Cl. The aqueous layer was extracted three times with EtOAc, and the combined organic layers were washed with H_2_O and brine. The organic layer was dried over MgSO_4_, filtered, and concentrated under reduced pressure. The crude product was purified by column chromatography to give the desired product.

General procedure G for the selective deprotection of silyl ether: (Method G1) To a solution of protected nucleoside (1.0 equiv.) in H_2_O and THF (1:1), glacial acetic acid (3 *v*/*v*) was added at 0 °C. The mixture was stirred for 2 days at 20 °C, then quenched with a cooled aqueous solution of HCl (1 M). The aqueous layer was extracted three times with EtOAc, and the combined organic layers were washed once with brine. The organic layer was dried over MgSO_4_, filtered, and concentrated under reduced pressure. The crude product was purified by column chromatography to give the desired product. Method G2: To a solution of protected nucleoside (1.0 equiv.) in THF (4 *v*/*v*), an aqueous solution of TFA (1:1) was added at 0 °C. The mixture was stirred for 2 h at 0 °C, then quenched with a cooled aqueous solution of saturated NaHCO_3_. The aqueous layer was extracted three times with EtOAc, and the combined organic layers were washed once with H_2_O and brine. The organic layer was dried over MgSO_4_, filtered, and concentrated under reduced pressure. The crude product was purified by column chromatography to give the desired product.

### 3.3. Synthesis

((*2R*,*3R*,*4R*,*5R*)-5-(6-amino-9H-purin-9-yl)-3-((*tert*-butyldimethylsilyl)oxy)-4-fluorotetrahydrofuran-2-yl)methyl sulfamate (**4**). The general procedure A (Method A2) was followed with compound **2** (590 mg, 1.54 mmol), prepared as previously described [22], in DMAc (2.4 mL), sulfamoyl chloride (426 mg, 3.69 mmol) in MeCN (1.9 mL). The crude product was purified by column chromatography (Eluent CH_2_Cl_2_/MeOH 94:6) to give compound **4** (570 mg, 1.23 mmol, 80%) as a white solid. ^1^H NMR (CD_3_CN, 500 MHz): δ 0.00 (s, 3H), 0.04 (s, 3H), 0.84 (s, 9H), 3.87 (dd, *J* = 12.2 Hz, *J* = 3.4 Hz, 1H), 4.03 (dd, *J* = 12.2 Hz, *J* = 2.3 Hz, 1H), 4.30–4.33 (m, 1H), 5.51 (ddd, *J*_HF_ = 18.5 Hz, *J* = 7.2 Hz, *J* = 4.6 Hz, 1H), 5.76 (ddd, *J*_HF_ = 51.9 Hz, *J* = 4.6 Hz, *J* = 1.8 Hz, 1H), 6.05 (br s, 4H), 6.26 (dd, *J*_HF_ = 18.3 Hz, *J* = 1.8 Hz, 1H), 8.11 (s, 1H), 8.24 (s, 1H). ^19^F NMR (CD_3_CN, 470 MHz): δ −202.0 (dt, *J*_FH_ = 51.9 Hz, *J*_FH_ = 18.5 Hz). ^13^C NMR (CD_3_CN, 150 MHz): δ −5.4, −5.3, 19.0, 26.2, 61.8, 74.8 (d, *J*_CF_ = 14.8 Hz), 82.0, 87.9 (d, *J*_CF_ = 33.6 Hz), 92.4 (d, *J*_CF_ = 189.9 Hz), 120.7, 140.5, 150.3, 154.1, 157.0. HRMS-ESI (*m*/*z*) calcd for C_16_H_28_FN_6_O_5_SiS [M+H]^+^ 463.1595, found 463.1602. The NMR spectrum is identical to that previously reported [13].

((*2R*,*3S*,*4S*,*5R*)-5-(6-amino-9H-purin-9-yl)-4-azido-3-((*tert*-butyldimethylsilyl)oxy)tetrahydrofuran-2-yl)methyl sulfamate (**5**). The general procedure A (Method A2) was followed with compound **3** (250 mg, 0.61 mmol), prepared as previously described [23], in DMAc (1 mL), sulfamoyl chloride (170 mg, 1.47 mmol) in MeCN (0.75 mL) to give compound **5** (265 mg, 0.54 mmol, 89%) as a white solid. The compound was used in the next step without further purification. ^1^H NMR (MeOD, 500 MHz): δ 0.23 (s, 3H), 0.24 (s, 3H), 1.00 (s, 9H), 4.28–4.32 (m, 2H), 4.40–4.43 (m, 1H), 4.72–4.74 (m, 1H), 4.87–4.89 (m, 1H), 6.17 (d, *J* = 6.1 Hz, 1H), 8.23 (s, 1H), 8.30 (s, 1H). ^13^C NMR (MeOD, 125 MHz): δ −4.8, −4.6, 21.3, 26.3, 66.1, 68.8, 74.1, 84.6, 87.6, 120.6, 141.1, 150.6, 154.1, 157.5. HRMS-ESI (*m*/*z*) calcd for C_16_H_28_N_9_O_5_SSi [M+H]^+^ 486.1703, found 486.1710.

((*2R*,*3R*,*4R*,*5R*)-5-(6-amino-9H-purin-9-yl)-3-((*tert*-butyldimethylsilyl)oxy)-4-fluorotetrahydrofuran-2-yl)methyl ((*tert*-butoxycarbonyl)-D-alanyl)sulfamate (**6**). The general procedure B was followed with compound **4** (375 mg, 0.81 mmol), Boc-D-Ala-OSu (232 mg, 0.81 mmol), and DBU (0.18 mL, 1.22 mmol) in DMF (10.8 mL). The crude was purified by column chromatography (Eluent CH_2_Cl_2_/MeOH 94:6 to 9:1) to give compound **6** (392 mg, 0.61 mmol, 76%) as a white foam. ^1^H NMR (DMSO-d_6_, 600 MHz): δ −0.02 (s, 3H), 0.00 (s, 3H), 0.82 (s, 9H), 1.17 (d, *J* = 7.1 Hz, 3H), 1.33 (s, 9H), 3.75–3.83 (m, 2H), 3.91–3.95 (m, 1H), 4.16–4.20 (m, 1H), 4.37–4.46 (m, 1H), 5.66 (d, *J*_HF_ = 52.1 Hz, 1H), 6.27 (dd, *J*_HF_ = 17.3 Hz, *J* = 2.3 Hz, 1H), 6.46 (br s, 1H), 7.40 (br s, 2H), 8.14 (s, 1H), 8.27 (s, 1H). ^19^F NMR (DMSO-d_6_, 565 MHz): δ −201.5 (m). ^13^C NMR (DMSO-d_6_, 150 MHz): δ −5.6, −5.5, 18.1, 18.5, 25.8, 28.2, 51.3, 62.2, 74.0, 77.8, 81.5, 85.7 (d, *J*_CF_ = 32.2 Hz), 91.2 (d, *J*_CF_ = 190.2 Hz), 119.0, 139.1, 148.8, 152.7, 154.8, 156.1, 176.1. HRMS-ESI (*m*/*z*) calcd for C_24_H_41_FN_7_O_8_SiS [M+H]^+^ 634.2491, found 634.2489.

((*2R*,*3S*,*4S*,*5R*)-5-(6-amino-9H-purin-9-yl)-4-azido-3-((*tert*-butyldimethylsilyl)oxy)tetrahydrofuran-2-yl)methyl ((*tert*-butoxycarbonyl)-D-alanyl)sulfamate (**7**). The general procedure B was followed with compound **5** (438 mg, 0.90 mmol), Boc-D-Ala-OSu (258 g, 0.90 mmol), and DBU (0.20 mL, 1.35 mmol) in DMF (12 mL). The crude was purified by column chromatography (Eluent EtOAc with 1% AcOH) to give compound **7** (408 mg, 0.62 mmol, 69%) as a white foam. ^1^H NMR (CD_3_CN, 600 MHz): δ 0.18 (s, 3H), 0.19 (s, 3H), 0.96 (s, 9H), 1.27 (d, *J* = 7.1 Hz, 3H), 1.37 (s, 9H), 4.00–4.02 (m, 1H), 4.30–4.32 (m, 1H), 4.44 (dd, *J* = 11.2 Hz, *J* = 3.7 Hz, 1H), 4.55–4.60 (m, 2H), 4.78–4.79 (m, 1H), 5.82 (br s, 1H), 6.09 (d, *J* = 7.1 Hz, 1H), 8.34 (s, 1H), 8.38 (s, 1H). ^13^C NMR (CD_3_CN, 150 MHz): δ −4.7, −4.6, 18.6, 19.6, 26.1, 28.6, 53.7, 66.5, 68.1, 73.8, 79.7, 84.5, 86.5, 120.2, 141.1, 150.6, 154.1, 156.5, 156.7, 171.7. HRMS-ESI (*m*/*z*) calcd for C_24_H_41_N_10_O_8_SSi [M+H]^+^ 657.2599, found 657.2601.

((*2R*,*3R*,*4R*,*5R*)-5-(6-amino-9H-purin-9-yl)-4-fluoro-3-hydroxytetrahydrofuran-2-yl)methyl (D-alanyl)sulfamate salt (**8**). The general procedure C (Method C2) was followed with compound **6** (498 mg, 0.78 mmol), TFA (9.7 mL), and CH_2_Cl_2_/H_2_O (8/2 mL) to give compound **8** (450 mg, 0.69 mmol, 89%) as a white solid (2 TFA salt). ^1^H NMR (D_2_O, 500 MHz): δ 1.52 (d, *J* = 7.1 Hz, 3H), 3.81 (dd, *J* = 13.1 Hz, *J* = 3.7 Hz, 1H), 3.89 (q, *J* = 7.1 Hz, 1H), 3.98 (dd, *J* = 13.1 Hz, *J* = 2.3 Hz, 1H), 4.42–4.46 (m, 1H), 5.51 (ddd, *J*_HF_ = 17.2 Hz, *J* = 7.6 Hz, *J* = 4.8 Hz, 1H), 5.76 (ddd, *J*_HF_ = 51.1 Hz, *J* = 4.8 Hz, *J* = 2.0 Hz, 1H), 6.51 (dd, *J*_HF_ = 18.4 Hz, *J* = 2.0 Hz, 1H), 8.45 (s, 1H), 8.51 (s, 1H). ^19^F NMR (D_2_O, 470 MHz): δ −200.8 (dt, *J*_FH_ = 51.1 Hz, *J*_FH_ = 17.6 Hz). ^13^C NMR (D_2_O, 150 MHz): δ 51.3, 59.2, 73.8 (d, *J*_CF_ = 14.9 Hz), 81.5, 87.3 (d, *J*_CF_ = 34.8 Hz), 91.4 (d, *J*_CF_ = 191.9 Hz), 116.2 (q, *J*_CF_ = 291.4 Hz), 117.2, 119.0, 143.3, 144.6, 147.9, 150.0, 162.9 (q, *J*_CF_ = 35.3 Hz, TFA), 176.4. HPLC purity: (96.6%, t_R_ = 4.48 min). HRMS-ESI (*m*/*z*) calcd for C_13_H_18_FN_7_O_6_S [M+H]^+^ 420.1102, found 420.1102.

((*2R*,*3S*,*4S*,*5R*)-5-(6-amino-9H-purin-9-yl)-4-azido-3-hydroxytetrahydrofuran-2-yl)methyl (D-alanyl)sulfamate salt (**9**). To a solution of compound **7** (165 mg, 0.25 mmol, 1 equiv.) in H_2_O (2.5 mL) was added TFA (10 mL) at 20 °C. The mixture was stirred for 12 h and concentrated under reduced pressure. The residue was taken up with a small amount of MeOH. By addition of Et_2_O, a precipitate appeared, which was isolated by filtration to give compound **9** (91 mg, 0.13 mmol, 54%) as a white solid (2 TFA salt). ^1^H NMR (DMSO-d_6_, 500 MHz): δ 1.29 (d, *J* = 7.1 Hz, 3H), 3.48–3.50 (m, 1H), 4.07 (dd, *J* = 11.1 Hz, *J* = 4.3 Hz, 1H), 4.14–4.17 (m, 1H), 4.19 (dd, *J* = 11.1 Hz, *J* = 3.7 Hz, 1H), 4.57–4.59 (m, 1H), 4.67–4.69 (m, 1H), 6.06 (d, *J* = 6.1 Hz, 1H), 6.17 (d, *J* = 5.4 Hz, 1H), 7.35 (br s, 1H), 7.81 (br s, 2H), 8.16 (s, 1H), 8.39 (s, 1H). ^13^C NMR (DMSO-d_6_, 150 MHz): δ 17.2, 50.8, 64.2, 67.2, 71.5, 83.0, 84.8, 118.9, 139.3, 149.3, 152.9, 156.1, 173.4. HPLC purity: (94.9%, t_R_ = 4.59 min). HRMS-ESI (*m*/*z*) calcd for C_13_H_19_N_10_O_6_S [M+H]^+^ 443.1210, found 443.1208.

(*2R*,*3R*,*4S*,*5R*,*6R*)-2-(acetoxymethyl)-6-((1-((*2R*,*3R*,*4S*,*5R*)-2-(6-amino-9H-purin-9-yl)-5-(((N-((*tert*-butoxycarbonyl)-D-alanyl)sulfamoyl)oxy)methyl)-4-((*tert*-butyldimethylsilyl)oxy)tetrahydrofuran-3-yl)-1H-1,2,3-triazol-4-yl)methoxy)tetrahydro-2H-pyran-3,4,5-triyl triacetate (**10**). The general procedure E was followed with compound **7** (269 mg, 0.41 mmol), glucosyl-*O*-propargyl ether (175 mg, 0.45 mmol), prepared as previously described [50], sodium ascorbate (8 mg, 0.04 mmol) and CuSO_4_.5H_2_O (5 mg, 0.02 mmol) in *t*-BuOH/H_2_O (4 mL). The crude was purified by column chromatography (Eluent EtOAc/MeOH 9:1) to give compound **10** (276 mg, 0.26 mmol, 64%) as a white solid. ^1^H NMR (CD_3_CN, 600 MHz): δ −0.18 (s, 3H), 0.01 (s, 3H), 0.73 (s, 9H), 1.23 (d, *J* = 7.4 Hz, 3H), 1.33 (s, 9H), 1.85 (s, 3H), 1.92 (s, 3H), 1.97 (s, 3H), 2.02 (s, 3H), 3.82 (ddd, *J* = 10.1 Hz, *J* = 4.7 Hz, *J* = 2.5 Hz, 1H), 3.98 (br s, 1H), 4.10 (dd, *J* = 12.4 Hz, *J* = 2.5 Hz, 1H), 4.25 (dd, *J* = 12.4 Hz, *J* = 4.7 Hz, 1H), 4.28 (dd, *J* = 10.4 Hz, *J* = 3.6 Hz, 1H), 4.38–4.39 (m, 1H), 4.44–4.46 (m, 1H), 4.68 (d, *J* = 12.4 Hz, 1H), 4.71 (d, *J* = 8.1 Hz, 1H), 4.81 (d, *J* = 12.4 Hz, 1H), 4.85–4.88 (m, 2H), 5.02 (t, *J* = 9.7 Hz, 1H), 5.18 (t, *J* = 9.7 Hz, 1H), 5.78 (br s, 1H), 5.96 (br s, 1H), 6.28 (br s, 2H), 6.80 (d, *J* = 5.6 Hz, 1H), 7.94 (s, 1H), 8.19 (s, 1H), 8.39 (br s, 1H). ^13^C NMR (CD_3_CN, 150 MHz): δ −4.9, −4.8, 18.3, 19.7, 20.8, 20.9 (2C), 21.0, 25.9, 28.6, 53.7, 62.7, 62.9, 66.2, 68.2, 69.2, 71.9, 72.5, 72.8, 73.4, 79.7, 85.1, 86.3, 100.3, 120.3, 126.0, 141.0, 144.3, 150.6, 154.1, 156.4, 156.8, 170.2, 170.5, 170.9, 171.4, 182.0. HRMS-ESI (*m*/*z*) calcd for C_41_H_63_N_10_O_18_SSi [M+H]^+^ 1043.3812, found 1043.3835.

((*2R*,*3S*,*4R*,*5R*)-5-(6-amino-9H-purin-9-yl)-3-hydroxy-4-(4-((((*2R*,*3R*,*4S*,*5S*,*6R*)-3,4,5-trihydroxy-6-(hydroxymethyl)tetrahydro-2H-pyran-2-yl)oxy)methyl)-1H-1,2,3-triazol-1-yl)tetrahydrofuran-2-yl)methyl (D-alanyl)sulfamate salt (**11**). A solution of compound **10** (70 mg, 0.06 mmol, 1 equiv.) in H_2_O (2 mL) and TFA (8 mL) was stirred for 12 h at 20 °C. Volatiles were removed under reduced pressure, and the residue was dissolved in MeOH (1 mL), and a solution of MeONa (5.4 M) in MeOH (0.05 mL, 0.3 mmol, 5 equiv.) was added at 20 °C. The mixture was stirred for 1 h and quenched with an aqueous solution of TFA (10%), then concentrated under reduced pressure. The residue was dissolved in THF (1 mL), and a solution of 37% triethylamine trihydrofluoride (0.1 mL, 0.24 mmol, 4 equiv.) was added at 20 °C. The mixture was stirred for 12 h, and volatiles were removed under reduced pressure. The crude was purified by HPLC RP-18 (MeCN/H_2_O, linear gradient) to give compound **11** (17 mg, 0.26 mmol, 32%) as a white solid (2 TFA salt). ^1^H NMR (D_2_O, 600 MHz): δ 1.48 (d, *J* = 7.2 Hz, 3H), 3.24–3.26 (m, 1H), 3.33–3.37 (m, 1H), 3.41–3.45 (m, 2H), 3.66 (dd, *J* = 12.7 Hz, *J* = 6.3 Hz, 1H), 3.85–3.87 (m, 1H), 3.89–3.93 (m, 1H), 4.46–4.48 (m, 1H), 4.51–4.57 (m, 2H), 4.62–4.64 (m, 1H), 4.88 (d, *J* = 12.7 Hz, 1H), 4.99 (d, *J* = 12.7 Hz, 1H), 5.05 (t, *J* = 5.6 Hz, 1H), 5.99 (t, *J* = 5.6 Hz, 1H), 6.99 (d, *J* = 4.9 Hz, 1H), 8.26 (s, 1H), 8.39 (s, 1H), 8.60 (s, 1H). ^13^C NMR (D_2_O, 150 MHz): δ 16.3, 51.0, 60.6, 61.7, 65.8, 68.3, 69.5, 69.8, 72.9, 75.6, 75.9, 83.2, 86.3, 101.4, 116.2 (q, *J*_CF_ = 291.1 Hz), 119.2, 126.6, 142.7, 143.7, 144.6, 148.1, 149.9, 162.9 (q, *J*_CF_ = 35.8 Hz), 175.4. HPLC purity: (88.5%, t_R_ = 2.42 min). HRMS-ESI (*m*/*z*) calcd for C_22_H_33_N_10_O_12_S [M+H]^+^ 661.2000, found 661.2016.

diisopropyl ((*E*)-4-(((*2R*,*3R*,*4R*,*5R*)-2-(6-(*N*-benzoylbenzamido)-9H-purin-9-yl)-4-((*tert*-butyldimethylsilyl)oxy)-5-(((*tert*-butyldimethylsilyl)oxy)methyl)tetrahydrofuran-3-yl)oxy)-1,1-difluorobut-2-en-1-yl)phosphonate (**13**). The general procedure F was followed with compound **12** (320 mg, 0.44 mmol), prepared as previously described [33], TBDMSCl (218 mg, 1.45 mmol), imidazole (196 mg, 2.89 mmol), and DMAP (4.9 mg, 0.04 mmol) in DMF (5 mL). The crude product was purified by column chromatography (Eluent Pentane/EtOAc 7:3) to give compound **13** (333 mg, 0.35 mmol, 79%) as a white foam. ^1^H NMR (CDCl_3_, 500 MHz): δ 0.06 (s, 3H), 0.08 (s, 3H), 0.11 (s, 6H), 0.90 (s, 9H), 0.92 (s, 9H), 1.33 (t, *J* = 6.2 Hz, 6H), 1.35 (dd, *J* = 6.2 Hz, *J*_HP_ = 1.7 Hz, 6H), 3.78 (dd, *J* = 11.7 Hz, *J* = 3.1 Hz, 1H), 3.98 (dd, *J* = 11.7 Hz, *J* = 3.4 Hz, 1H), 4.13 (dt, *J* = 5.7 Hz, *J* = 3.1 Hz, 1H), 4.22–4.31 (m, 2H), 4.36 (dd, *J* = 5.7 Hz, *J* = 3.6 Hz, 1H), 4.53–4.55 (m, 1H), 4.79–4.86 (m, 2H), 5.95–6.03 (m, 1H), 6.18 (d, *J* = 3.6 Hz, 1H), 6.26–6.33 (m, 1H), 7.33–7.36 (m, 4H), 7.46–7.49 (m, 2H), 7.84–7.86 (m, 4H), 8.37 (s, 1H), 8.65 (s, 1H). ^19^F NMR (CDCl_3_, 565 MHz): δ −109.5 (ddd, *J*_FF_ = 298.3 Hz, *J*_FP_ = 113.2 Hz, *J*_FH_ = 12.4 Hz), −110.3 (ddd, *J*_FF_ = 298.3 Hz, *J*_FP_ = 113.2 Hz, *J*_FH_ = 12.4 Hz). ^31^P NMR (CDCl_3_, 243 MHz): δ 4.3 (t, *J*_PF_ = 113.2 Hz). ^13^C NMR (CDCl_3_, 150 MHz): δ −5.3, −5.2, −4.7, −4.5, 18.2, 18.6, 23.8 and 23.9 (d, *J*_CP_ = 4.1 Hz), 24.3 (d, *J*_CP_ = 3.4 Hz, 2C), 25.8, 26.2, 61.8, 69.3, 69.8, 73.9 (d, *J*_CP_ = 7.1 Hz, 2C), 82.1, 85.1, 87.3, 116.8 (dt, *J*_CF_ = 258.9 Hz, *J*_CP_ = 220.7 Hz), 122.7 (dt, *J*_CF_ = 22.3 Hz, *J*_CP_ = 13.4 Hz), 128.3, 128.8 (4C), 129.6 (4C), 133.1 (2C), 134.2 (2C), 134.4 (dt, *JCF* = 9.8 Hz, *JCP* = 5.6 Hz), 143.7, 151.9, 152.3, 152.7, 172.4. HRMS-ESI (*m*/*z*) calcd for C_46_H_67_F_2_N_5_O_9_PSi_2_ [M+H]^+^ 958.4183, found 958.4179.

*tert*-butyl (*tert*-butoxycarbonyl)(9-((*2R*,*3R*,*4R*,*5R*)-4-((*tert*-butyldimethylsilyl)oxy)-5-(((*tert*-butyldimethylsilyl)oxy)methyl)-3-(((*E*)-4-(diisopropoxyphosphoryl)-4,4-difluorobut-2-en-1-yl)oxy)tetrahydrofuran-2-yl)-9H-purin-6-yl)carbamate (**14**). To a solution of compound **13** (300 mg, 0.44 mmol, 1 equiv.) in EtOH (3.5 mL), CH_3_NH_2_ (2.1 mL, 33% in EtOH) was added at 20 °C. The mixture was stirred for 1 h and followed by TLC. When the conversion was quantitative, the solvent was removed under reduced pressure. It was dissolved in DMF (4 mL), and triethylamine (0.17 mL, 1.20 mmol, 3 equiv.) and DMAP (5 mg, 0.04 mmol, 0.5 equiv.) were added at 20 °C. The solution was cooled, and (Boc)_2_O (0.27 mL, 1.20 mmol, 3 equiv.) was added dropwise at 0 °C. The reaction mixture was stirred for 12 h and quenched with an aqueous solution of HCl (1 M). The aqueous layer was extracted three times with EtOAc. The combined organic layers were washed with brine, dried over MgSO_4_, filtered, and concentrated under reduced pressure. The crude product was purified by column chromatography (Eluent Pentane/EtOAc 7:3) to give compound **14** (326 mg, 0.34 mmol, 78%) as a waxy oil. ^1^H NMR (CDCl_3_, 600 MHz): δ 0.07 (s, 3H), 0.08 (s, 3H), 0.12 (s, 3H), 0.13 (s, 3H), 0.91 (s, 9H), 0.94 (s, 9H), 1.33 (dd, *J* = 6.4 Hz, *J*_HP_ = 4.3 Hz, 6H), 1.36 (dd, *J* = 6.4 Hz, *J*_HP_ = 1.9 Hz, 6H), 1.45 (s, 18H), 3.81 (dd, *J* = 11.6 Hz, *J* = 2.6 Hz, 1H), 4.05 (dd, *J* = 11.6 Hz, *J* = 3.4 Hz, 1H), 4.16 (dt, *J* = 6.3 Hz, *J* = 2.6 Hz, 1H), 4.24 (dd, *J* = 4.7 Hz, *J* = 3.1 Hz, 1H), 4.26–4.30 (m, 1H), 4.36–4.40 (m, 1H), 4.52 (dd, *J* = 6.3 Hz, *J* = 4.7 Hz, 1H), 4.80–4.86 (m, 2H), 5.98–6.06 (m, 1H), 6.22 (d, *J* = 3.1 Hz, 1H), 6.29–6.33 (m, 1H), 8.54 (s, 1H), 8.84 (s, 1H). ^19^F NMR (CDCl_3_, 565 MHz): δ −109.6 (ddd, *J*_FF_ = 298.4 Hz, *J*_FP_ = 112.4 Hz, *J*_FH_ = 12.2 Hz), −110.2 (ddd, *J*_FF_ = 298.4 Hz, *J_FP_* = 112.4 Hz, *J_FH_* = 12.2 Hz). ^31^P NMR (CDCl_3_, 243 MHz): δ 4.3 (t, *J*_PF_ = 112.4 Hz). ^13^C NMR (CDCl_3_, 150 MHz): δ −5.3, −5.2, −4.8, −4.5, 18.2, 18.6, 23.8 and 23.9 (d, *J*_CP_ = 4.3 Hz), 24.3 (d, *J*_CP_ = 3.4 Hz, 2C), 25.8, 26.2, 27.9, 61.5, 69.3, 69.4, 73.9 (d, *J*_CP_ = 7.1 Hz, 2C), 82.5, 84.0, 84.7, 87.3, 116.7 (dt, *J*_CF_ = 259.1 Hz, *J*_CP_ = 220.8 Hz), 122.7 (dt, *J*_CF_ = 21.3 Hz, *J*_CP_ = 13.4 Hz), 129.4, 134.4 (dt, *J*_CF_ = 9.8 Hz, *J*_CP_ = 5.3 Hz), 143.4, 150.4, 150.6, 152.2, 152.6. HRMS-ESI (*m*/*z*) calcd for C_42_H_75_F_2_N_5_O_11_PSi_2_ [M+H]^+^ 950.4707, found 950.4697.

*tert*-butyl (*tert*-butoxycarbonyl)(9-((*2R*,*3R*,*4R*,*5R*)-4-((*tert*-butyldimethylsilyl)oxy)-3-(((*E*)-4-(diisopropoxyphosphoryl)-4,4-difluorobut-2-en-1-yl)oxy)-5-(hydroxymethyl)tetrahydrofuran-2-yl)-9H-purin-6-yl)carbamate (**15**). The general procedure G (method G1) was followed with compound **14** (666 mg, 0.70 mmol) in H_2_O (1.2 mL), THF (1.2 mL), and AcOH (3.75 mL). The crude product was purified by column chromatography (Eluent Cyclohexane/EtOAc 6:4 to 4:6) to give compound **15** (315 mg, 0.38 mmol, 54%) as a colorless oil. ^1^H NMR (CDCl_3_, 600 MHz): δ 0.12 (s, 6H), 0.93 (s, 9H), 1.31 (dd, *J* = 6.2 Hz, *J*_HP_ = 2.6 Hz, 6H), 1.33 (d, *J* = 6.2 Hz, 6H), 1.46 (s, 18H), 3.73 (dd, *J* = 13.1 Hz, *J* = 1.4 Hz, 1H), 3.86–3.89 (m, 1H), 3.97 (dd, *J* = 13.1 Hz, *J* = 1.8 Hz, 1H), 4.08–4.11 (m, 1H), 4.21–4.23 (m, 1H), 4.57–4.58 (m, 1H), 4.72 (dd, *J* = 7.6 Hz, *J* = 4.5 Hz, 1H), 4.77–4.83 (m, 2H), 5.83–5.90 (m, 1H), 6.01 (d, *J* = 7.6 Hz, 1H), 6.10–6.15 (m, 1H), 8.22 (s, 1H), 8.82 (s, 1H). ^19^F NMR (CDCl_3_, 470 MHz): δ −109.7 (ddd, *J*_FF_ = 315.6 Hz, *J*_FP_ = 113.2 Hz, *J*_FH_ = 12.3 Hz), −110.6 (ddd, *J*_FF_ = 315.6 Hz, *J*_FP_ = 113.2 Hz, *J*_FH_ = 12.3 Hz). ^31^P NMR (CDCl_3_, 202 MHz): δ 4.2 (t, *J*_PF_ = 113.2 Hz). ^13^C NMR (CDCl_3_, 150 MHz): δ −4.6, −4.5, 18.2, 23.8 and 23.9 (d, *J*_CP_ = 2.3 Hz), 24.2 and 24.3 (d, *J*_CP_ = 1.4 Hz), 25.8, 27.9, 62.9, 69.1, 71.6, 73.9 and 74.0 (d, *J*_CP_ = 3.7 Hz), 80.9, 84.3, 89.5, 89.6, 116.6 (dt, *J*_CF_ = 258.6 Hz, *J*_CP_ = 220.6 Hz), 122.7 (dt, *J*_CF_ = 21.2 Hz, *J*_CP_ = 13.4 Hz), 130.4, 134.0 (dt, *J*_CF_ = 10.1 Hz, *J*_CP_ = 5.8 Hz), 144.9, 150.5, 151.4, 151.6, 152.0. HRMS-ESI (*m*/*z*) calcd for C_36_H_61_F_2_N_5_O_11_PSi [M+H]^+^ 836.3843, found 836.3840.

((*2R*,*3R*,*4R*,*5R*)-5-(6-(bis(*tert*-butoxycarbonyl)amino)-9H-purin-9-yl)-3-((*tert*-butyldimethylsilyl)oxy)-4-(((*E*)-4-(diisopropoxyphosphoryl)-4,4-difluorobut-2-en-1-yl)oxy)tetrahydrofuran-2-yl)methyl sulfamate (**16**). The general procedure A (Method A2) was followed with compound **15** (100 mg, 0.12 mmol) in DMAc (0.6 mL), sulfamoyl chloride (33 mg, 0.29 mmol) in MeCN (0.5 mL) to give compound **16** (105 mg, 0.10 mmol, 84%) with DMAc (13% w/w) as a colorless oil. This compound was used in the next step without further purification to avoid its degradation. ^1^H NMR (CDCl_3_, 600 MHz): δ 0.12 (s, 6H), 0.91 (s, 9H), 1.33 (dd, *J* = 6.2 Hz, *J*_HP_ = 2.4 Hz, 6H), 1.36 (d, *J* = 6.2 Hz, 6H), 1.45 (s, 18H), 4.20–4.27 (m, 2H), 4.30–4.33 (m, 2H), 4.47–4.49 (m, 1H), 4.53–4.55 (m, 1H), 4.58–4.60 (m, 1H), 4.80–4.87 (m, 2H), 5.88 (br, s, 2H), 5.93–5.96 (m, 1H), 6.20 (d, *J* = 4.2 Hz, 1H), 6.20–6.24 (m, 1H), 8.44 (s, 1H), 8.85 (s, 1H). ^19^F NMR (CDCl_3_, 565 MHz): δ −109.7 (ddd, *J*_FF_ = 334.1 Hz, *J*_FP_ = 114.2 Hz, *J*_FH_ = 12.8 Hz, 1F), −110.5 (ddd, *J*_FF_ = 334.1 Hz, *J*_FP_ = 114.2 Hz, *J*_FH_ = 12.8 Hz, 1F). ^31^P NMR (CDCl_3_, 243 MHz): δ 4.0 (t, *J*_PF_ = 114.2 Hz). ^13^C NMR (CDCl_3_, 150 MHz): δ −4.8, −4.6, 18.1, 23.8 and 23.9 (d, *J*_CP_ = 4.7 Hz), 24.2 and 24.3 (d, *J*_CP_ = 2.3 Hz), 25.8, 27.9, 67.9, 69.3, 70.5, 74.3 and 74.4 (d, *J*_CP_ = 7.2 Hz), 80.6, 82.7, 84.3, 87.5, 116.5 (dt, *J*_CF_ = 258.4 Hz, *J*_CP_ = 220.7 Hz), 123.3 (dt, *J*_CF_ = 21.3 Hz, *J*_CP_ = 13.7 Hz), 128.9, 134.4 (dt, *J*_CF_ = 9.8 Hz, *J*_CP_ = 5.7 Hz), 143.8, 150.4, 150.6, 152.4, 152.7. HRMS-ESI (*m*/*z*) calcd for C_36_H_62_F_2_N_6_O_13_SiPS [M+H]^+^ 915.3571, found 915.3564.

((*2R*,*3R*,*4R*,*5R*)-5-(6-(bis(*tert*-butoxycarbonyl)amino)-9H-purin-9-yl)-3-((*tert*-butyldimethylsilyl)oxy)-4-(((*E*)-4-(diisopropoxyphosphoryl)-4,4-difluorobut-2-en-1-yl)oxy)tetrahydrofuran-2-yl)methyl ((*tert*-butoxycarbonyl)-D-alanyl)sulfamate (**17**). The general procedure B was followed with compound **16** (285 mg, 0.31 mmol), Boc-D-Ala-OSu (89 mg, 0.31 mmol), and DBU (0.07 mL, 0.46 mmol) in DMF (4.4 mL). The crude was purified by column chromatography (Eluent EtOAc/CycloHex 9:1) to give compound **17** (195 mg, 0.18 mmol, 58%) as a waxy oil. ^1^H NMR (Acetone-d_6_, 500 MHz): δ 0.19 (s, 3H), 0.21 (s, 3H), 0.97 (s, 9H), 1.29 (dd, *J* = 6.3 Hz, *J*_HP_ = 1.8 Hz, 6H), 1.31 (d, *J* = 6.4 Hz, 3H), 1.33 (d, *J* = 6.2 Hz, 6H), 1.39 (s, 9H), 1.44 (s, 18H), 4.01–4.06 (m, 1H), 4.28–4.38 (m, 4H), 4.41–4.46 (m, 1H), 4.75–4.83 (m, 4H), 5.96–6.04 (m, 2H), 6.26–6.31 (m, 1H), 6.39–6.41 (m, 1H), 8.77 (s, 1H), 8.83 (s, 1H). ^19^F NMR (Acetone-d_6_, 470 MHz): δ −109.8 (ddd, *J*_FF_ = 291.6 Hz, *J*_FP_ = 113.4 Hz, *J*_FH_ = 12.9 Hz), −110.5 (ddd, *J*_FF_ = 291.6 Hz, *J*_FP_ = 113.4 Hz, *J*_FH_ = 12.9 Hz). ^31^P NMR (Acetone-d_6_, 202 MHz): δ 3.7 (t, *J*_PF_ = 111.6 Hz). ^13^C NMR (Acetone-d_6_, 150 MHz): δ −4.5, −4.4, 18.6, 19.9, 23.9 and 24.0 (d, *J*_CP_ = 3.1 Hz), 24.3 (d, *J*_CP_ = 3.3 Hz, 2C), 26.2, 27.9, 28.7, 53.2, 68.9, 69.6, 71.9, 74.3 and 74.4 (d, *J*_CP_ = 6.4 Hz), 79.0, 82.2, 84.0, 84.3, 87.4, 117.7 (dt, *J*_CF_ = 257.6 Hz, *J_CP_* = 222.3 Hz), 122.7 (dt, *J*_CF_ = 22.1 Hz, *J*_CP_ = 13.5 Hz), 129.7, 135.8 (dt, *J*_CF_ = 10.3 Hz, *J*_CP_ = 5.6 Hz), 145.1, 151.0, 151.2, 152.7, 153.9, 155.9, 181.0. HRMS-ESI (*m*/*z*) calcd for C_44_H_75_F_2_N_7_O_16_PSSi [M+H]^+^ 1086.4466, found 1086.4462.

((*2R*,*3R*,*4R*,*5R*)-5-(6-amino-9H-purin-9-yl)-4-(((*E*)-4-(diisopropoxyphosphoryl)-4,4-difluorobut-2-en-1-yl)oxy)-3-hydroxytetrahydrofuran-2-yl)methyl (D-alanyl)sulfamate salt (**18**). The general procedure C (Method C2) was followed with compound **17** (317 mg, 0.29 mmol), TFA (3.6 mL), and CH_2_Cl_2_/H_2_O (3:0.7 mL). The crude was purified by HPLC RP-18 (MeCN/H_2_O, linear gradient) to give compound **18** (112 mg, 0.12 mmol, 43%) as a white solid (2 TFA salt). ^1^H NMR (MeOD, 600 MHz): δ 1.31 (d, *J* = 6.1 Hz, 6H), 1.34 (dd, *J* = 6.1 Hz, *J*_HP_ = 1.8 Hz, 6H), 1.52 (d, *J* = 7.1 Hz, 3H), 3.87 (q, *J* = 7.1 Hz, 1H), 4.32–4.36 (m, 2H), 4.43–4.47 (m, 2H), 4.48–4.53 (m, 2H), 4.75–4.80 (m, 2H), 5.91–5.99 (m, 1H), 6.27 (d, *J* = 4.1 Hz, 1H), 6.29–6.33 (m, 1H), 8.42 (s, 1H), 8.62 (s, 1H). ^19^F NMR (MeOD, 470 MHz): δ −111.5 (m), −111.2 (m). ^31^P NMR (MeOD, 202 MHz): δ 4.2 (t, *J*_PF_ = 114.6 Hz). ^13^C NMR (MeOD, 150 MHz): δ 17.3, 24.0 (d, *J*_CP_ = 4.9 Hz, 2C), 24.3 and 24.4 (d, *J*_CP_ = 1.2 Hz), 52.0, 70.2, 70.5, 72.2, 75.9 (d, *J*_CP_ = 7.3 Hz, 2C), 83.7, 85.0, 88.8, 117.8 (dt, *J*_CF_ = 252.8 Hz, *J*_CP_ = 225.1 Hz), 120.4, 122.6 (dt, *J*_CF_ = 21.9 Hz, *J*_CP_ = 13.8 Hz), 136.9 (dt, *J*_CF_ = 9.8 Hz, *J*_CP_ = 5.6 Hz), 143.4, 146.3, 149.8, 152.4, 174.1. HPLC purity: (77.6%, t_R_ = 10.53 min). HRMS-ESI (*m*/*z*) calcd for C_23_H_37_F_2_N_7_O_10_PS [M+H]^+^ 672.2028, found 672.2036.

2′,3′-*O*-isopropylidene-5′-*N*-(3-amino-cyclobut-3-ene-1,2-dione)aminodeoxyadenosine (**21**). To a solution of **20** (0.92 g, 3 mmol, 1 equiv.), prepared as previously described [36,37], in MeOH (20 mL), dimethyl squarate (0.89 g, 6.26 mmol, 2 equiv.) was added at 20 °C. The mixture was stirred for 4 h and concentrated under reduced pressure. The residue was dissolved in MeOH (70 mL), and a solution of NH_3_ (7 N) in MeOH (4.87 mL) was added at 20 °C. The mixture was stirred for 16 h, and the reaction mixture was filtered. The solid was washed with pentane to give compound **21** (0.58 g, 1.45 mmol, 48%) as a white foam. The product was used in the next step without further purification. ^1^H NMR (DMSO-d_6_, 500 MHz): δ 1.32 (s, 3H), 1.54 (s, 3H), 3.70–3.76 (m, 1H), 3.94 (br s, 1H), 4.24–4.27 (m, 1H), 5.00–5.02 (m, 1H), 5.43 (s, 1H), 6.20 (s, 1H), 7.37 (br s, 2H), 7.44 (br s, 1H), 8.19 (s, 1H), 8.33 (s, 1H). ^13^C NMR (DMSO-d_6_, 150 MHz): δ 25.7, 27.5, 27.3, 45.6, 81.6, 83.6, 89.2, 114.2, 119.6, 140.4, 149.2, 153.4, 156.6, 170.1, 183.8.

2′,3′-*O*-isopropylidene-5′-*N*-(*N*-*tert*-butoxycarbonyl-D-alanyl]-(3amino-cyclobut-3-ene-1,2-dione))aminodeoxy adenosine (**22**). The general procedure B was followed with compound **21** (200 mg, 0.5 mmol), Boc-D-Ala-OSu (180 mg, 0.63 mmol), and DBU (0.19 mL, 1.2 mmol) in DMF (5 mL) at 60 °C for 4h. The crude mixture was purified by column chromatography (Eluent EtOAc/MeOH 9:1) to give compound **22** (140 mg, 0.244 mmol, 49%) as a white solid. ^1^H NMR (DMSO-d_6_, 500 MHz): δ 1.19–1.20 (m, 5H), 1.31 (s, 3H), 1.36 (s, 18H), 1.53 (s, 3H), 3.69–3.74 (m, 1H), 3.81–3.87 (m, 2H), 4.23–4.27 (m, 1H), 5.01–5.03 (m, 1H), 5.42 (s, 1H), 6.18 (s, 1H), 6.87 (br s, 1H), 7.36 (s, 1H), 7.85 (br s, 1H), 8.17 (s, 1H), 8.33 (s, 1H). ^13^C NMR (DMSO-d_6_, 150 MHz): δ 18.1, 25.7, 27.5, 28.6, 45.5, 49.7, 78.2, 81.7, 83.2, 83.6, 89.2, 113.8, 114.2, 119.6, 140.3, 149.3, 153.4, 155.5, 156.6, 170.2, 175.5, 183.7. HRMS-ESI (*m*/*z*) calcd for C_25_H_33_N_8_O_8_ [M+H]^+^ 573.2421 found 573.2428.

5′-*N*-(*N*-D-alanyl]-(3-amino-cyclobut-3-ene-1,2-dione))aminodeoxy adenosine salt (**23**). The general procedure C (Method C2) was followed with compound **22** (210 mg, 0.37 mmol), TFA (5 mL), and CH_2_Cl_2_/H_2_O (4/1 mL). The crude mixture was purified by HPLC RP-C18 (MeCN/H_2_O, linear gradient) to give compound **23** (100.8 mg, 0.153 mmol, 64%) as a white solid (2 TFA salt). ^1^H NMR (DMSO-d_6_, 500 MHz): δ 1.38–1.43 (m, 3H), 3.86–3.94 (m, 1H), 4.05–4.13 (m, 3H),4.18–4.23 (m, 1H), 4.61–4.65 (m, 1H), 5.93 (d, *J* = 5.5 Hz, 1H), 7.80 (br s, 1H), 8.10 (br s, 2H), 8.26 (br s, 2H), 8.28 (s, 1H), 8.43 (s, 1H), 11.98 (s, 1H). ^13^C NMR (DMSO-d_6_, 150 MHz): δ 16.6, 45.8, 48.5, 70.8, 73.1, 83.2,87.7, 119.1, 140.7, 148.9, 150.1, 154.1, 169.3, 172.1, 172.8, 181.4, 189.3. HPLC purity: (97.1%, t_R_ = 3.10 min). HRMS-ESI (*m*/*z*) calcd for C_17_H_21_N_8_O_6_ [M+H]^+^ 433.1584, found 433.1585.

*tert*-butyl (*tert*-butoxycarbonyl)(9-((*3aR*,*4R*,*6R*,*6aR*)-6-(((*tert*-butoxycarbonyl)(*N*-((*tert*-butoxycarbonyl)-D-alanyl)sulfamoyl)amino)methyl)-2,2-dimethyltetrahydrofuro[3,4-d][1,3]dioxol-4-yl)-9H-purin-6-yl)carbamate (**28**). The general procedure B was followed with compound **26** (1.2 g, 1.76 mmol), prepared as previously described [20], Boc-D-Ala-OSu (640 mg, 2.23 mmol), and DBU (0.6 mL, 4.22 mmol) in DMF (25 mL). The crude was purified by flash chromatography (Eluent CH_2_Cl_2_/MeOH 9:1) to give compound **28** (1.1 g, 1.33 mmol, 76%) as a white foam. ^1^H NMR (CDCl_3_, 500 MHz): δ 1.32 (d, *J* = 7.4 Hz, 3H), 1.39 (s, 3H), 1.43 (s, 18H), 1.44 (s, 9H), 1.62 (s, 3H), 4.04–4.15 (m, 3H), 4.20 (dd, *J* = 15.4 Hz, *J* = 5.9 Hz, 1H), 4.53–4.58 (m, 1H), 4.83–4.95 (m, 1H), 5.13–5.17 (m, 1H), 5.38–5.41 (m, 1H), 6.18 (d, *J* = 2.1 Hz, 1H), 8.21 (s, 1H), 8.87 (s, 1H). ^13^C NMR (CDCl_3_, 125 MHz): δ 16.4, 25.6, 27.4, 27.9, 28.0, 28.4, 50.3, 82.4, 83.9, 84.5, 85.3, 85.4, 90.7, 114.9, 129.6, 144.0, 150.5, 150.7, 152.4, 152.7, 171.7. HRMS-ESI (*m*/*z*) calcd for C_31_H_48_N_8_O_12_S [M+H]^+^ 757.3191, found 757.3193.

*tert*-butyl (*tert*-butoxycarbonyl)(9-((*2R*,*3R*,*4R*,*5R*)-5-(((*tert*-butoxycarbonyl)(*N*-((*tert*-butoxycarbonyl)-D-alanyl)sulfamoyl)amino)methyl)-4-((*tert*-butyldimethylsilyl)oxy)-3-fluorotetrahydrofuran-2-yl)-9H-purin-6-yl)carbamate (**29**). The general procedure B was followed with compound **27** (185 mg, 0.24 mmol), prepared as previously described [20], Boc-D-Ala-OSu (70 mg, 0.24 mmol), and DBU (55 µL, 0.36 mmol) in DMF (3.3 mL). The crude was purified by column chromatography (Eluent Pentane/EtOAc 1:1) to give compound **29** (173 mg, 0.18 mmol, 77%) as a white foam. ^1^H NMR (CD_3_CN, 600 MHz): δ 0.16 (s, 3H), 0.19 (s, 3H), 0.95 (s, 9H), 1.19 (d, *J* = 7.2 Hz, 3H), 1.25 (s, 9H), 1.40 (s, 18H), 1.41 (s, 9H), 3.97–4.03 (m, 2H), 4.14–4.17 (m, 1H), 4.34–4.35 (m, 1H), 4.80–4.83 (m, 1H), 5.53–5.63 (m, 1H), 5.77 (br s, 1H), 6.30 (dd, *J*_FH_ = 17.5 Hz, *J* = 2.4 Hz, 1H), 8.44 (s, 1H), 8.81 (s, 1H). ^19^F NMR (CD_3_CN, 470 MHz): δ −205.6 (m). ^13^C NMR (CD_3_CN, 150 MHz): δ −4.7, −4.5, 18.2, 18.7, 26.1, 27.9, 28.1, 28.7, 50.4, 53.3, 73.0 (d, *J*_CF_ = 15.9 Hz), 80.9, 83.1, 85.0, 85.9, 88.3 (d, *J*_CF_ = 32.9 Hz), 93.3 (d, *J*_CF_ = 190.8 Hz), 130.1, 146.0, 151.0, 151.5, 152.8, 153.6, 155.6, 157.2, 179.5. HRMS-ESI (*m*/*z*) calcd for C_39_H_66_FN_8_O_13_SiS [M+H]^+^ 933.4223, found 933.4211.

(*R*)-2-amino-*N*-(*N*-(((*2R*,*3S*,*4R*,*5R*)-5-(6-amino-9H-purin-9-yl)-3,4-dihydroxytetrahydrofuran-2-yl)methyl)sulfamoyl)propenamide salt (**30**). The general procedure C (Method C2) was followed with compound **28** (528 mg, 0.62 mmol), TFA (7.5 mL), and CH_2_Cl_2_/H_2_O (6/1.5 mL). The crude was purified by HPLC RP-C18 (MeCN/H_2_O, linear gradient) to give compound **30** (219 mg, 0.34 mmol, 55%) as a white solid (2 TFA salt). ^1^H NMR (D_2_O, 500 MHz): δ 1.52 (d, *J* = 7.2 Hz, 3H), 3.37–3.38 (m, 2H), 3.97 (q, *J* = 7.2 Hz, 1H), 4.31–4.34 (m, 1H), 4.41–4.44 (m, 1H), 4.80–4.83 (m, 1H), 6.04 (d, *J* = 5.8 Hz, 1H), 8.37 (s, 1H), 8.39 (s, 1H). ^13^C NMR (D_2_O, 125 MHz): δ 16.4, 44.3, 50.4, 71.0, 73.5, 83.7, 89.1, 119.3, 142.8, 147.1, 148.2, 151.7, 173.0. HRMS-ESI (*m*/*z*) calcd for C_13_H_20_N_8_O_6_S [M+H]^+^ 417.1305, found 417.1304.

(*R*)-2-amino-*N*-(*N*-(((*2R*,*3R*,*4R*,*5R*)-5-(6-amino-9H-purin-9-yl)-4-fluoro-3-hydroxytetrahydrofuran-2-yl)methyl)sulfamoyl)propenamide salt (**31**). The general procedure C (Method C2) was followed with compound **29** (173 mg, 0.18 mmol), TFA (2.3 mL), and CH_2_Cl_2_/H_2_O (2/0.5 mL) to give compound **31** (105 mg, 0.16 mmol, 88%) as a white solid (2 TFA salt). ^1^H NMR (D_2_O, 500 MHz): δ 1.47 (d, *J* = 7.2 Hz, 3H), 3.33 (dd, *J* = 14.3 Hz, *J* = 4.5 Hz, 1H), 3.42 (dd, *J* = 14.3 Hz, *J* = 2.8 Hz, 1H), 3.85 (q, *J* = 7.2 Hz, 1H), 4.28–4.32 (m, 1H), 4.70 (ddd, *J*_HF_ = 18.3 Hz, *J* = 7.2 Hz, *J* = 4.7 Hz, 1H), 5.50 (ddd, *J*_HF_ = 52.1 Hz, *J* = 4.7 Hz, *J* = 2.4 Hz, 1H), 6.34 (dd, *J*_HF_ = 17.3 Hz, *J* = 2.4 Hz, 1H), 8.28 (s, 1H), 8.32 (s, 1H). ^19^F NMR (D_2_O, 470 MHz): δ −203.9 (dt, *J*_FH_ = 52.1 Hz, *J*_FH_ = 17.3 Hz, 1F). ^13^C NMR (D_2_O, 125 MHz): 16.6, 43.4, 51.0, 69.5 (d, *J*_CF_ = 17.1 Hz), 81.6, 87.1 (d, *J*_CF_ = 33.9 Hz), 93.0 (d, *J*_CF_ = 187.9 Hz), 119.1, 141.3, 148.3, 150.6, 154.1, 175.3. HPLC purity: (98.3%, t_R_ = 2.08 min). HRMS-ESI (*m*/*z*) calcd for C_13_H_19_FN_8_O_5_S [M+H]^+^ 419.1261, found 419.1264.

*tert*-butyl (*E*)-(*tert*-butoxycarbonyl)(9-(2,4-difluoro-3-(hydroxymethyl)but-2-en-1-yl)-9H-purin-6-yl)carbamate (**34**). A solution of compound **33** (247 mg, 0.48 mmol, 1 equiv.), prepared as previously described [40], in *t*-BuOH (5 mL) was added CsF (111 mg, 0.96 mmol, 2.0 equiv.). The reaction mixture was stirred at 70 °C for 4 h, and the solvent was removed under reduced pressure. The residue was taken up with EtOAc/H_2_O, and the aqueous layers were extracted three times with EtOAc. The combined organic layers were washed with brine, dried over MgSO_4_, filtered, and concentrated under reduced pressure. The crude product was purified by column chromatography (Eluent Pentane/EtOAc 1:1 to 6:4) to give compound **34** (93 mg, 0.21 mmol, 43%) as a colorless oil. ^1^H NMR (CDCl_3_, 500 MHz): δ 1.44 (s, 18H), 2.33 (br s, 1H), 4.34 (s, 2H), 5.14 (dd, *J*_HF_ = 21.7 Hz, *J*_HF_ = 1.8 Hz, 2H), 5.35 (dd, *J*_HF_ = 47.8 Hz, *J*_HF_ = 2.2 Hz, 2H), 8.14 (s, 1H), 8.85 (s, 1H). ^19^F NMR (CDCl_3_, 470 MHz): δ −107.4 (t, *J*_FH_ = 21.7 Hz), −212.1 (t, *J*_FH_ = 47.8 Hz). ^13^C NMR (CDCl_3_, 125 MHz): δ 27.9, 41.0 (d, *J*_CF_ = 29.6 Hz), 56.3 (dd, *J*_CF_ = 8.7 Hz, *J*_CF_ = 1.2 Hz), 78.6 (dd, *J*_CF_ = 166.7 Hz, *J*_CF_ = 10.1 Hz), 84.1, 118.8 (dd, *J*_CF_ = 15.5 Hz, *J*_CF_ = 13.4 Hz), 128.5, 144.5, 150.5, 150.6, 152.5, 153.2, 153.7 (dd, *J*_CF_ = 269.6 Hz, *J*_CF_ = 10.8 Hz). HRMS-ESI (*m*/*z*) calcd for C_20_H_28_F_2_N_5_O_5_ [M+H]^+^ 456.2059, found 456.2054.

*tert*-butyl (*E*)-(*tert*-butoxycarbonyl)(9-(4-((*tert*-butoxycarbonyl)(sulfamoyl)amino)-2-fluoro-3-(fluoromethyl)but-2-en-1-yl)-9H-purin-6-yl)carbamate (**36**). The general procedure D was followed with compound **34** (120 mg, 0.26 mmol), *tert*-butyl sulfamoylcarbamate (155 mg, 0.78 mmol), triphenylphosphine (102 mg, 0.39 mmol), and diisopropyl azodicarboxylate (0.05 mL, 0.27 mmol) in dry THF (5.2 mL). The crude product was purified by column chromatography (EtOAc/Pentane 1:2) to give compound **36** (123 mg, 0.19 mmol, 74%) as a white foam. ^1^H NMR (CDCl_3_, 600 MHz): δ 1.44 (s, 9H), 1.46 (s, 18H), 4.57 (br s, 2H), 5.16 (d, *J*_HF_ = 21.1 Hz, 2H), 5.33 (d, *J*_HF_ = 47.8 Hz, 2H), 5.40 (br s, 2H), 8.15 (s, 1H), 8.85 (s, 1H). ^19^F NMR (CDCl_3_, 565 MHz): δ −103.1 (t, *J*_FH_ = 21.1 Hz), −208.1 (t, *J*_FH_ = 47.8 Hz). ^13^C NMR (CDCl_3_, 150 MHz): δ 27.9, 28.1, 41.1 (d, *J*_CF_ = 30.1 Hz), 43.1 (d, *J*_CF_ = 8.4 Hz), 79.6 (dd, *J*_CF_ = 165.9 Hz, *J*_CF_ = 10.4 Hz), 84.1, 85.3, 114.9 (dd, *J*_CF_ = 13.9 Hz, *J*_CF_ = 12.1 Hz), 128.6, 144.4, 150.6, 150.7, 151.9, 152.6, 153.1, 155.6 (dd, *J*_CF_ = 263.6 Hz, *J*_CF_ = 11.7 Hz). HRMS-ESI (*m*/*z*) calcd for C_25_H_38_F_2_N_7_O_8_S [M+H]^+^ 634,2471 found 634.2474.

*tert*-butyl (*E*)-(*tert*-butoxycarbonyl)(9-(4-((*N*-((*tert*-butoxycarbonyl)-D-alanyl)sulfamoyl)amino)-2-fluoro-3-(fluoromethyl)but-2-en-1-yl)-9H-purin-6-yl)carbamate (**37**). The general procedure B was followed with compound **36** (130 mg, 0.20 mmol), Boc-D-Ala-OSu (59 mg, 0.20 mmol), and DBU (46 µL, 0.31 mmol) in DMF (2.6 mL). The crude was purified by column chromatography (Eluent EtOAc/Pentane 1:1 to 1:0) to give compound **37** (100 mg, 0.12 mmol, 61%) as a white foam. ^1^H NMR (CDCl_3_, 600 MHz): δ 1.31 (d, *J*_HF_ = 6.9 Hz, 3H), 1.40 (s, 9H), 1.44 (s, 18H), 1.45 (s, 18H), 4.69 (br s, 2H), 5.17 (d, *J*_HF_ = 21.3 Hz, 2H), 5.26 (d, *J*_HF_ = 47.8 Hz, 2H), 8.18 (s, 1H), 8.86 (s, 1H). ^19^F NMR (CDCl_3_, 565 MHz): δ −103.9–103.6 (m), −209.6 (t, *J_FH_* = 47.8 Hz). ^13^C NMR (CDCl_3_, 150 MHz): δ 18.1, 27.8, 27.9, 28.4, 41.1 (d, *J*_CF_ = 29.4 Hz), 44.1, 52.1, 78.4 (dd, *J*_CF_ = 166.4 Hz, *J*_CF_ = 9.6 Hz), 80.1, 84.1, 84.4, 116.2 (m), 128.4, 144.8, 150.2, 150.6, 152.3, 152.7, 153.3, 154.6 (dd, *J*_CF_ = 262.3 Hz, *J*_CF_ = 10.9 Hz), 156.0, 177.7. HRMS-ESI (*m*/*z*) calcd for C_33_H_51_F_2_N_8_O_11_S [M+H]^+^ 805.3366, found 805.3391.

(*R*,*E*)-2-amino-*N*-(*N*-(4-(6-amino-9H-purin-9-yl)-3-fluoro-2-(fluoromethyl)but-2-en-1-yl)sulfamoyl)propenamide salt (**38**). The general procedure C (Method C2) was followed with compound **37** (100 mg, 0.12 mmol), TFA (1.4 mL) and CH_2_Cl_2_/H_2_O (1.2/0.3 mL) to give compound **38** (76 mg, 0.11 mmol, 90%) as a white solid (2 TFA salt). ^1^H NMR (MeOD, 600 MHz): δ 1.52 (d, *J* = 7.1 Hz, 3H), 3.88 (br s, 2H), 3.92 (q, *J* = 7.1 Hz), 5.28 (dd, *J*_HF_ = 20.6 Hz, *J*_HF_ = 1.6 Hz, 2H), 5.33 (dd, *J*_HF_ = 47.6 Hz, *J*_HF_ = 1.8 Hz, 2H), 8.24 (s, 1H), 8.32 (s, 1H). ^19^F NMR (CDCl_3_, 565 MHz): δ −105.5 (m), −211.3 (m).^13^C NMR (MeOD, 150 MHz): δ 17.1, 39.0 (d, *J*_CF_ = 9.1 Hz), 41.8 (d, *J*_CF_ = 28.8 Hz), 50.5, 79.3 (dd, *J*_CF_ = 163.1 Hz, *J*_CF_ = 9.2 Hz), 116.6 (dd, *J*_CF_ = 15.1 Hz, *J_CF_* = 12.6 Hz), 118.1 (q, *J*_CF_ = 292.1 Hz), 119.7, 143.8, 149.8, 150.6, 154.6, 156.5 (dd, *J*_CF_ = 261.7 Hz, *J*_CF_ = 10.8 Hz), 163.4 (q, *J*_CF_ = 32.4 Hz), 170.1. HPLC purity: (95.8%, t_R_ = 3.23 min). HRMS-ESI (*m*/*z*) calcd for C_13_H_19_N_8_O_3_F_2_S [M+H]^+^ 405.1269, found 405.1271.

*tert*-butyl ((*R*)-1-(1-(((*2R*,*3R*,*4R*,*5R*)-5-(6-amino-9H-purin-9-yl)-4-fluoro-3-hydroxytetrahydrofuran-2-yl)methyl)-1H-1,2,3-triazol-4-yl)-1-oxopropan-2-yl)carbamate (**45**). The general procedure E was followed with compound **43** (410 mg, 1.39 mmol), prepared as previously described [43], alkyne **S6** (295 mg, 1.53 mmol), sodium ascorbate (28 mg, 0.14 mmol), and CuSO_4_.5H_2_O (17 mg, 0.07 mmol) in *t*-BuOH/H_2_O (9 mL). The crude was purified by column chromatography (Eluent EtOAc/MeOH 94:6) to give compound **45** (470 g, 0.94 mmol, 68%) as a white solid. ^1^H NMR (DMSO-d_6_, 500 MHz): δ 1.26 (d, *J* = 7.4 Hz, 3H), 1.32 (s, 9H), 4.34 (dt, *J* = 7.4 Hz, *J* = 3.2 Hz, 1H), 4.69–4.77 (m, 1H), 4.82 (dd, *J* = 14.8 Hz, *J* = 7.4 Hz, 1H), 4.88 (dd, *J* = 14.8 Hz, *J* = 2.8 Hz, 1H), 4.89–4.95 (m, 1H), 5.50 (ddd, *J*_HF_ = 52.6 Hz, *J* = 4.5 Hz, *J* = 1.3 Hz, 1H), 6.08 (d, *J* = 6.4 Hz, 1H), 6.24 (dd, *J*_HF_ = 20.6 Hz, *J* = 1.3 Hz, 1H), 7.24 (d, *J* = 6.8 Hz, 1H), 7.33 (br s, 2H), 8.10 (s, 1H), 8.23 (s, 1H), 8.60 (s, 1H). ^19^F NMR (DMSO-d_6_, 470 MHz): δ −201.2 (dt, *J*_FH_ = 52.6 Hz, *J*_FH_ = 20.6 Hz). ^13^C NMR (DMSO-d_6_, 125 MHz): δ 16.6, 28.1, 51.0, 52.3, 69.7 (d, *J*_CF_ = 16.3 Hz), 78.0, 80.0, 86.5 (d, *J*_CF_ = 34.8 Hz), 93.0 (d, *J*_CF_ = 186.5 Hz), 119.2, 128.7, 140.0, 144.6, 148.6, 152.7, 155.2, 156.1, 193.4. HRMS-ESI (*m*/*z*) calcd for C_20_H_27_FN_9_O_5_ [M+H]^+^ 492.2119, found 492.2123.

*N*-((*R*)-1-(1-(((*3aR*,*4R*,*6R*,*6aR*)-6-(6-amino-9H-purin-9-yl)-2,2-dimethyltetrahydrofuro[3,4-d][1,3]dioxol-4-yl)methyl)-1H-1,2,3-triazol-4-yl)-1-oxopropan-2-yl)heptanamide (**46**). The general procedure E was followed with compound **44** (400 mg, 1.20 mmol), prepared as previously described [44], alkyne **S7** (277 mg, 1.32 mmol), sodium ascorbate (24 mg, 0.12 mmol), and CuSO_4_.5H_2_O (15 mg, 0.06 mmol) in *t*-BuOH/H_2_O (8 mL). The crude was purified by column chromatography (Eluent EtOAc/MeOH 1:0 to 98:2) to give compound **46** (511 mg, 0.95 mmol, 79%) as a brown foam. ^1^H NMR (CDCl_3_, 500 MHz): δ 0.80 (t, *J* = 7.0 Hz, 3H), 1.19–1.25 (m, 6H), 1.35 (s, 3H), 1.44 (d, *J* = 7.6 Hz, 3H), 1.56 (s, 3H), 2.18 (t, *J* = 7.0 Hz, 2H), 4.56–4.61 (m, 1H), 4.84–4.86 (m, 2H), 5.18–5.20 (m, 1H), 5.38–5.39 (m, 1H), 5.44–5.52 (m, 1H), 6.07 (s, 1H), 6.61 (s, 1H), 6.67–6.72 (m, 1H), 7.86 (s, 1H), 8.03 (s, 1H), 8.25 (s, 1H). ^13^C NMR (CDCl_3_, 125 MHz): δ 14.1, 18.8, 22.5, 25.4, 25.6, 27.2, 28.9, 31.6, 36.6, 52.0, 81.8, 84.1, 84.2, 85.3, 90.7, 115.1, 120.4, 128.3, 140.4, 145.3, 148.8, 152.9, 155.9, 172.9, 193.4. HRMS-ESI (*m*/*z*) calcd for C_25_H_36_N_9_O_5_ [M+H]^+^ 542.2839, found 542.2841.

*N*-((*R*)-1-(1-(((*3aR*,*4R*,*6R*,*6aR*)-6-(6-amino-9H-purin-9-yl)-2,2-dimethyltetrahydrofuro[3,4-d][1,3]dioxol-4-yl)methyl)-1H-1,2,3-triazol-4-yl)-1-oxopropan-2-yl)-[1,1′-biphenyl]-4-carboxamide (**47**). The general procedure E was followed with compound **44** (400 mg, 1.20 mmol), prepared as previously described [44], alkyne **S8** (367 mg, 1.32 mmol), sodium ascorbate (24 mg, 0.12 mmol), and CuSO_4_.5H_2_O (15 mg, 0.06 mmol) in *t*-BuOH/H_2_O (8 mL). The crude was purified by column chromatography (Eluent EtOAc/MeOH 1:0 to 99:1) to give compound **47** (561 mg, 0.92 mmol, 77%) as a brown foam. ^1^H NMR (CDCl_3_, 500 MHz): δ 1.33 (s, 3H), 1.55 (s, 3H), 1.59–1.62 (m, 3H), 4.56–4.61 (m, 1H), 4.80–4.88 (m, 1H), 5.17–5.19 (m, 1H), 5.36–5.37 (m, 1H), 5.66–5.73 (m, 1H), 6.06 (br s, 1H), 6.62 (br s, 2H), 7.31–7.34 (m, 1H), 7.38–7.41 (m, 2H), 7.50–7.58 (m, 5H), 7.85–7.88 (m, 3H), 8.04–8.05 (m, 1H), 8.27 (br s, 1H). ^13^C NMR (CDCl_3_, 125 MHz): δ 18.9, 25.4, 27.1, 29.7, 51.9, 52.7, 81.7, 84.1, 85.3, 90.6, 115.1, 120.4, 127.1 (2C), 127.2 (2C), 127.7 (2C), 128.4, 128.9 (2C), 132.8, 140.0, 140.2, 144.2, 145.2, 148.8, 153.3, 156.1, 166.6, 193.2. HRMS-ESI (*m*/*z*) calcd for C_31_H_32_N_9_O_5_ [M+H]^+^ 610.2526, found 610.2531.

*N*-((*R*)-1-(1-(((*3aR*,*4R*,*6R*,*6aR*)-6-(6-amino-9H-purin-9-yl)-2,2-dimethyltetrahydrofuro[3,4-d][1,3]dioxol-4-yl)methyl)-1H-1,2,3-triazol-4-yl)-1-oxopropan-2-yl)-4-(hexyloxy)benzamide (**48**). The general procedure E was followed with compound **44** (400 mg, 1.20 mmol), prepared as previously described [44], alkyne **S9** (398 mg, 1.32 mmol), sodium ascorbate (24 mg, 0.12 mmol), and CuSO_4_.5H_2_O (15 mg, 0.06 mmol) in *t*BuOH/H_2_O (8 mL). The crude was purified by column chromatography (Eluent EtOAc/MeOH 99:1) to give compound **48** (617 mg, 0.97 mmol, 81%) as a brown foam. ^1^H NMR (CDCl_3_, 500 MHz): δ 0.87–0.90 (m, 3H), 1.30–1.36 (m, 4H), 1.40 (s, 3H), 1.41–1.45 (m, 2H), 1.58–1.61 (m, 6H), 1.72–1.79 (m, 2H), 3.92–3.97 (m, 2H), 4.59–4.61 (m, 1H), 4.84–4.91 (m, 2H), 5.13–5.20 (m, 1H), 5.33–5.39 (m, 1H), 5.64–5.70 (m, 1H), 6.06 (br s, 1H), 6.82–6.88 (m, 2H), 7.75–7.77 (m, 2H), 7.88 (s, 1H), 8.04 (s, 1H), 8.28 (s, 1H). ^13^C NMR (CDCl_3_, 125 MHz): δ 14.1, 19.1, 22.7, 25.4, 25.8, 27.2, 29.2, 31.6, 52.1, 52.7, 68.3, 81.8, 84.2, 85.4, 90.7, 114.2 (2C), 115.2, 120.5, 126.2, 128.4, 129.0 (2C), 129.7, 140.6, 145.4, 149.2, 152.6, 162.0, 166.4, 193.4. HRMS-ESI (*m*/*z*) calcd for C_31_H_40_N_9_O_6_ [M+H]^+^ 634.3102, found 634.3104.

4-nitrobenzyl ((*R*)-1-(1-(((*3aR*,*4R*,*6R*,*6aR*)-6-(6-amino-9H-purin-9-yl)-2,2-dimethyltetrahydrofuro[3,4-d][1,3]dioxol-4-yl)methyl)-1H-1,2,3-triazol-4-yl)-1-oxopropan-2-yl)carbamate (**49**). The general procedure E was followed with compound **44** (400 mg, 1.20 mmol), prepared as previously described [44], compound **S10** (365 mg, 1.32 mmol), sodium ascorbate (24 mg, 0.12 mmol), and CuSO_4_.5H_2_O (15 mg, 0.06 mmol) in *t*-BuOH/H_2_O (8 mL). The crude was purified by column chromatography (Eluent EtOAc/MeOH 1:0 to 99:1) to give compound **49** (470 mg, 0.77 mmol, 64%) as a brown foam. ^1^H NMR (CDCl_3_, 500 MHz): δ 1.37 (s, 3H), 1.53 (t, *J* = 7.6 Hz, 3H), 1.58 (s, 3H), 4.58–4.61 (m, 1H), 4.87–4.88 (m, 2H), 5.13–5.22 (m, 3H), 5.32–5.35 (m, 1H), 5.40–5.42 (m, 1H), 6.04–6.07 (m, 1H), 6.35 (br s, 2H), 7.46–7.47 (m, 2H), 7.88 (s, 1H), 7.97 (s, 1H), 8.11–8.16 (m, 1H), 8.29 (s, 1H). ^13^C NMR (CDCl_3_, 125 MHz): δ 19.1, 25.4, 27.2, 29.8, 52.1, 53.7, 65.3, 81.9, 84.2, 85.4, 90.8, 115.2, 123.8 (2C), 128.1 (2C), 128.2, 140.6, 144.1, 145.2, 147.6, 152.9, 155.3, 155.8, 193.1. HRMS-ESI (*m*/*z*) calcd for C_26_H_29_N_10_O_8_ [M+H]^+^ 609.2170, found 609.2173.

(*R*)-2-amino-1-(1-(((*2R*,*3R*,*4R*,*5R*)-5-(6-amino-9H-purin-9-yl)-4-fluoro-3-hydroxytetrahydrofuran-2-yl)methyl)-1H-1,2,3-triazol-4-yl)propan-1-one salt (**50**). The general procedure C (Method C2) with compound **45** (380 mg, 0.77 mmol), TFA (9.5 mL), and CH_2_Cl_2_/H_2_O (8.7/1.8 mL) to give compound **50** (470 mg, 0.68 mmol, 88%) as a white solid (2 TFA). ^1^H NMR (D_2_O, 500 MHz): δ 1.65 (d, *J* = 7.2 Hz, 3H), 4.58–4.61 (m, 1H), 4.73 (ddd, *J*_HF_ = 22.7 Hz, *J* = 9.2 Hz, *J* = 4.4 Hz, 1H), 4.93 (q, *J* = 7.2 Hz, 1H), 5.00 (d, *J* = 3.9 Hz, 2H), 5.58 (dd, *J*_HF_ = 51.6 Hz, *J* = 4.8 Hz, 1H), 6.42 (d, *J*_HF_ = 20.3 Hz, 1H), 8.23 (s, 1H), 8.30 (s, 1H), 8.55 (s, 1H). ^19^F NMR (D_2_O, 470 MHz): δ −201.5 (ddd, *J*_FH_ = 51.6 Hz, *J*_FH_ = 22.7 Hz, *J*_FH_ = 20.3 Hz, 1F). ^13^C NMR (D_2_O, 150 MHz): δ 16.2, 50.1, 52.6, 69.3 (d, *J*_CF_ = 16.9 Hz), 79.6, 87.3 (d, *J*_CF_ = 36.1 Hz), 92.8 (d, *J*_CF_ = 186.1 Hz), 115.3, 118.7, 130.5, 142.8, 145.6, 147.7, 150.7, 189.7. HPLC purity: (98.5%, t_R_ = 10.24 min). HRMS-ESI (*m*/*z*) calcd for C_15_H_19_FN_9_O_3_ [M+H]^+^ 392.1595, found 392.1594.

*N*-((*R*)-1-(1-(((*2R*,*3S*,*4R*,*5R*)-5-(6-amino-9H-purin-9-yl)-3,4-dihydroxytetrahydrofuran-2-yl)methyl)-1H-1,2,3-triazol-4-yl)-1-oxopropan-2-yl)heptanamide salt (**51**). The general procedure C (Method C1) was followed with compound **46** (250 mg, 0.46 mmol), HCl aq (1 mL, 3 M), and MeOH (2.5 mL). The crude was precipitated in MeOH/Et_2_O to give compound **51** (219 mg, 0.41 mmol, 89%) as a white solid (HCl salt). ^1^H NMR (DMSO-d_6_, 500 MHz): δ 0.83 (t, *J* = 7.1 Hz, 3H), 1.18–1.24 (m, 6H), 1.27–1.29 (m, 3H), 1.41–1.44 (m, 2H), 2.06–2.10 (m, 2H), 4.27–4.29 (m, 1H), 4.37–4.40 (m, 1H), 4.62–4.64 (m, 1H), 4.85–4.91 (m, 2H), 5.11–5.16 (m, 1H), 5.98 (d, *J* = 5.2 Hz, 1H), 8.27 (br s, 1H), 8.54 (s, 1H), 8.74 (s, 1H), 8.77 (s, 1H), 9.07 (br s, 1H), 9.78 (br s, 1H). ^13^C NMR (DMSO-d_6_, 125 MHz): δ 14.0, 16.7, 22.0, 25.2, 28.3, 31.1, 34.8, 50.9, 51.7, 70.8, 73.4, 82.5, 88.3, 118.9, 128.9, 142.8, 144.7, 144.9, 148.2, 150.3, 172.1, 193.0. HPLC purity: (93.6%, t_R_ = 10.83 min). HRMS-ESI (*m*/*z*) calcd for C_22_H_32_N_9_O_5_ [M+H]^+^ 502.2526, found 502.2527.

*N*-((*R*)-1-(1-(((*2R*,*3S*,*4R*,*5R*)-5-(6-amino-9H-purin-9-yl)-3,4-dihydroxytetrahydrofuran-2-yl)methyl)-1H-1,2,3-triazol-4-yl)-1-oxopropan-2-yl)-[1,1′-biphenyl]-4-carboxamide salt (**52**). The general procedure C (Method C1) was followed with compound **47** (250 mg, 0.41 mmol), HCl aq (1 mL, 3 M), and MeOH (2.5 mL). The crude was precipitated in MeOH/Et_2_O to give compound **52** (205 mg, 0.34 mmol, 83%) as a white solid (HCl salt). ^1^H NMR (DMSO-d_6_, 500 MHz): δ 1.46–1.48 (m, 3H), 4.29–4.31 (m, 1H), 4.39–4.43 (m, 1H), 4.63 (dt, *J* = 7.8 Hz, *J* = 5.1 Hz, 1H), 5.87–5.94 (m, 2H), 5.36–5.42 (m, 1H), 5.99–6.01 (m, 1H), 7.39–7.42 (m, 1H), 7.47–7.51 (m, 2H), 7.72–7.78 (m, 4H), 7.97–8.00 (m, 2H), 8.53 (d, *J* = 1.6 Hz, 1H), 8.75 (d, *J* = 4.8 Hz, 1H), 8.79 (d, *J* = 9.8 Hz, 1H), 8.90 (d, *J* = 6.2 Hz, 1H), 8.97 (br s, 1H), 9.67 (br s, 1H). ^13^C NMR (DMSO-d_6_, 125 MHz): δ 16.4, 51.7, 52.0, 70.8, 73.4, 82.5, 88.3, 118.9, 126.4, (2C), 126.9 (2C), 128.1, 128.3 (2C), 128.8, 129.1 (2C), 132.5, 139.1, 142.7, 142.9, 144.8, 145.3, 148.3, 150.5, 165.9, 192.7. HPLC purity: (98.8%, t_R_ = 12.32 min). HRMS-ESI (*m*/*z*) calcd for C_28_H_28_N_9_O_5_ [M+H]^+^ 570.2213, found 570.2214.

*N*-((*R*)-1-(1-(((*2R*,*3S*,*4R*,*5R*)-5-(6-amino-9H-purin-9-yl)-3,4-dihydroxytetrahydrofuran-2-yl)methyl)-1H-1,2,3-triazol-4-yl)-1-oxopropan-2-yl)-4-(hexyloxy)benzamide salt (**53**). The general procedure C (Method C1) was followed with compound **48** (491 mg, 0.77 mmol), HCl aq (1.9 mL, 3 M), and MeOH (4.7 mL). The crude was precipitated in MeOH/Et_2_O to give compound **53** (370 mg, 0.58 mmol, 76%) as a white solid (HCl salt). ^1^H NMR (DMSO-d_6_, 500 MHz): δ 0.87 (t, *J* = 7.0 Hz, 3H), 1.28–1.31 (m, 4H), 1.39–1.44 (m, 5H), 1.68–1.73 (m, 2H), 4.01 (t, *J* = 6.6 Hz, 2H), 4.28–4.30 (m, 1H), 4.38–4.42 (m, 1H), 4.61–4.64 (m, 1H), 4.88–4.91 (m, 1H), 5.31–5.38 (m, 1H), 5.98–6.00 (m, 1H), 6.95–6.98 (m, 2H), 7.84–7.86 (m, 2H), 8.53 (s, 1H), 8.65–8.66 (m, 1H), 8.74–8.75 (m, 1H), 8.76–8.78 (m, 1H), 8.96 (br s, 1H), 9.66 (br s, 1H). ^13^C NMR (DMSO-d_6_, 125 MHz): δ 13.9, 16.4, 22.1, 25.2, 28.6, 31.0, 51.6, 51.8, 67.7, 70.8, 73.3, 82.4, 88.3, 113.8 (2C), 118.9, 125.7, 128.8, 129.4 (2C), 142.7, 144.8, 145.3, 148.3, 150.1, 161.2, 165.7, 193.1. HPLC purity: (96.5%, t_R_ = 14.41 min). HRMS-ESI (*m*/*z*) calcd for C_28_H_36_N_9_O_6_ [M+H]^+^ 594.2789, found 594.2797.

4-nitrobenzyl ((*R*)-1-(1-(((*2R*,*3S*,*4R*,*5R*)-5-(6-amino-9H-purin-9-yl)-3,4-dihydroxytetrahydrofuran-2-yl)methyl)-1H-1,2,3-triazol-4-yl)-1-oxopropan-2-yl)carbamate salt (**54**). The general procedure C (Method C1) was followed with compound **49** (250 mg, 0.41 mmol), HCl aq (1 mL, 3 M), and MeOH (2.5 mL). The crude was precipitated in MeOH/Et_2_O to give compound **54** (195 mg, 0.32 mmol, 79%) as a white solid (HCl salt). ^1^H NMR (DMSO-d_6_, 500 MHz): δ 1.34 (t, *J* = 7.4 Hz, 3H), 4.27–4.29 (m, 1H), 4.38–4.41 (m, 1H), 4.62–4.64 (m, 1H), 4.89–4.91 (m, 2H), 5.02–5.07 (m, 1H), 5.15 (s, 2H), 5.98 (d, *J* = 4.9 Hz, 1H), 7.59 (d, *J* = 8.1 Hz, 2H), 7.94 (d, *J* = 7.4 Hz, 1H), 8.22 (d, *J* = 8.1 Hz, 2H), 8.52 (s, 1H), 8.75 (s, 1H), 8.77 (s, 1H), 9.01 (br s, 1H), 9.69 (br s, 1H). ^13^C NMR (DMSO-d_6_, 125 MHz): δ 16.8, 51.6, 52.8, 64.2, 70.8, 73.4, 82.4, 88.3, 118.8, 123.5 (2C), 128.1 (2C), 128.9, 142.7, 144.5, 145.0, 145.1, 146.9, 148.2, 150.4, 155.5, 193.0. HPLC purity: (98.3%, t_R_ = 10.74 min). HRMS-ESI (*m*/*z*) calcd for C_23_H_25_N_10_O_8_ [M+H]^+^ 569.1857, found 569.1860.

### 3.4. Determination of the Binding Affinity (Molecular Docking)

The molecular docking was performed by AutoDock Vina 1.2.7 software, which uses a non-deterministic Docking Algorithm with 8 maximum exhaustiveness to determine the binding affinity for each ligand. Molecular docking was simulated with the crystal structure of *Bacillus cereus* D-alanyl carrier protein ligase [51] from the Protein Data Bank (PDB ID: 3FFC) using Research Collaboratory for Structural Bioinformatics. Crystallization cofactors such as ATP, Magnesium, as well as traces of residual water from the 3FCC structure, were removed using PyMol 3.1 software. Kollman and Gasteiger charges, as well as the polar hydrogens, were added, respectively, to the receptor and the ligands using AutoDockTools 1.5.7 software to prepare molecules in pdbqt format. The active site was defined by default through the ligand co-crystallized in the 3FCC protein, and the grid box was established with a dimension (Å) of X: 19.8903, Y: 20.2516, and Z: 24.5359; and for center, X: 18.9110, Y: 9.0989, and Z: 13.3228. Docking scores listed are the average of nine different minimized energy states.

### 3.5. Determination of the Half-Maximal Inhibitory Concentration (IC_50_)

The DltA-catalyzed ATP-hydrolysis reaction was monitored in vitro via PPi release (DltA +D-alanine +ATP → DltA-DAla-AMP +PPi) using a coupled-enzyme assay. The purified DltA was pretreated with DltC purified from *E. coli* to clear D-Ala-AMP already present in the enzyme preparation after expression in *E. coli*. Holo-DltC is the natural protein partner of DltA, and DltC purified from *E. coli* BL21 (DE3) contained 51% of this active form of DltC as determined by HPLC analysis. DltA was pretreated with DltC (ratio 1:1.2), incubated for 30 min at 37 °C and 30 min at 4 °C. Three successive washes were realized on Amicon Ultra-2 Centrifugal Filter Unit—Ultracel 30 membrane 30 kDa MWCO (Merck, Darmstadt, Germany) with elution buffer EB at 4 °C. The components of the coupled enzyme assay included 1.5 μM pre-treated DltA, 20 μM of ATP, and 14 μM of D-alanine. The half-maximal inhibitory concentration (IC_50_) of potential DltA inhibitors was determined by the addition of different concentrations of the synthesized molecules. PPi release was determined using the PiPer—Pyrophosphate assay kit (Invitrogen, Waltham, MA, USA) according to the recommendation of the supplier. Reactions were carried out in 96-well Chimney Style, non-binding Microplates with μClear Film Bottom (Greiner Bio-one, Frickenhausen, Germany). Plates were incubated at 37 °C for 5 h and read in a FlexStation 3 Multi-Mode plate reader (Molecular Devices, Silicon Valley, CA, USA), with λ excitation of 530 nm and λ emission of 590 nm. Experiments were conducted in sextuplicate, and the IC_50_ was determined by linear regression of the curve.

### 3.6. MIC Determination

Colonies from BHI agar plates were suspended in saline solution (0.9% NaCl) and adjusted to an OD_600_ of 0.5. For growth kinetics determination, 96-well microplates (Starlab, Orsay, France) containing 200 μL of fresh BHI medium were inoculated with bacterial suspensions to an OD_600_ of 0.02 with a wide range of DltA inhibitors (from 0 to 2 mM). Plates were incubated at 37 °C with shaking (orbital amplitude of 3 mm) in a microplate reader (InfiniteM Nano, Tecan, Seoul, Republic of Korean). OD_600nm_ was measured every 10 min for 24 h. Determination of MIC/MBC of antibiotics and bacterial survival assays were performed as previously described using BHI medium [17,18,19]. The DltA inhibitor was diluted in pure water and added to the growth medium to a final concentration of 0, 0.1, 0.25, 0.5, 0.75, or 1 mM.

### 3.7. Quantification of D-Alanyl Ester in the Bacterial Cell Wall

Ester-linked D-alanines were quantified as described previously [48], with the following modifications. Cultures were grown in 5 mL of BHI supplemented with DltA inhibitor to a final concentration of 0.05, 0.1, 0.25, 0.5, 0.75, or 1 mM for 16 h at 37 °C under shaking (120 rpm). Briefly, the quantification is based on the oxidation of a chromogen by H_2_O_2_ generated when free D-alanines are oxidized by a D-amino acid oxidase. Three independent experiments with duplicate samples were performed.

## 4. Conclusions

In this paper, a new series of D-Ala-AMP analogues was synthesized and tested as adjuvants to re-sensitize MRSA to IPM. Modification of the 2′-position of adenosine was first envisaged to increase cell permeability and/or metabolic stability. Indeed, a fluorine atom, an azido group, a difluorophosphonylated allylic ether, and a glucose moiety were successfully introduced. Squaramide, sulfamide, and triazole derivatives were also prepared as phosphate surrogates, as well as fluorinated transbutenyl acyclonucleosides as sugar mimics. Functionalization of the *N*-terminal moiety to facilitate bacterial penetration was also realized to produce a series of *N*-acylated derivatives. The binding affinity of each molecule was measured towards *B. Subtilis* DltA (PDB 3FCC), revealing docking scores close to reference molecule **1**. Each new compound was tested in vitro as a DltA inhibitor. Indeed, we observed seven nucleosides containing modifications onto the carbohydrate or the linker, which had IC_50_ values in the same order of magnitude as the reference. Among them, two molecules containing either a difluorophosphonylated allylic ether onto the 2′-position or the sulfamide linker were able to re-sensitize MRSA to imipenem as efficiently as the reference. Quantification of D-alanyl esters confirmed that these two compounds reduced the level of bacterial cell wall D-alanyl residues by 50% and 80%. These new series represent a promising structure entry not reported yet for DltA inhibition and re-sensitization of bacteria towards known antibiotics. Further investigations will be realized to evaluate these new DltA inhibitors in MRSA-infected larvae of *Galleria mellonella* and will be reported in due course.

## Figures and Tables

**Table 1:** *N*-terminal functionalization of triazolyl nucleoside derivatives.

Entry	Products	
1	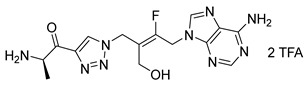	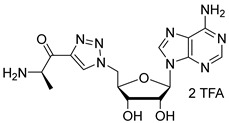
	**39**	**40**
2	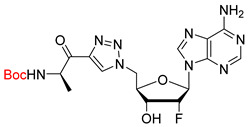	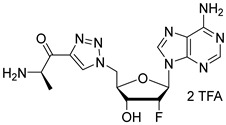
	**45**, 68%	**50**, 88%
3	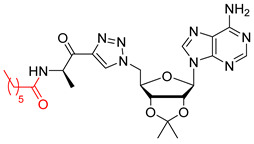	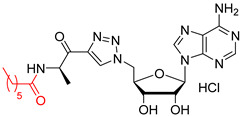
	**46**, 79%	**51**, 89%
4	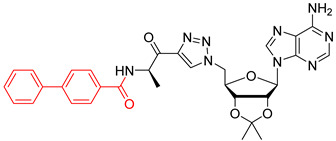	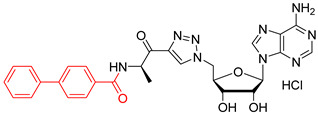
	**47**, 77%	**52**, 83%
5	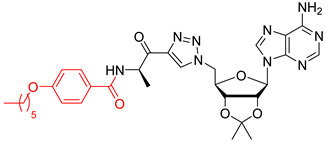	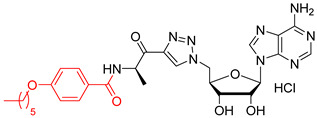
	**48**, 81%	**53**, 76%
6	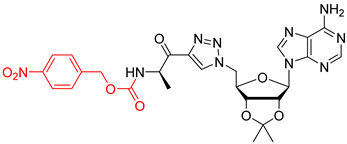	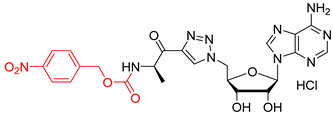
	**49**, 64%	**54**, 79%

**Table 2:** *In silico* binding affinity and biological studies.

Entry	Inhibitor	Docking Score (kcal/mol)	IC_50_ (μM)	MIC of IPM ^c^ (μg/mL)	Reduction in D-Ala Ester Residues (%)
1	**1**	−8.2	2.3 (0.4) ^a^, 2.4 (0.04) ^b^	0.25	86.7 (10.5)
2	**8**	−8.2	2.6 (0.5) ^a^	16	0
3	**9**	−8.5	3.8 (1.1) ^a^	8	74.9 (9.4)
4	**11**	−9.7	39.5 (0.9) ^b^	32	0
5	**18**	−8.8	7.6 (0.9) ^b^	0.25	51.5 (14.2)
6	**23**	−8.7	28.4 (3.7) ^a^	64	11.5 (1.3)
7	**30**	−8.2	1.8 (0.2) ^a^, 2.0 (0.3) ^b^	1	80.9 (18.4)
8	**31**	−8.3	3.2 (0.8) ^a^	32	0
9	**38**	−7.7	>100 ^b^	32	2
10	**39**	−8.1	7.4 (1.3) ^a^	16	0
11	**40**	−8.9	4.5 (2.6) ^a^	8	0
12	**50**	−9.0	14.7 (6.7) ^a^	32	8.6 (1.1)
13	**51**	−8.9	22.2 (3.5) ^b^	16	22.2 (5.0)
14	**52**	−10.5	12.4 (2.7) ^b^	8	16.0 (4.2)
15	**53**	−9.3	16 (2.4) ^b^	16	12.5 (14.0)
16	**54**	−10.0	21.9 (2.6) ^b^	16	0

^a^ DltA from *E. faecalis*; ^b^ Dlt from *S. epidermidis*, ^c^ experiments performed with *S. aureus.*

## Data Availability

The data are contained within the article and Supplementary Materials.

## Supplementary Materials

The following supporting information can be downloaded at https://www.mdpi.com/article/10.3390/molecules30122569/s1, Experimental procedure for the preparation of **35**, **S1**–**S10**, ^1^H, ^13^C, ^31^P and ^19^F NMR spectra of compounds **5**–**9**, **10**, **11**, **13**–**18**, **21**–**23**, **28**–**31**, **36**–**38**, **45**–**54**, **S2**–**S5**, **S7**–**S10**, HPLC chromatogram for final products **8**, **9**, **11**, **18**, **23**, **30**, **31**, **38**, **50**–**54**.
