# Supplementary material for: Synthesis of New DltA Inhibitors and Their Application as Adjuvant Antibiotics to Re-Sensitize Methicillin-Resistant Staphylococcus aureus
Source: Molecules. 2025 Jun 12;30(12):2569. doi: 10.3390/molecules30122569 (PMC12196251; doi:10.3390/molecules30122569)
Supplement: Supplementary file 1 [file molecules-30-02569-s001.zip › molecules-3682336-supplementary.pdf]

# Synthesis of new DltA inhibitors and their application as adjuvant antibiotics to re-sensitize methicillin resistant *Staphylococcus aureus*

**David Leparfait [1], Alexandre Mahé [2], Xiao Feng [1], Delphine Coupri [2], Fabien Le Cavelier [1], Nicolas Verneuil [2], Emmanuel Pfund [1,*], Aurélie Budin-Verneuil [2], and Thierry Lequeux [1,*]**

[1] ENSICAEN, Univ Caen Normandie, Univ Rouen Normandie, INSA Rouen Normandie, CNRS, Institut CARMeN UMR 6064, F-14050 Caen, France
[2] CBSA UR 4312, Université de Caen Normandie, F-14000 Caen, Normandy, France
* Correspondence: emmanuel.pfund@ensicaen.fr and thierry.lequeux@ensicaen.fr

## Supplementary Information

## Table of content

1 – General procedures ........ 2
2 – Synthesis ........ 3
  2.1 – Preparation of diene **35** ........ 3
  2.2 – Preparation of keto-alkynes **S6**-**S10** ........ 4
3 – NMR spectra and HPLC chromatogram ........ 8

## 1 – General procedures

### General procedure H for the preparation of Weinreb amides:

To a solution of amino acid (1 equiv.) in dioxane/$H_2O$ or THF/$H_2O$ (1:1) was added $NaHCO_3$ (2.0 equiv.) or $K_2CO_3$ (2.0 equiv.) at 0 °C. The mixture was stirred for 5 min at 0 °C and chloroformate (1.2 equiv.) or succinimidyl ester (1.2 equiv.) was added in one portion. The reaction mixture was stirred for 2 h at 0 °C and 20 h at 20 °C. The aqueous layer was extracted twice with $Et_2O$ and then acidified to pH = 2-3 with an aqueous solution of HCl (1 M) or citric acid (5%). The resulting solution was extracted three times with EtOAc. The combined organic layers were washed with $H_2O$, brine, dried over $MgSO_4$, filtered and concentrated under reduced pressure to give the desired product which was used without further purification. To a solution of crude *N*-acylated D-alanine (1.0 equiv.) in $CH_2Cl_2$ (0.4 M) were added EDCI (1.5 equiv.) and HOBt (1.5 equiv.) at 20 °C. The mixture was stirred for 30 min. Then, *N*,*O*-dimethylhydroxylamine (1.5 equiv.) was added followed by dropwise addition of DIPEA (2.0 equiv.). The reaction mixture was stirred for 20 h at 20 °C and quenched with $H_2O$. The aqueous layer was extracted three times with $CH_2Cl_2$ and the combined organic layers were washed with an aqueous solution of HCl (1 M), $H_2O$, three time with brine, dried over $MgSO_4$, filtered and evaporated. The crude product was purified by column chromatography on silica gel to give the desired product.

### General procedure I for the preparation of keto-alkynes:

To a solution of Weinreb amide (1.0 equiv.) in THF (0.1 M) was slowly added a solution (0.5 M in THF) of Grignard's reagent (4.0 equiv.) at –78 °C *via* syringe pump (1 mL/min). The reaction mixture was stirred at –78 °C for 1 h and 12 h at 20 °C. The mixture was poured into a cold $NaHSO_4$ solution (0.1 M, 15 mL) at 0 °C and stirred for 1 h at 20 °C. The solvent was removed under reduced pressure and the resulting aqueous layer was extracted twice with $Et_2O$. The combined organic layers were washed successively with an aqueous solution of $NaHSO_4$ (0.1 M), $NaHCO_3$ sat and brine. The organic layer was dried over $Na_2SO_4$, filtered and concentrated under reduced pressure. The crude was purified by column chromatography on silica gel to give the desired product.

2 – Synthesis

## 2.1 – Preparation of diene **35**

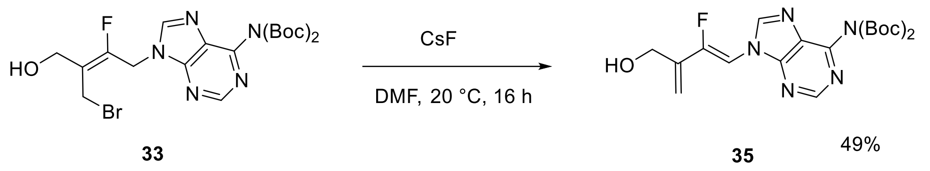


To a solution of compound **29** (400 mg, 0.78 mmol) in DMF (7 mL) was added cesium fluoride (235 mg, 1.55 mmol) at 20 °C. The mixture was stirred for 16 h then the solvent was evaporated under reduced pressure. The residue was taken up with $H_2O$/$Et_2O$ and the aqueous layer was extracted three times with $Et_2O$. The combined organic layers were washed with brine, dried over $MgSO_4$, filtered and concentrated under reduced pressure. The crude product was purified by column chromatography on silica gel (Eluent Pentane/EtOAc 1:1) to give compound **31** (165 mg, 0.38 mmol, 49%) as colorless waxy oil. $^1H$ NMR ($CDCl_3$, 500 MHz): δ 1.43 (s, 18H), 4.43 (br s, 2H), 5.54 (d, *J* = 3.5 Hz, 1H), 5.72 (s, 1H), 7.19 (d, *J* = 30.8 Hz, 1H), 8.65 (s, 1H), 8.87 (s, 1H). $^{19}F$ NMR ($CDCl_3$, 470 MHz): δ – 123.3 (d, *J* = 30.8 Hz). $^{13}C$ NMR ($CDCl_3$, 125 MHz): δ 27.9, 62.2 (d, *J* = 4.9 Hz), 84.1, 101.3 (d, *J* = 9.2 Hz), 116.6 (d, *J* = 6.3 Hz), 127.7, 136.9 (d, *J* = 18.6 Hz), 143.8 (d, *J* = 15.9 Hz), 150.3, 150.6 (dd, *J* = 256.1 Hz), 150.8, 152.5, 152.6. HRMS-ESI (m/z) calcd for $C_{20}H_{26}FN_5O_5Na$ [M+Na]$^+$ 458.1816, found 458.1811.

## 2.2 – Preparation of keto-alkynes **S6-S10**

The synthesis was realized according to the following synthetic scheme:

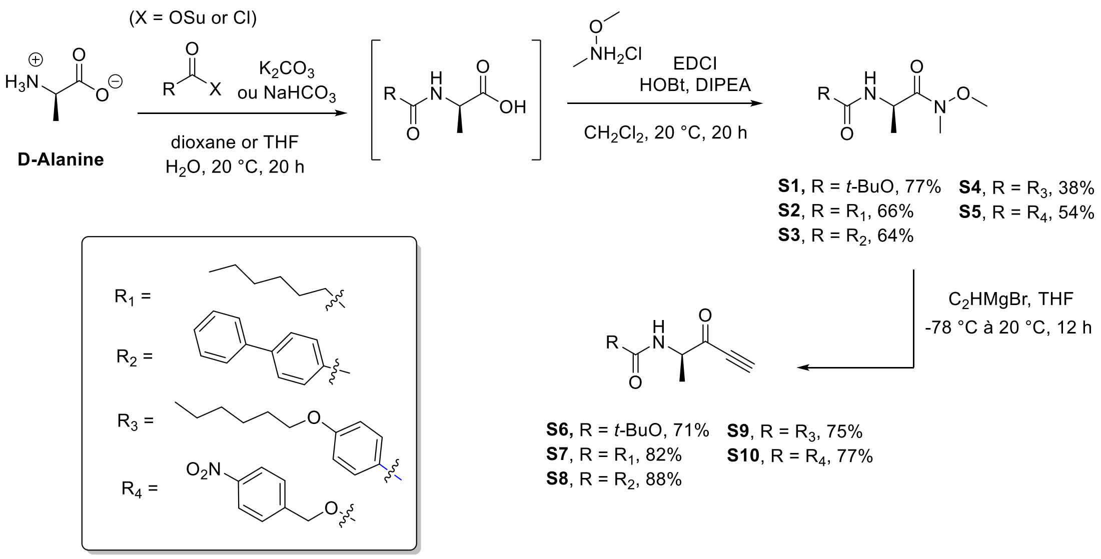


*Weinreb amide **S1***: To a solution of *N*-Boc-D-Ala (600 mg, 3.17 mmol) in $CH_2Cl_2$ (8.6 mL) were added EDCI (910 mg, 4.76 mmol) and HOBt (640 mg, 4.76 mmol) at 20 °C. The mixture was stirred for 30 min. Then, *N,O*-dimethylhydroxylamine (460 mg, 4.76 mmol) was added followed by dropwise addition of DIPEA (1.1 mL, 6.34 mmol). The reaction mixture was stirred for 20 h at 20 °C and quenched with $H_2O$. The aqueous layer was extracted three times with $CH_2Cl_2$. The combined organic layers were washed with an aqueous solution of HCl (1 M), $H_2O$, three time with brine, dried over $MgSO_4$, filtered and evaporated to give compound **S1** (565 mg, 2.44 mmol, 77%) as a white solid. $^1H$ NMR ($CDCl_3$, 500 MHz): δ 1.27 (d, *J* = 7.1 Hz, 3H), 1.39 (s, 9H), 3.17 (s, 3H), 3.73 (s, 3H), 4.60-4.68 (m, 1H), 5.25 (br s, 1H). $^{13}C$ NMR ($CDCl_3$, 125 MHz): δ18.7, 28.4, 32.2, 46.6, 61.7, 79.5, 155.2, 173.7. HRMS-ESI (m/z) calcd for $C_{10}H_{20}N_2O_4Na$ $[M+Na]^+$ 255.1321, found 255.1311.

*Weinreb amide **S2***: The general procedure H was followed with D-Alanine (1.4 g, 16.37 mmol), $K_2CO_3$ (3.7 g, 27.28 mmol), 2,5-Dioxopyrrolidin-1-yl heptanoate (3.1 g, 13.64 mmol), THF/$H_2O$ (80 mL), crude *N*-acylated alanine (2.7 g, 13.66 mmol), EDCI (3.92 g, 20.49 mmol), HOBt (2.77 g, 20.49 mmol), *N,O*-dimethylhydroxylamine (2.00 g, 20.49 mmol), DIPEA (4.73 mL, 27.32 mmol) and $CH_2Cl_2$ (46 mL). The crude product was purified by column chromatography on silica gel (Eluent EtOAc/Pentane 6:4) to give compound **S2** (2.2 g, 9.00 mmol, 66%) as a colorless oil. $^1H$ NMR ($CDCl_3$, 500 MHz): δ 0.84 (t, *J* = 6.6 Hz,

3H), 1.23–1.30 (m, 6H), 1.29 (t, *J* = 7.1 Hz, 3H), 1.55–1.62 (m, 2H), 2.16 (t, *J* = 7.6 Hz, 2H), 3.18 (s, 3H), 3.74 (s, 3H), 4.91–4.95 (m, 1H), 6.38 (br s, 1H). $^{13}$C NMR ($CDCl_3$, 125 MHz): δ 14.1, 18.5, 22.5, 25.6, 29.0, 31.6, 32.2, 36.7, 45.4, 61.7, 172.8, 173.4. HRMS-ESI (m/z) calcd for $C_{12}H_{24}N_2O_3Na$ $[M+Na]^+$ 267.1685, found 267.1682.

*Weinreb amide* ***S3***: The general procedure H was followed with D-Alanine (535 mg, 6.01 mmol), $K_2CO_3$ (1.38 mg, 10.02 mmol), (2,5-dioxopyrrolidin-1-yl)4-phenylbenzoate (1.5 g, 5.01 mmol), THF/$H_2O$ (80 mL), crude *N*-acylated alanine (1.1 g, 3.71 mmol), EDCI (1.1 g, 5.57 mmol), HOBt (752 mg, 5.57 mmol), *N,O*-dimethylhydroxylamine (543 mg, 5.57 mmol), DIPEA (1.3 mL, 7.42 mmol) and $CH_2Cl_2$ (13 mL). The crude product was purified by column chromatography on silica gel (Eluent EtOAc/Pentane 1:1) to give compound **S3** (913 mg, 2.37 mmol, 64%) as a waxy oil. $^1$H NMR ($CDCl_3$, 500 MHz): δ 1.47 (t, *J* = 6.9 Hz, 3H), 3.25 (s, 3H), 3.82 (s, 3H), 5.16–5.21 (m, 1H), 7.19 (br s, 1H), 7.35–7.39 (m, 1H), 7.43–7.46 (m, 2H), 7.59–7.61 (m, 2H), 7.63–7.65 (m, 2H), 7.88–7.90 (m, 2H). $^{13}$C NMR ($CDCl_3$, 125 MHz): δ 18.6, 32.3, 46.1, 61.8, 127.2 (2C), 127.3 (2C), 127.7 (2C), 129.0 (2C), 132.8, 140.1, 144.4, 166.4, 173.3. HRMS-ESI (m/z) calcd for $C_{18}H_{20}N_2O_3Na$ $[M+Na]^+$ 335.1372, found 335.1369.

*Weinreb amide* ***S4***: The general procedure H was followed with D-Alanine (500 mg, 5.61 mmol), $NaHCO_3$ (940 mg, 11.22 mmol), n-hexyloxybenzoyl chloride (1.6 g, 6.73 mmol), dioxane/$H_2O$ (20 mL), crude *N*-acylated alanine (911 mg, 3.10 mmol), EDCI (891 mg, 4.65 mmol), HBOt (628 mg, 4.65 mmol), *N,O*-dimethylhydroxylamine (453 mg, 4.65 mmol), DIPEA (1.1 mL, 6.20 mmol) and $CH_2Cl_2$ (10 mL). The crude product was purified by column chromatography on silica gel (Eluent EtOAc/Cyclohexane 1:1) to give compound **S4** (710 mg, 2.11 mmol, 38%) as a white solid. $^1$H NMR ($CDCl_3$, 500 MHz): δ 0.90 (t, *J* = 7.1 Hz, 3H), 1.31–1.35 (m, 4H), 1.43 (t, *J* = 7.1 Hz, 3H), 1.41–1.47 (m, 2H), 1.75–1.80 (m, 2H), 3.23 (s, 3H), 3.80 (s, 3H), 3.97 (t, *J* = 6.6 Hz, 2H), 5.11–5.17 (m, 1H), 6.87–6.90 (m, 2H), 6.95 (br s, 1H), 7.74–7.77 (m, 2H). $^{13}$C NMR ($CDCl_3$, 125 MHz): δ 14.1, 18.8, 22.7, 25.8, 29.2, 31.7, 32.3, 46.0, 61.8, 68.3, 114.3 (2C), 126.2, 129.0 (2C), 162.0, 166.3, 173.5. HRMS-ESI (m/z) calcd for $C_{18}H_{28}N_2O_4Na$ $[M+Na]^+$ 359.1947, found 359.1945.

*Weinreb amide* ***S5****:* The general procedure H was followed with D-Alanine (500 mg, 5.61 mmol), $NaHCO_3$ (940 mg, 11.22 mmol), nitrobenzylchloroformate (1.4 g, 6.73 mmol), dioxane/$H_2O$ (20 mL), crude *N*-acylated alanine (1.3 g, 4.85 mmol), EDCI (1.4 g, 7.26 mmol), HOBt (981 mg, 7.26 mmol), *N,O*-dimethylhydroxylamine (708 mg, 7.26 mmol), DIPEA (1.7 mL, 9.70 mmol) and $CH_2Cl_2$ (13.1 mL). The

crude product was purified by column chromatography on silica gel (Eluent EtOAc/Pentane 6:4) to give compound **S5** (927 mg, 3.00 mmol, 54%) as a white solid. $^{1}$H NMR ($CDCl_3$, 500 MHz): δ 1.36 (d, *J* = 6.9 Hz, 3H), 3.22 (s, 3H), 3.76 (s, 3H), 4.69–4.75 (m, 1H), 5.19 (s, 2H), 5.66 (br s, 1H), 7.50 (d, *J* = 7.6 Hz, 2H), 8.20 (d, *J* = 7.6 Hz, 2H). $^{13}$C NMR ($CDCl_3$, 125 MHz): δ 18.7, 32.3, 47.5, 61.8, 65.3, 123.9 (2C), 128.1 (2C), 144.1, 147.7, 155.3, 173.1. HRMS-ESI (m/z) calcd for $C_{13}H_{17}N_3O_6Na$ $[M+Na]^+$ 334.1015, found 334.1011.

*Keto-alkyne **S6**:* The general procedure I was followed with compound **S1** (200 mg, 0.86 mmol), ethynylmagnesium bromide (6.9 mL, 3.44 mmol) and THF (8.6 mL). The crude product was purified by column chromatography on silica gel (Eluent Pentane/EtOAc 8:2) to give compound **S6** (120 mg, 0.61 mmol, 71%) as a brownish solid. $^{1}$H NMR ($CDCl_3$, 500 MHz): δ 1.42 (s, 9H), 3.37 (s, 1H), 4.37-4.40 (m, 1H), 5.14 (br s, 1H). $^{13}$C NMR ($CDCl_3$, 125 MHz): δ 17.3, 28.4, 57.0, 79.5, 80.2, 82.0, 155.1, 186.8. HRMS-ESI (m/z) calcd for $C_{10}H_{15}NO_3Na$ $[M+Na]^+$ 220.0950, found 220.0952.

*Keto-alkyne **S7**:* The general procedure I was followed with compound **S2** (1.00 g, 4.09 mmol), ethynylmagnesium bromide (32.7 mL, 16.36 mmol) and THF (45 mL). The crude product was purified by column chromatography on silica gel (Eluent Pentane/EtOAc 7:3) to give compound **S2** (702 mg, 3.35 mmol, 82%) as a yellow oil. $^{1}$H NMR ($CDCl_3$, 500 MHz): δ 0.86 (t, *J* = 7.3 Hz, 3H), 1.25–1.32 (m, 6H), 1.45 (d, *J* = 7.2 Hz, 3H), 1.58–1.64 (m, 2H), 2.19–2.23 (m, 2H), 3.39 (s, 1H), 4.66–4.73 (m, 1H), 6.19 (br s, 1H). $^{13}$C NMR ($CDCl_3$, 125 MHz): δ 14.1, 17.4, 22.6, 25.6, 29.0, 31.6, 36.6, 55.9, 79.5, 82.2, 172.9, 186.2. HRMS-ESI (m/z) calcd for $C_{12}H_{19}NO_2Na$ $[M+Na]^+$ 232.1313, found 232.1310.

*Keto-alkyne **S8**:* The general procedure I was followed with compound **S3** (900 mg, 2.88 mmol), ethynylmagnesium bromide (23 mL, 11.52 mmol) in THF (32 mL). The crude product was purified by column chromatography on silica gel (Eluent Pentane/EtOAc 7:3) to give compound **S8** (708 mg, 2.55 mmol, 88%) as a white solid. $^{1}$H NMR ($CDCl_3$, 500 MHz): δ 1.62 (t, *J* = 7.1 Hz, 3H), 3.43 (s, 1H), 4.92–4.98 (m, 1H), 6.88 (br s, 1H), 7.38–7.41 (m, 1H), 7.45–7.49 (m, 2H), 7.60–7.63 (m, 2H), 7.66–7.69 (m, 2H), 7.88–7.91 (m, 2H). $^{13}$C NMR ($CDCl_3$, 125 MHz): δ 17.7, 56.5, 79.5, 82.6, 127.4 (2C), 127.5 (2C), 127.8 (2C), 128.2, 129.1 (2C), 132.5, 140.1, 144.9, 166.7, 186.1. HRMS-ESI (m/z) calcd for $C_{18}H_{15}NO_2Na$ $[M+Na]^+$ 300.1000, found 300.0997.

*Keto-alkyne **S9**:* The general procedure I was followed with compound **S4** (680 mg, 2.11 mmol), ethynylmagnesium bromide (16.9 mL, 8.43 mmol) and THF (24 mL). The crude product was purified by column chromatography on silica gel (Eluent Pentane/EtOAc 7:3) to give compound **S9** (477 mg, 1.58

mmol, 75%) as a brown solid. $^{1}$H NMR ($CDCl_3$, 500 MHz): δ 0.91 (t, *J* = 7.1 Hz, 3H), 1.32–1.36 (m, 4H), 1.43–1.49 (m, 2H), 1.58 (d, *J* = 7.1 Hz, 3H), 1.76–1.82 (m, 2H), 3.41 (s, 1H), 3.99 (t, *J* = 6.6 Hz, 2H), 4.87–4.93 (m, 1H), 6.75 (br s, 1H), 6.90–6.93 (m, 2H), 7.74–7.78 (m, 2H). $^{13}$C NMR ($CDCl_3$, 125 MHz): δ 14.2, 17.7, 22.7, 25.8, 29.2, 31.7, 56.4, 68.4, 79.5, 82.4, 114.4 (2C), 125.8, 129.1 (2C), 162.3, 166.5, 186.3. HRMS-ESI (m/z) calcd for $C_{18}H_{24}NO_3$ [M+H]$^+$ 302.1756, found 302.1751.

*Keto-alkyne **S10**:* The general procedure I was followed with compound **S5** (650 mg, 2.08 mmol), ethynylmagnesium bromide (16.6 mL, 8.35 mmol) and THF (22 mL). The crude product was purified by column chromatography on silica gel (Eluent Pentane/EtOAc 7:3) to give compound **S10** (447 mg, 1.62 mmol, 77%) as a brown solid. $^{1}$H NMR ($CDCl_3$, 500 MHz): δ 1.50 (d, *J* = 7.3 Hz, 3H), 3.42 (s, 1H), 4.46–4.52 (m, 1H), 5.20 (s, 2H), 5.52 (br s, 1H), 7.49–7.52 (m, 2H), 8.19–8.22 (m, 2H). $^{13}$C NMR ($CDCl_3$, 125 MHz): δ 17.5, 57.6, 65.6, 79.3, 82.7, 123.9 (2C), 128.2 (2C), 143.8, 147.8, 155.2, 185.7. HRMS-ESI (m/z) calcd for $C_{13}H_{12}N_2O_5Na$ [M+Na]$^+$ 299.0644, found 299.0640.

## 3 – NMR spectra and HPLC chromatogram

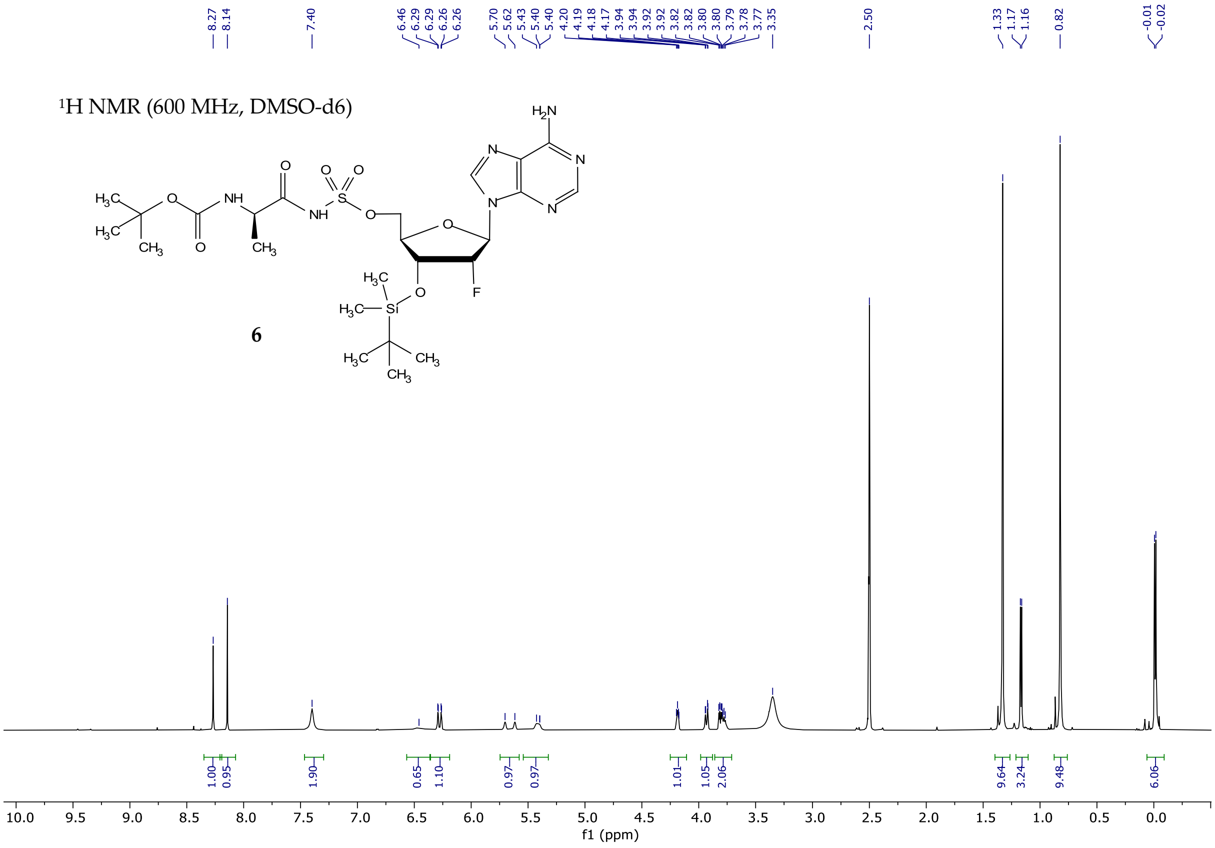


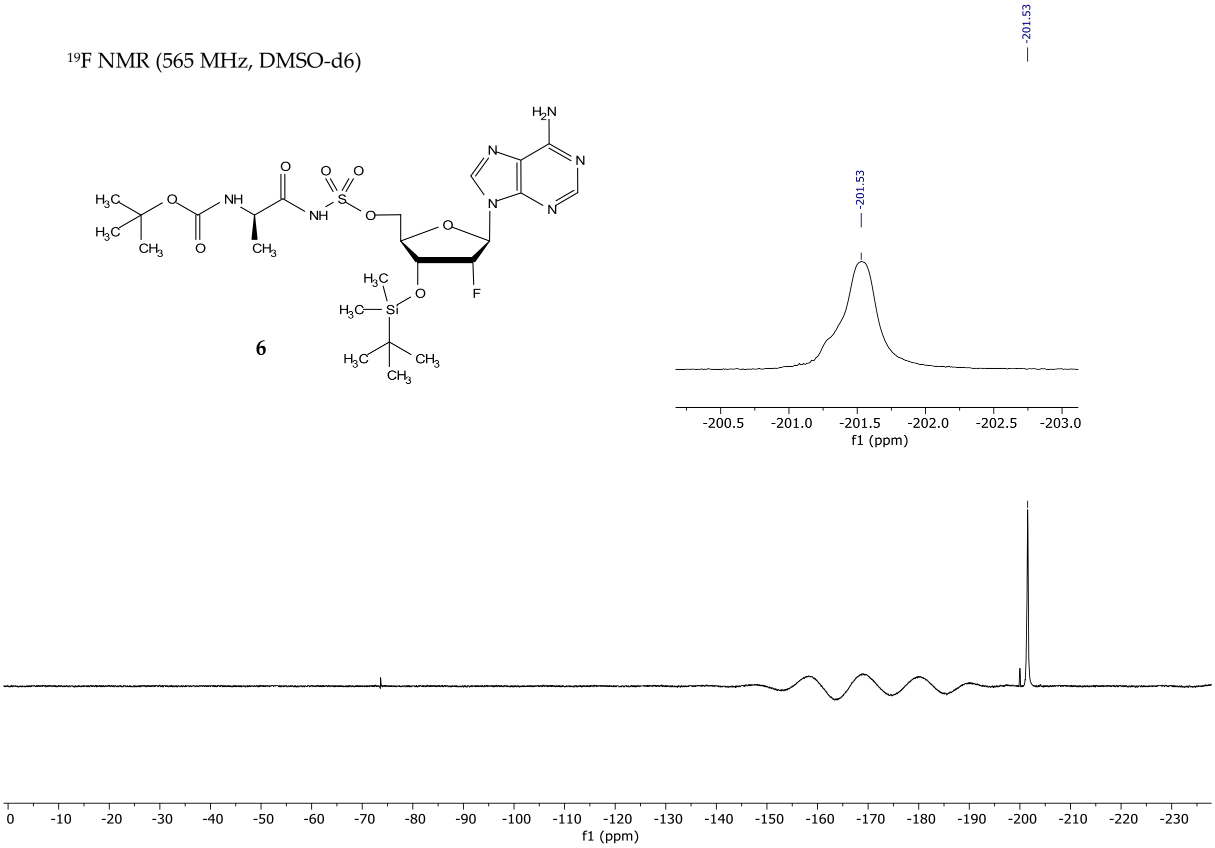

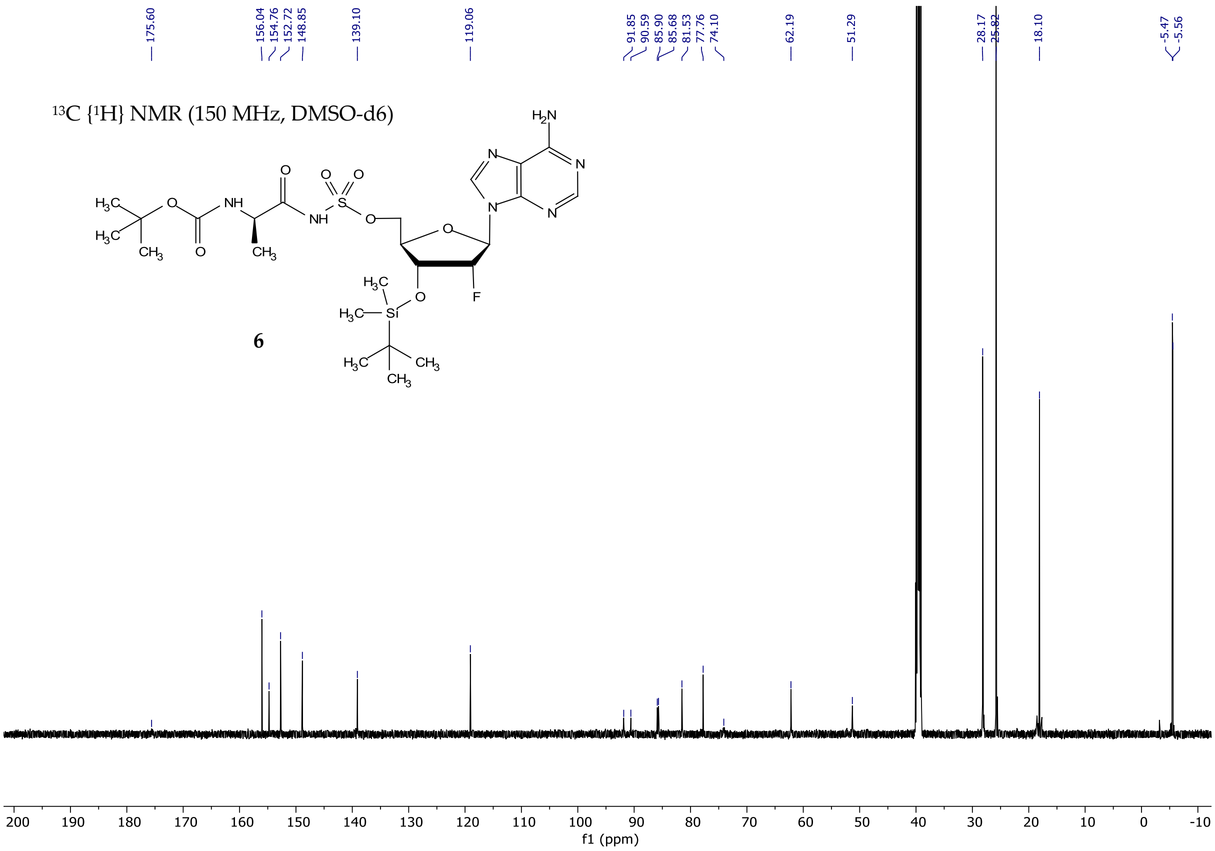
13C {1H} NMR (150 MHz, DMSO-d6)
175.60
156.04
154.76
152.72
148.85
139.10
119.06
91.85
90.59
85.90
85.68
81.53
77.76
74.10
62.19
51.29
28.17
25.82
18.10
-5.47
-5.56
6
200 190 180 170 160 150 140 130 120 110 100 90 80 70 60 50 40 30 20 10 0 -10
f1 (ppm)

$^1H$ NMR (500 MHz, $D_2O$)

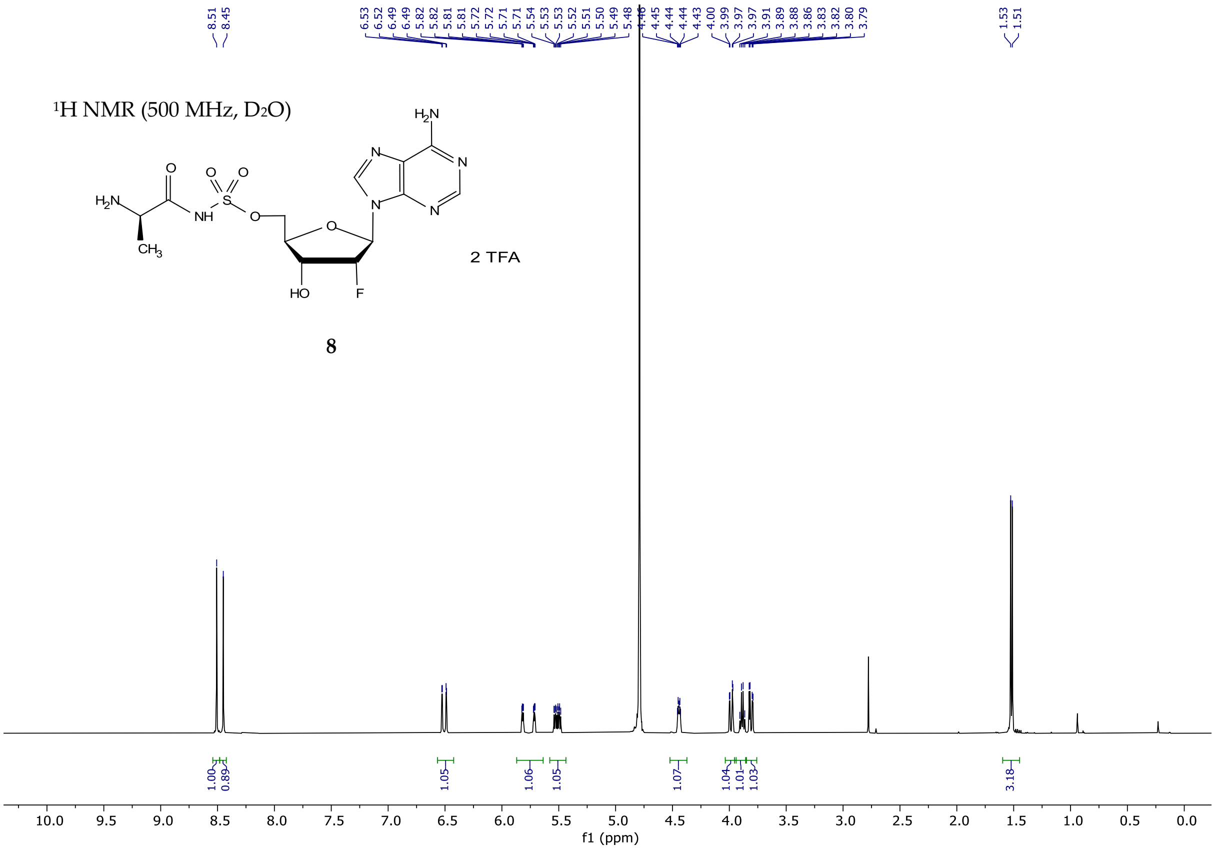


**8**

$^{19}F$ NMR (470 MHz, $D_2O$)

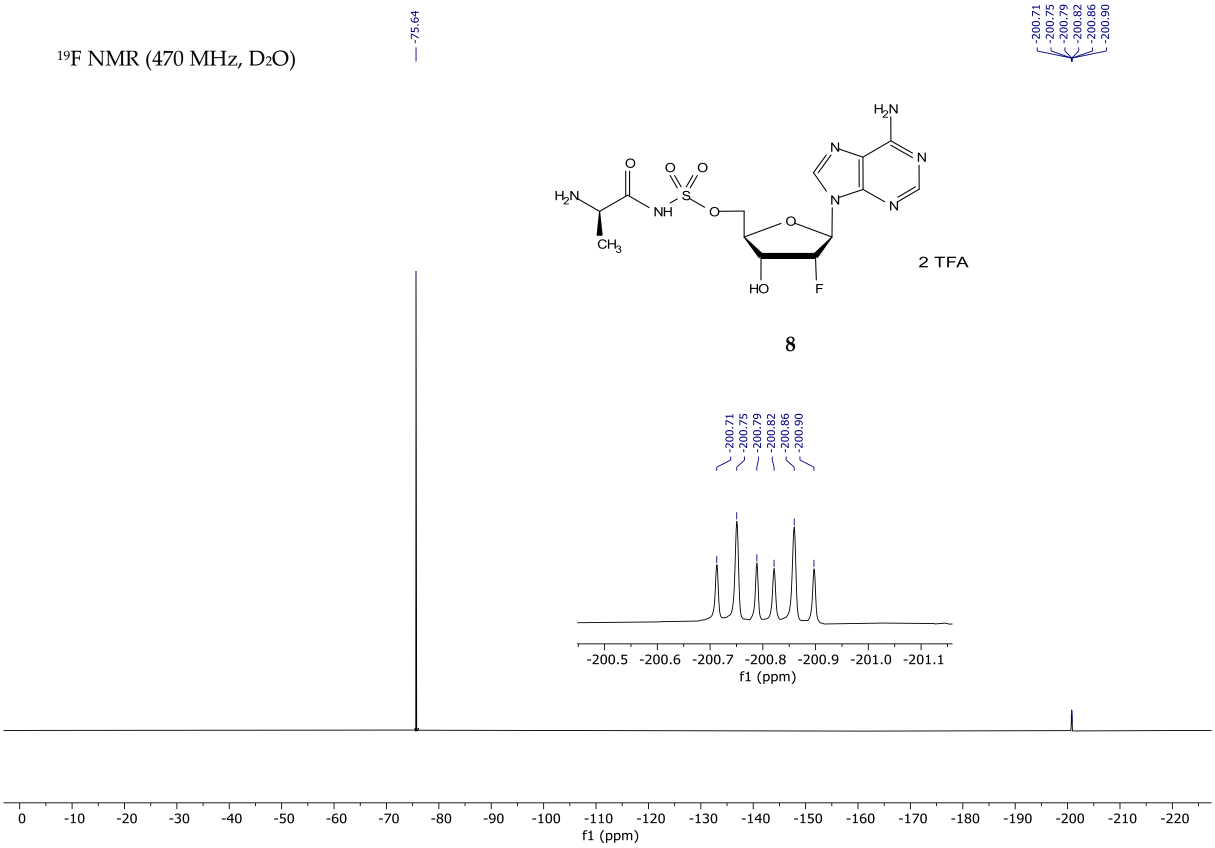


**8**

$^{13}C$ {$^{1}H$} NMR (150 MHz, $D_2O$)

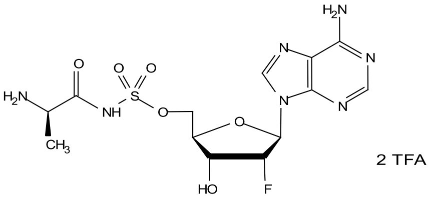


**8**

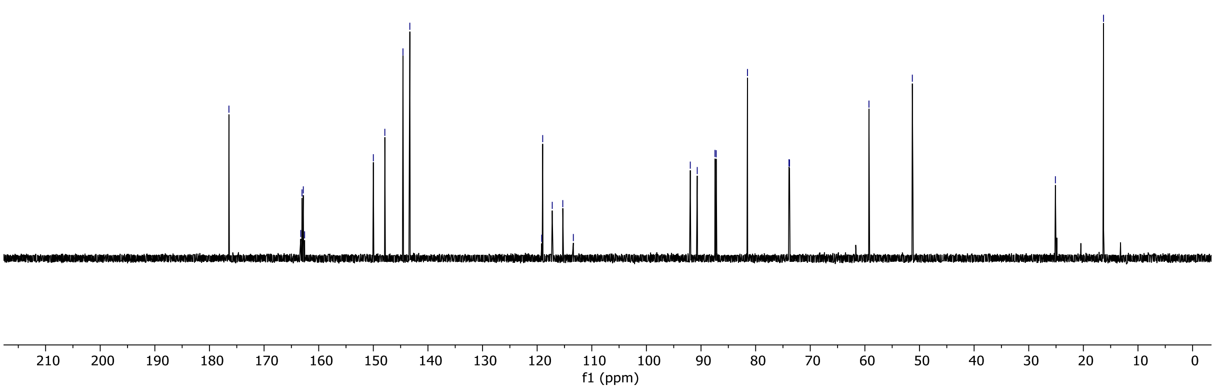

RAPPORT HPLC

Empower Node Lcmt-hplc3

**X-terra 2018**

**Colonne Waters XterraMS C18 3.5µm 4.6x150mm**

# DL306

Date Acquired 05/10/2021 11:36:26 CET

**Instrument Method: Grad 9802 40min**

*Stored: 01/01/2002 00:59:04 CET*

*Revision*
This method cc

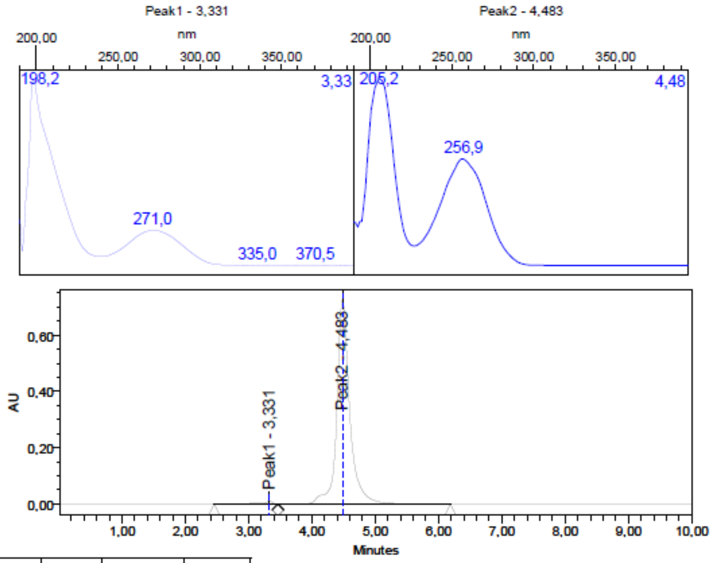


| | Peak Name | RT | Area | % Area |
|---|---|---|---|---|
| 1 | Peak1 | 3,331 | 150109 | 1,39 |
| 2 | Peak2 | 4,483 | 10451958 | 96,57 |
| 3 | Peak3 | 21,094 | 221518 | 2,05 |

**PDA 254,0 nm**

Reported by User: System
Report Method: RAPPORT HPLC
Report Method ID: !5834
Page: 1 of 1

Project Name: X-terra 2018
Date Printed:
06/10/2021
09:36:49 Europe/Paris

HPLC analysis for compound **8**

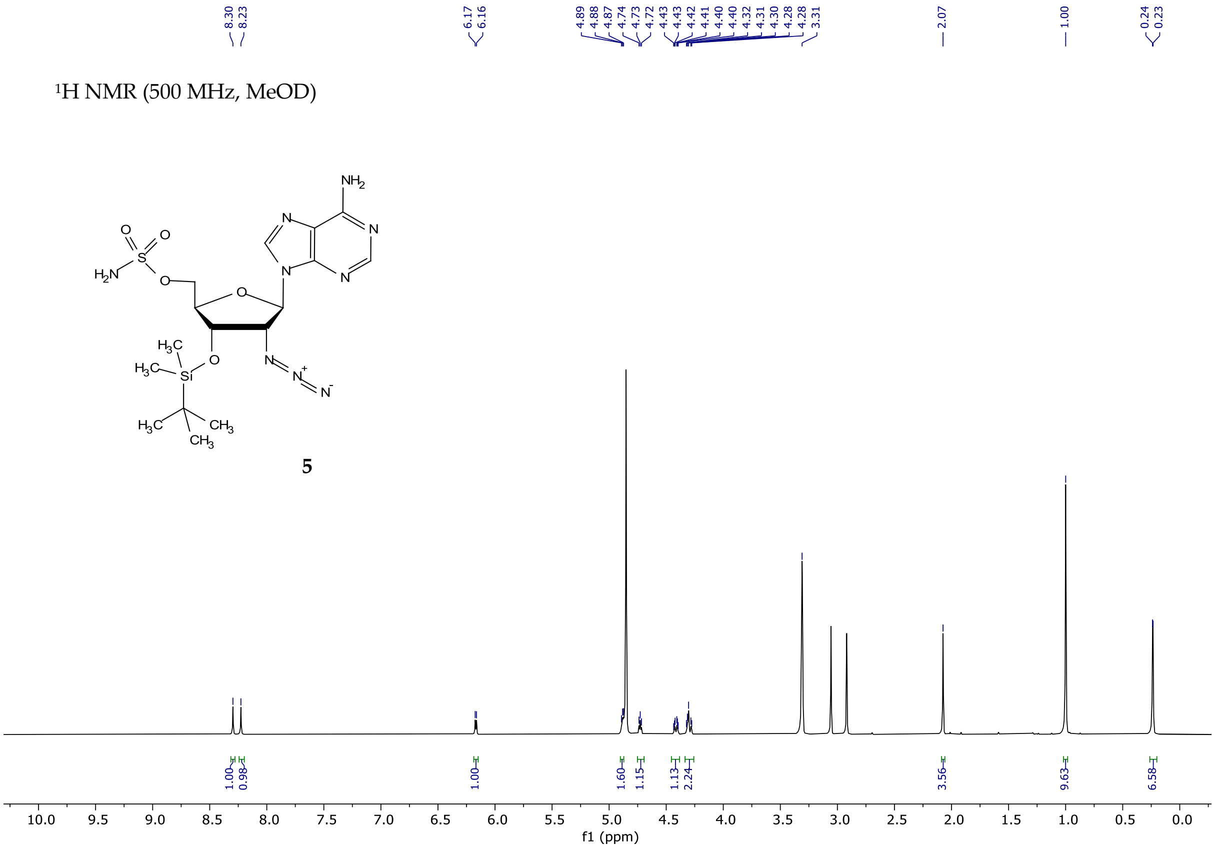
1H NMR (500 MHz, MeOD)
8.30
8.23
6.17
6.16
4.89
4.88
4.87
4.74
4.73
4.72
4.43
4.43
4.42
4.41
4.40
4.40
4.32
4.31
4.30
4.28
4.28
3.31
2.07
1.00
0.24
0.23
NH2
O
S
H2N
N
Si
H3C
CH3
5
1.00
0.98
1.00
1.60
1.15
1.13
2.24
3.56
9.63
6.58
f1 (ppm)

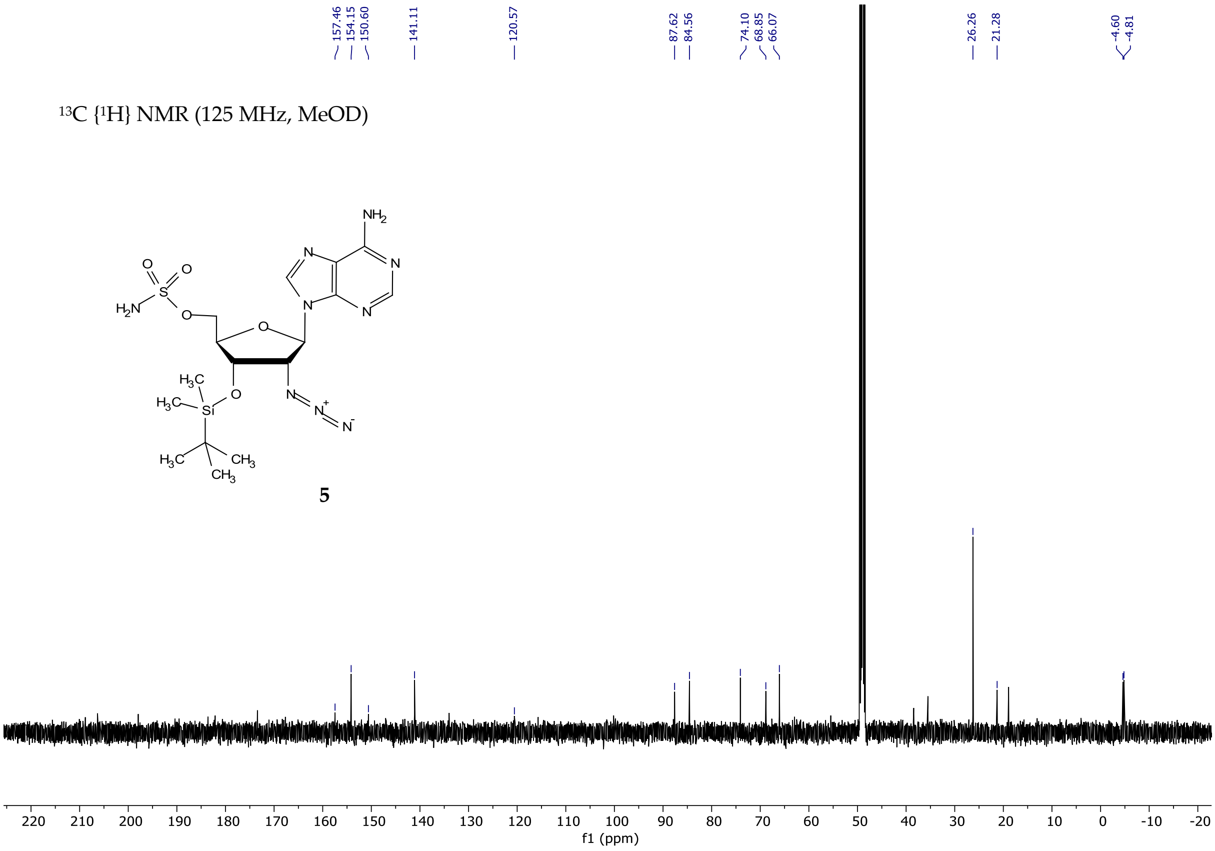
13C {1H} NMR (125 MHz, MeOD)
157.46
154.15
150.60
141.11
120.57
87.62
84.56
74.10
68.85
66.07
26.26
21.28
-4.60
-4.81
NH2
O
S
H2N
N
Si
H3C
CH3
5
f1 (ppm)

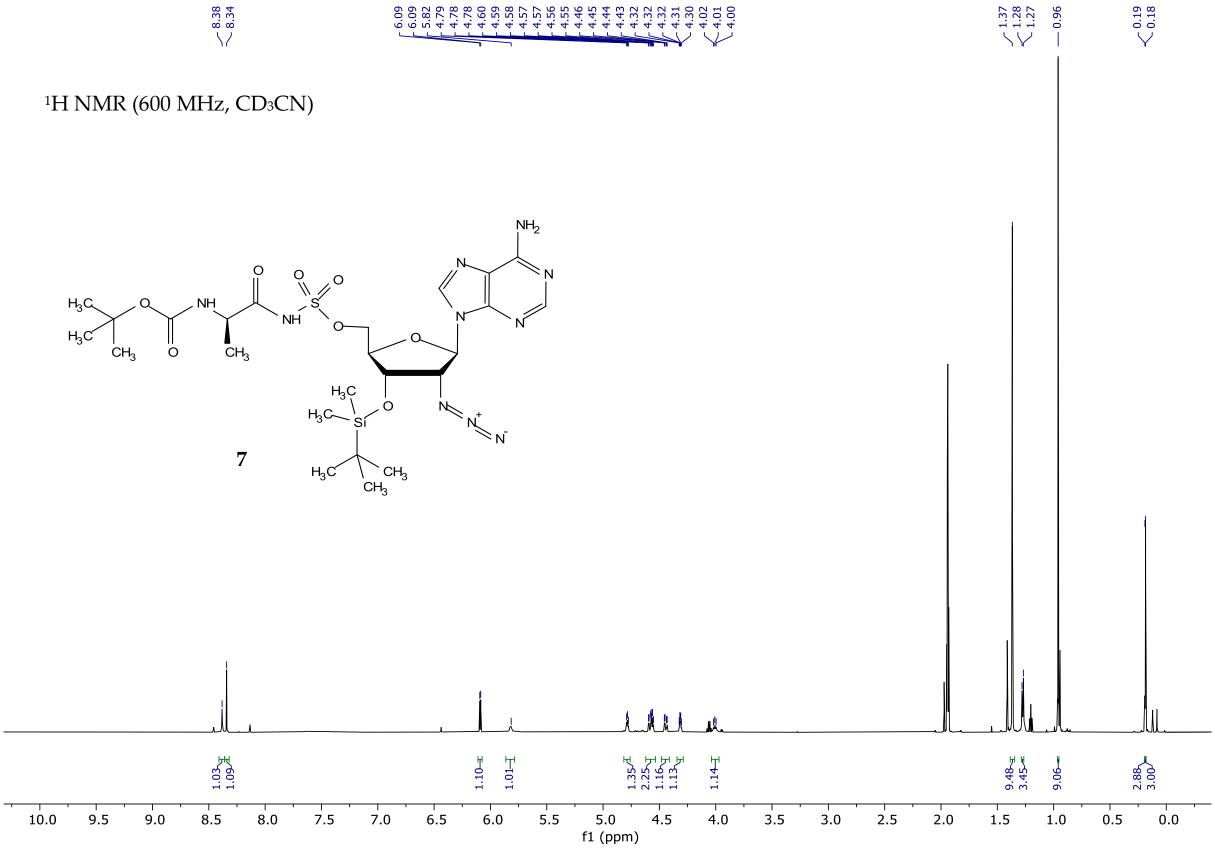

8.38
8.34
6.09
6.09
5.82
4.79
4.78
4.78
4.60
4.59
4.58
4.57
4.57
4.56
4.55
4.46
4.45
4.44
4.43
4.32
4.32
4.32
4.31
4.30
4.02
4.01
4.00
1.37
1.28
1.27
0.96
0.19
0.18
1H NMR (600 MHz, CD3CN)
7
1.03
1.09
1.10
1.01
1.35
2.25
1.16
1.13
1.14
9.48
3.45
9.06
2.88
3.00
f1 (ppm)


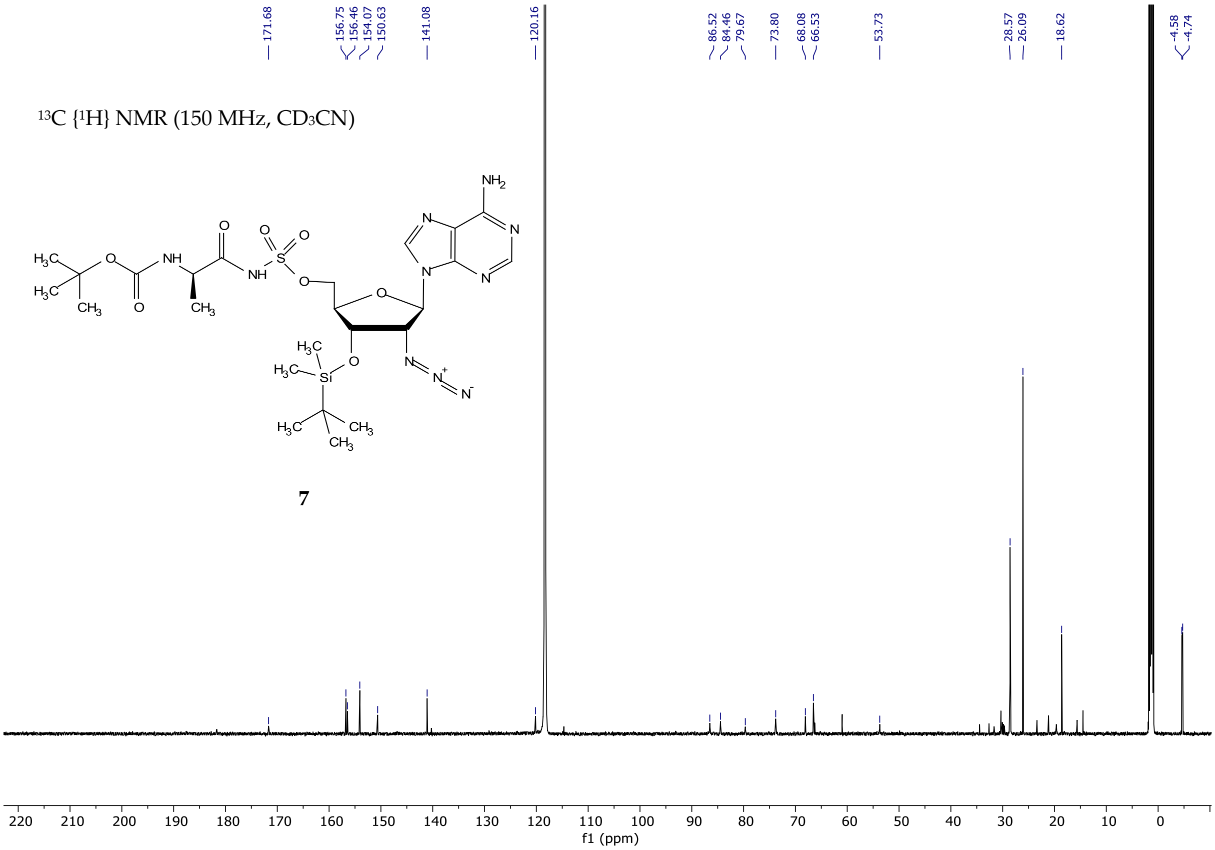

171.68
156.75
156.46
154.07
150.63
141.08
120.16
86.52
84.46
79.67
73.80
68.08
66.53
53.73
28.57
26.09
18.62
-4.58
-4.74
13C {1H} NMR (150 MHz, CD3CN)
7
f1 (ppm)

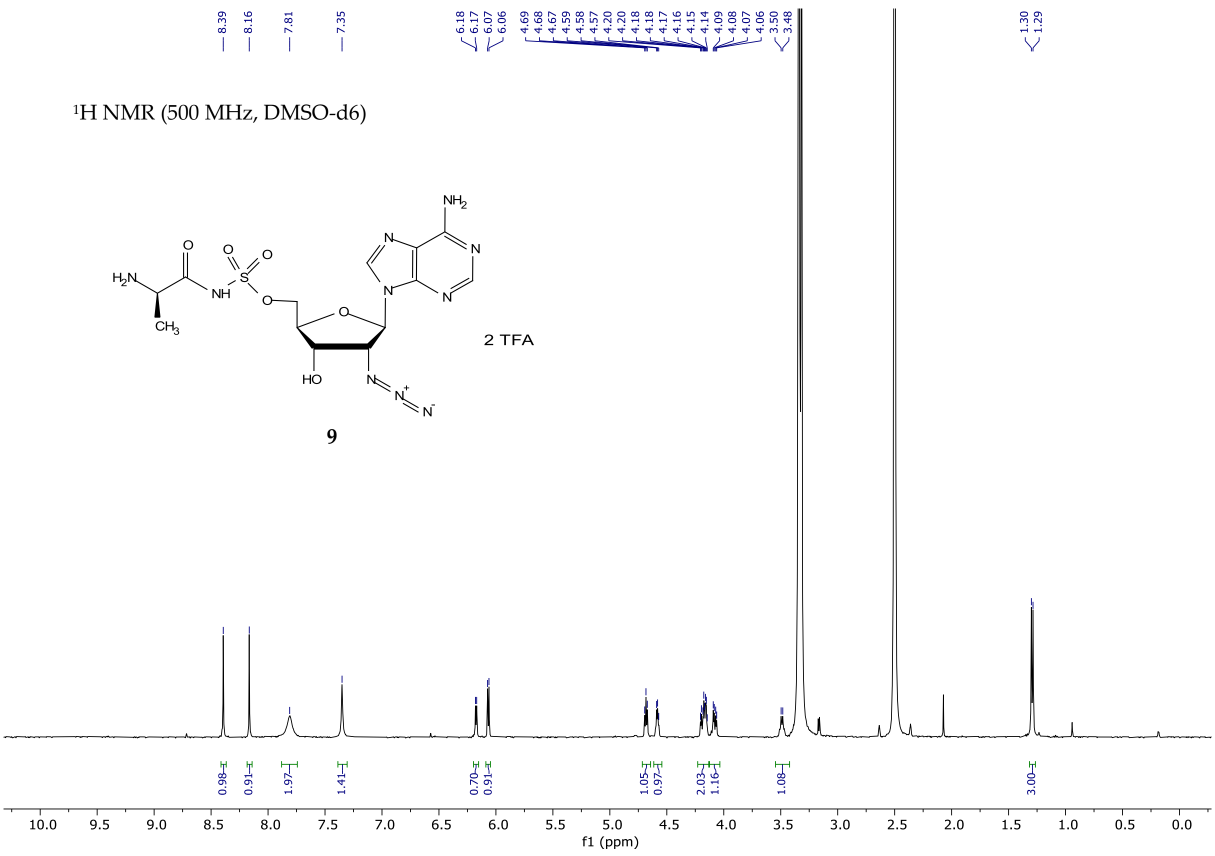

1H NMR (500 MHz, DMSO-d6)
8.39
8.16
7.81
7.35
6.18
6.17
6.07
6.06
4.69
4.68
4.67
4.59
4.58
4.57
4.20
4.20
4.18
4.18
4.17
4.16
4.15
4.14
4.09
4.08
4.07
4.06
3.50
3.48
1.30
1.29
0.98
0.91
1.97
1.41
0.70
0.91
1.05
0.97
2.03
1.16
1.08
3.00
NH2
O
H2N
NH
S
CH3
N
HO
2 TFA
9
f1 (ppm)


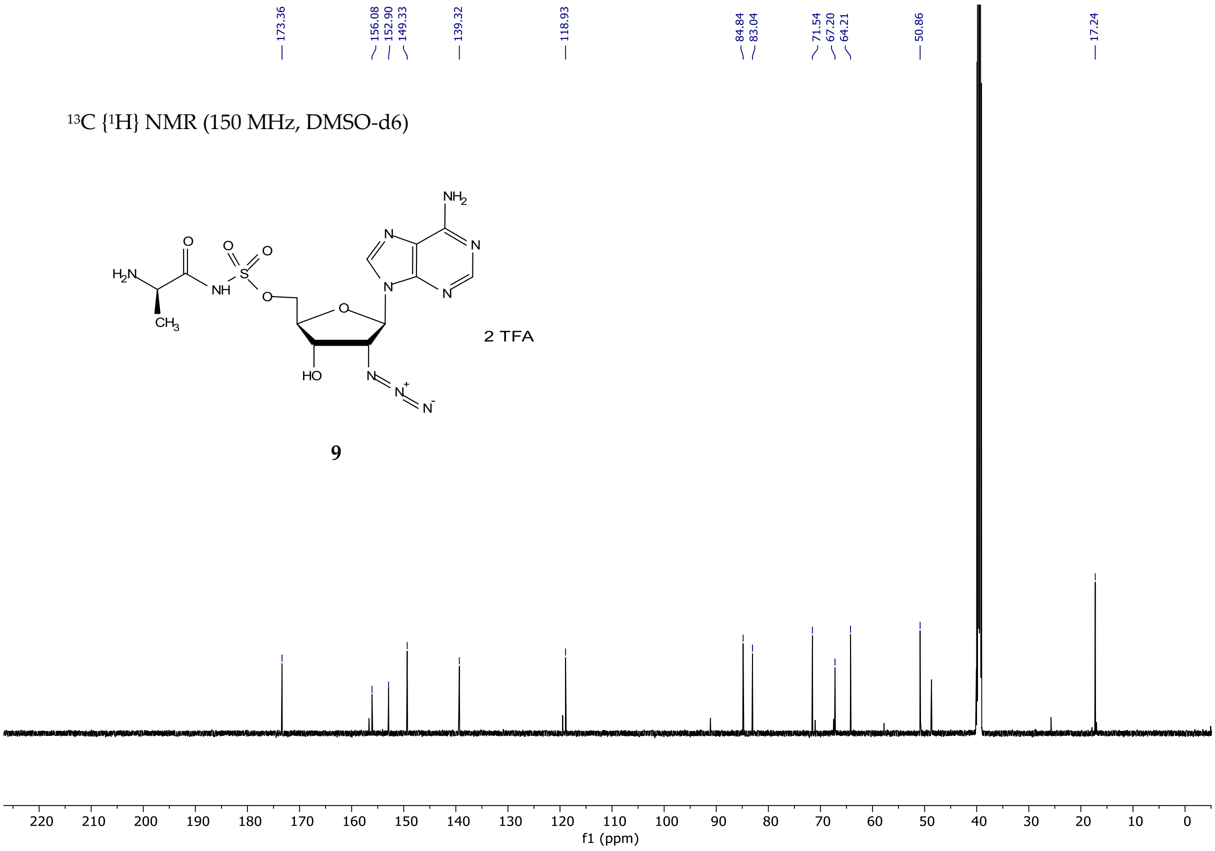

13C {1H} NMR (150 MHz, DMSO-d6)
173.36
156.08
152.90
149.33
139.32
118.93
84.84
83.04
71.54
67.20
64.21
50.86
17.24
NH2
O
H2N
NH
S
CH3
N
HO
2 TFA
9
f1 (ppm)

PDF Complete
Your complimentary use period has ended. Thank you for using PDF Complete.
Click Here to upgrade to Unlimited Pages and Expanded Features



INFORMATION

| | | | |
|---|---|---|---|
| Sample Name: | DL615A | Acquired By: | System |
| Sample Type: | Unknown | Sample Set Name: | 2 |
| Vial: | 2 | Acq. Method Set: | Pureté HPLC |
| Injection #: | 1 | Processing Method: | 2 |
| Injection Volume: | 10,00 ul | Channel Name: | 254,0nm |
| Run Time: | 30,0 Minutes | Proc. Chnl. Descr.: | PDA 254,0 nm |
| Date Acquired: | 08/02/2023 11:54:20 CET | | |
| Date Processed: | 08/02/2023 12:30:30 CET | | |

Spectrum Index Plot

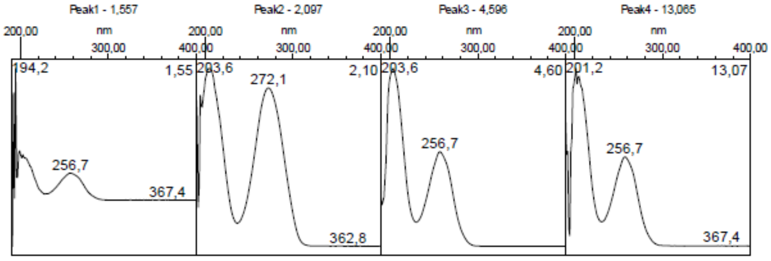


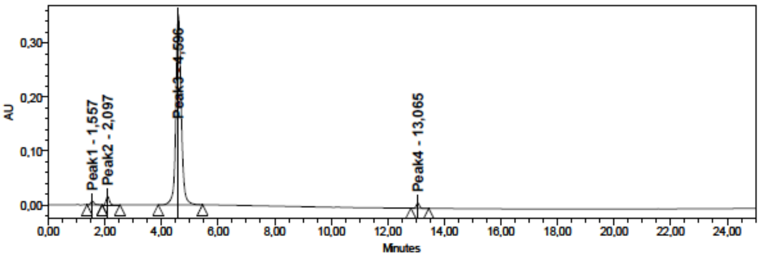


SampleName: DL615A; Vial: 2; Injection: 1; Date Acquired: 08/02/2023 11:54:20 CET

Peak Results

| | Name | RT | Area | % Area |
|---|---|---|---|---|
| 1 | Peak1 | 1,557 | 52630 | 1,07 |
| 2 | Peak2 | 2,097 | 124955 | 2,55 |
| 3 | Peak3 | 4,596 | 4646070 | 94,86 |
| 4 | Peak4 | 13,065 | 74309 | 1,52 |

Reported by User: System
Report Method: rapport
Report Method ID 9479
Page: 1 of 1

Project Name: IA-CELL1-AMY2
Date Printed:
08/02/2023
12:31:05 Europe/Paris

HPLC analysis for compound **9**

$^{1}$H NMR (500 MHz, $CD_3CN$)

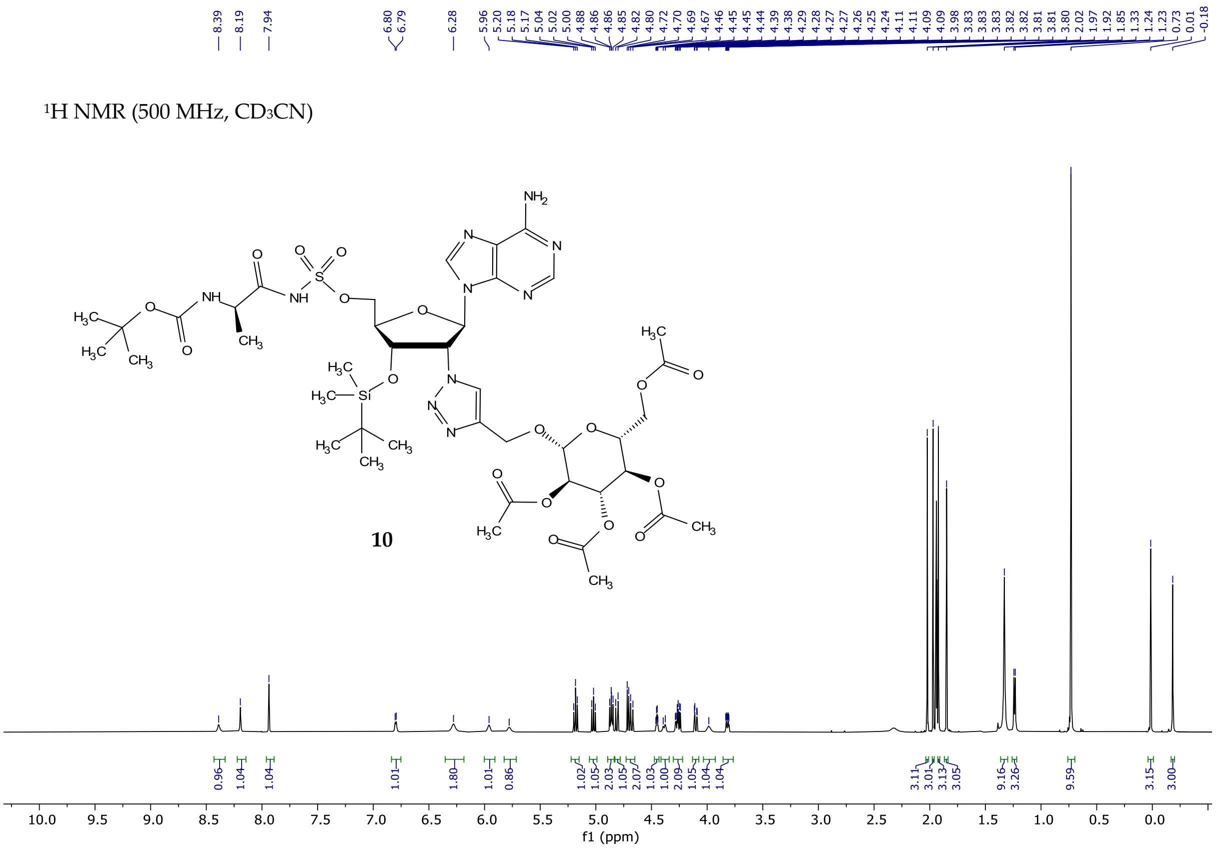


$^{13}$C {$^{1}$H} NMR (150 MHz, $CD_3CN$)

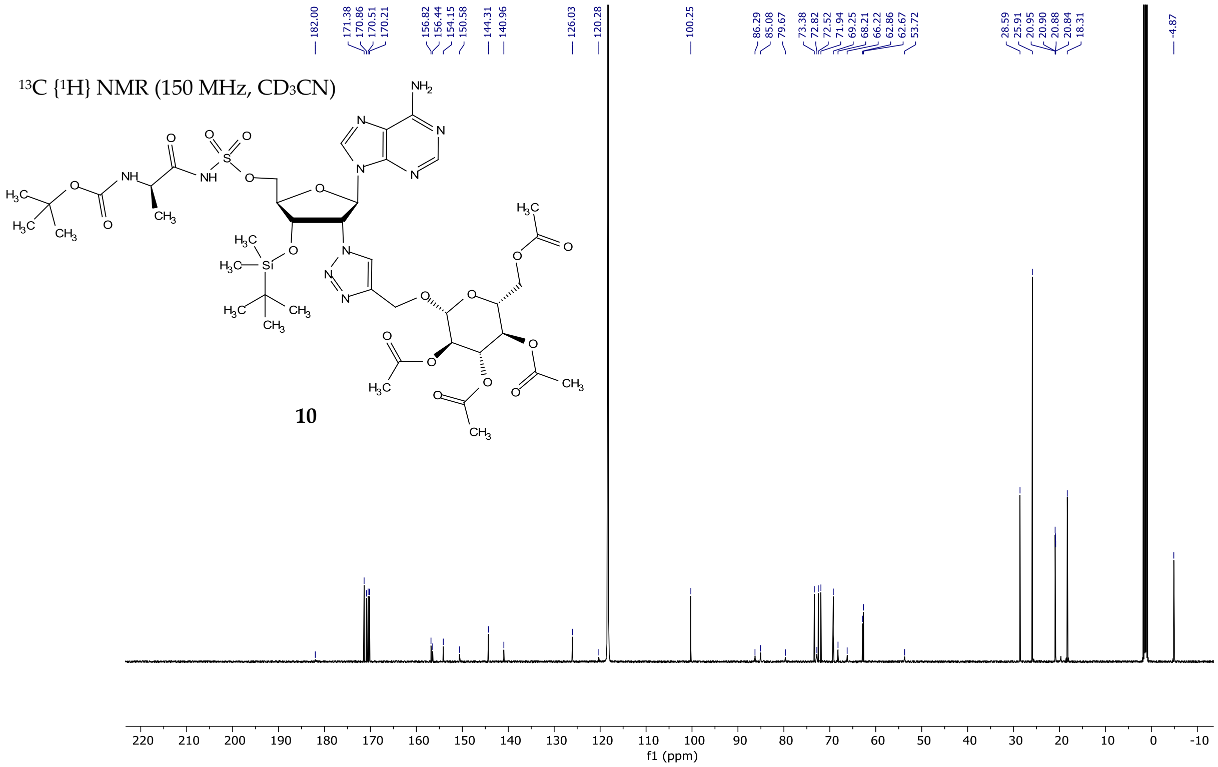

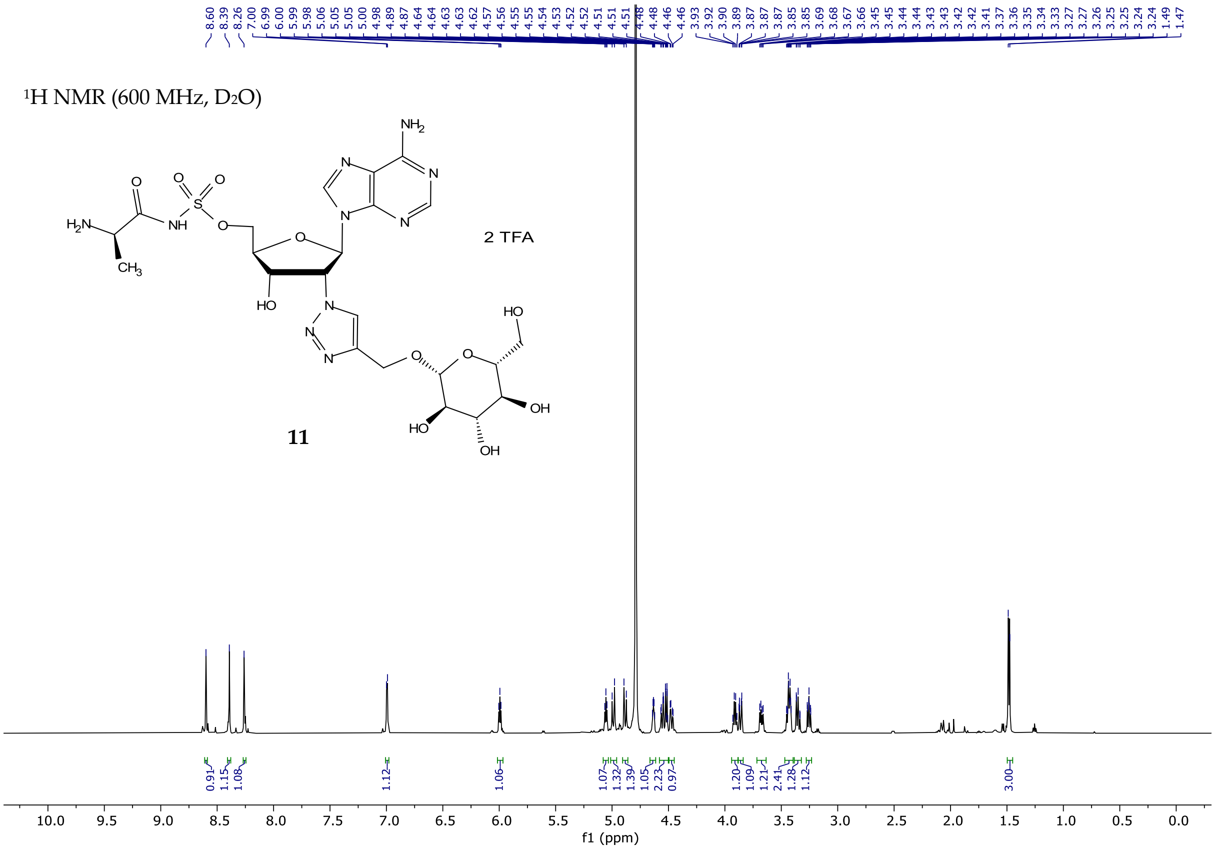
1H NMR (600 MHz, D2O)
8.60 8.39 8.26 7.00 6.99 6.00 5.99 5.98 5.06 5.05 5.05 5.00 4.98 4.89 4.87 4.64 4.64 4.63 4.63 4.62 4.57 4.56 4.55 4.55 4.54 4.53 4.52 4.52 4.51 4.51 4.51 4.48 4.48 4.46 4.46 3.93 3.92 3.90 3.89 3.87 3.87 3.87 3.85 3.85 3.69 3.68 3.67 3.66 3.45 3.45 3.44 3.44 3.43 3.43 3.42 3.42 3.41 3.37 3.36 3.35 3.34 3.33 3.27 3.26 3.25 3.25 3.24 3.24 1.49 1.47
2 TFA
11
0.91 1.15 1.08 1.12 1.06 1.07 1.32 1.39 1.05 2.23 0.97 1.20 1.09 1.21 2.41 1.28 1.12 3.00
f1 (ppm)

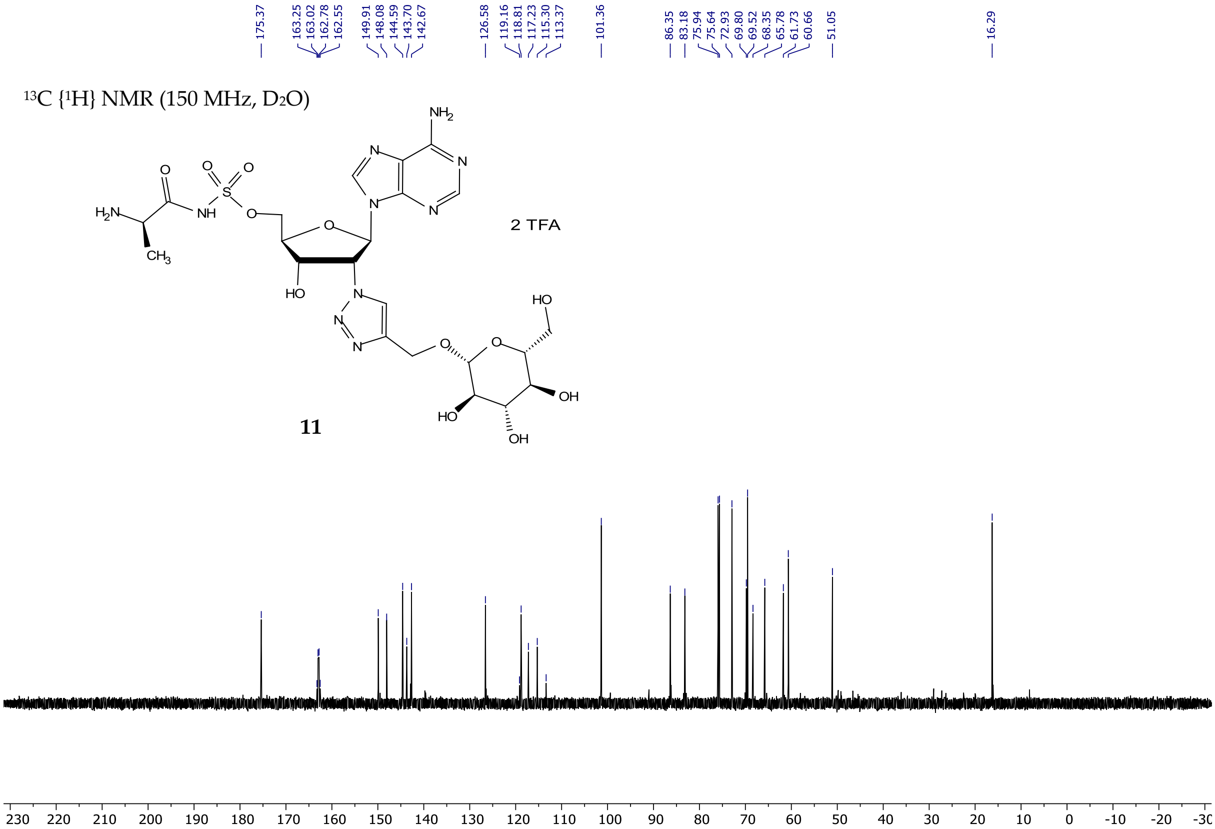
13C {1H} NMR (150 MHz, D2O)
175.37 163.25 163.02 162.78 162.55 149.91 148.08 144.59 143.70 142.67 126.58 119.16 118.81 117.23 115.30 113.37 101.36 86.35 83.18 75.94 75.64 72.93 69.80 69.52 68.35 65.78 61.73 60.66 51.05 16.29
2 TFA
11
f1 (ppm)

PDF Complete
Your complimentary use period has ended. Thank you for using PDF Complete.
Click Here to upgrade to Unlimited Pages and Expanded Features

rapport

INFORMATION

| | | | |
|---|---|---|---|
| Sample Name: | DL627A | Acquired By: | System |
| Sample Type: | Unknown | Sample Set Name: | GF |
| Vial: | 5 | Acq. Method Set: | Pureté HPLC |
| Injection #: | 1 | Processing Method: | 3 |
| Injection Volume: | 10,00 ul | Channel Name: | 254,0nm@4 |
| Run Time: | 30,0 Minutes | Proc. Chnl. Descr.: | PDA 254,0 nm |
| Date Acquired: | 05/09/2023 13:47:52 CET | | |
| Date Processed: | 05/09/2023 14:04:42 CET | | |

**Spectrum Index Plot**

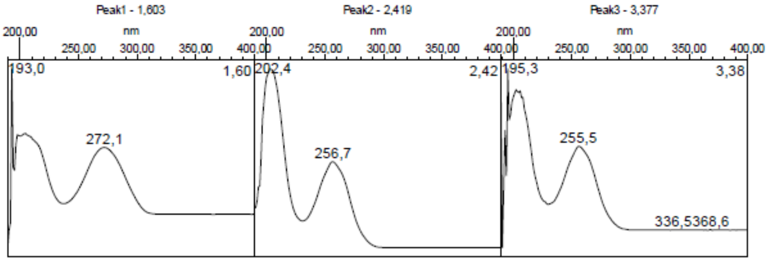


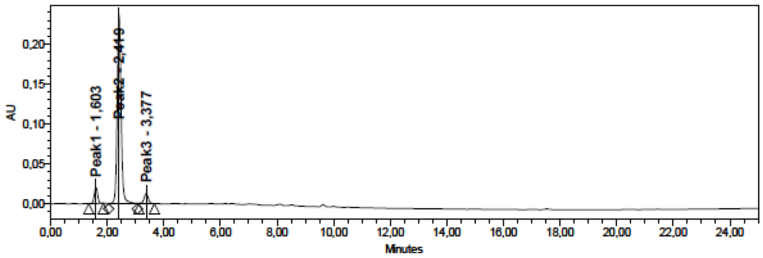


SampleName: DL627A; Vial: 5; Injection: 1; Date Acquired: 05/09/2023 13:47:52 CET

**Peak Results**

| | Name | RT | Area | % Area |
|---|---|---|---|---|
| 1 | Peak1 | 1,603 | 165346 | 6,43 |
| 2 | Peak2 | 2,419 | 2273834 | 88,48 |
| 3 | Peak3 | 3,377 | 130759 | 5,09 |

Reported by User: System
Report Method: rapport
Report Method ID 1001
Page: 1 of 1

Project Name: C18 2023
Date Printed:
27/03/2025
11:29:30 Europe/Paris

HPLC analysis for compound **11**

$^{1}H$ NMR (500 MHz, $CDCl_3$)

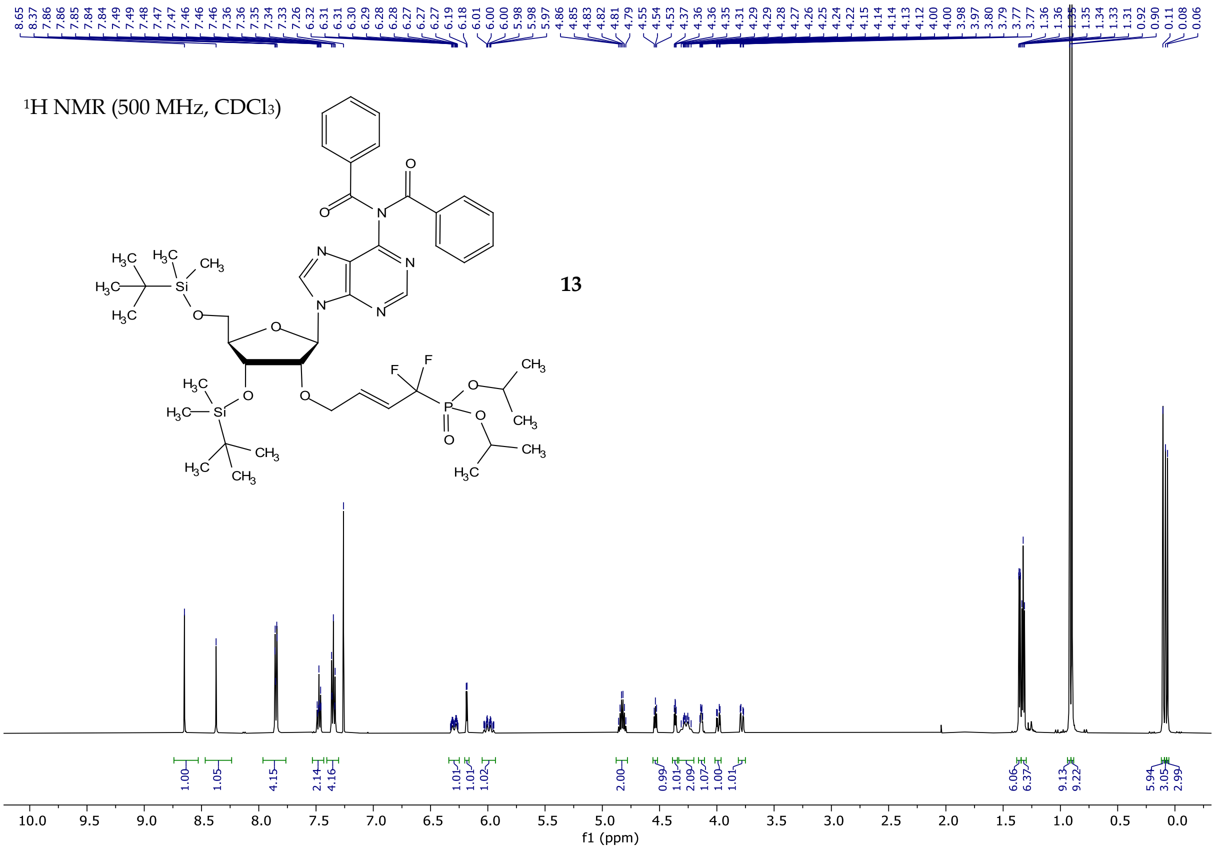


$^{19}F$ NMR (565 MHz, $CDCl_3$)

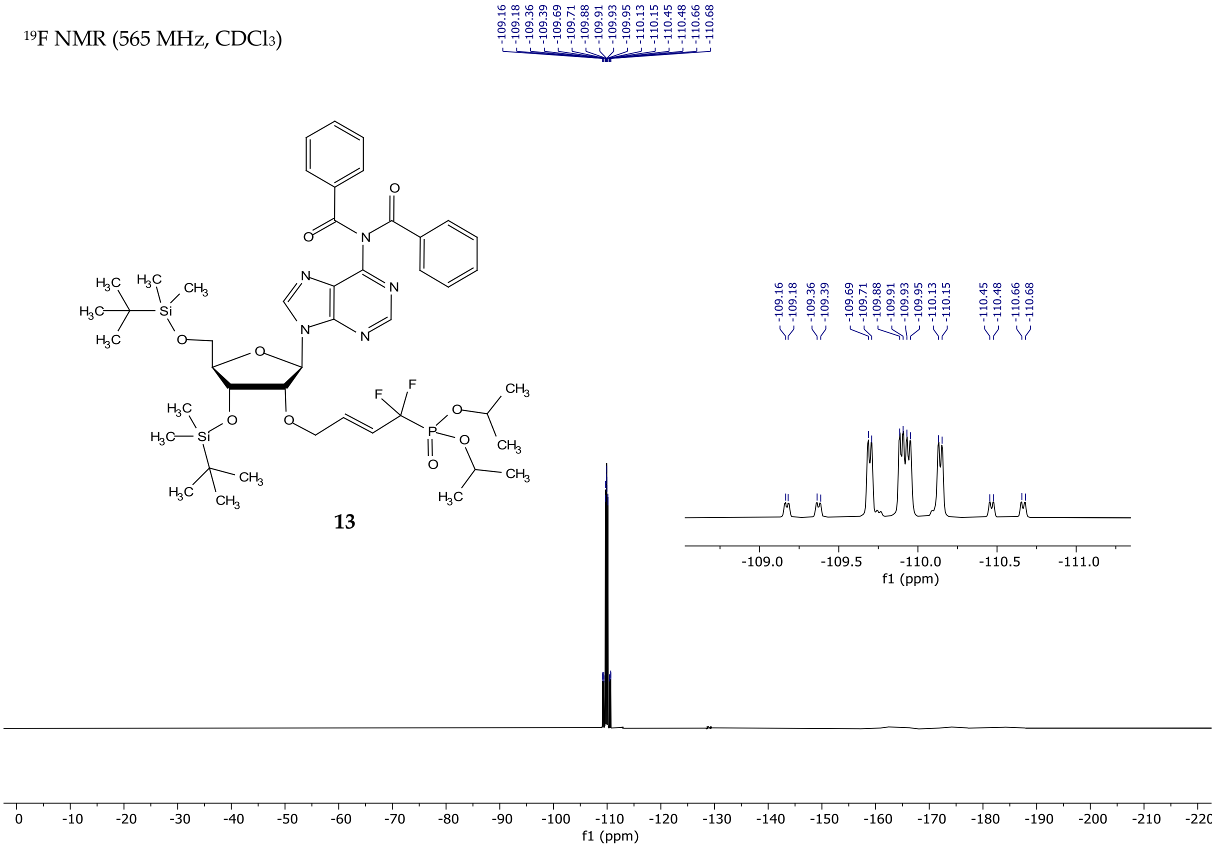

$^{31}P$ NMR (243 MHz, $CDCl_3$)

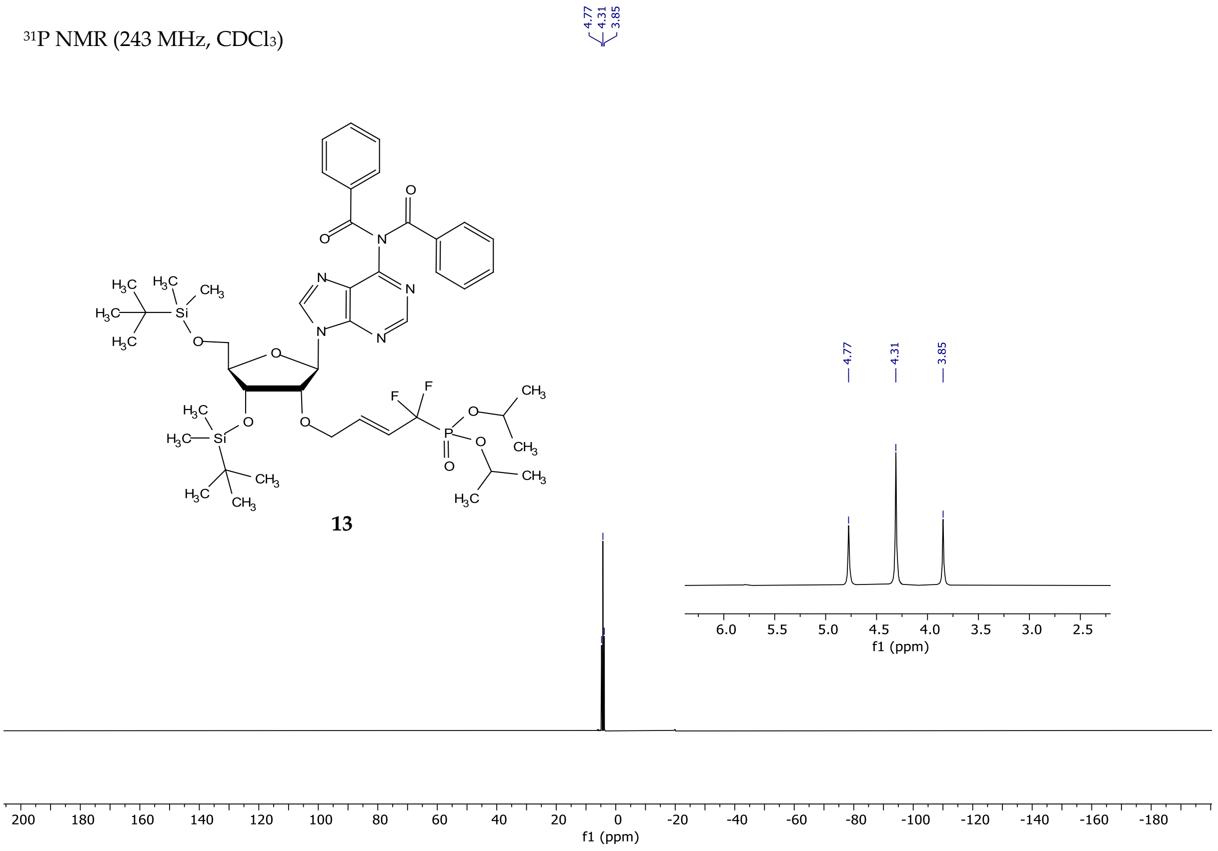


$^{13}C$ {$^1H$} NMR (150 MHz, $CDCl_3$)

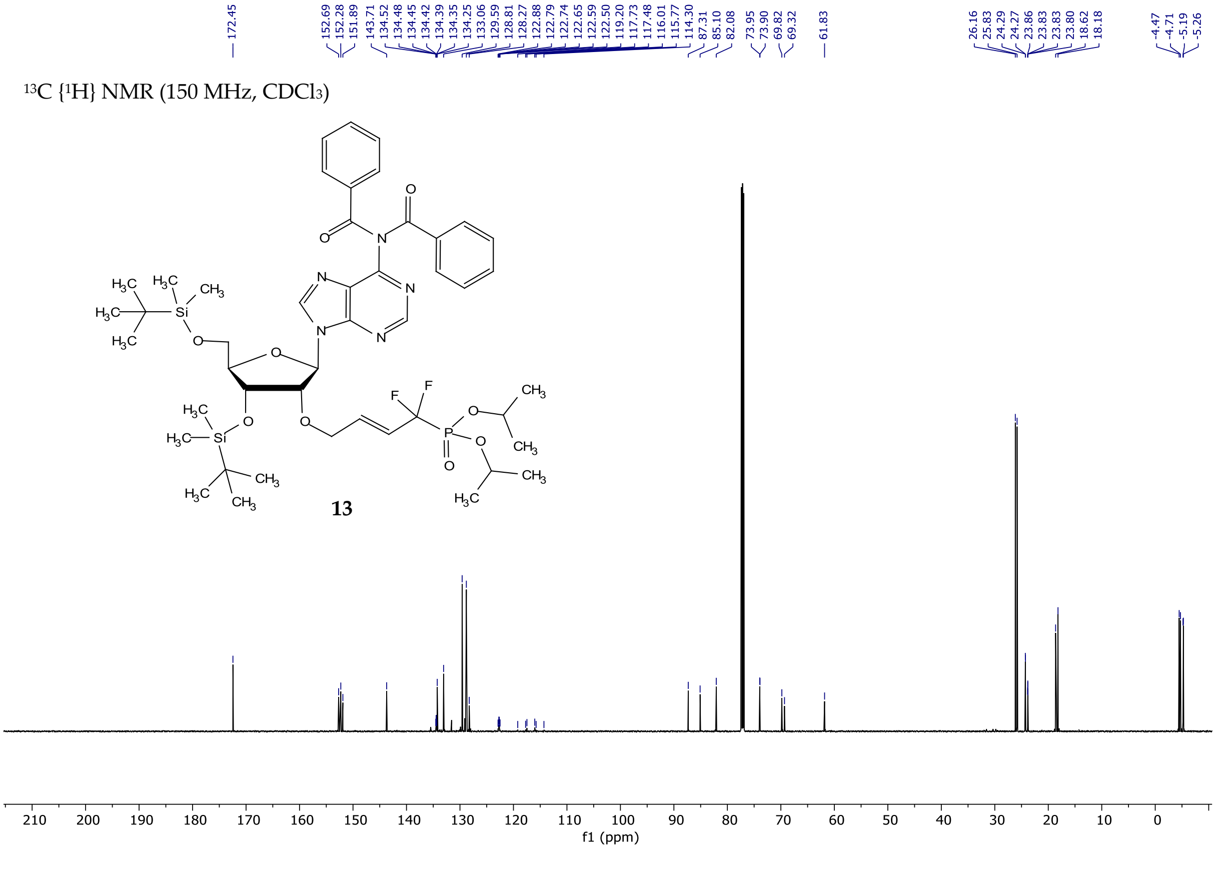

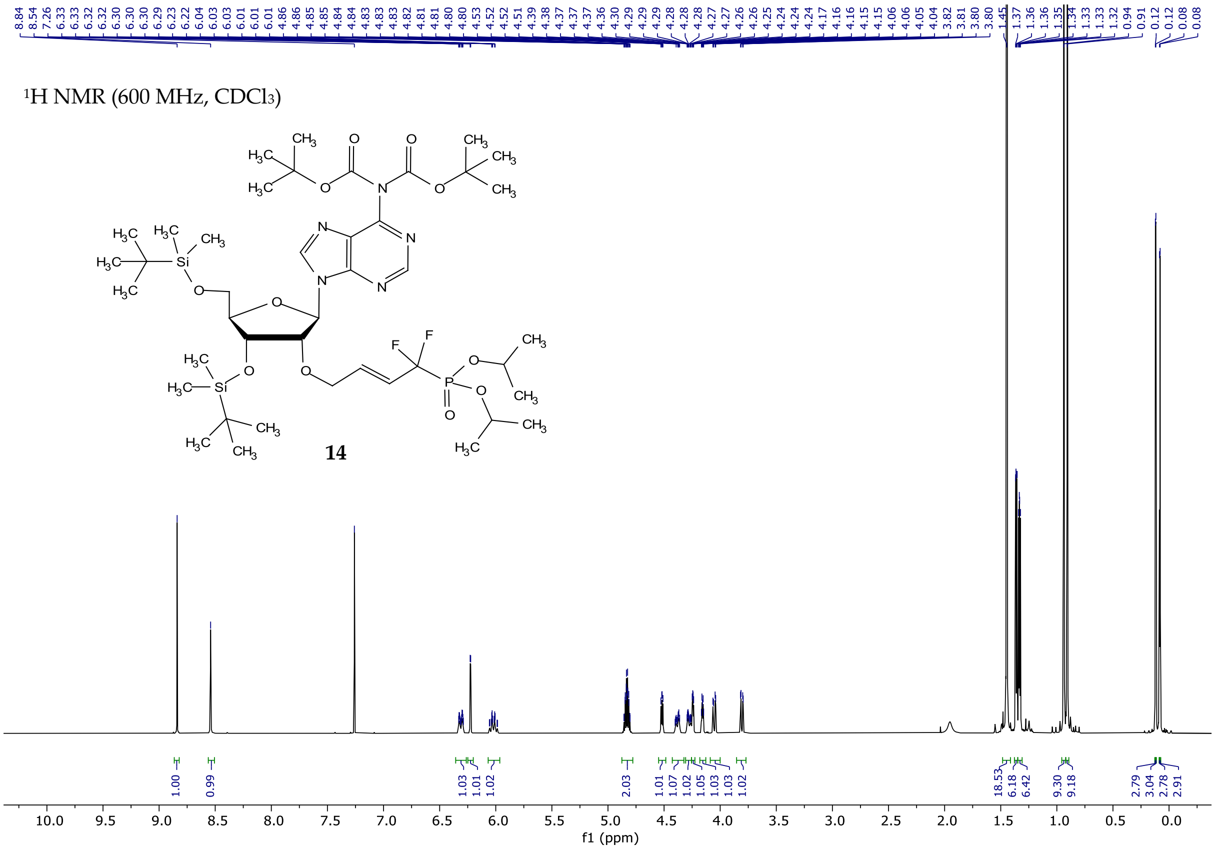
8.84 8.54 7.26 6.33 6.33 6.32 6.32 6.30 6.30 6.30 6.29 6.23 6.22 6.04 6.03 6.03 6.01 6.01 6.01 4.86 4.86 4.85 4.85 4.84 4.84 4.83 4.83 4.83 4.82 4.81 4.81 4.80 4.80 4.53 4.52 4.52 4.51 4.39 4.38 4.37 4.37 4.37 4.36 4.30 4.29 4.29 4.29 4.28 4.28 4.28 4.27 4.27 4.26 4.25 4.24 4.24 4.24 4.17 4.16 4.16 4.15 4.15 4.06 4.05 4.04 3.82 3.81 3.80 3.80 1.45 1.37 1.36 1.36 1.35 1.34 1.33 1.33 1.32 0.94 0.91 0.12 0.12 0.08 0.08
$^1$H NMR (600 MHz, CDCl$_3$)
14
1.00 0.99 1.03 1.01 1.02 2.03 1.01 1.07 1.02 1.05 1.03 1.03 1.02 18.53 6.18 6.42 9.30 9.18 2.79 3.04 2.78 2.91
f1 (ppm)

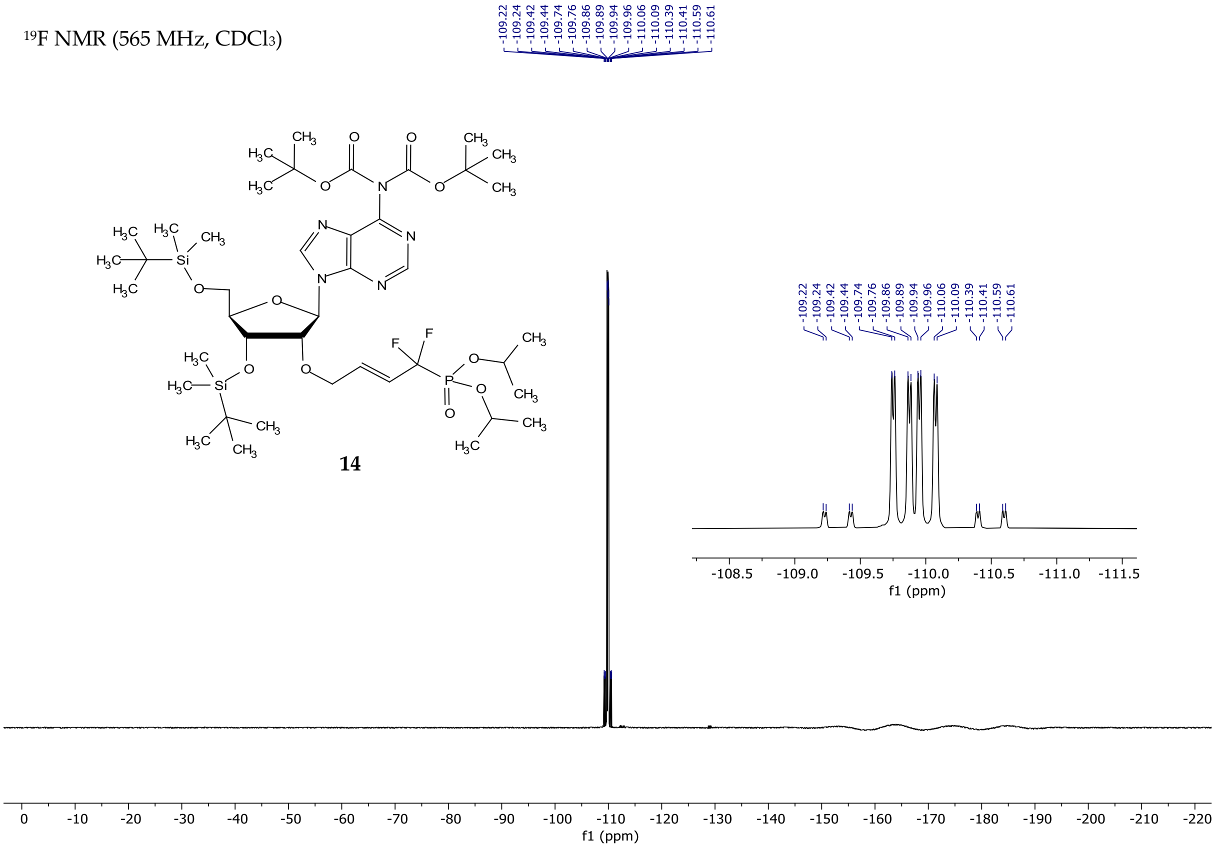
$^{19}$F NMR (565 MHz, CDCl$_3$)
-109.22 -109.24 -109.42 -109.44 -109.74 -109.76 -109.86 -109.89 -109.94 -109.96 -110.06 -110.09 -110.39 -110.41 -110.59 -110.61
14
f1 (ppm)

$^{31}P$ NMR (243 MHz, $CDCl_3$)

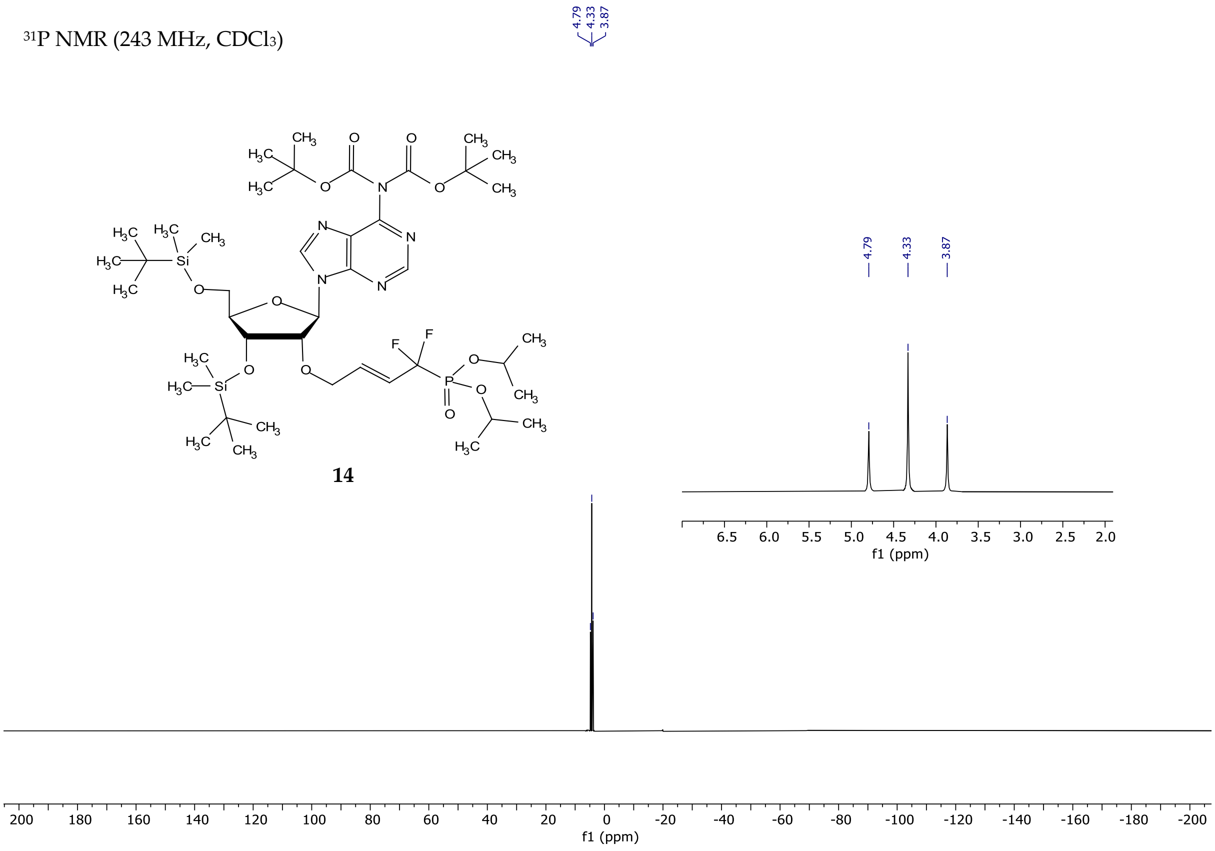


$^{13}C$ {$^{1}H$} NMR (150 MHz, $CDCl_3$)

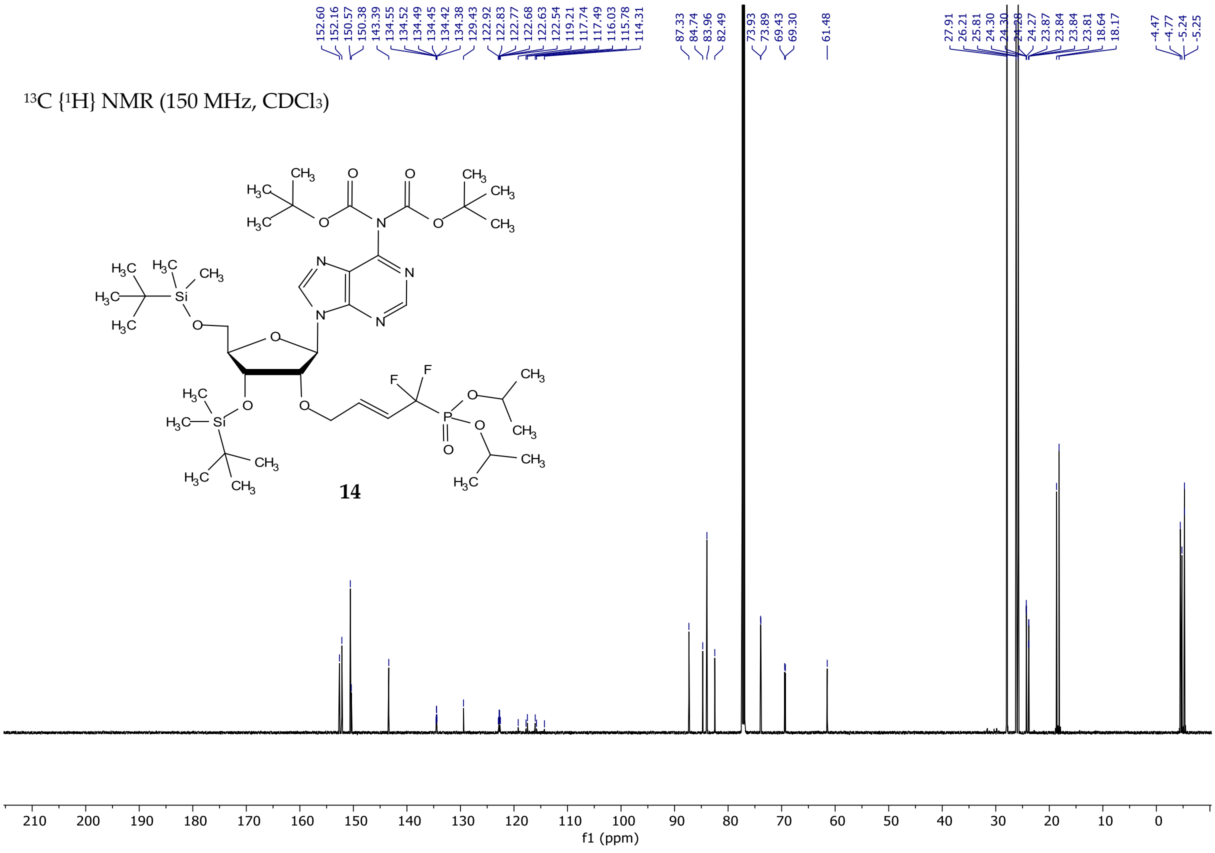

$^1H$ NMR (600 MHz, $CDCl_3$)

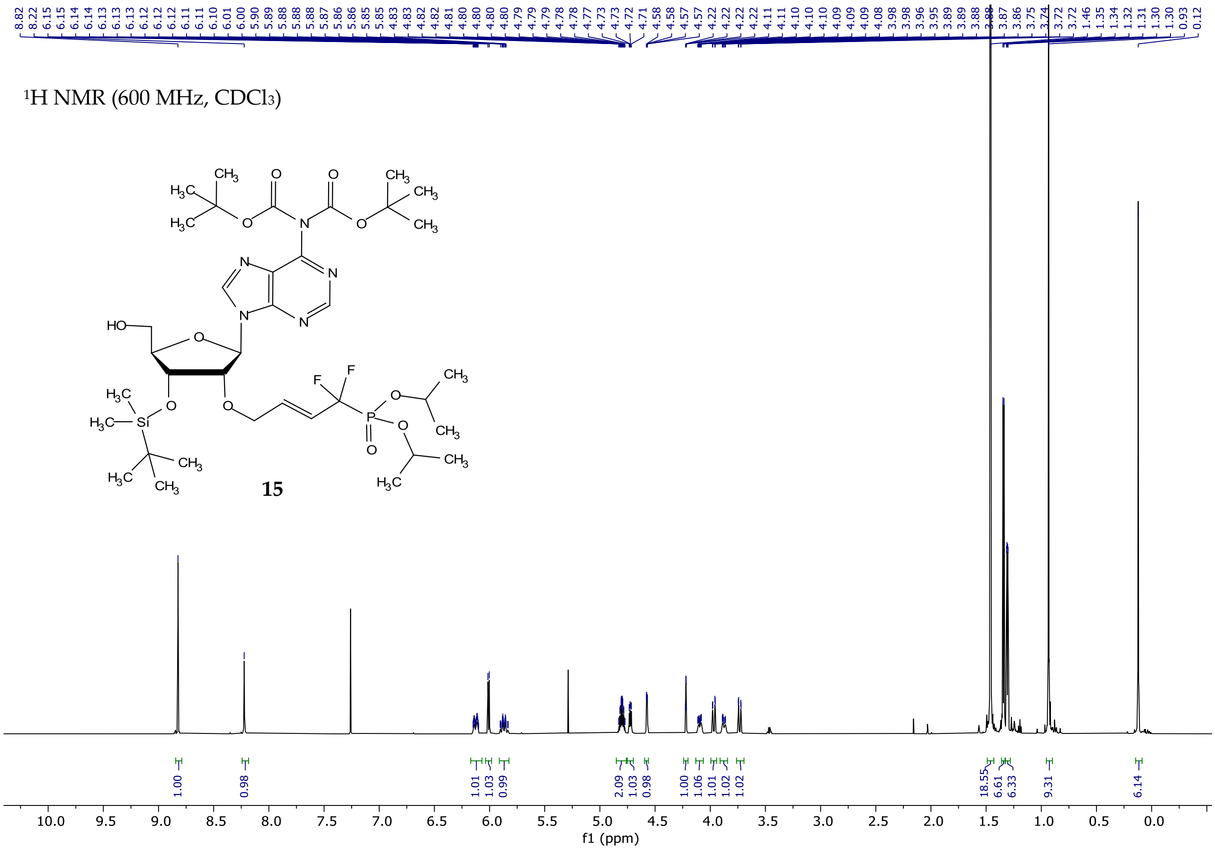


$^{19}F$ NMR (470 MHz, $CDCl_3$)

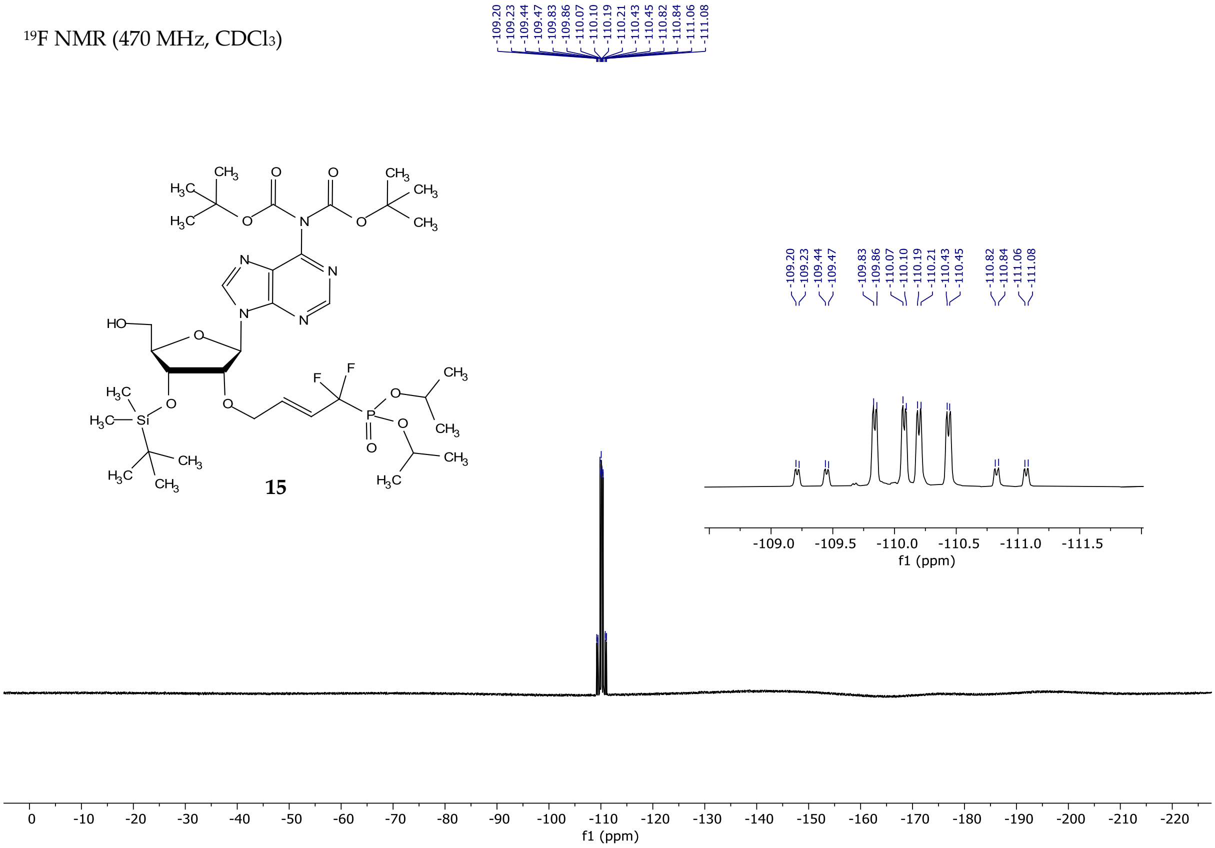

$^{31}P$ NMR (202 MHz, $CDCl_3$)

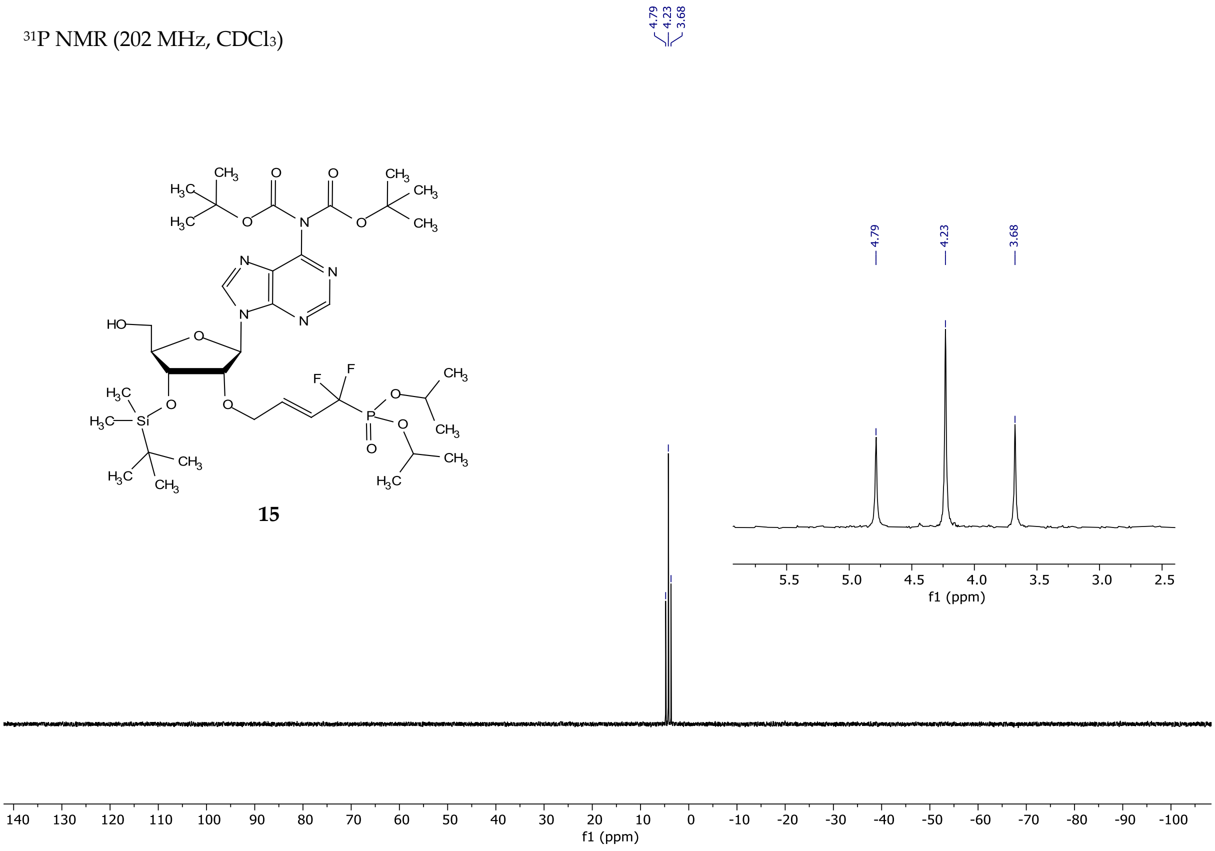


$^{13}C$ {$^{1}H$} NMR (150 MHz, $CDCl_3$)

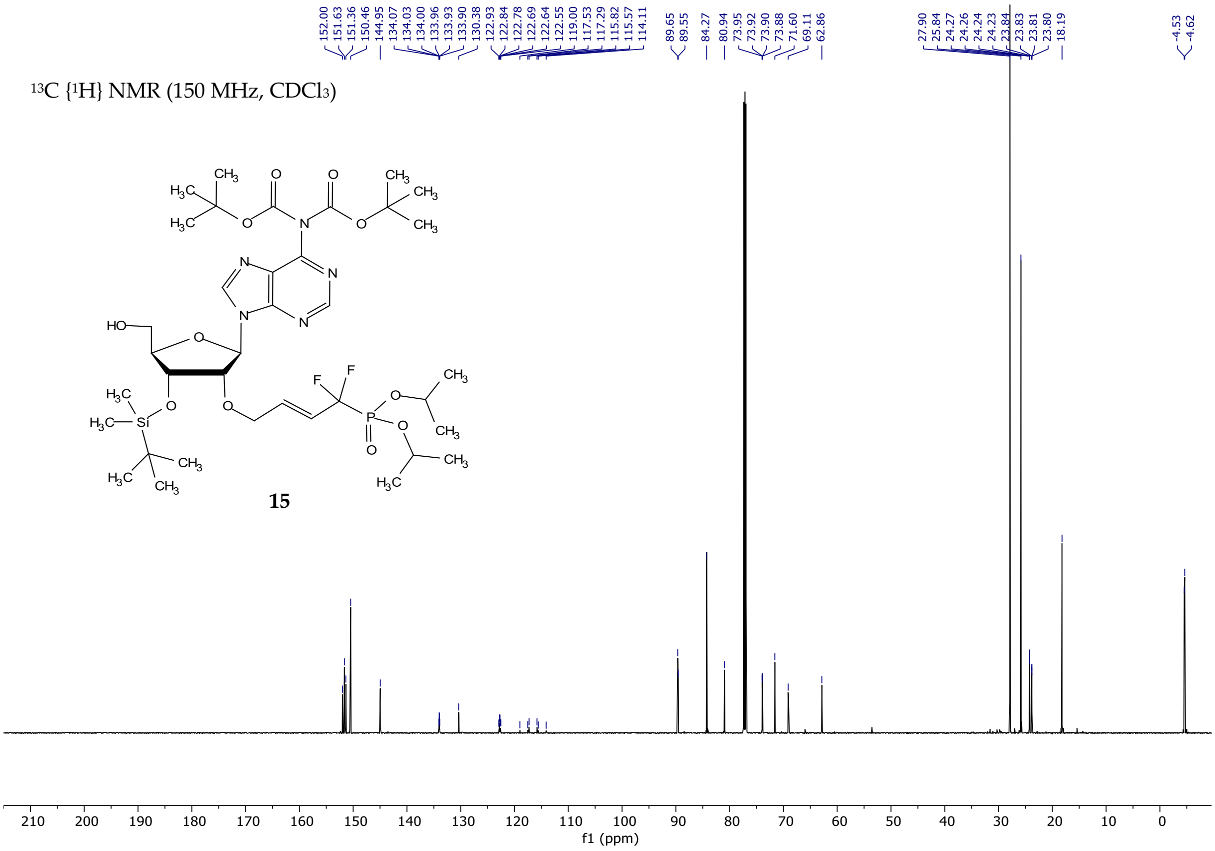

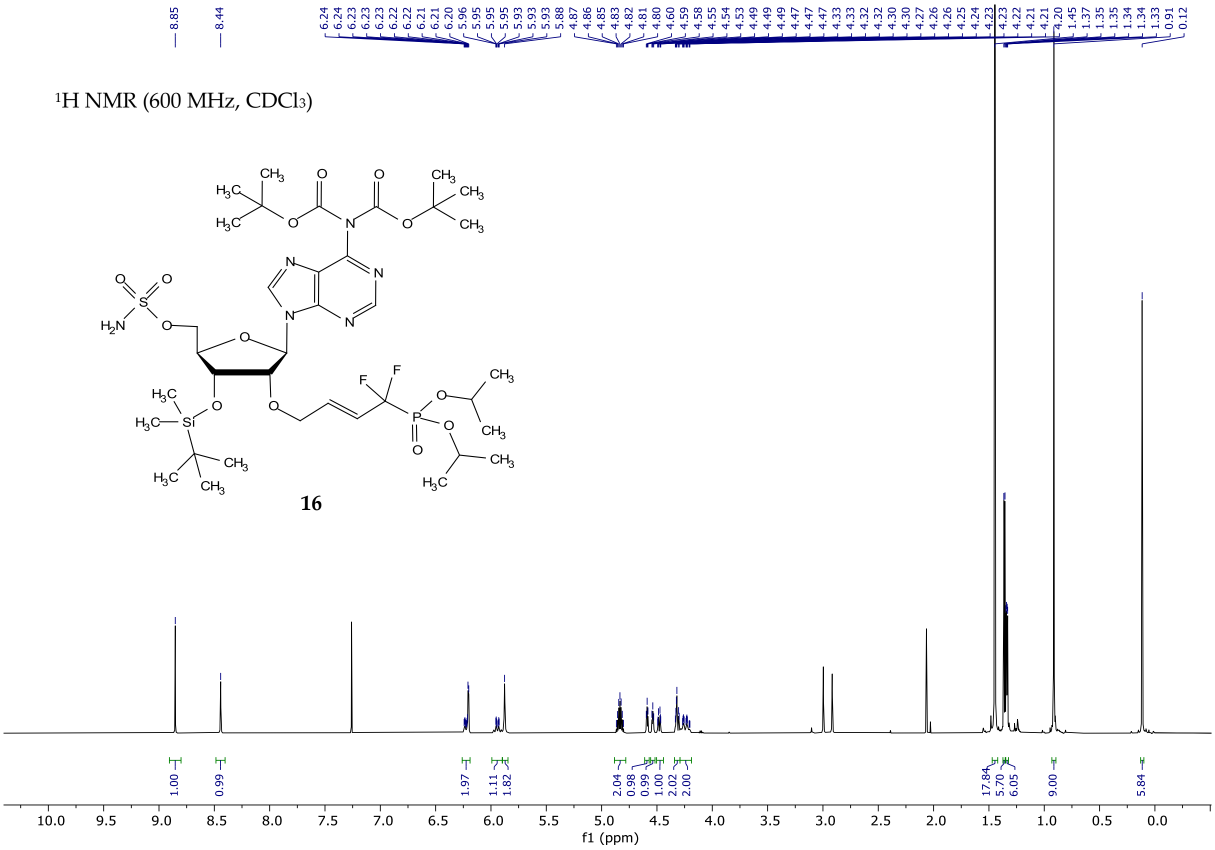
1H NMR (600 MHz, CDCl3)
8.85
8.44
6.24 6.24 6.23 6.23 6.23 6.22 6.22 6.21 6.21 6.20 5.96 5.95 5.95 5.95 5.93 5.93 5.93 5.88 4.87 4.86 4.85 4.83 4.82 4.81 4.80 4.60 4.59 4.58 4.55 4.54 4.53 4.49 4.49 4.49 4.47 4.47 4.47 4.33 4.33 4.32 4.32 4.30 4.30 4.27 4.26 4.26 4.25 4.24 4.23 4.23 4.22 4.21 4.21 4.20 1.45 1.37 1.35 1.35 1.34 1.33 0.91 0.12
16
1.00 0.99 1.97 1.11 1.82 2.04 0.98 0.99 1.00 2.02 2.00 17.84 5.70 6.05 9.00 5.84
f1 (ppm)

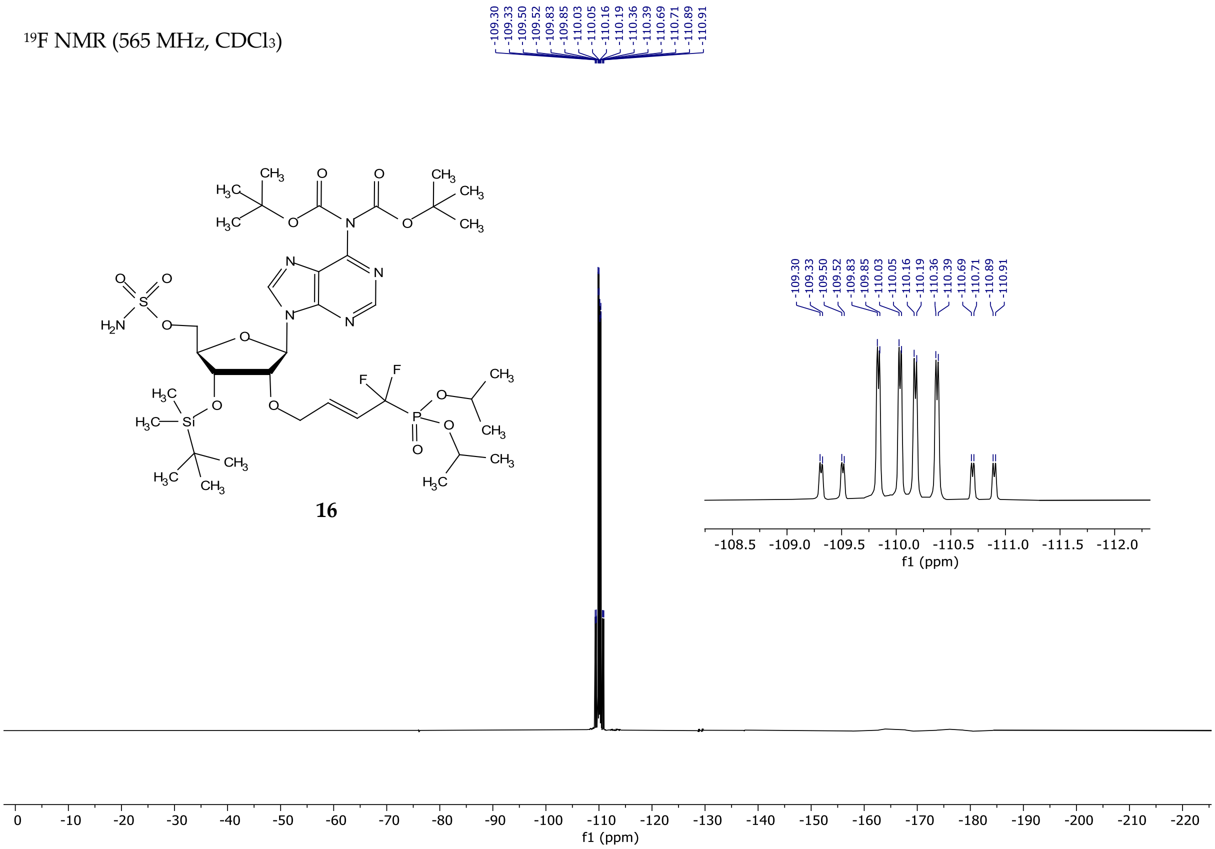
19F NMR (565 MHz, CDCl3)
-109.30 -109.33 -109.50 -109.52 -109.83 -109.85 -110.03 -110.05 -110.16 -110.19 -110.36 -110.39 -110.69 -110.71 -110.89 -110.91
16
f1 (ppm)

$^{31}P$ NMR (243 MHz, $CDCl_3$)

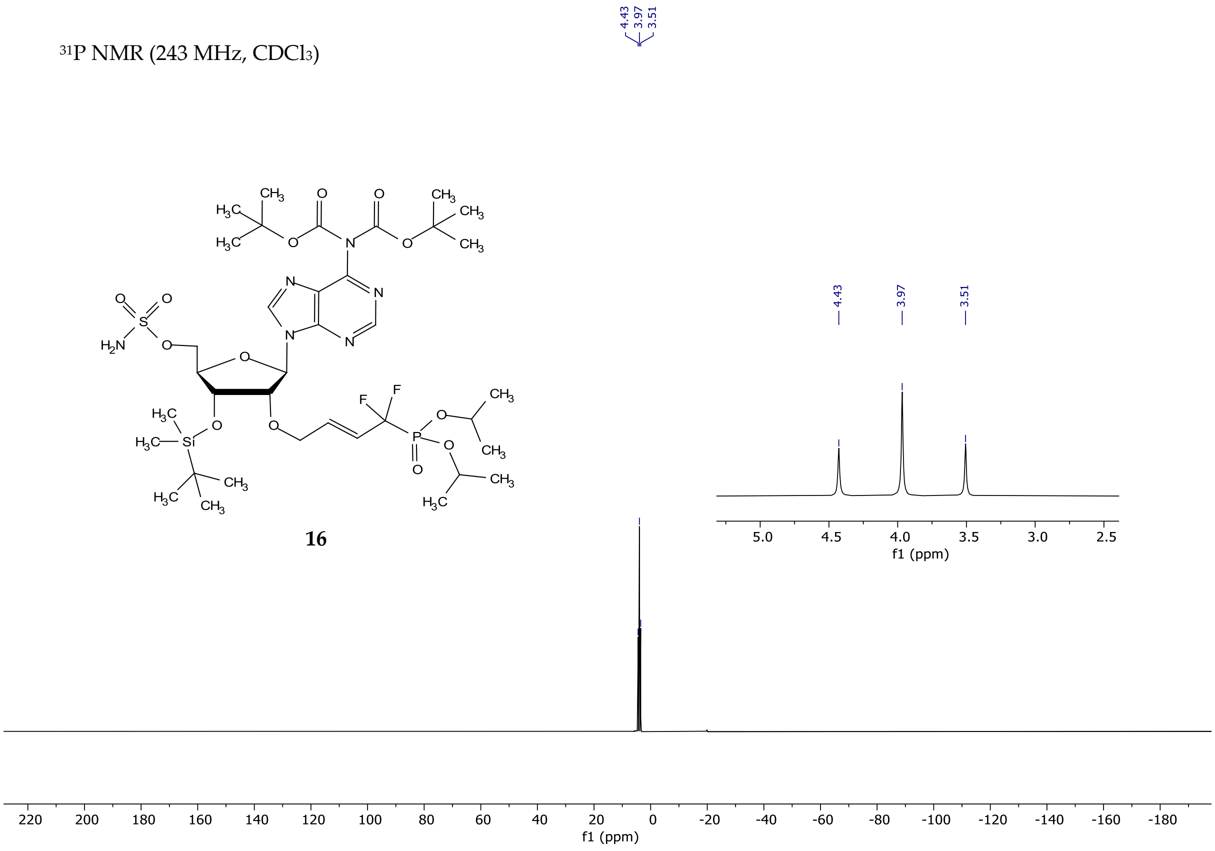


**16**

$^{13}C$ {$^{1}H$} NMR (150 MHz, $CDCl_3$)

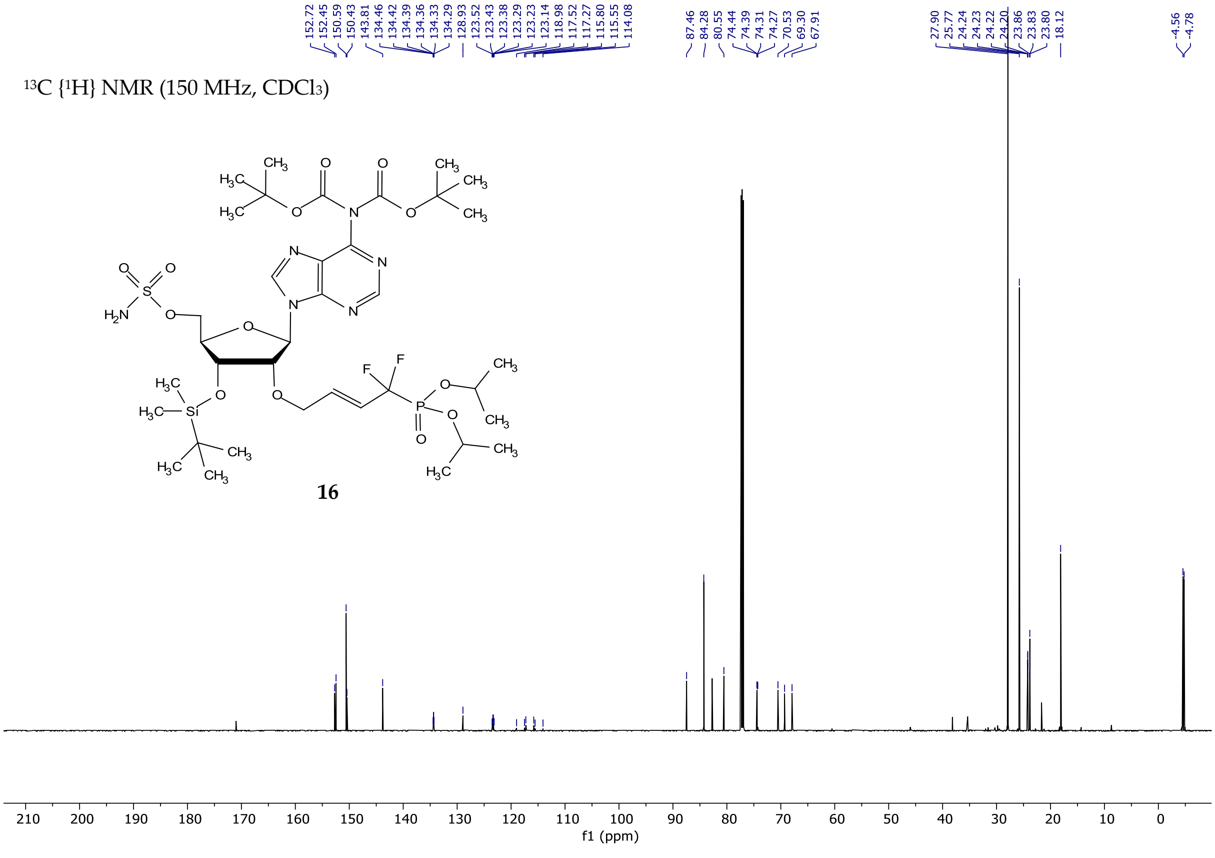


**16**

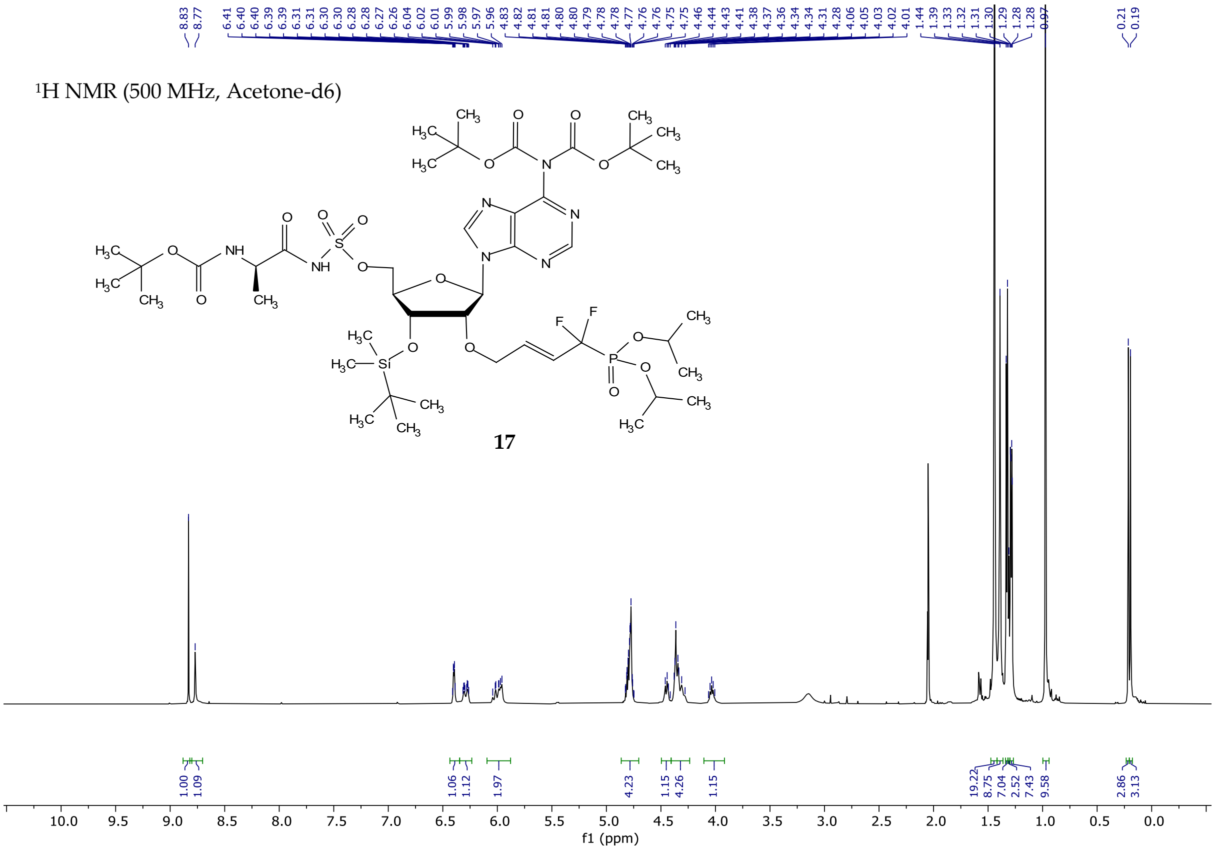
$^{1}$H NMR (500 MHz, Acetone-d6)
17
f1 (ppm)

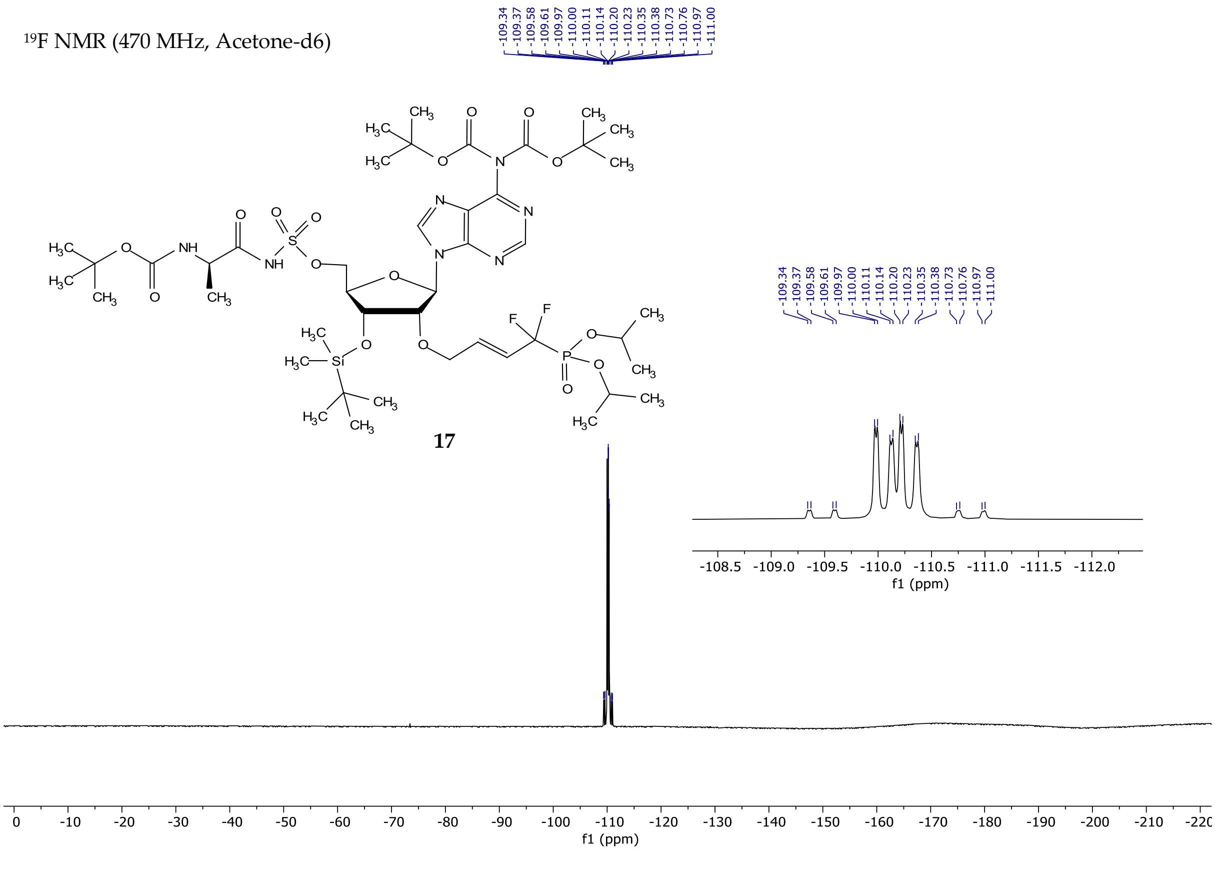
$^{19}$F NMR (470 MHz, Acetone-d6)
17
f1 (ppm)

$^{31}P$ NMR (202 MHz, Acetone-d6)

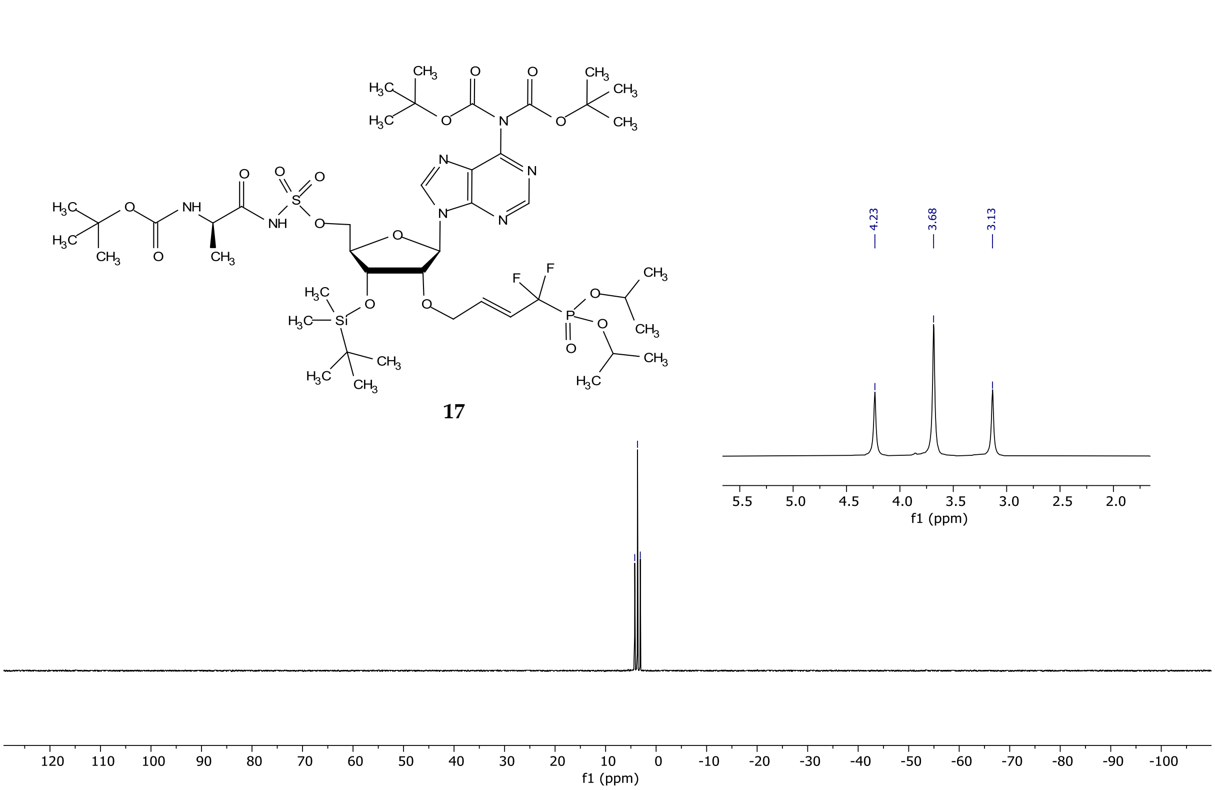


$^{13}C$ {$^{1}H$} NMR (150 MHz, Acetone-d6)

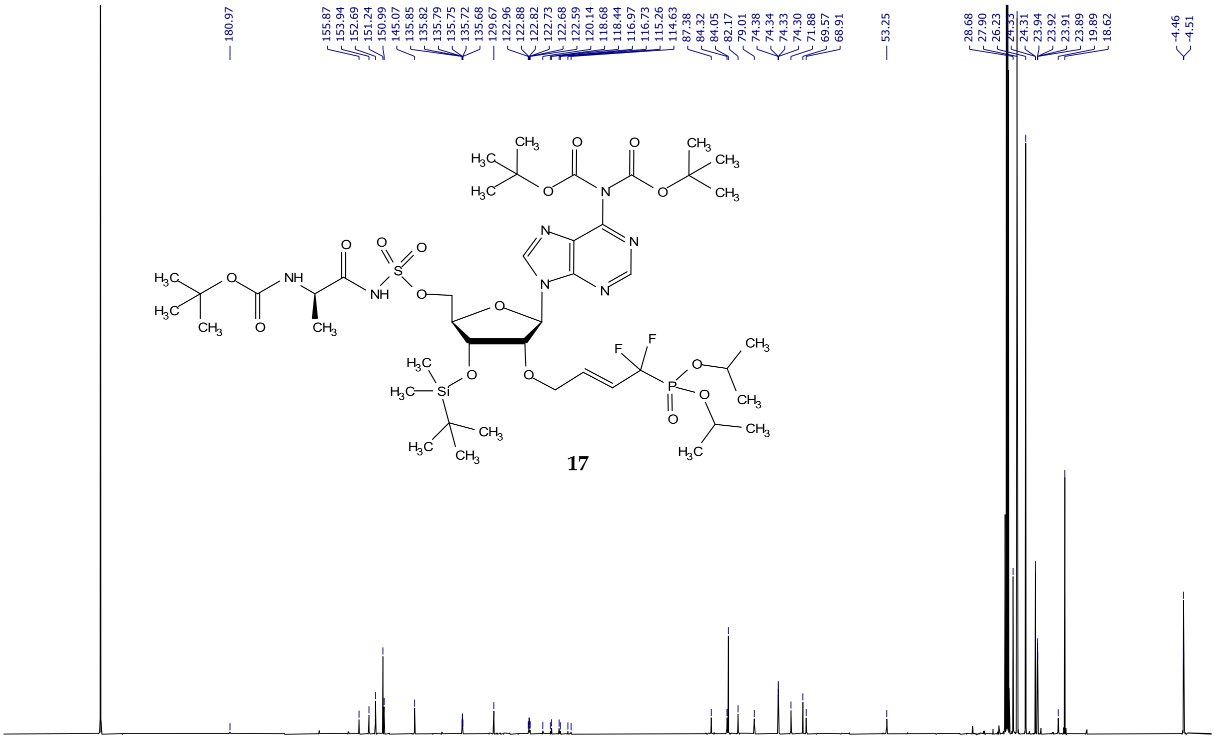

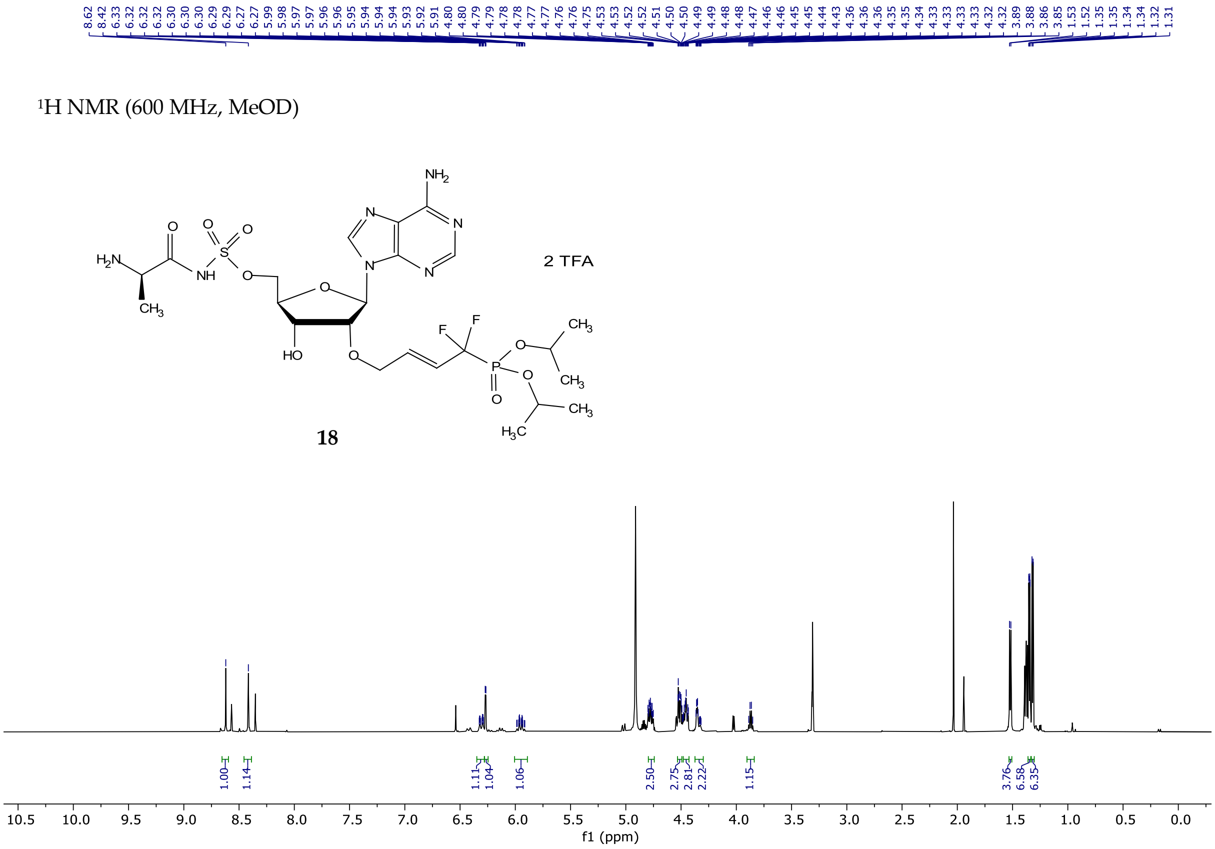
1H NMR (600 MHz, MeOD)
2 TFA
18
f1 (ppm)

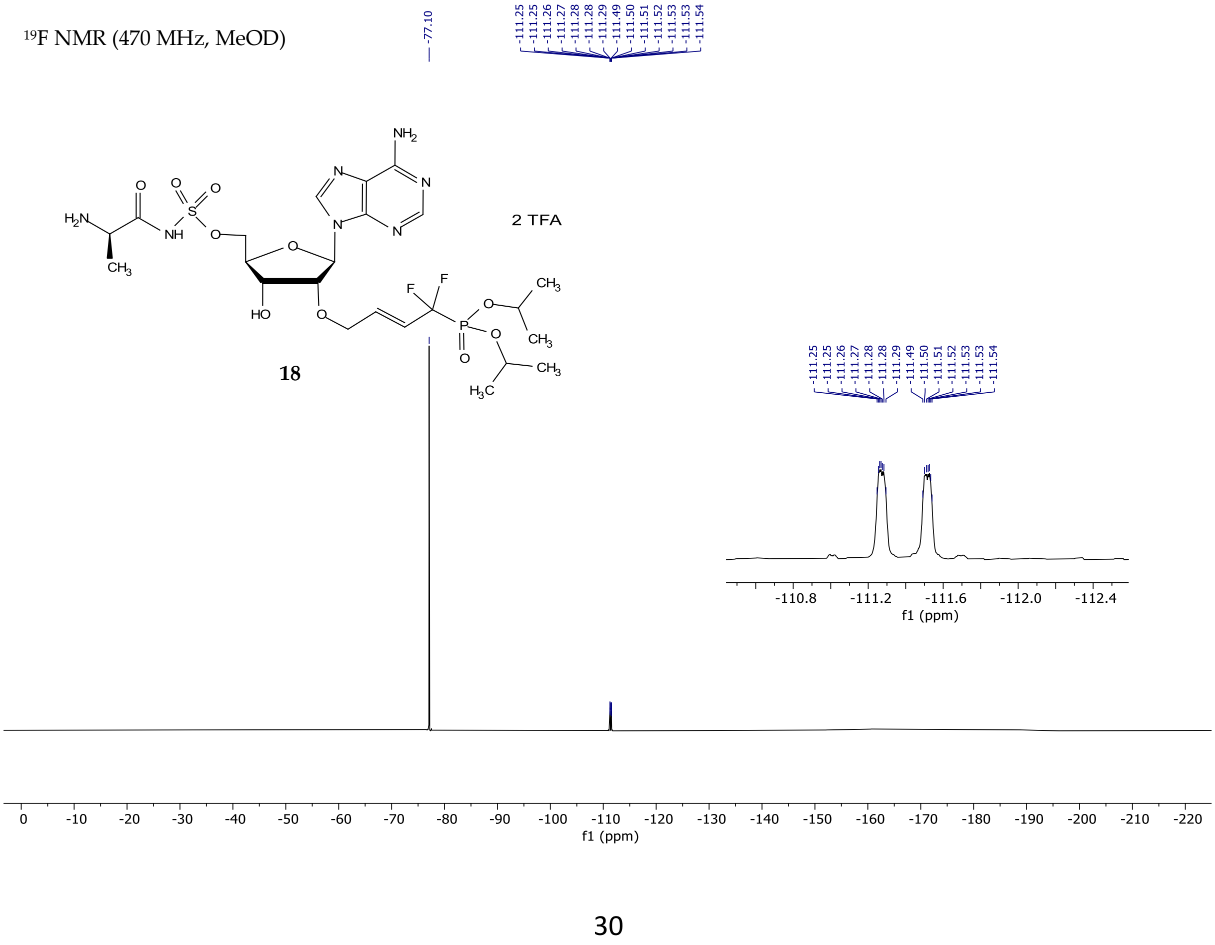
19F NMR (470 MHz, MeOD)
-77.10
2 TFA
18
f1 (ppm)

$^{31}P$ NMR (202 MHz, MeOD)

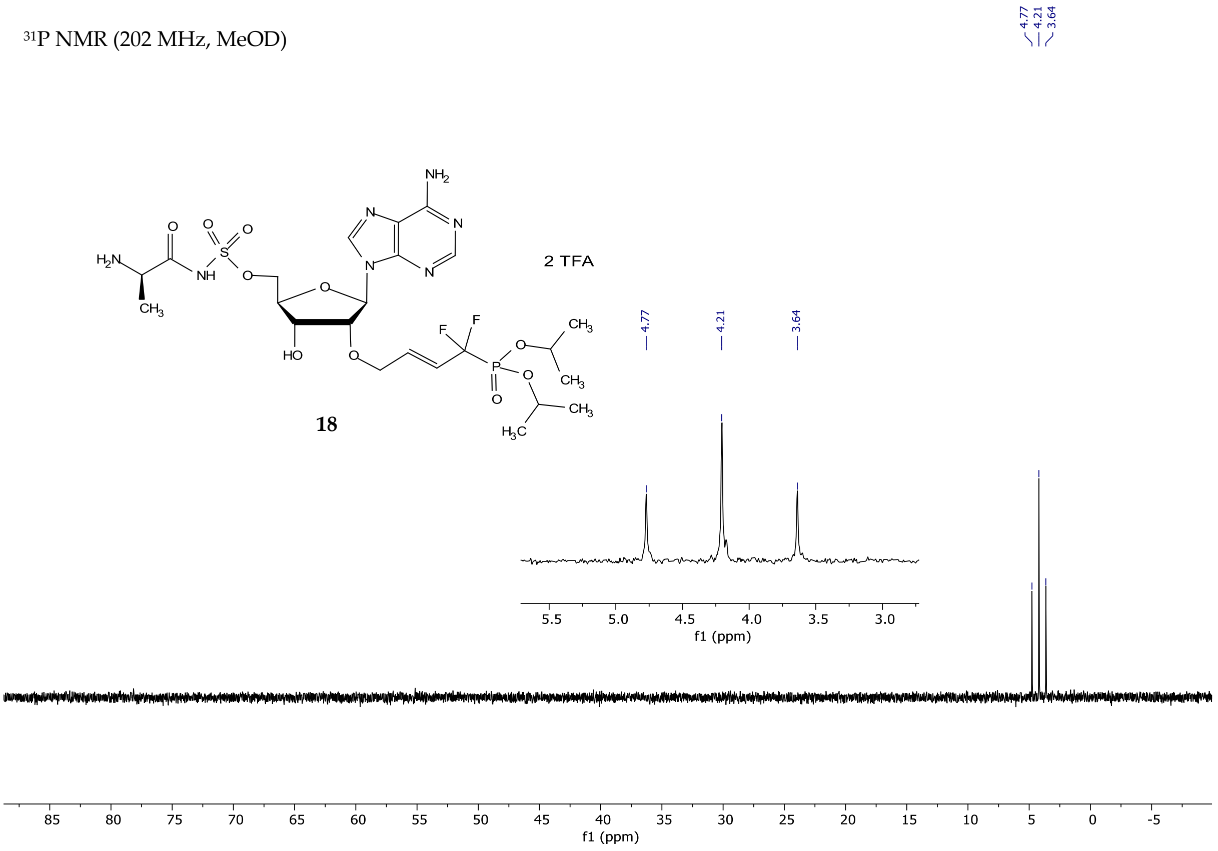


$^{13}C$ {$^{1}H$} NMR (150 MHz, MeOD)

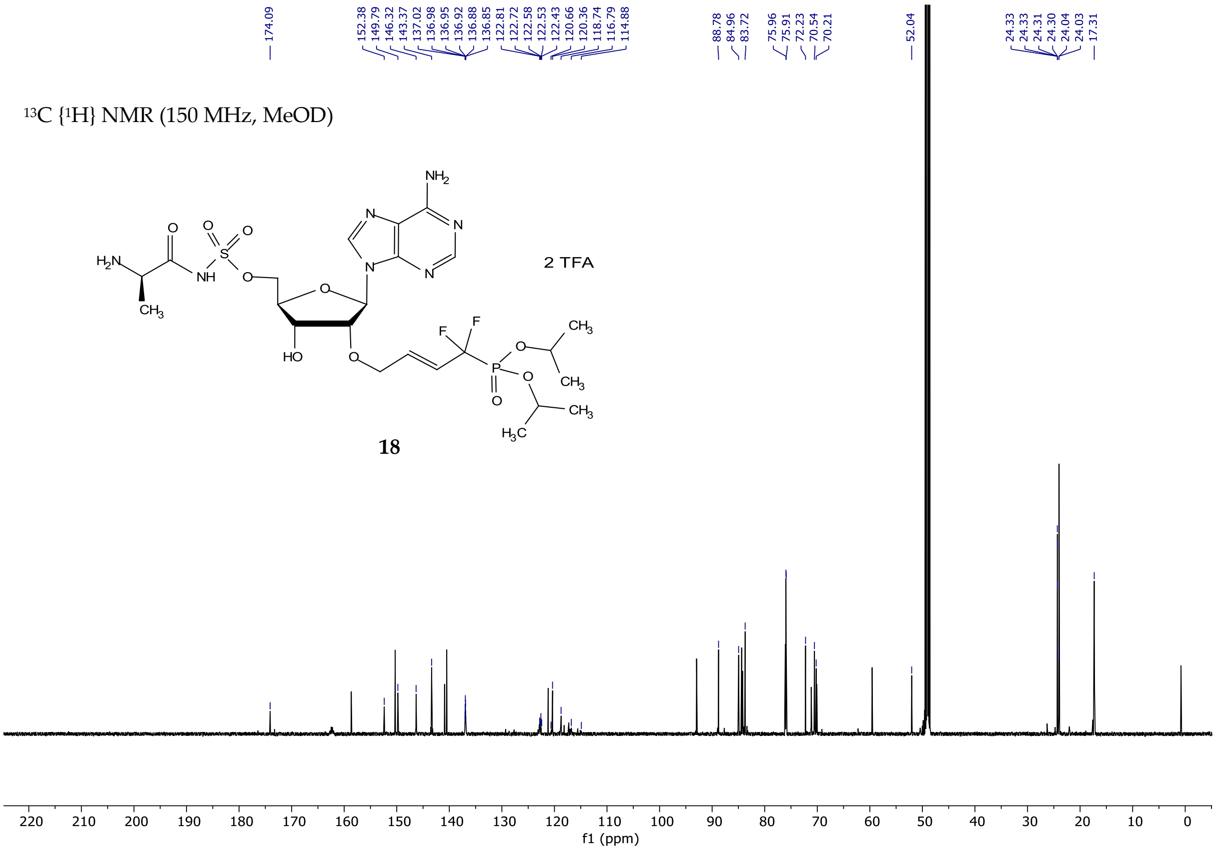

PDF Complete
Your complimentary use period has ended. Thank you for using PDF Complete.
Click Here to upgrade to Unlimited Pages and Expanded Features

rapport

INFORMATION

| | | | |
|---|---|---|---|
| Sample Name: | DL527 PURIFIE | Acquired By: | System |
| Sample Type: | Unknown | Sample Set Name: | 1 |
| Vial: | 36 | Acq. Method Set: | Pureté HPLC |
| Injection #: | 1 | Processing Method: | 2 |
| Injection Volume: | 10,00 ul | Channel Name: | 254,0nm |
| Run Time: | 30,0 Minutes | Proc. Chnl. Descr.: | PDA 254,0 nm |
| Date Acquired: | 20/10/2022 09:51:01 CET | | |
| Date Processed: | 20/10/2022 12:44:23 CET | | |

Spectrum Index Plot

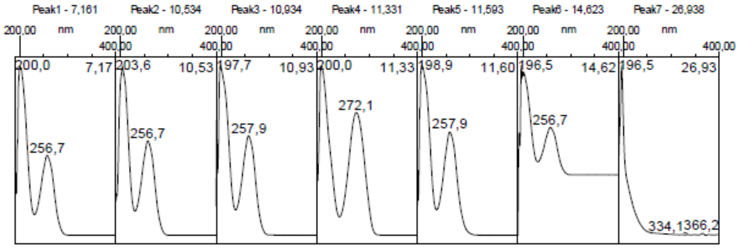


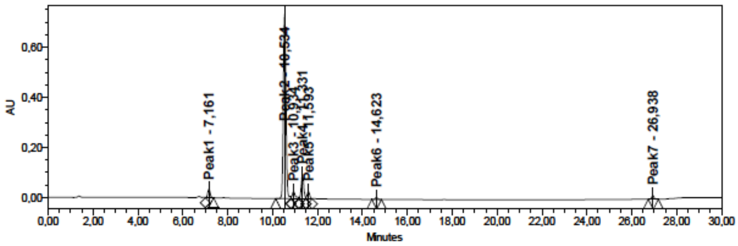


SampleName: DL527 PURIFIE; Vial: 36; Injection: 1; Date Acquired: 20/10/2022 09:51:01 CET

Peak Results

| | Name | RT | Area | % Area |
|---|---|---|---|---|
| 1 | Peak1 | 7,161 | 253212 | 3,38 |
| 2 | Peak2 | 10,534 | 5817058 | 77,55 |
| 3 | Peak3 | 10,934 | 283249 | 3,78 |
| 4 | Peak4 | 11,331 | 765543 | 10,21 |

Reported by User: System
Report Method: rapport
Report Method ID 5622
Page: 1 of 2

Project Name: IA-CELL1-AMY2
Date Printed:
20/10/2022
14:00:21 Europe/Paris

HPLC analysis for compound **18**

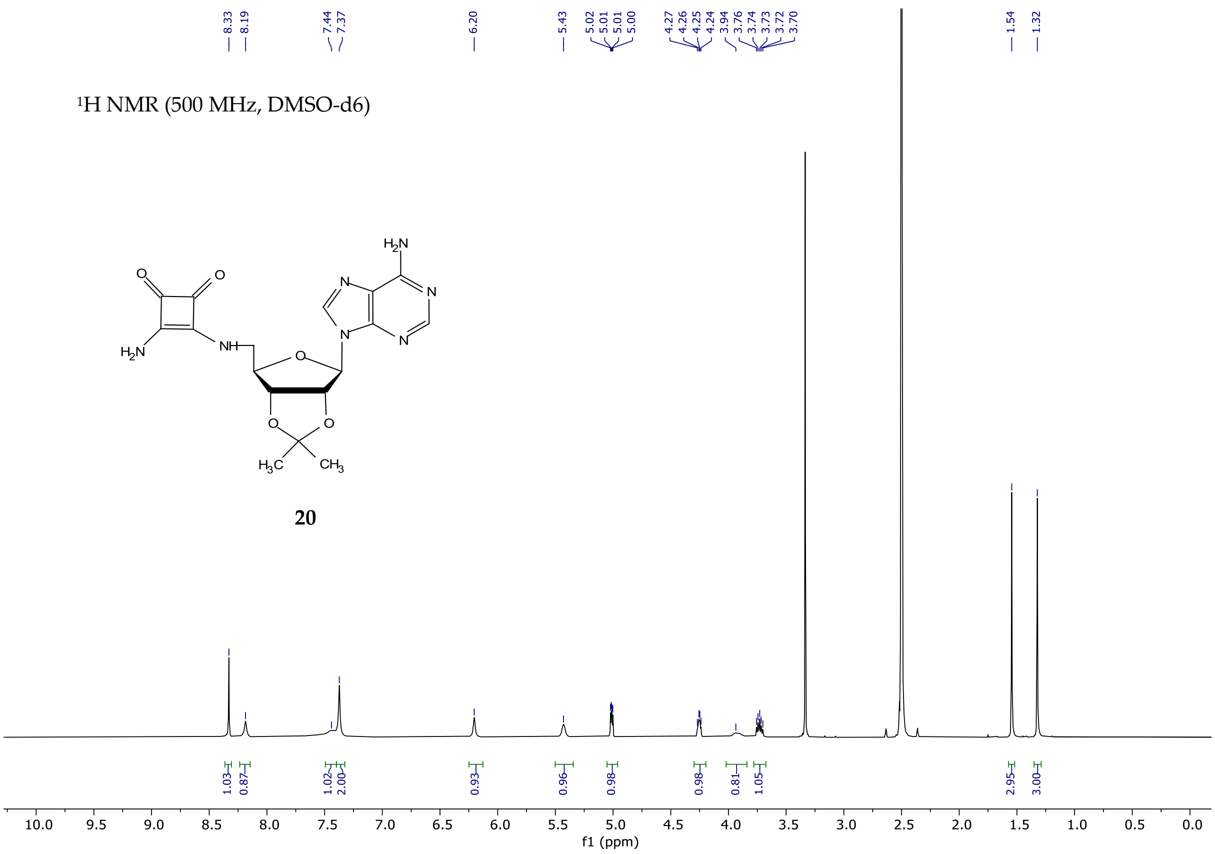

$^{1}$H NMR (500 MHz, DMSO-d6)
20
f1 (ppm)


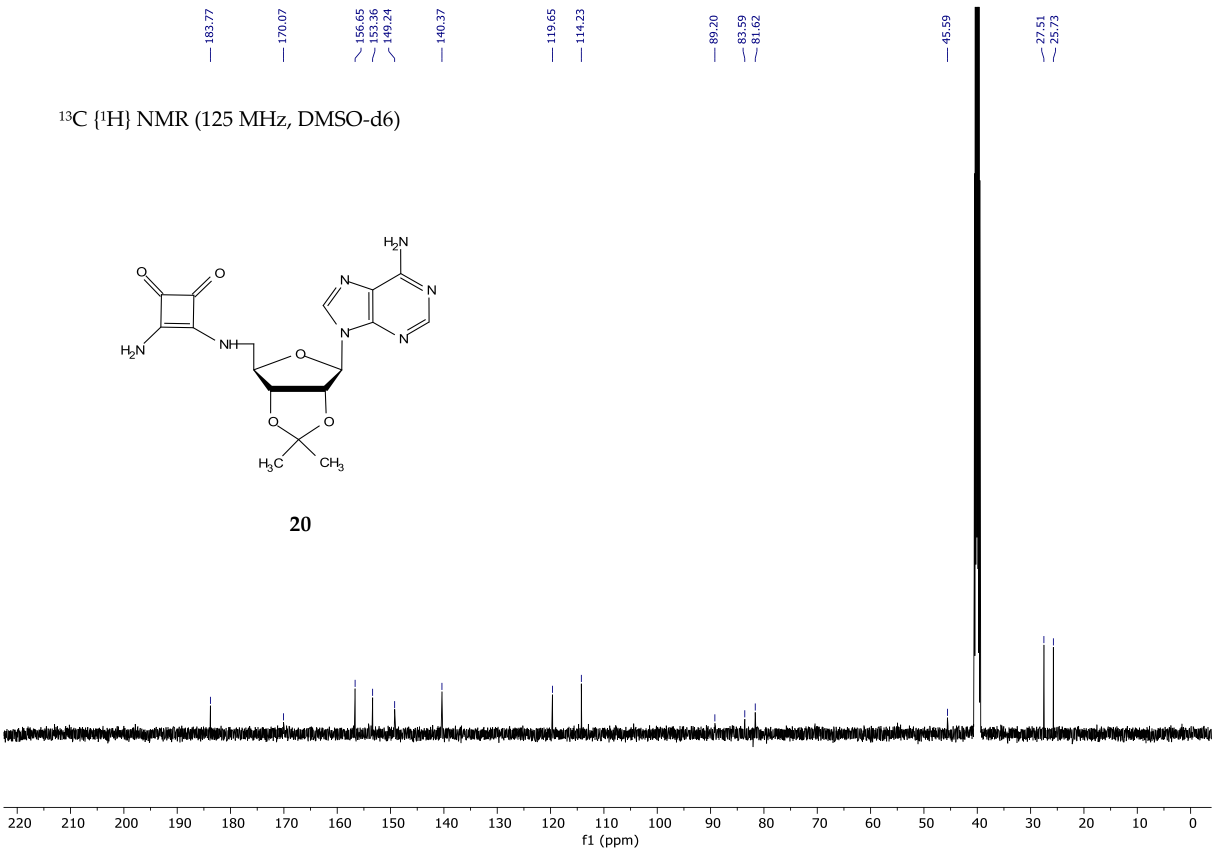

$^{13}$C {$^{1}$H} NMR (125 MHz, DMSO-d6)
20
f1 (ppm)

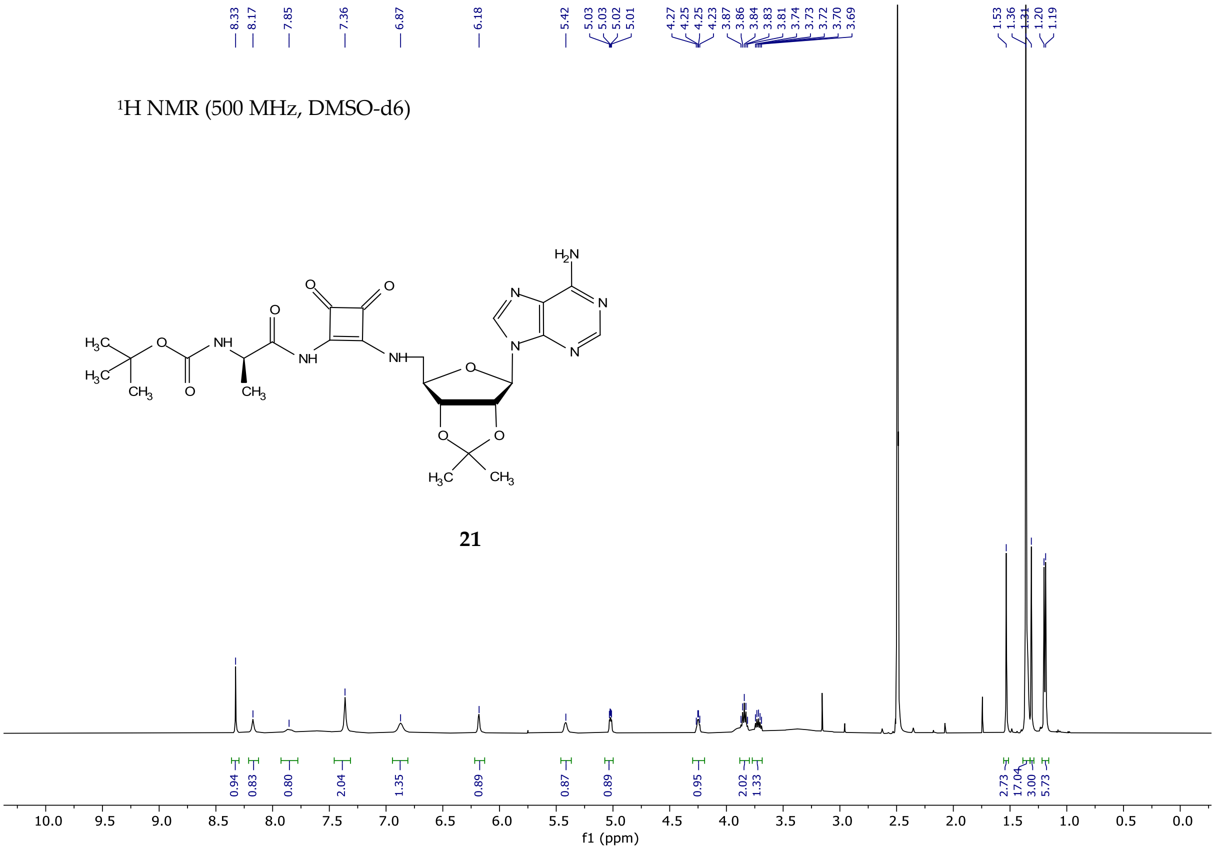

1H NMR (500 MHz, DMSO-d6)
8.33 8.17 7.85 7.36 6.87 6.18 5.42 5.03 5.03 5.02 5.01 4.27 4.25 4.25 4.23 3.87 3.86 3.84 3.83 3.81 3.74 3.73 3.72 3.70 3.69 1.53 1.36 1.31 1.20 1.19
0.94 0.83 0.80 2.04 1.35 0.89 0.87 0.89 0.95 2.02 1.33 2.73 17.04 3.00 5.73
21
f1 (ppm)


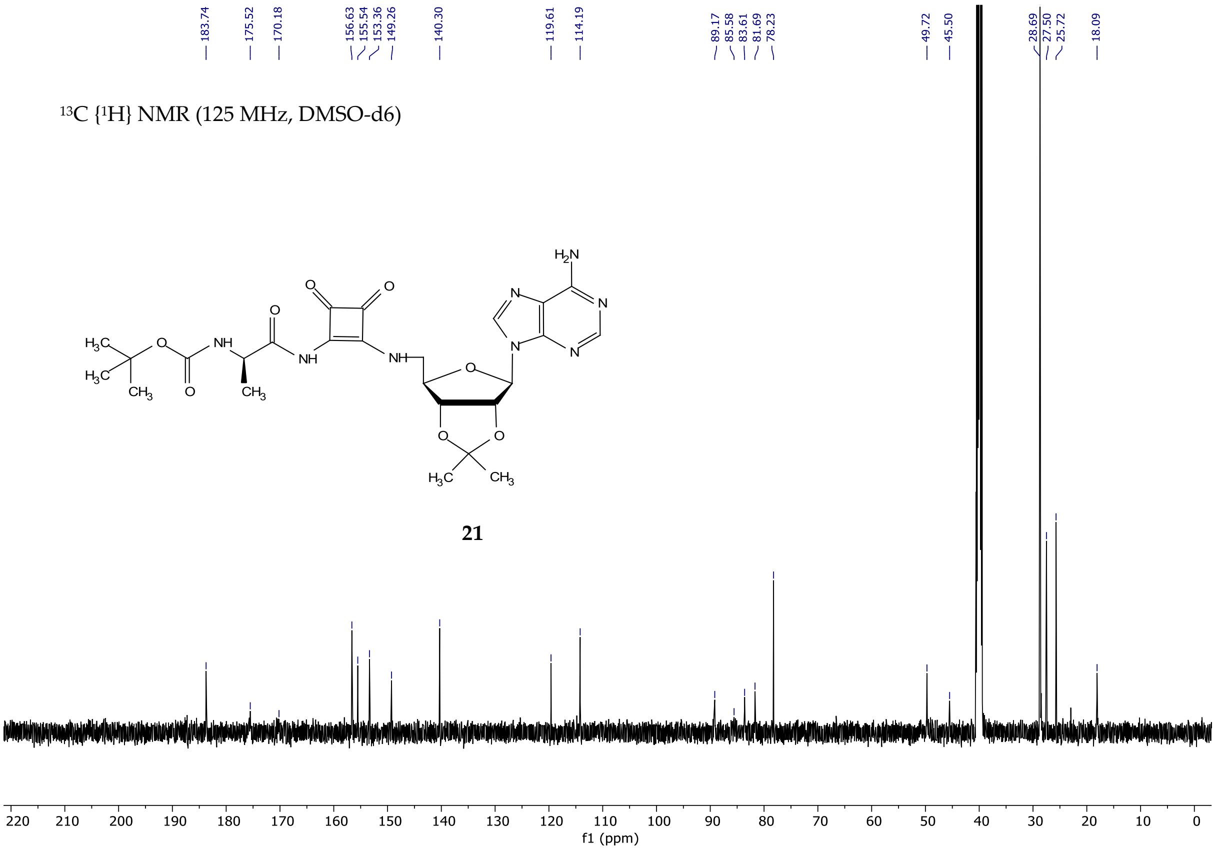

13C {1H} NMR (125 MHz, DMSO-d6)
183.74 175.52 170.18 156.63 155.54 153.36 149.26 140.30 119.61 114.19 89.17 85.58 83.61 81.69 78.23 49.72 45.50 28.69 27.50 25.72 18.09
21
f1 (ppm)

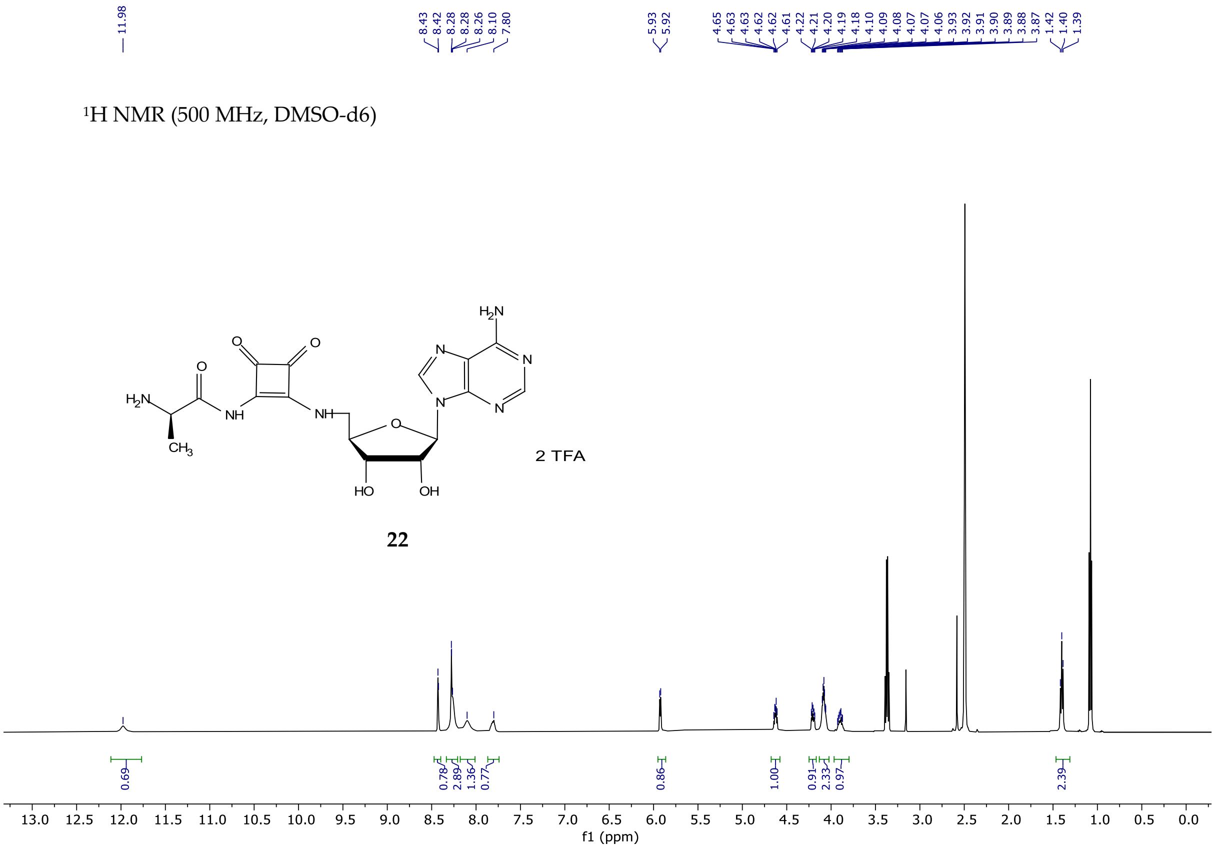
11.98
8.43 8.42 8.28 8.28 8.26 8.10 7.80
5.93 5.92
4.65 4.63 4.63 4.62 4.62 4.61 4.22 4.21 4.20 4.19 4.18 4.10 4.09 4.08 4.07 4.07 4.06 3.93 3.92 3.91 3.90 3.89 3.88 3.87 1.42 1.40 1.39
¹H NMR (500 MHz, DMSO-d6)
2 TFA
22
0.69 0.78 2.89 1.36 0.77 0.86 1.00 0.91 2.33 0.97 2.39
f1 (ppm)

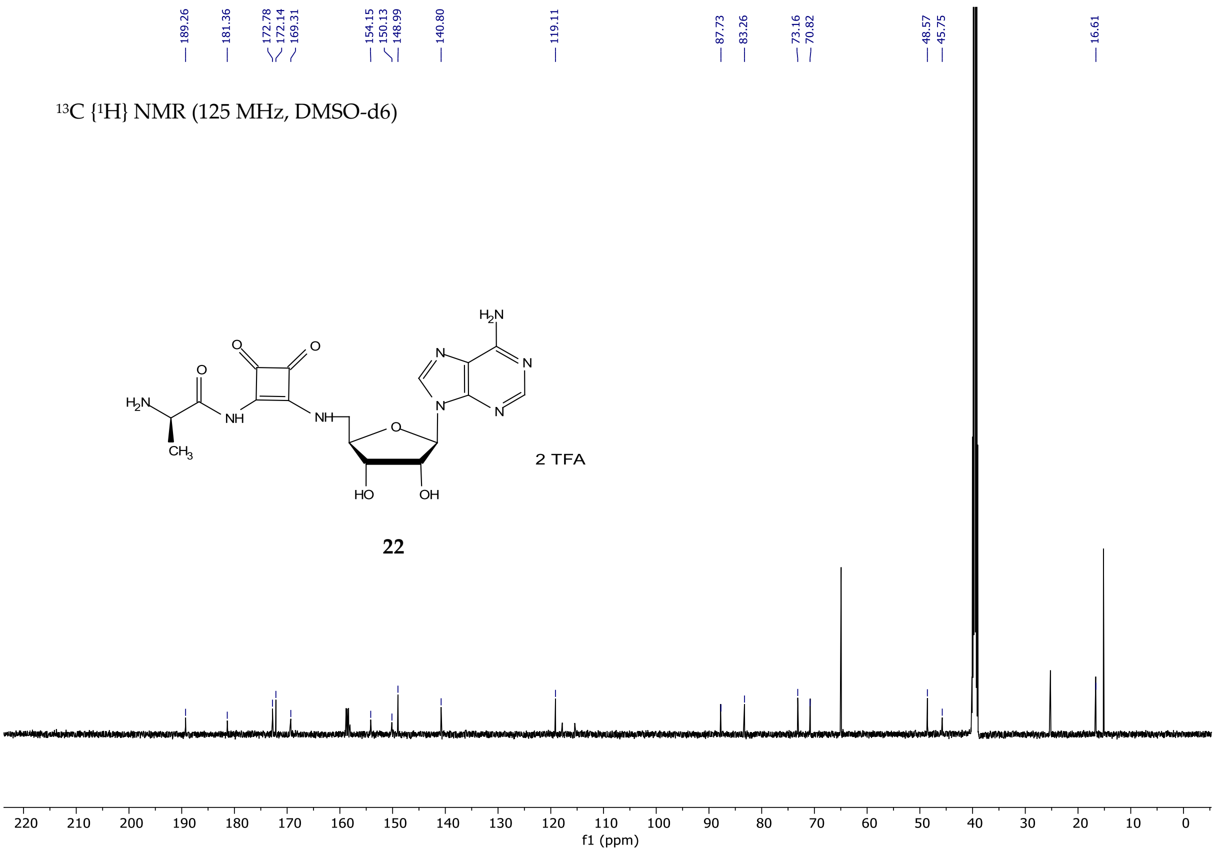
189.26 181.36 172.78 172.14 169.31 154.15 150.13 148.99 140.80 119.11 87.73 83.26 73.16 70.82 48.57 45.75 16.61
¹³C {¹H} NMR (125 MHz, DMSO-d6)
2 TFA
22
f1 (ppm)

RAPPORT HPLC

Empower Node Lcmt-hplc3

# XFGAB73

Date Acquired 19/06/2021 14:05:46 CET

## Instrument Method: Grad 955

*Stored: 19/06/2021 13:42:51 CET*

*Revision I*
This method co

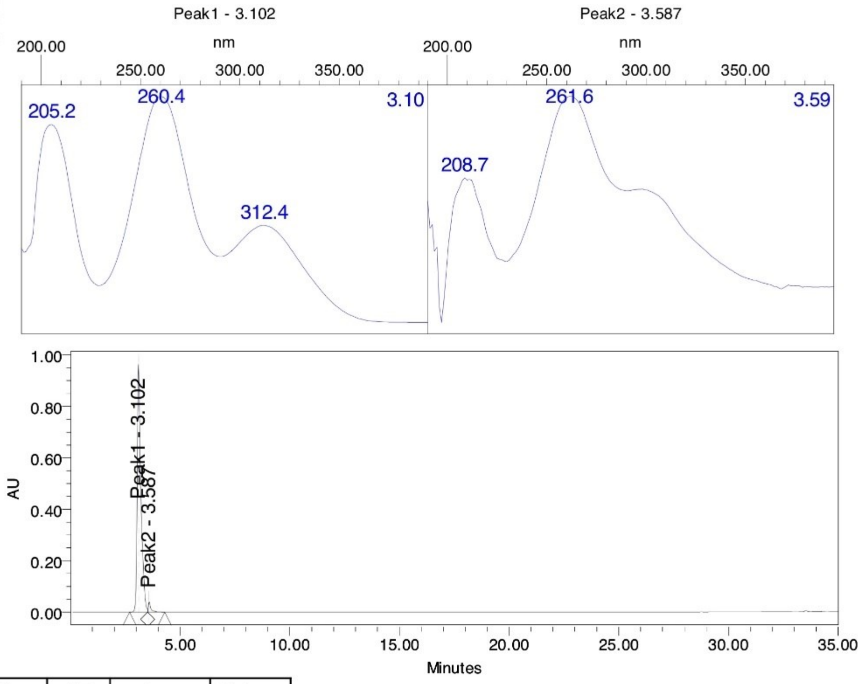


| | Peak Name | RT | Area | % Area |
|---|---|---|---|---|
| 1 | Peak1 | 3.102 | 11370859 | 97.12 |
| 2 | Peak2 | 3.587 | 336671 | 2.88 |

**PDA 254.0 nm**

Reported by User: System
Report Method: RAPPORT HPLC
Report Method ID: [illegible]8150
Page: 1 of 1

Project Name: X-terra 2018
Date Printed:
16/05/2025
13:51:18 Europe/Paris

HPLC analysis for compound **22**

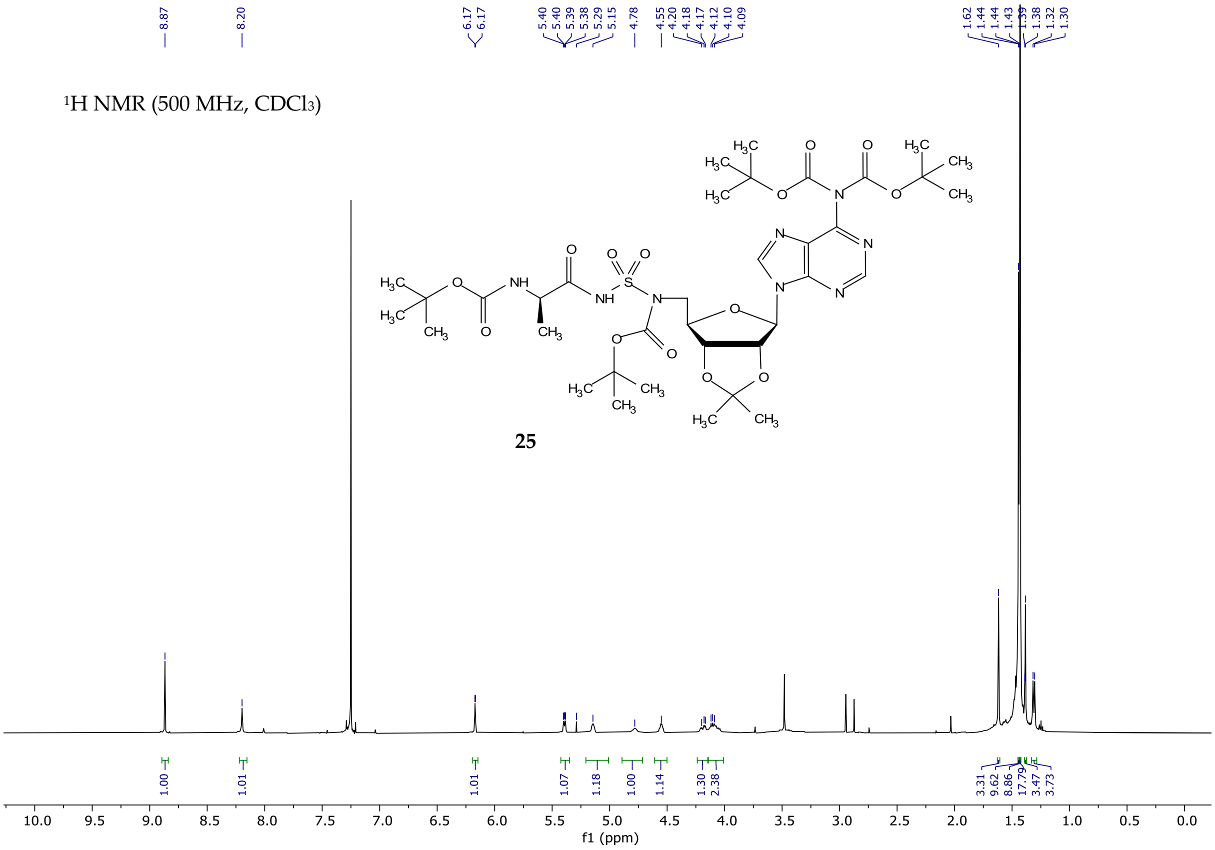
1H NMR (500 MHz, CDCl3)
8.87
8.20
6.17 6.17
5.40 5.40 5.39 5.38 5.29 5.15
4.78
4.55 4.20 4.18 4.17 4.12 4.10 4.09
1.62 1.44 1.44 1.43 1.39 1.38 1.32 1.30
1.00 1.01 1.01 1.07 1.18 1.00 1.14 1.30 2.38
3.31 9.62 8.86 17.79 3.47 3.73
25
f1 (ppm)

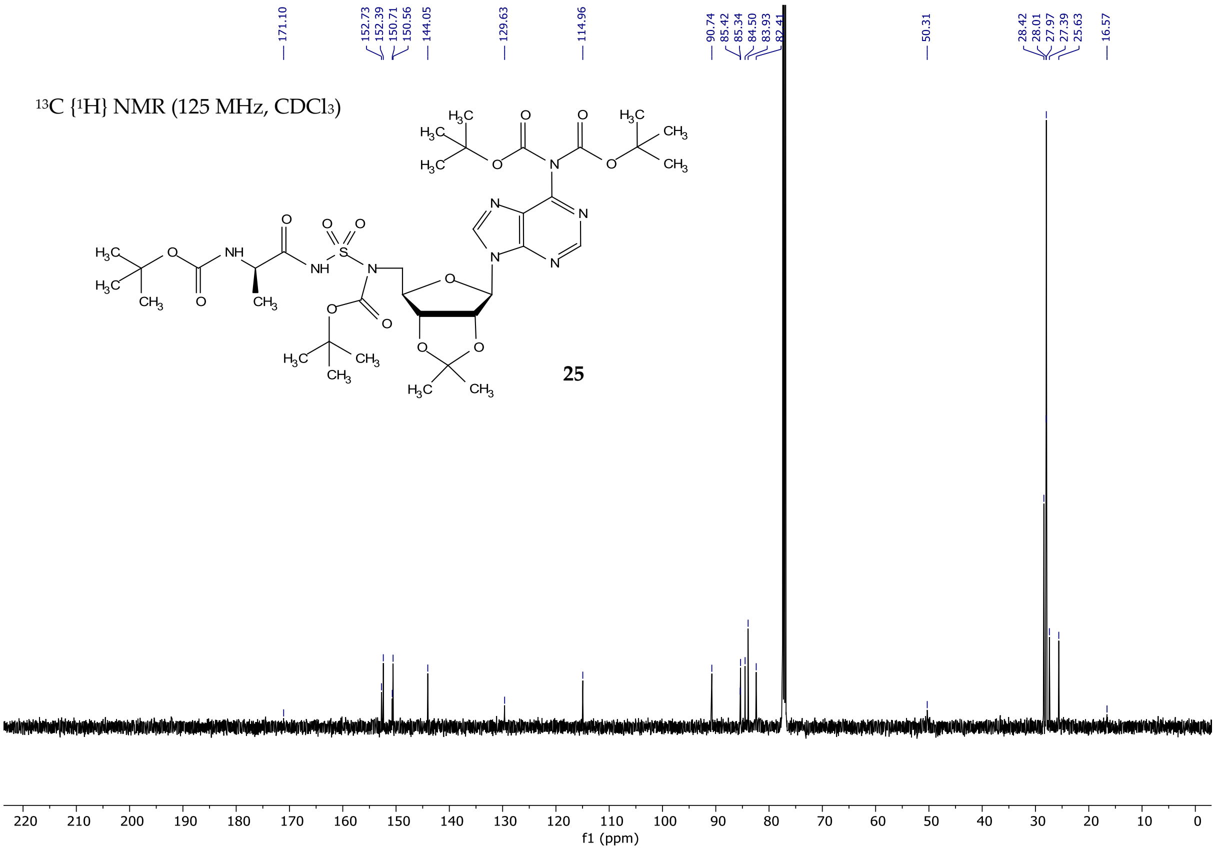
13C {1H} NMR (125 MHz, CDCl3)
171.10
152.73 152.39 150.71 150.56 144.05
129.63
114.96
90.74 85.42 85.34 84.50 83.93 82.41
50.31
28.42 28.01 27.97 27.39 25.63
16.57
25
f1 (ppm)

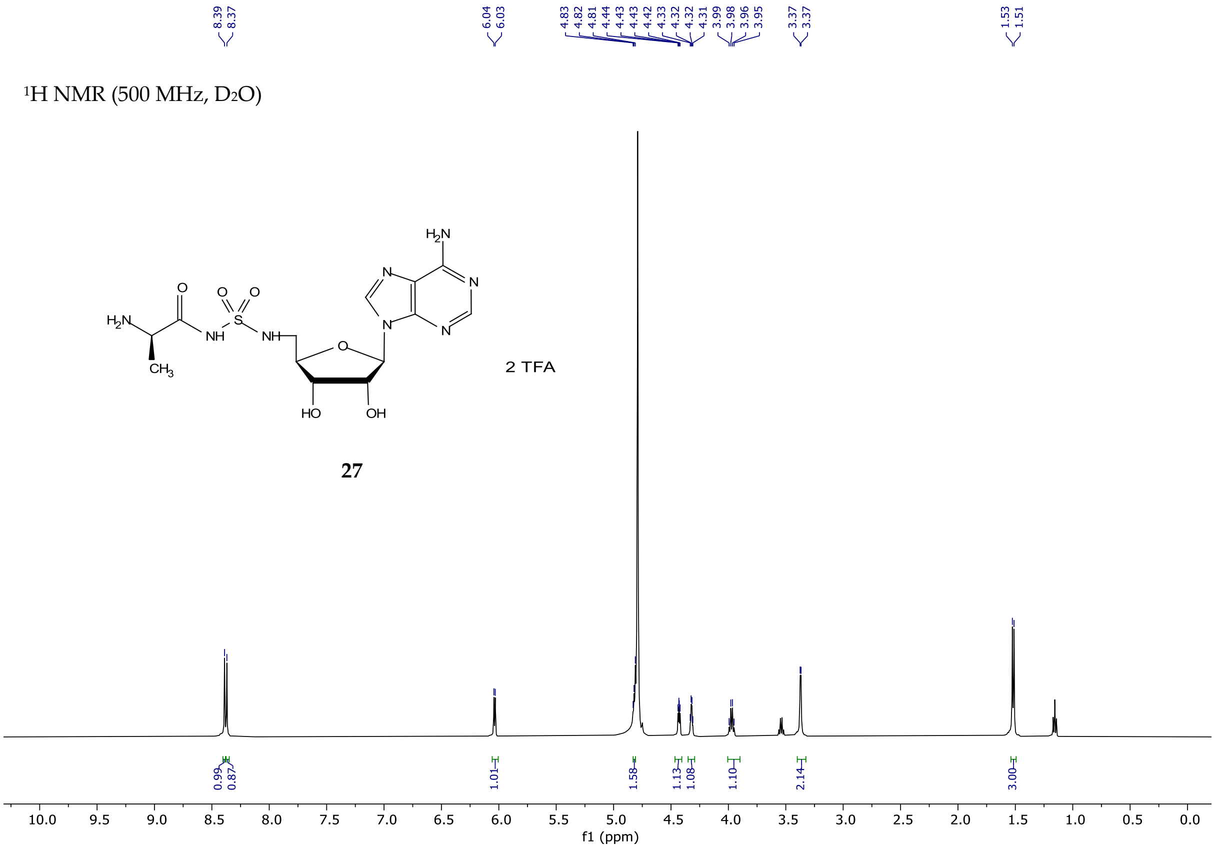
8.39
8.37
6.04
6.03
4.83
4.82
4.81
4.44
4.43
4.43
4.42
4.33
4.32
4.32
4.31
3.99
3.98
3.96
3.95
3.37
3.37
1.53
1.51
1H NMR (500 MHz, D2O)
H2N
O
NH
O
S
O
NH
O
HO
OH
N
N
N
N
H2N
CH3
2 TFA
27
0.99
0.87
1.01
1.58
1.13
1.08
1.10
2.14
3.00
10.0
9.5
9.0
8.5
8.0
7.5
7.0
6.5
6.0
5.5
5.0
4.5
4.0
3.5
3.0
2.5
2.0
1.5
1.0
0.5
0.0
f1 (ppm)

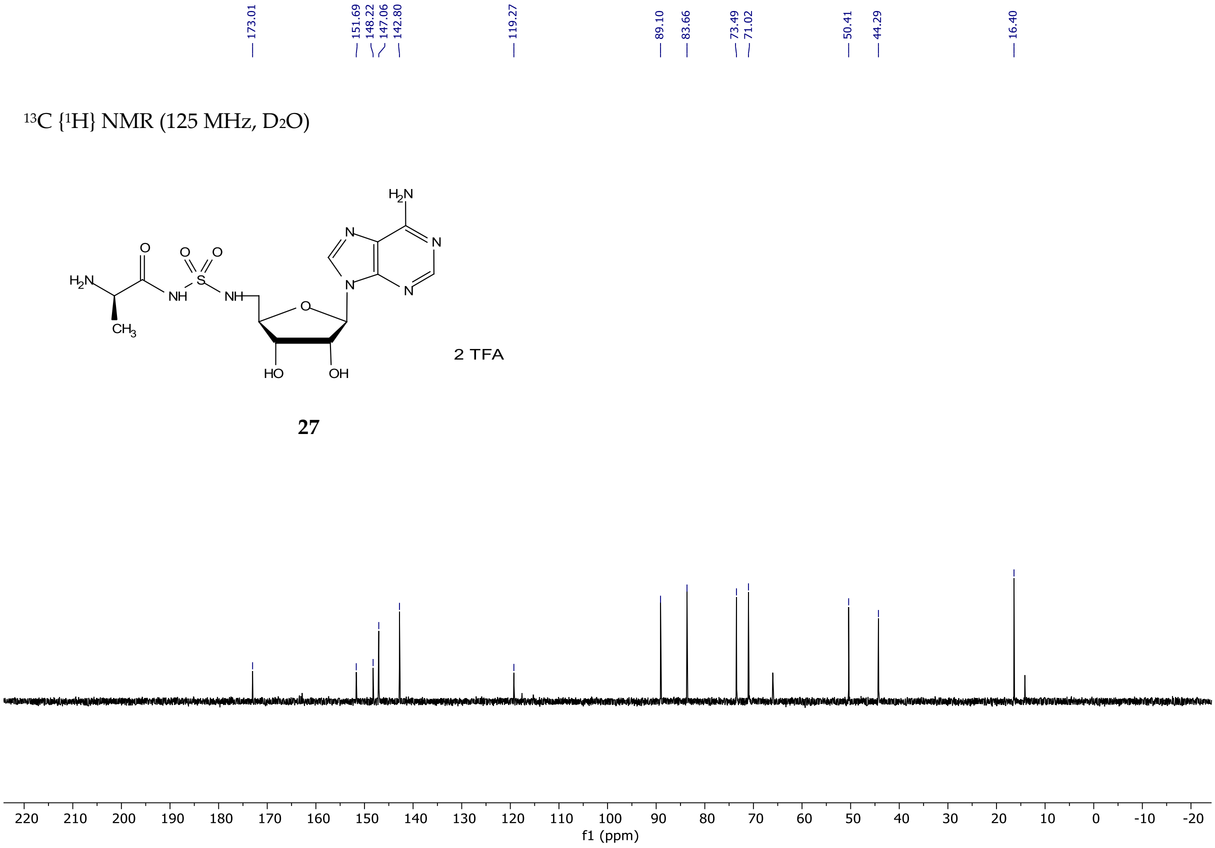
173.01
151.69
148.22
147.06
142.80
119.27
89.10
83.66
73.49
71.02
50.41
44.29
16.40
13C {1H} NMR (125 MHz, D2O)
H2N
O
NH
O
S
O
NH
O
HO
OH
N
N
N
N
H2N
CH3
2 TFA
27
220
210
200
190
180
170
160
150
140
130
120
110
100
90
80
70
60
50
40
30
20
10
0
-10
-20
f1 (ppm)

$^{1}H$ NMR (600 MHz, $CD_3CN$)

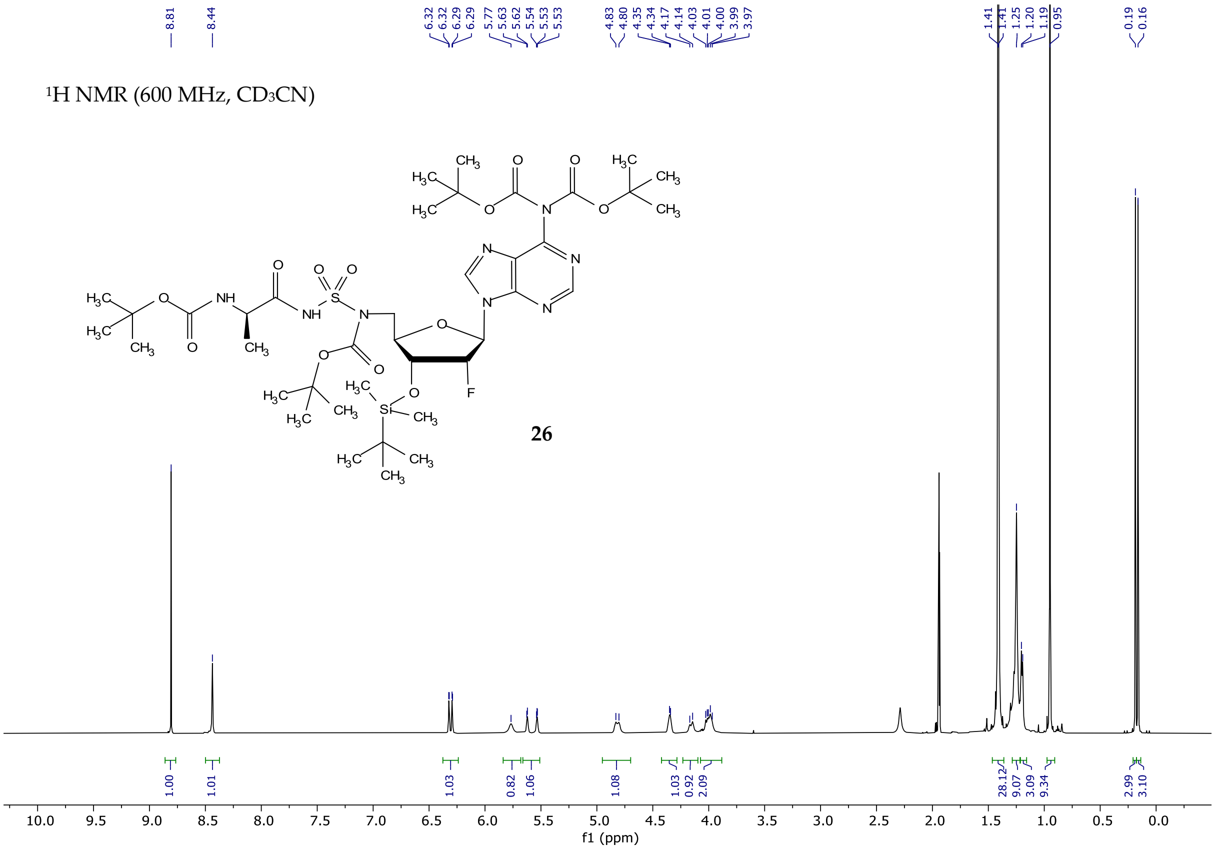


$^{19}F$ NMR (470 MHz, $CD_3CN$)

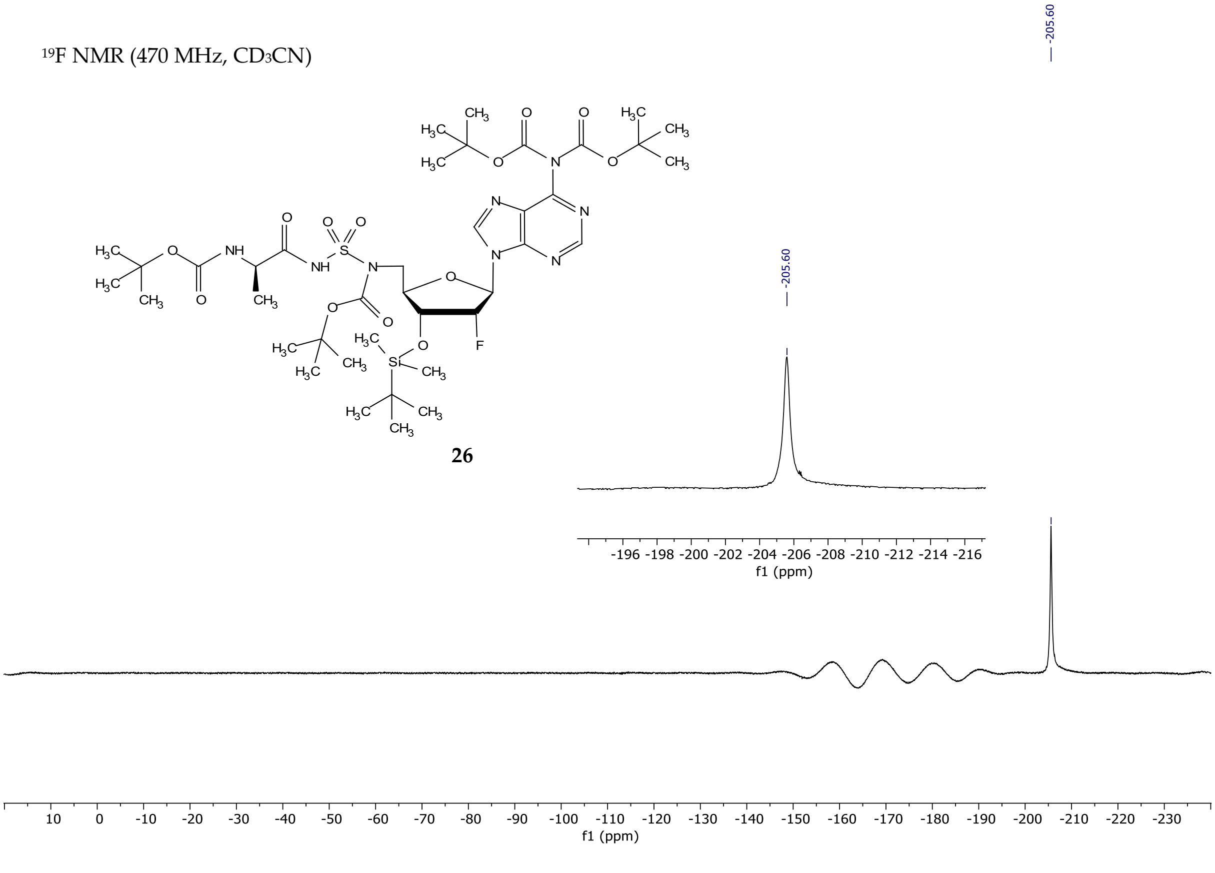

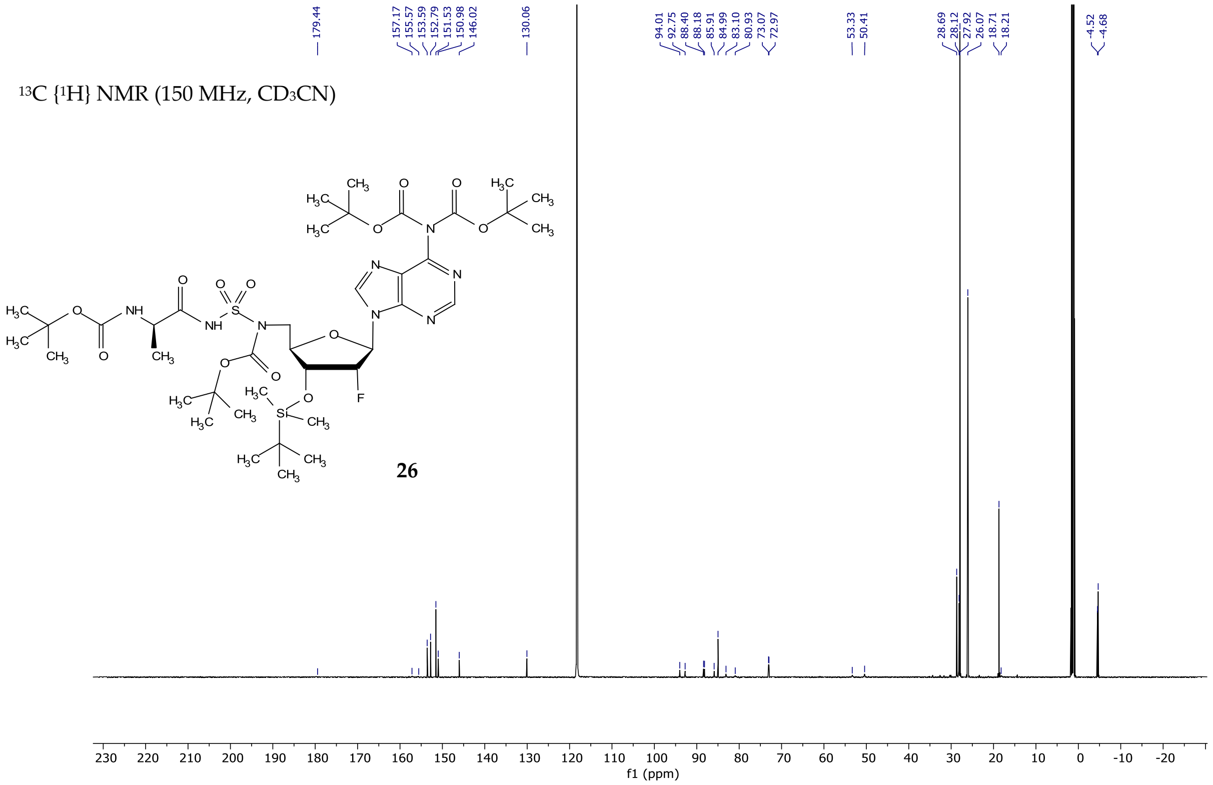

$^{13}C$ {$^{1}H$} NMR (150 MHz, $CD_3CN$)
179.44
157.17
155.57
153.59
152.79
151.53
150.98
146.02
130.06
94.01
92.75
88.40
88.18
85.91
84.99
83.10
80.93
73.07
72.97
53.33
50.41
28.69
28.12
27.92
26.07
18.71
18.21
-4.52
-4.68
26
230 220 210 200 190 180 170 160 150 140 130 120 110 100 90 80 70 60 50 40 30 20 10 0 -10 -20
f1 (ppm)

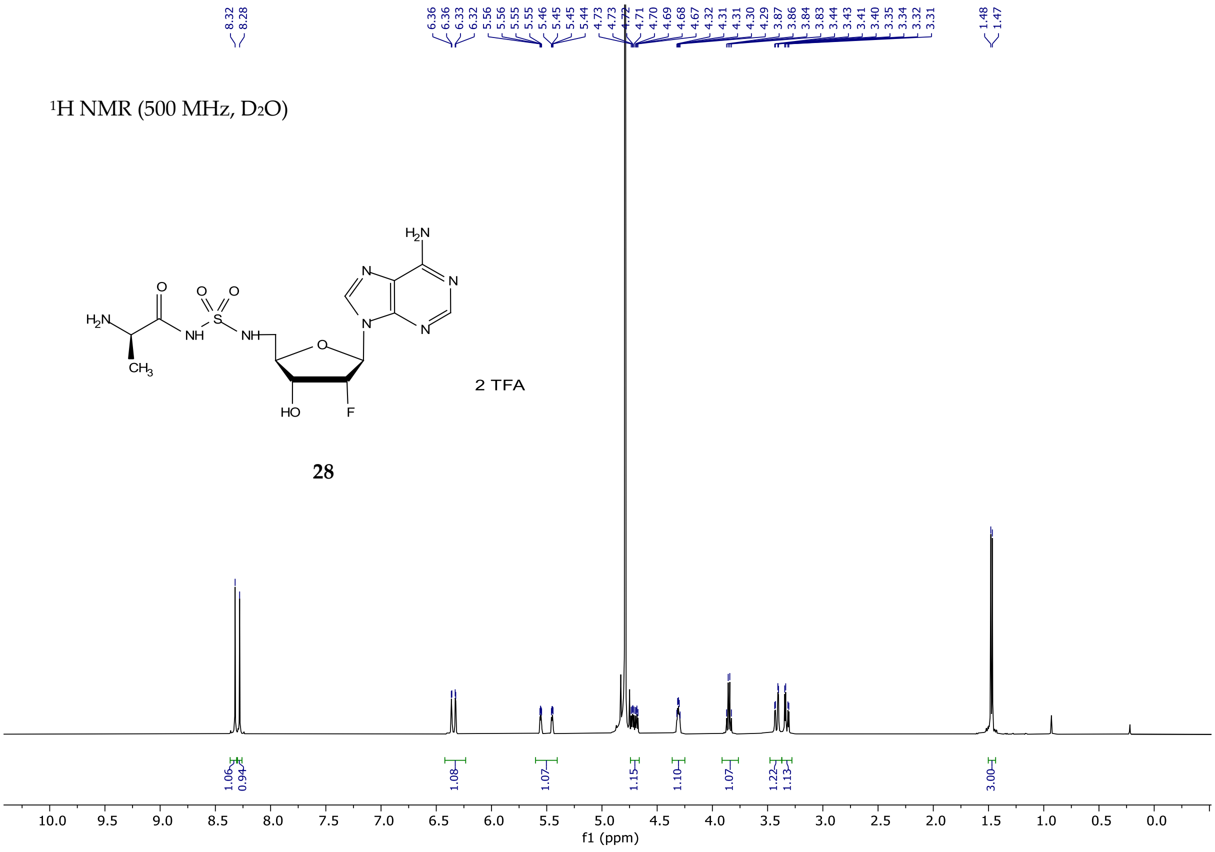
8.32
8.28
6.36
6.36
6.33
6.32
5.56
5.56
5.55
5.55
5.46
5.45
5.45
5.44
4.73
4.73
4.72
4.71
4.70
4.69
4.68
4.67
4.32
4.31
4.31
4.30
4.29
3.87
3.86
3.84
3.83
3.44
3.43
3.41
3.40
3.35
3.34
3.32
3.31
1.48
1.47
¹H NMR (500 MHz, D₂O)
H₂N
O
O
O
S
NH
NH
O
N
N
N
N
HO
F
CH₃
2 TFA
28
1.06
0.94
1.08
1.07
1.15
1.10
1.07
1.22
1.13
3.00
10.0 9.5 9.0 8.5 8.0 7.5 7.0 6.5 6.0 5.5 5.0 4.5 4.0 3.5 3.0 2.5 2.0 1.5 1.0 0.5 0.0
f1 (ppm)

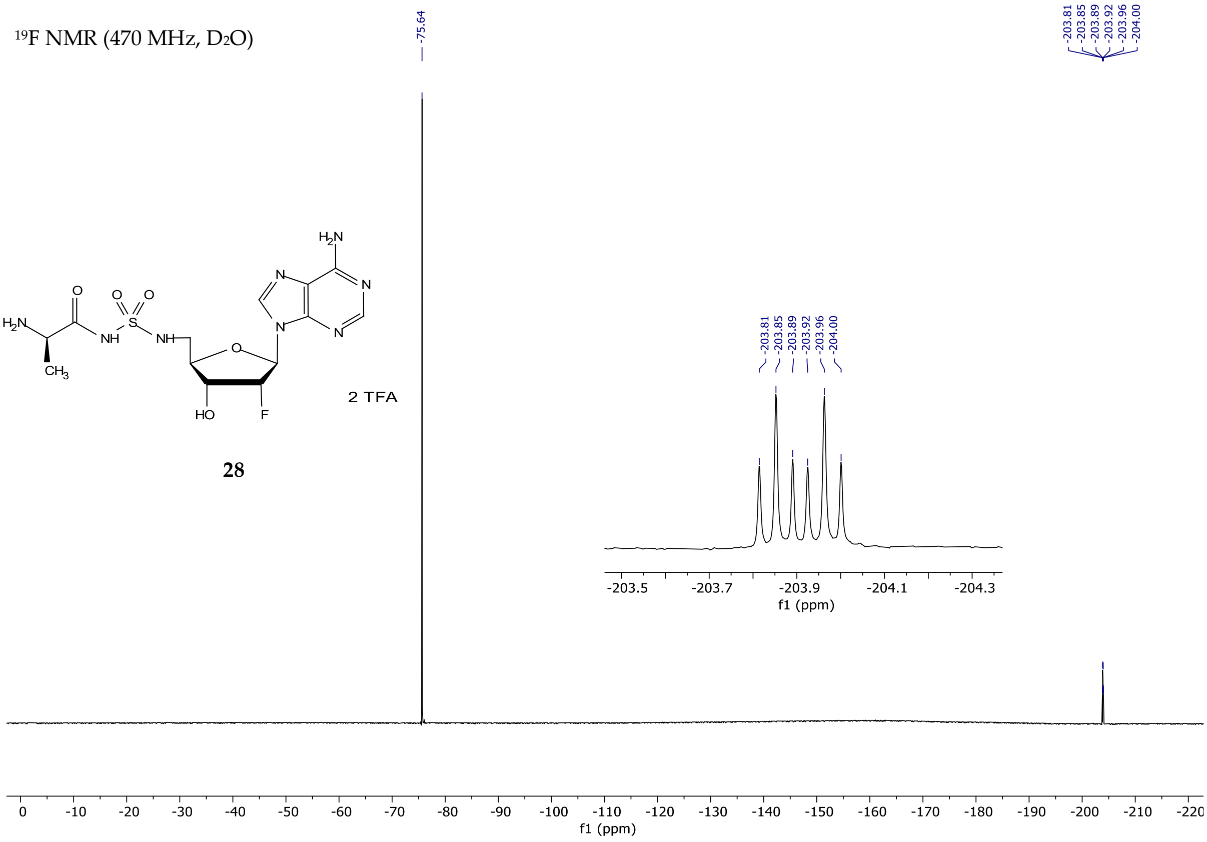
¹⁹F NMR (470 MHz, D₂O)
-75.64
-203.81
-203.85
-203.89
-203.92
-203.96
-204.00
H₂N
O
O
O
S
NH
NH
O
N
N
N
N
HO
F
CH₃
2 TFA
28
-203.5 -203.7 -203.9 -204.1 -204.3
f1 (ppm)
0 -10 -20 -30 -40 -50 -60 -70 -80 -90 -100 -110 -120 -130 -140 -150 -160 -170 -180 -190 -200 -210 -220
f1 (ppm)

$^{13}C$ {$^{1}H$} NMR (150 MHz, $CD_3CN$)

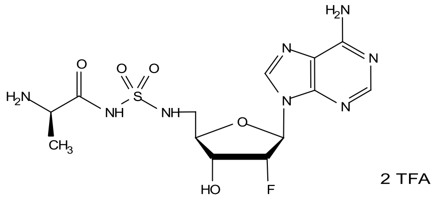


**28**

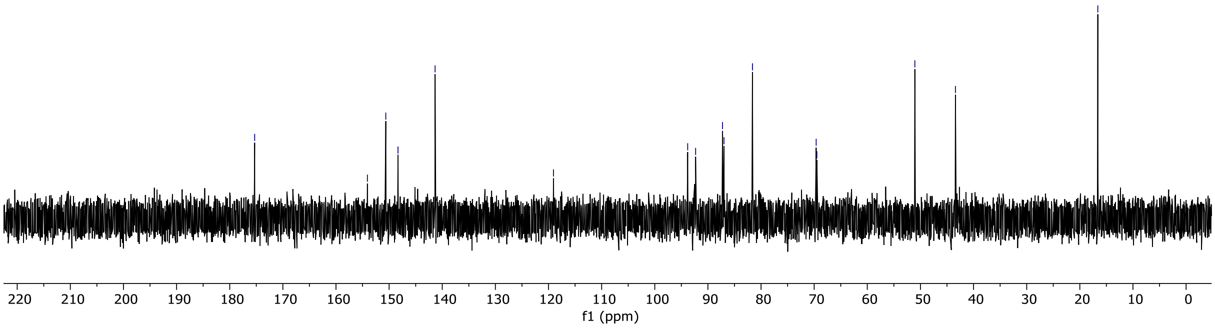

Empower2 software

RAPPORT HPLC

Empower Node Lcmt-hplc3

**X-terra 2018**

**Colonne Waters XterraMS C18 3.5µm 4.6x150mm**

# DL341A

Date Acquired 06/12/2021 11:59:56 CET

## Instrument Method: im PURETE HPLC

*Stored: 06/12/2021 11:01:47 CET*

*Revision i*

This method cc

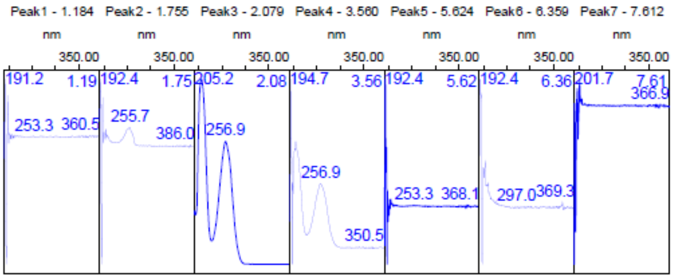


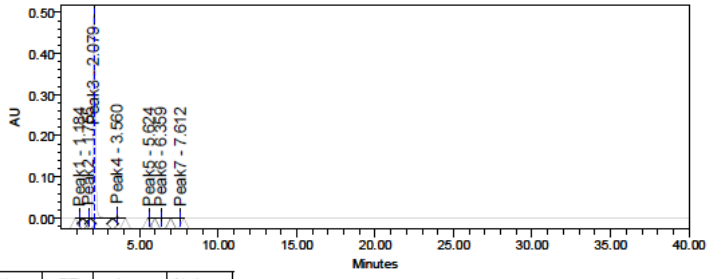


| | Peak Name | RT | Area | % Area |
|---|---|---|---|---|
| 1 | Peak1 | 1.184 | 1434 | 0.03 |
| 2 | Peak2 | 1.755 | 15083 | 0.31 |
| 3 | Peak3 | 2.079 | 4756124 | 98.26 |
| 4 | Peak4 | 3.560 | 62657 | 1.29 |

Reported by User: System
Report Method: RAPPORT HPLC
Report Method ID: 6053
Page: 1 of 2

Project Name: X-terra 2018
Date Printed:
06/12/2021
15:01:06 Europe/Paris

HPLC analysis for compound **28**

$^{1}H$ NMR (500 MHz, $CDCl_3$)

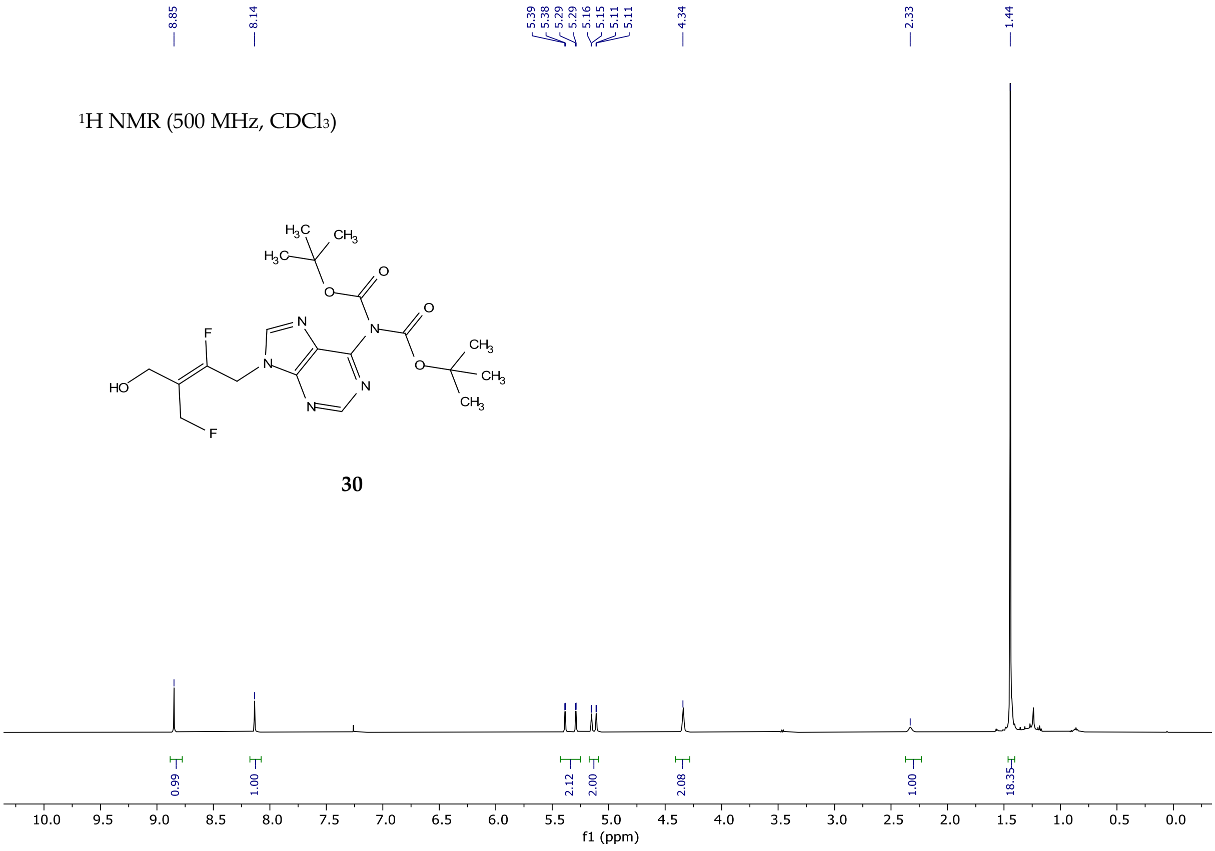


30

$^{19}F$ NMR (470 MHz, $CDCl_3$)

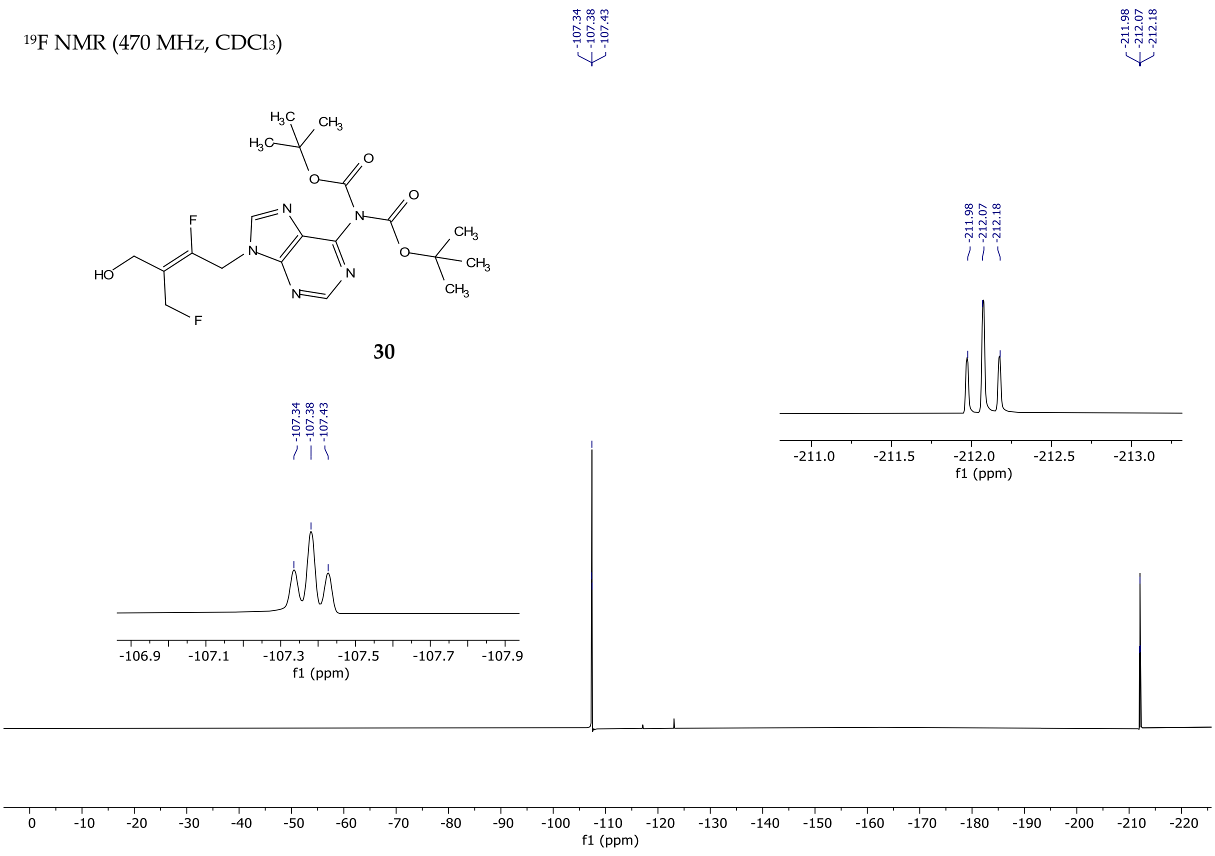


30

$^{13}C$ {$^{1}H$} NMR (125 MHz, $CDCl_3$)

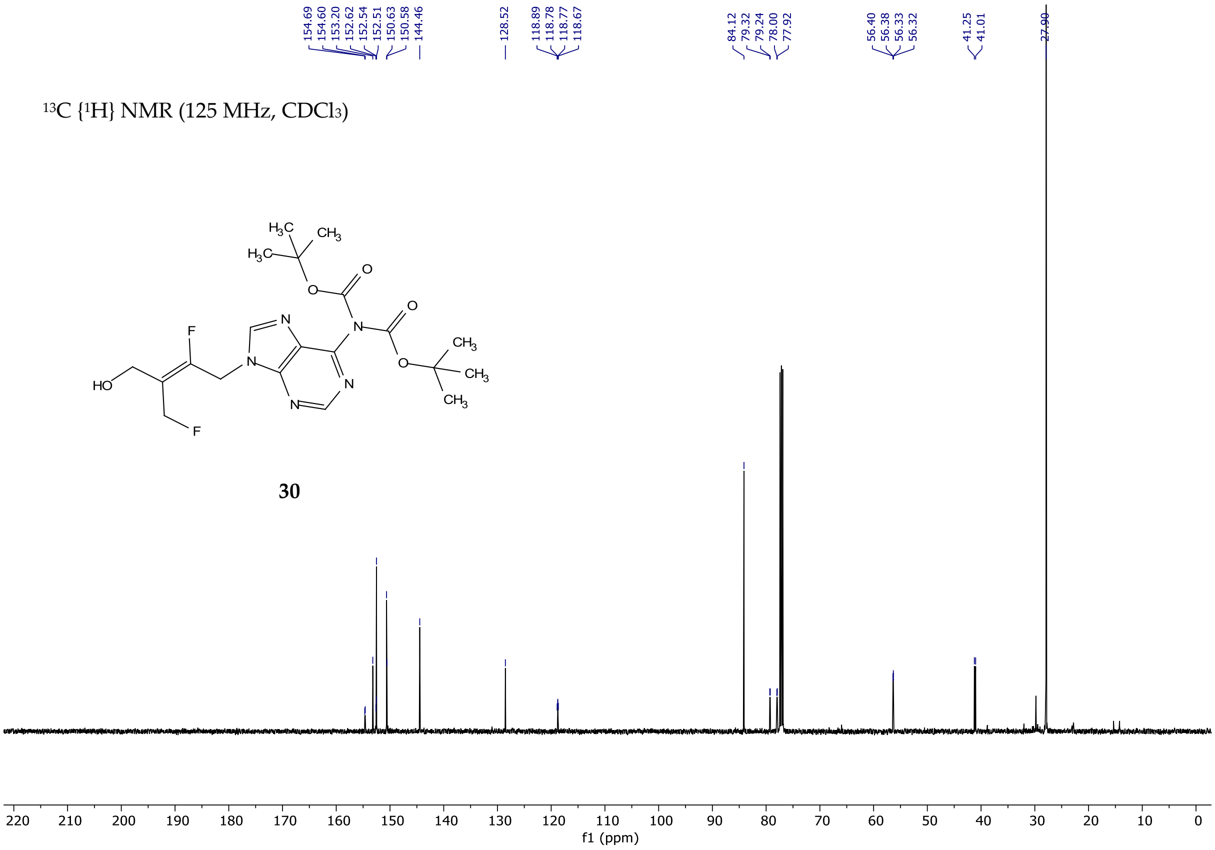


**30**

$^{1}H$ NMR (500 MHz, $CDCl_3$)

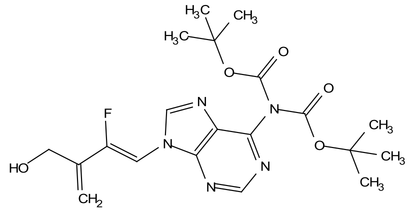


**31**

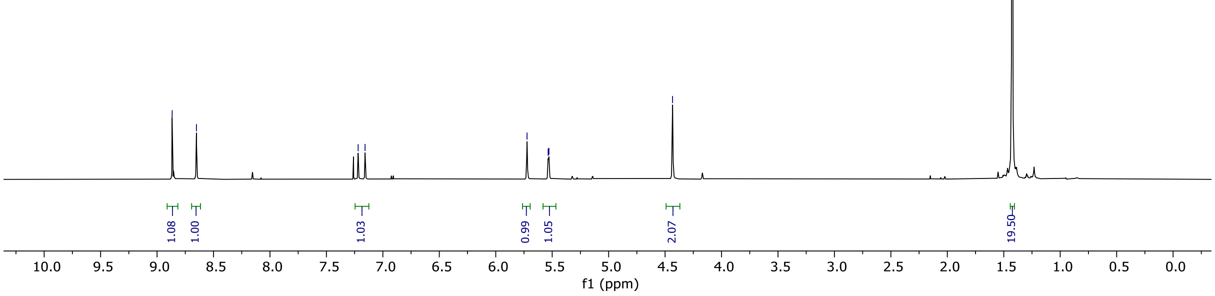


$^{19}F$ NMR (470 MHz, $CDCl_3$)

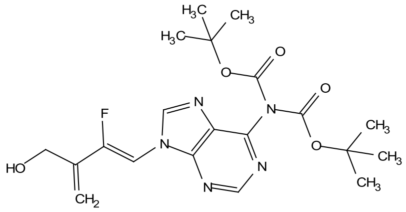


**31**

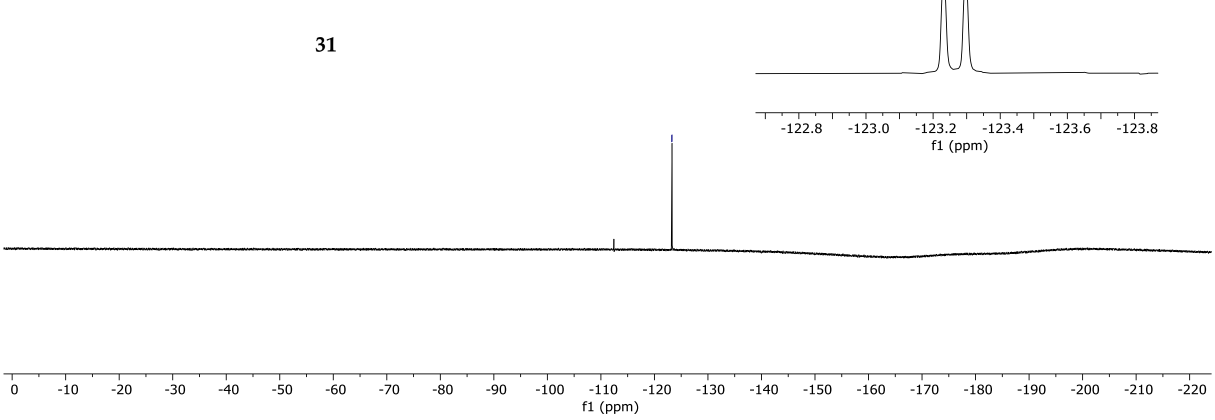

$^{13}C$ {$^{1}H$} NMR (125 MHz, $CDCl_3$)

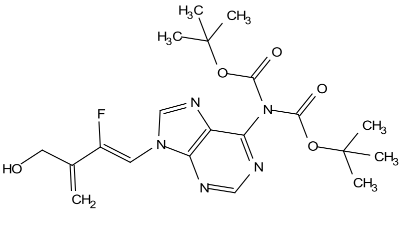


**31**

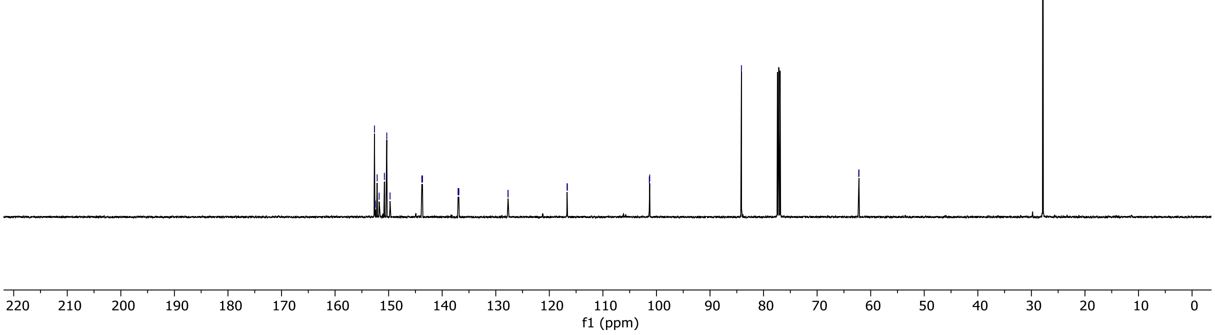

$^{1}H$ NMR (600 MHz, $CDCl_3$)

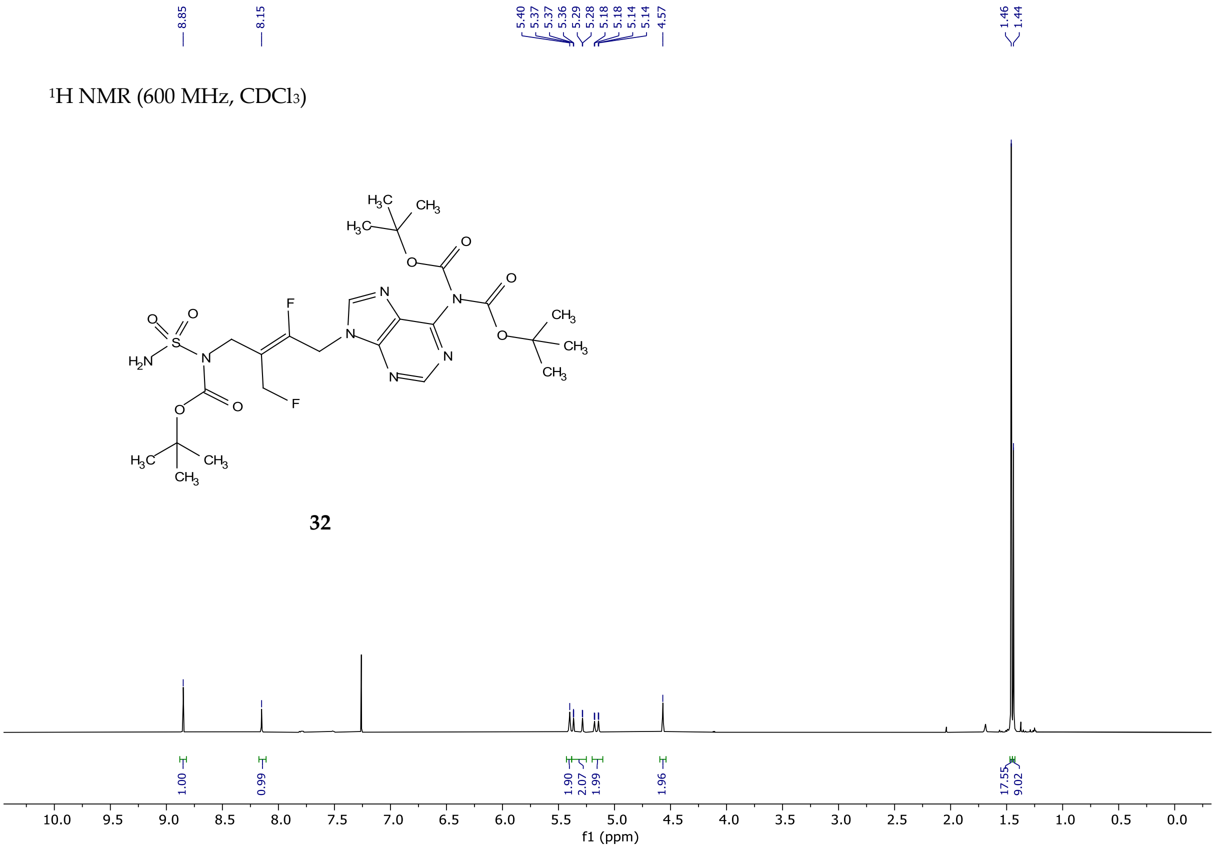


$^{19}F$ NMR (470 MHz, $CDCl_3$)

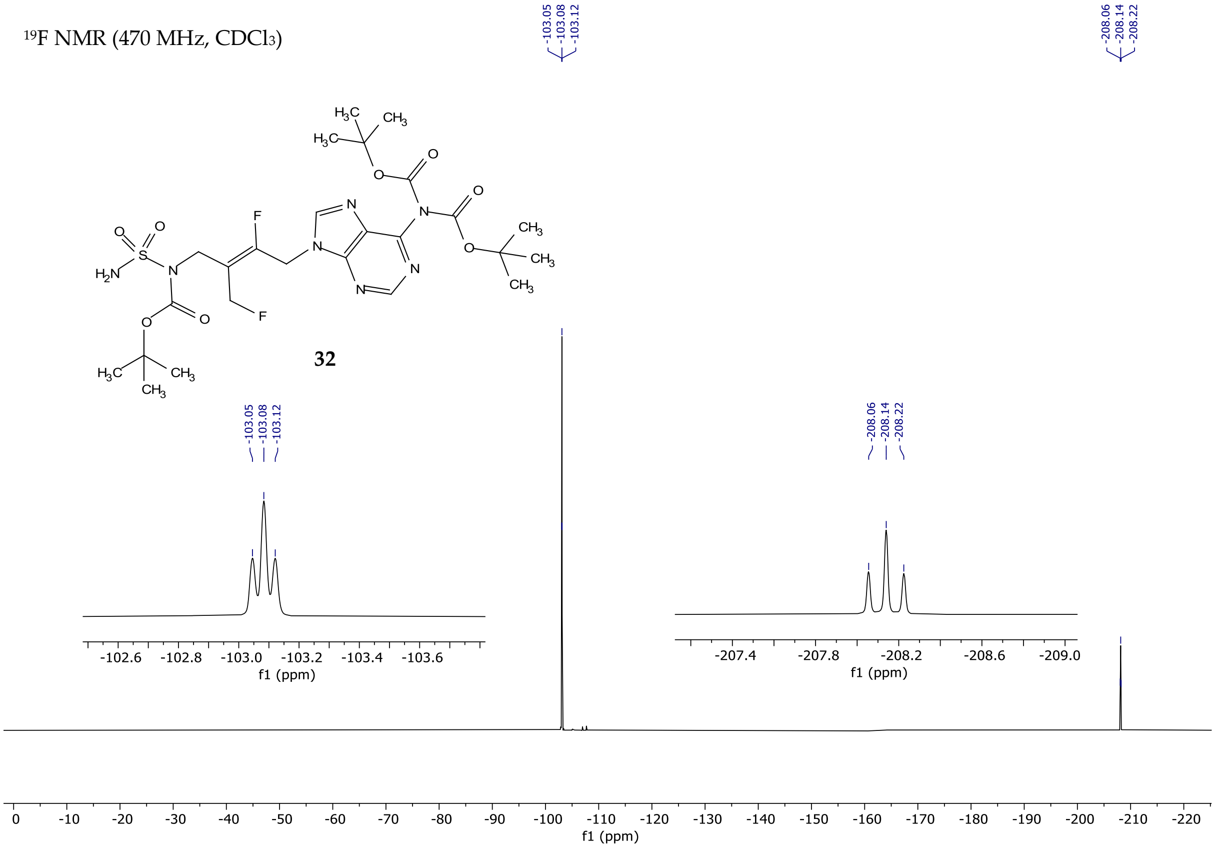

$^{13}C\{^{1}H\}$ NMR (125 MHz, $CDCl_3$)

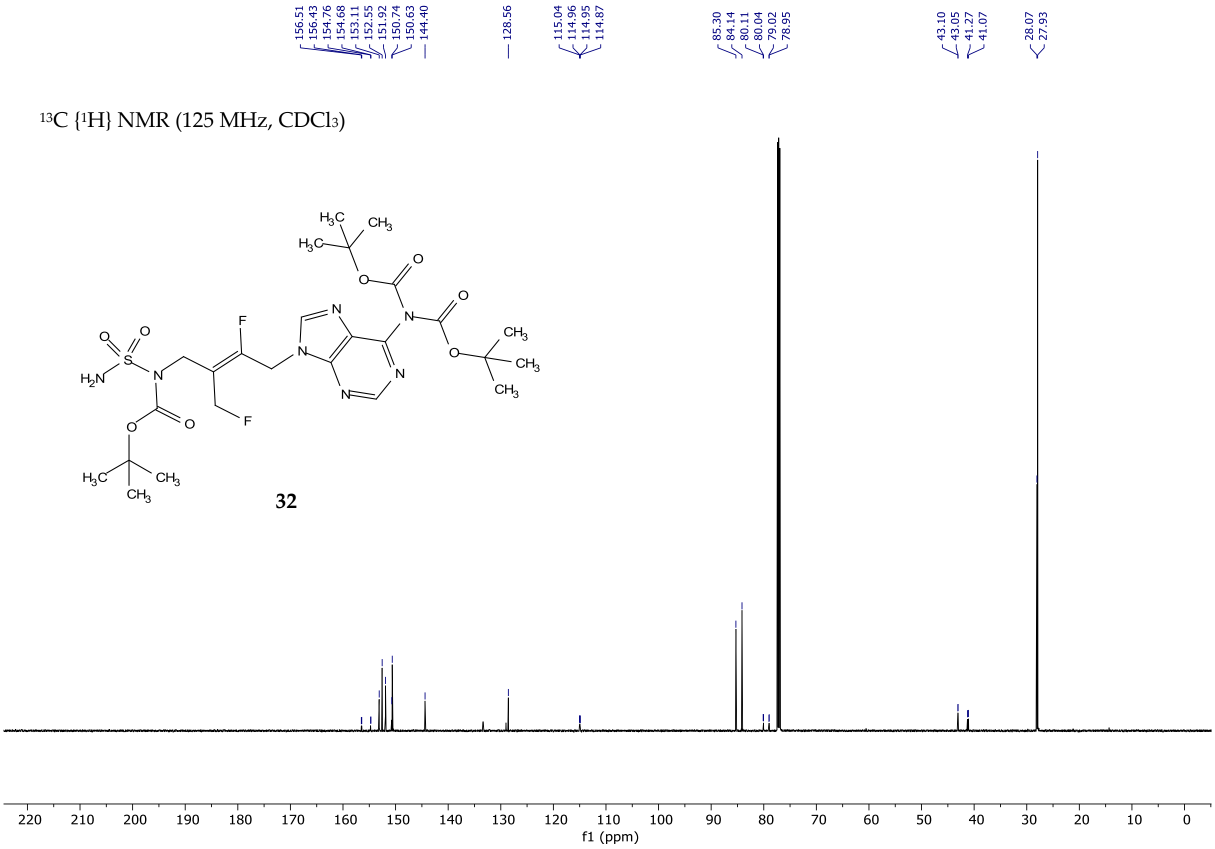


**32**

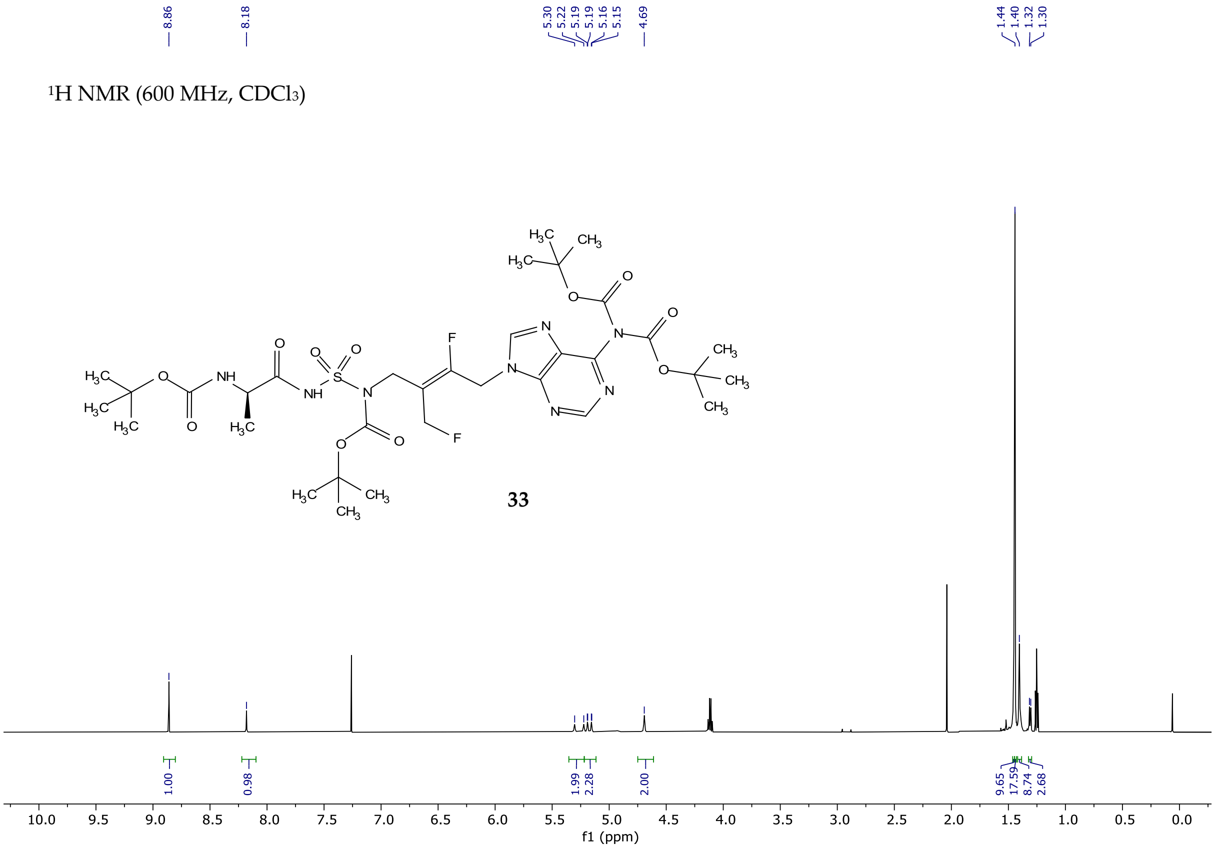
8.86
8.18
5.30
5.22
5.19
5.19
5.16
5.15
4.69
1.44
1.40
1.32
1.30
¹H NMR (600 MHz, CDCl3)
33
1.00
0.98
1.99
2.28
2.00
9.65
17.59
8.74
2.68
f1 (ppm)

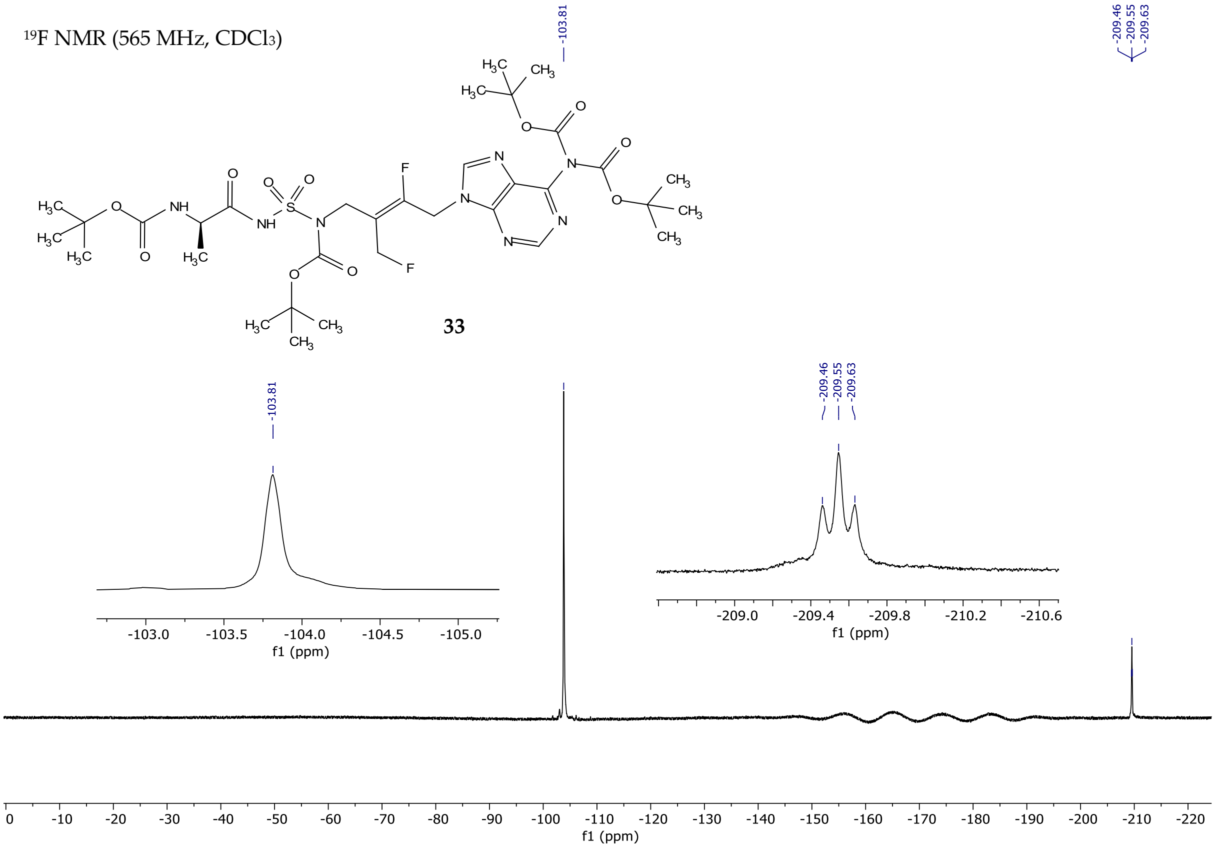
¹⁹F NMR (565 MHz, CDCl3)
-103.81
-209.46
-209.55
-209.63
33
f1 (ppm)

$^{13}C\{^{1}H\}$ NMR (150 MHz, $CDCl_3$)

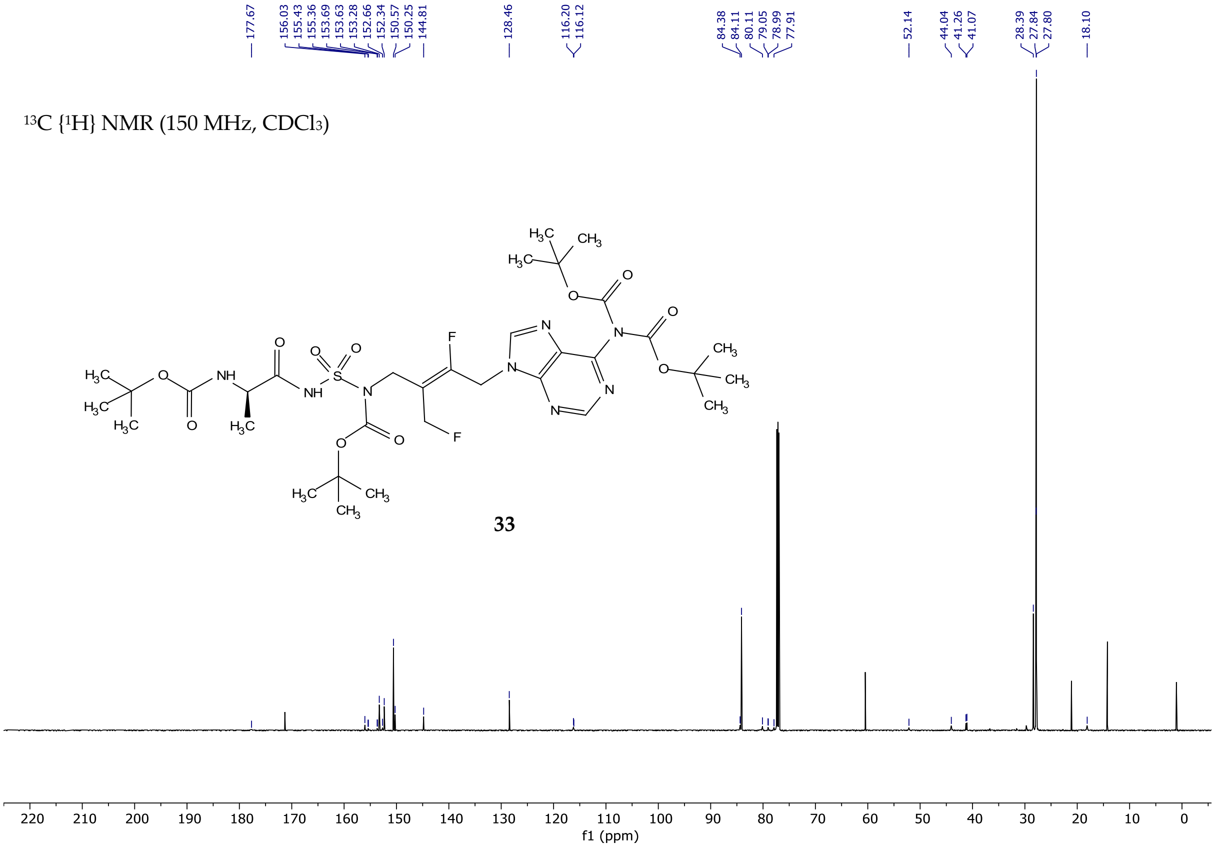


33

$^{1}$H NMR (600 MHz, MeOD)

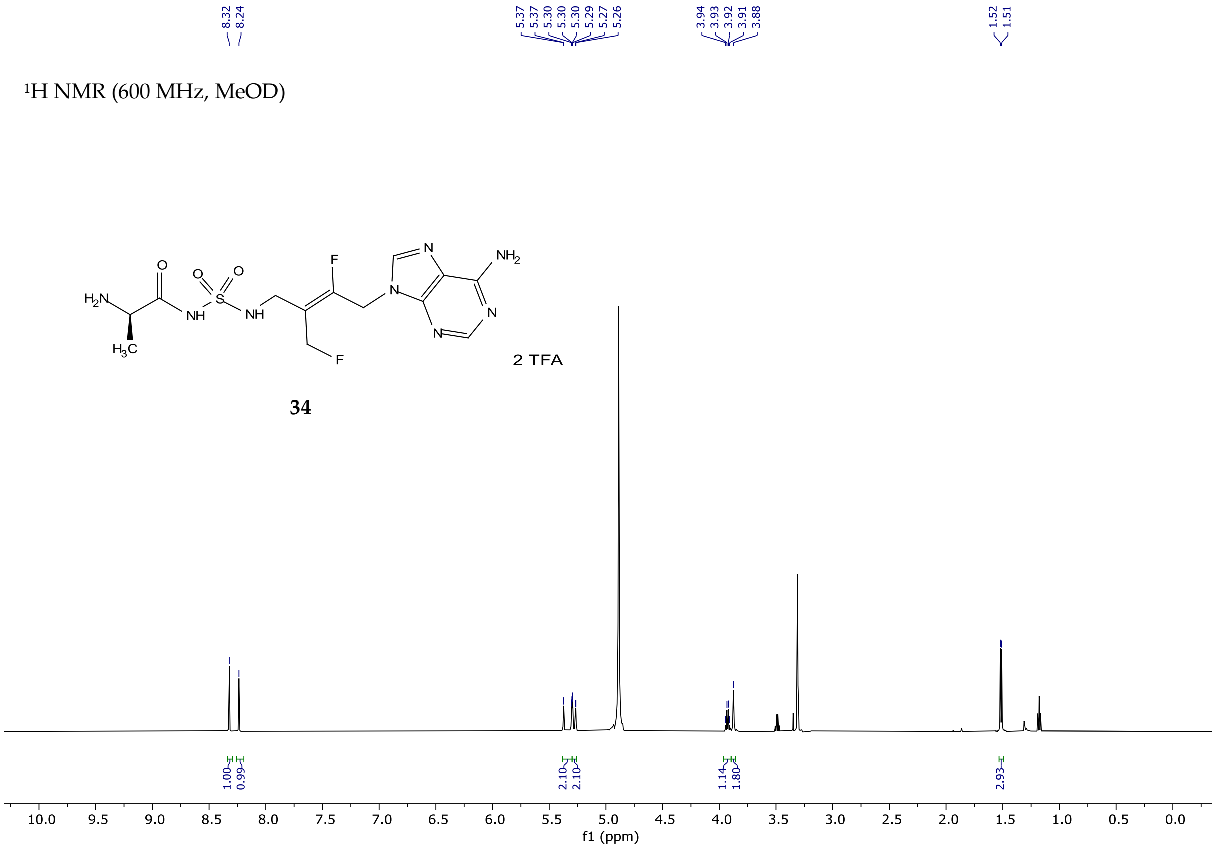


$^{19}$F NMR (565 MHz, MeOD)

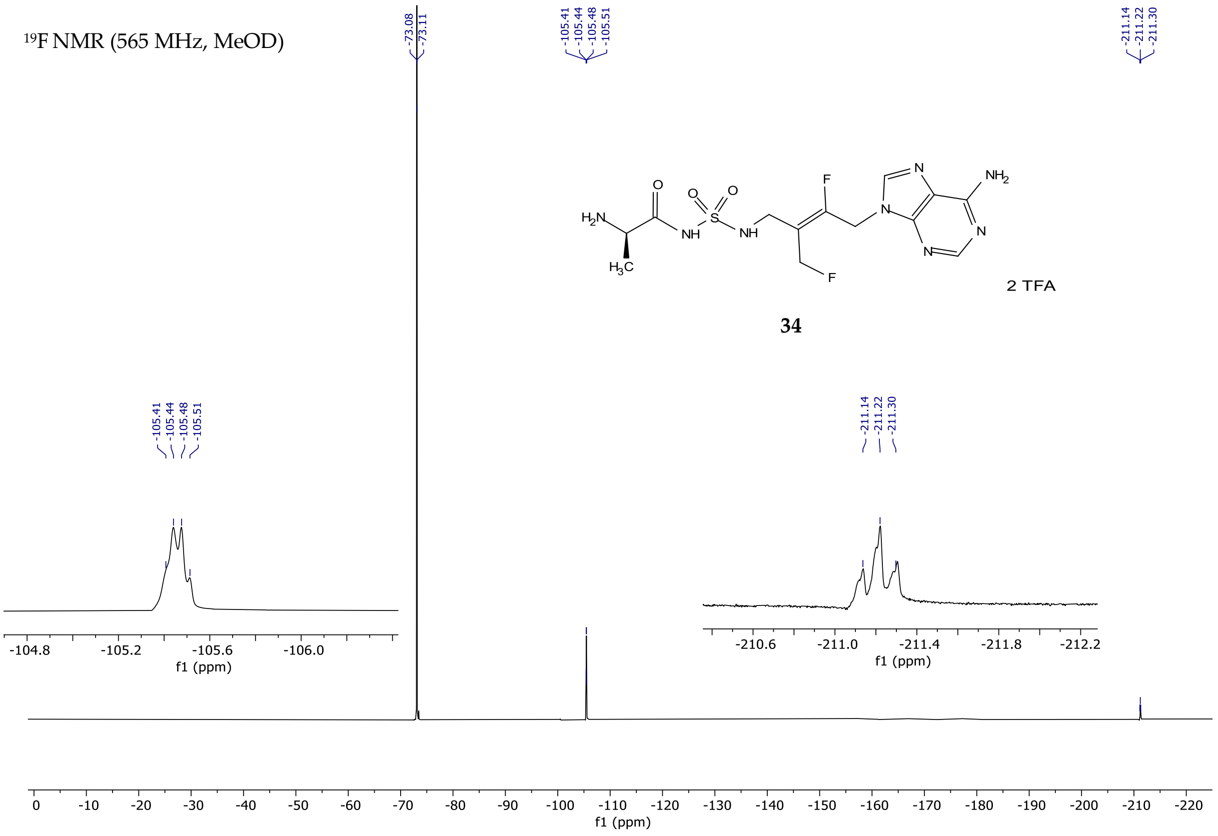

$^{13}C$ {$^{1}H$} NMR (150 MHz, MeOD)

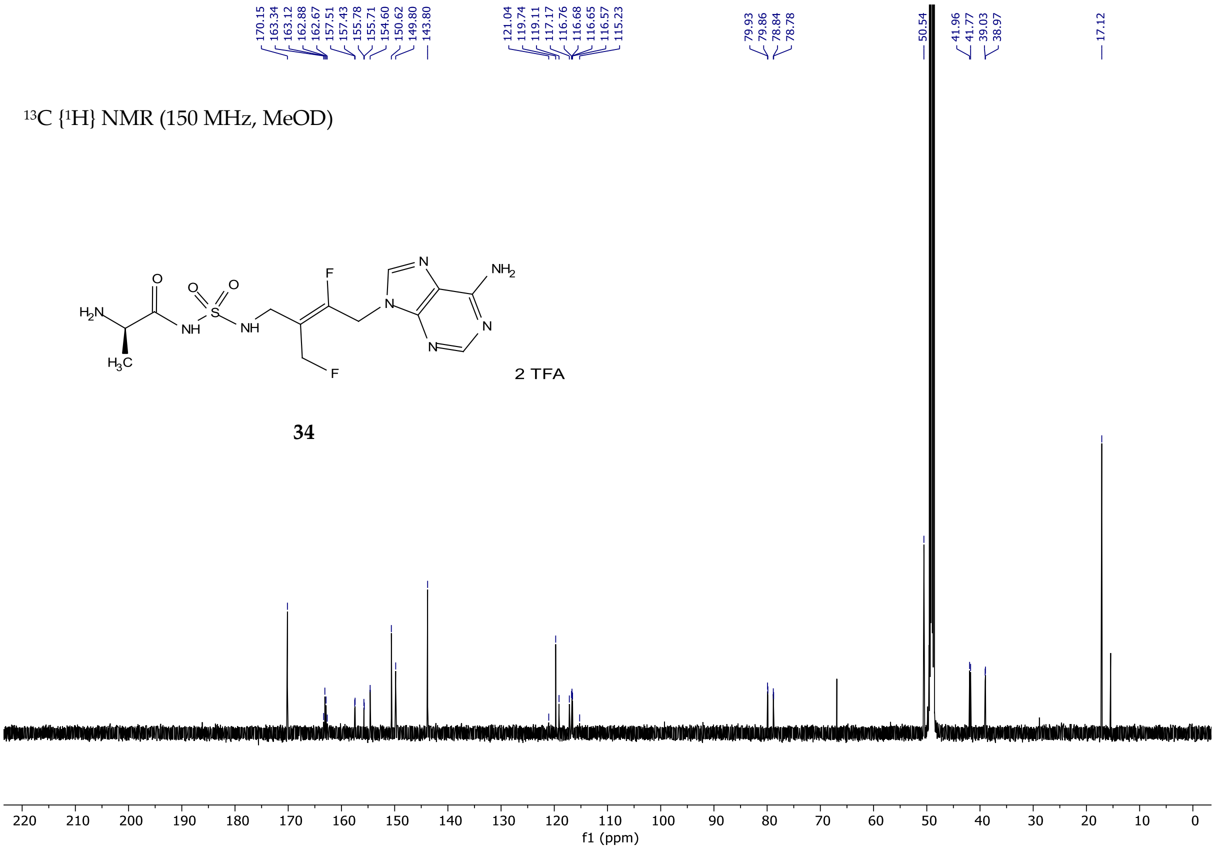

PDF Complete
Your complimentary use period has ended. Thank you for using PDF Complete.
Click Here to upgrade to Unlimited Pages and Expanded Features

rapport CC

## INFORMATION

| | | | |
|---|---|---|---|
| Sample Name: | DL694CYL | Acquired By: | System |
| Sample Type: | Unknown | Sample Set Name: | G |
| Vial: | 59 | Acq. Method Set: | Pureté HPLC |
| Injection #: | 1 | Processing Method: | 3 |
| Injection Volume: | 10,00 ul | Channel Name: | 254,0nm |
| Run Time: | 30,0 Minutes | Proc. Chnl. Descr.: | PDA 254,0 nm |
| Date Acquired: | 31/05/2023 09:40:41 CET | | |
| Date Processed: | 31/05/2023 10:15:52 CET | | |

Spectrum Index Plot

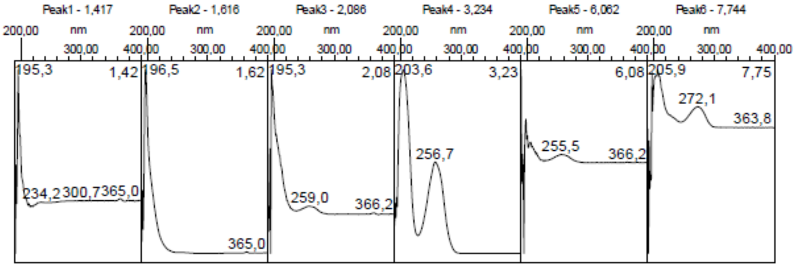


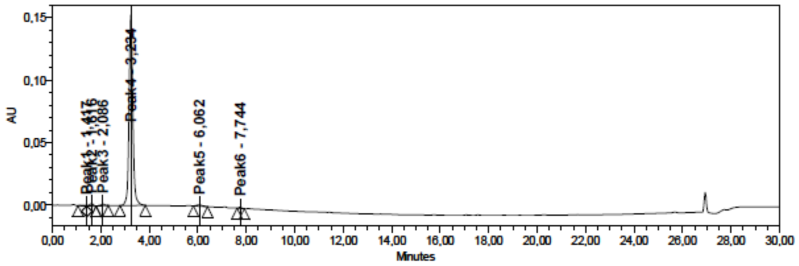


SampleName: DL694CYL; Vial: 59; Injection: 1; Date Acquired: 31/05/2023 09:40:41 CET

Peak Results

| | Name | RT | Area | Amount | Units |
|---|---|---|---|---|---|
| 1 | Peak1 | 1,417 | 9024 | | |
| 2 | Peak2 | 1,616 | 21824 | | |
| 3 | Peak3 | 2,086 | 16397 | | |
| 4 | Peak4 | 3,234 | 1640781 | | |



PDF Complete
Your complimentary use period has ended. Thank you for using PDF Complete.
Click Here to upgrade to Unlimited Pages and Expanded Features

ak Results

| Area | Amount | Units |
|---|---|---|
| 16827 | | |
| 7681 | | |



HPLC analysis for compound **34**

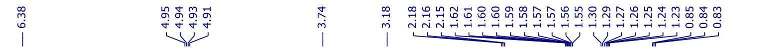

$^{1}H$ NMR (500 MHz, $CDCl_3$)

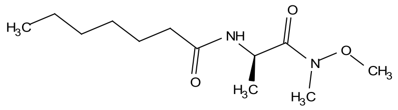

**S2**

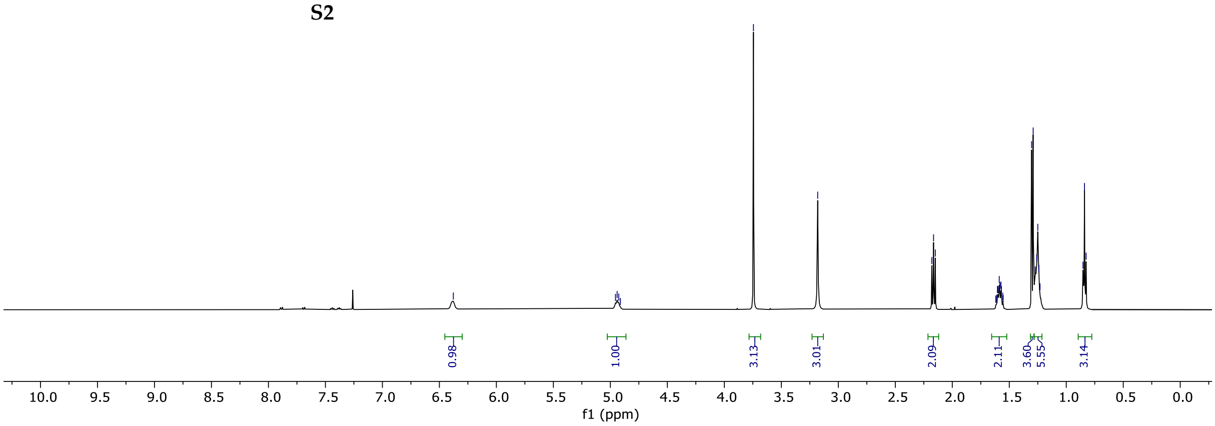

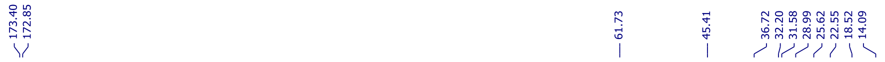

$^{13}C$ {$^{1}H$} NMR (125 MHz, $CDCl_3$)

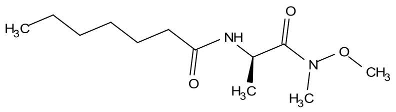

**S2**

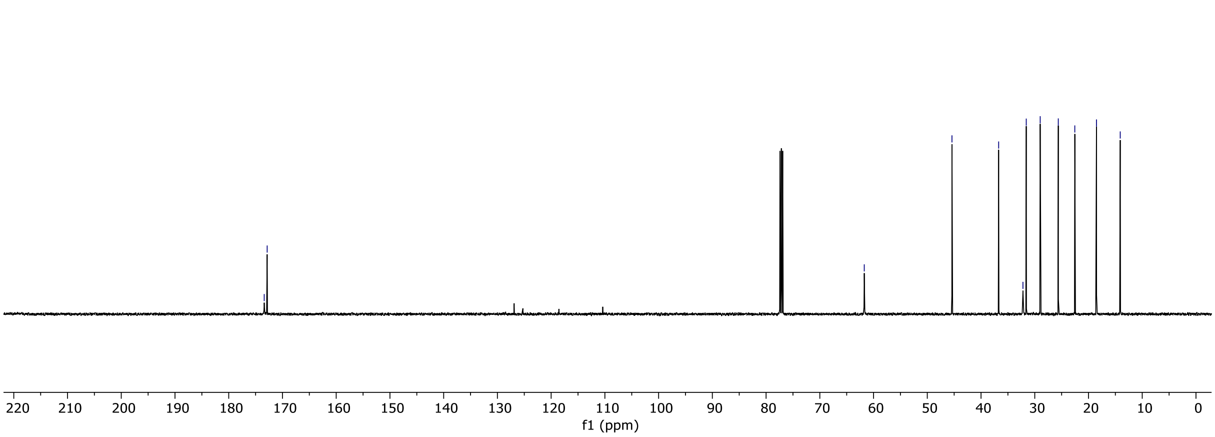

$^{1}$H NMR (500 MHz, $CDCl_3$)

**S5**

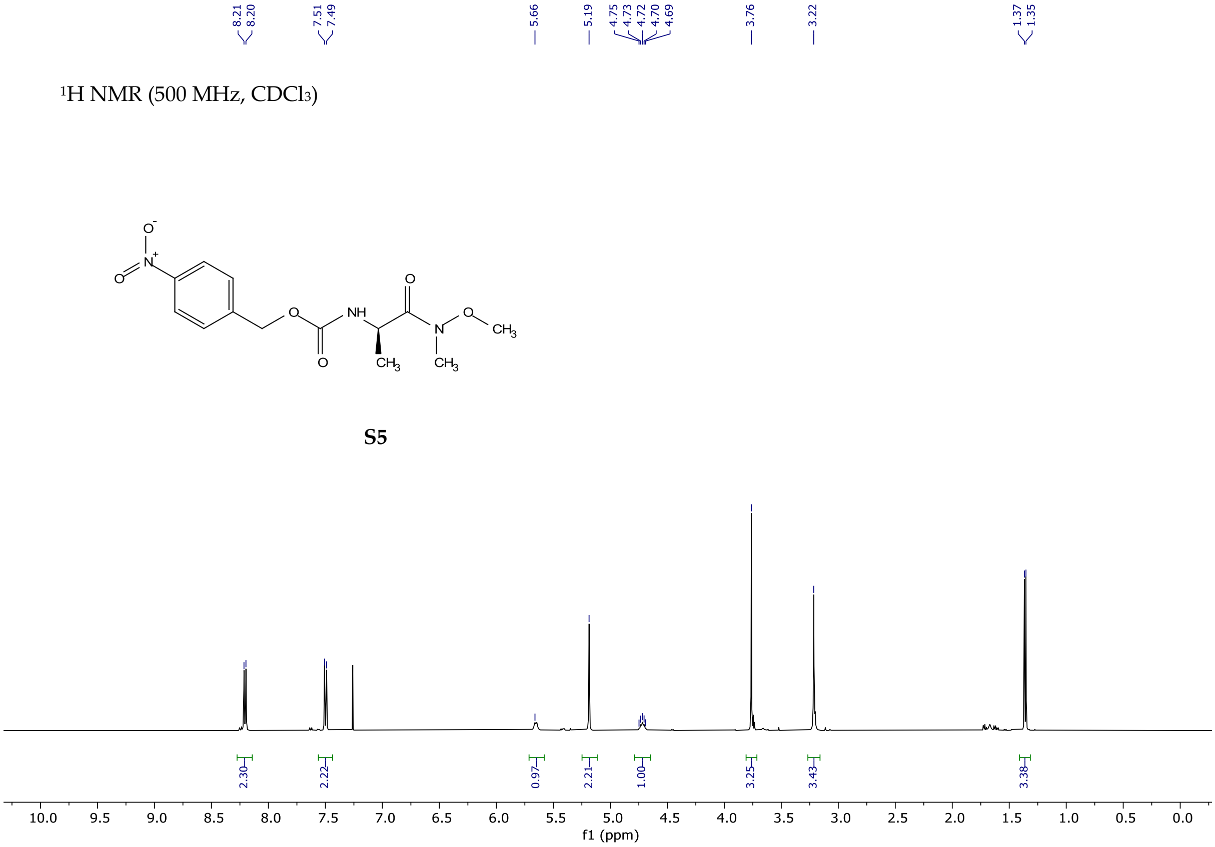


$^{13}$C {$^{1}$H} NMR (125 MHz, $CDCl_3$)

**S5**

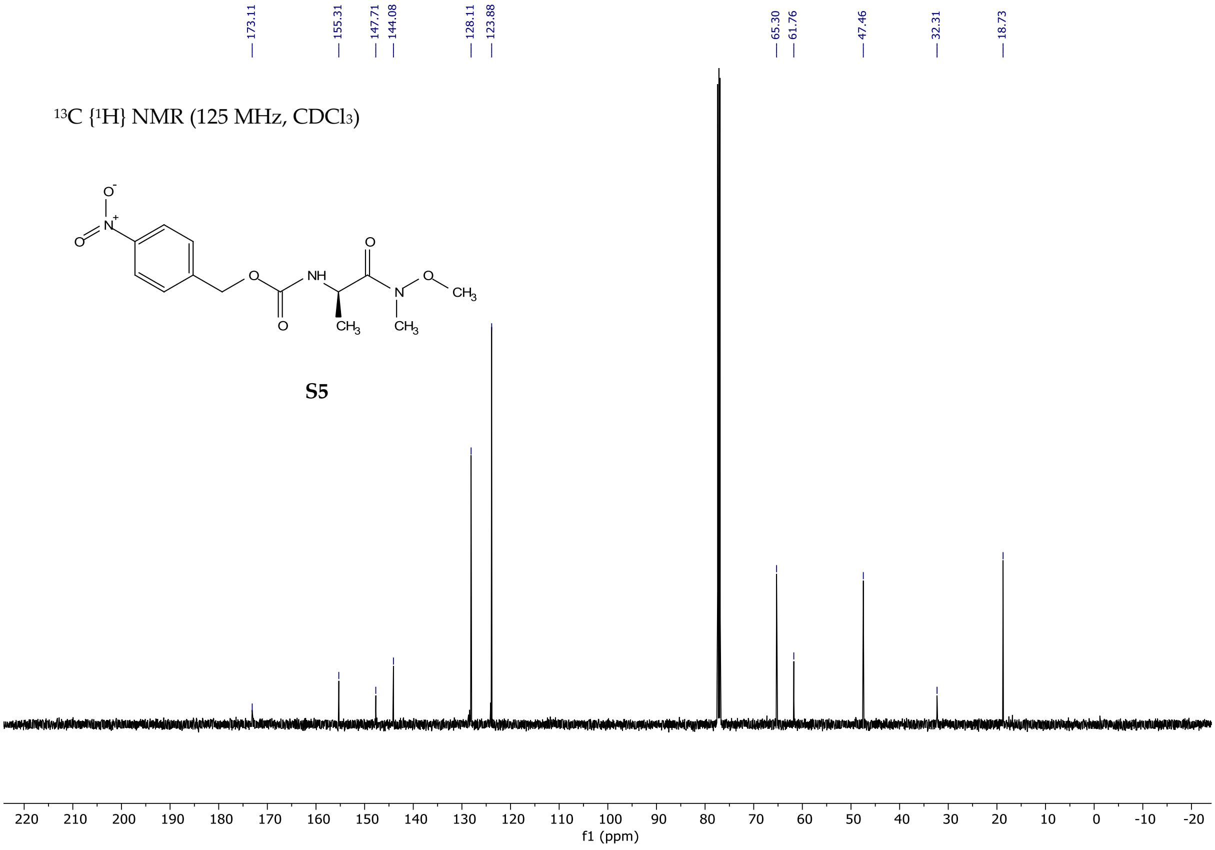

$^{1}$H NMR (500 MHz, $CDCl_3$)

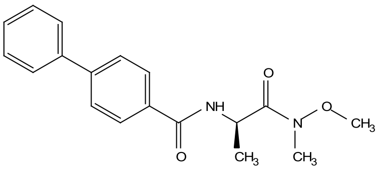


**S3**

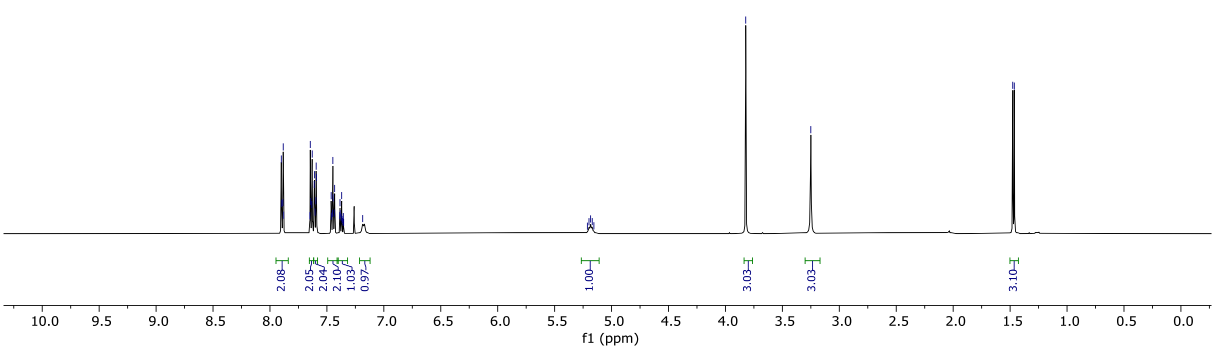


$^{13}$C {$^{1}$H} NMR (125 MHz, $CDCl_3$)

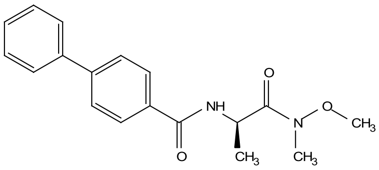


**S3**

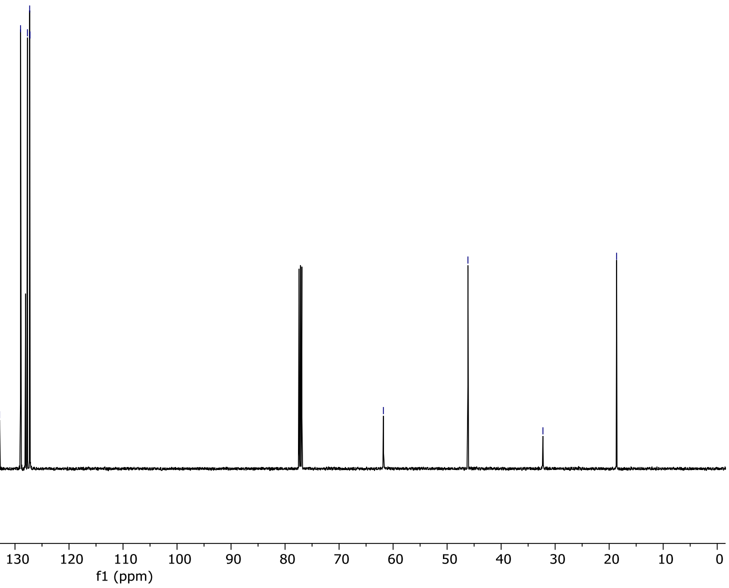

$^{1}$H NMR (500 MHz, $CDCl_3$)

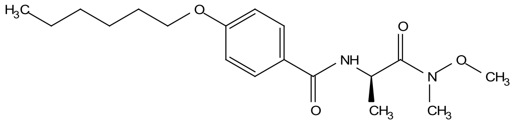


**S4**

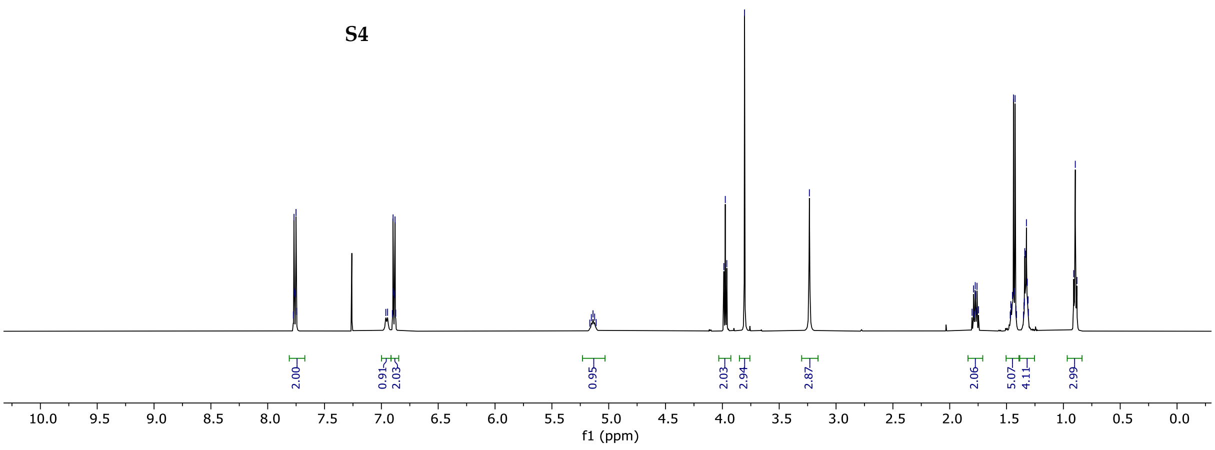


$^{13}$C {$^{1}$H} NMR (125 MHz, $CDCl_3$)

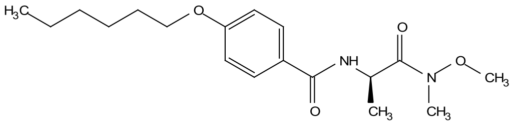


**S4**

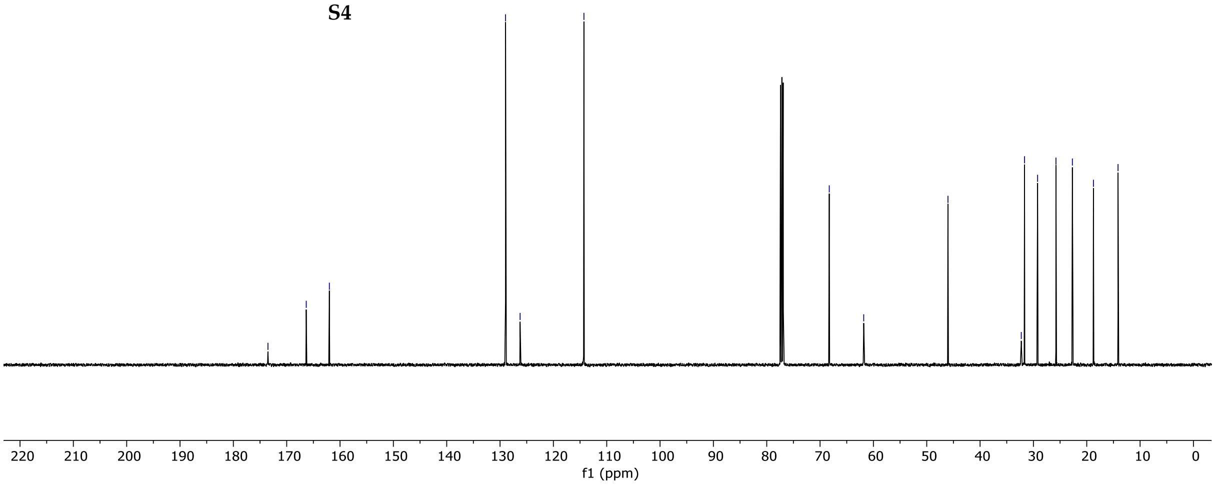

$^{1}H$ NMR (500 MHz, $CDCl_3$)

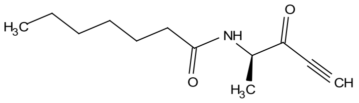


**S7**

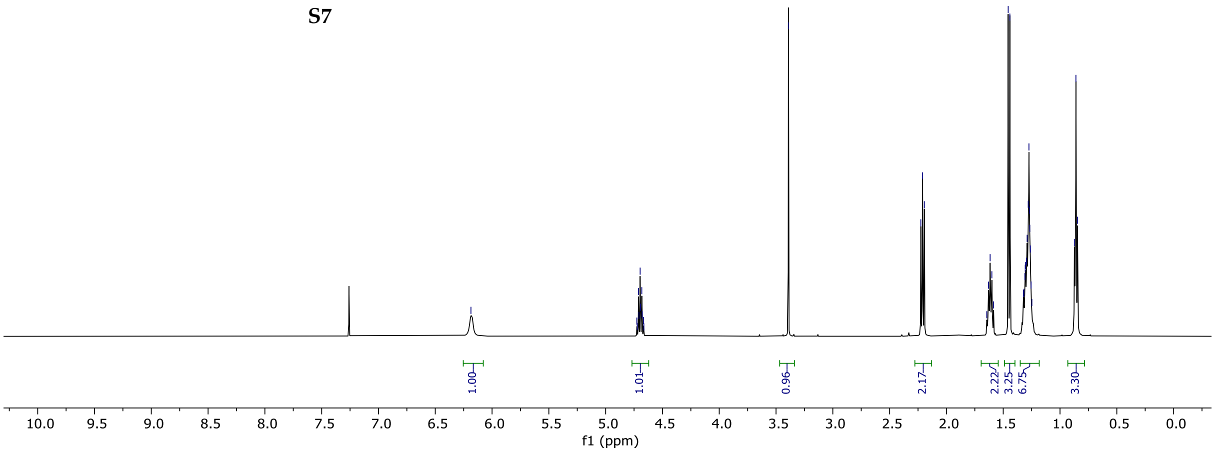


$^{13}C$ {$^{1}H$} NMR (125 MHz, $CDCl_3$)

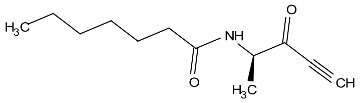


**S7**

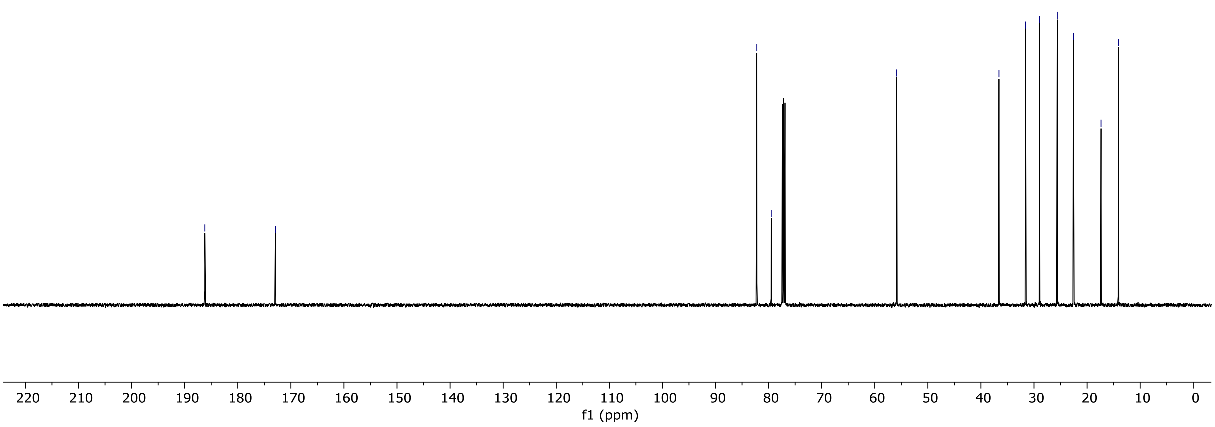

$^{1}$H NMR (500 MHz, $CDCl_3$)

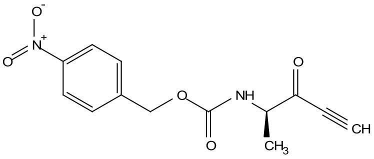

**S10**

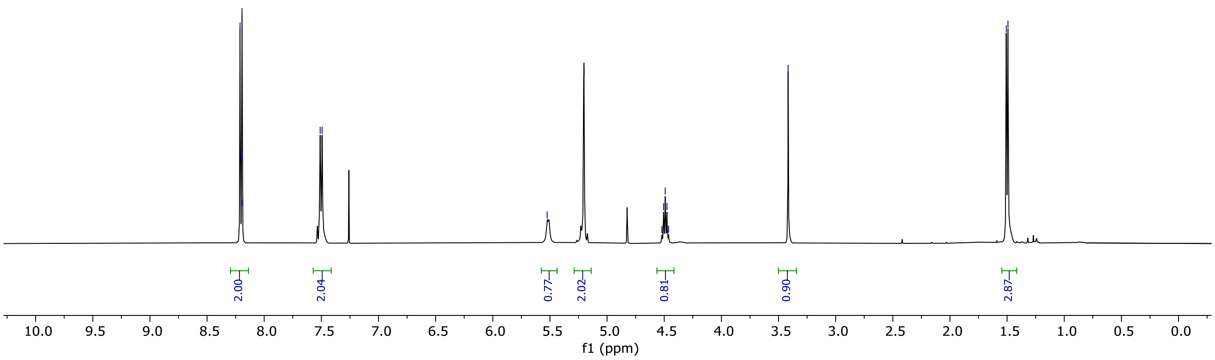


$^{13}$C {$^{1}$H} NMR (125 MHz, $CDCl_3$)

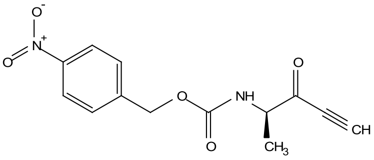

**S10**

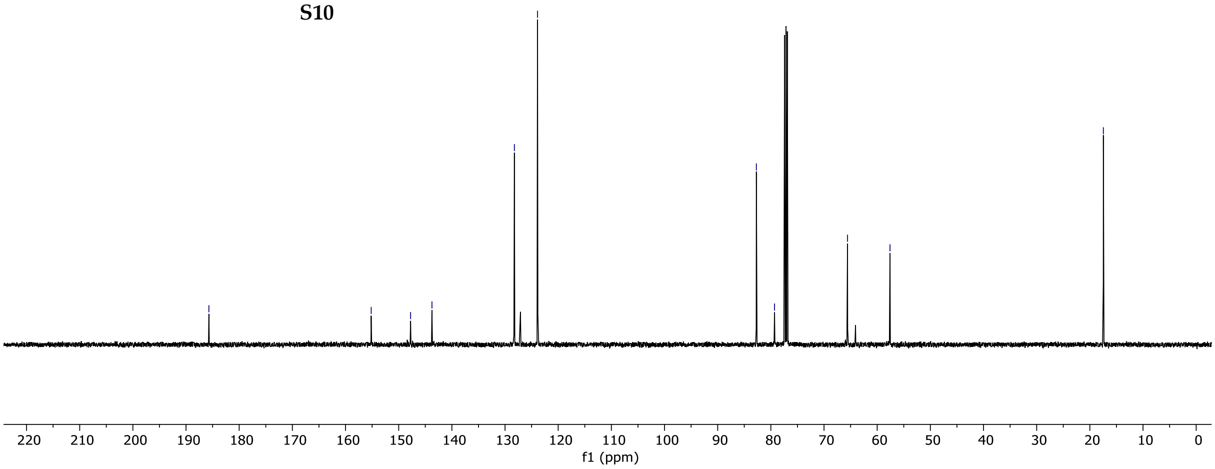

$^1H$ NMR (500 MHz, $CDCl_3$)

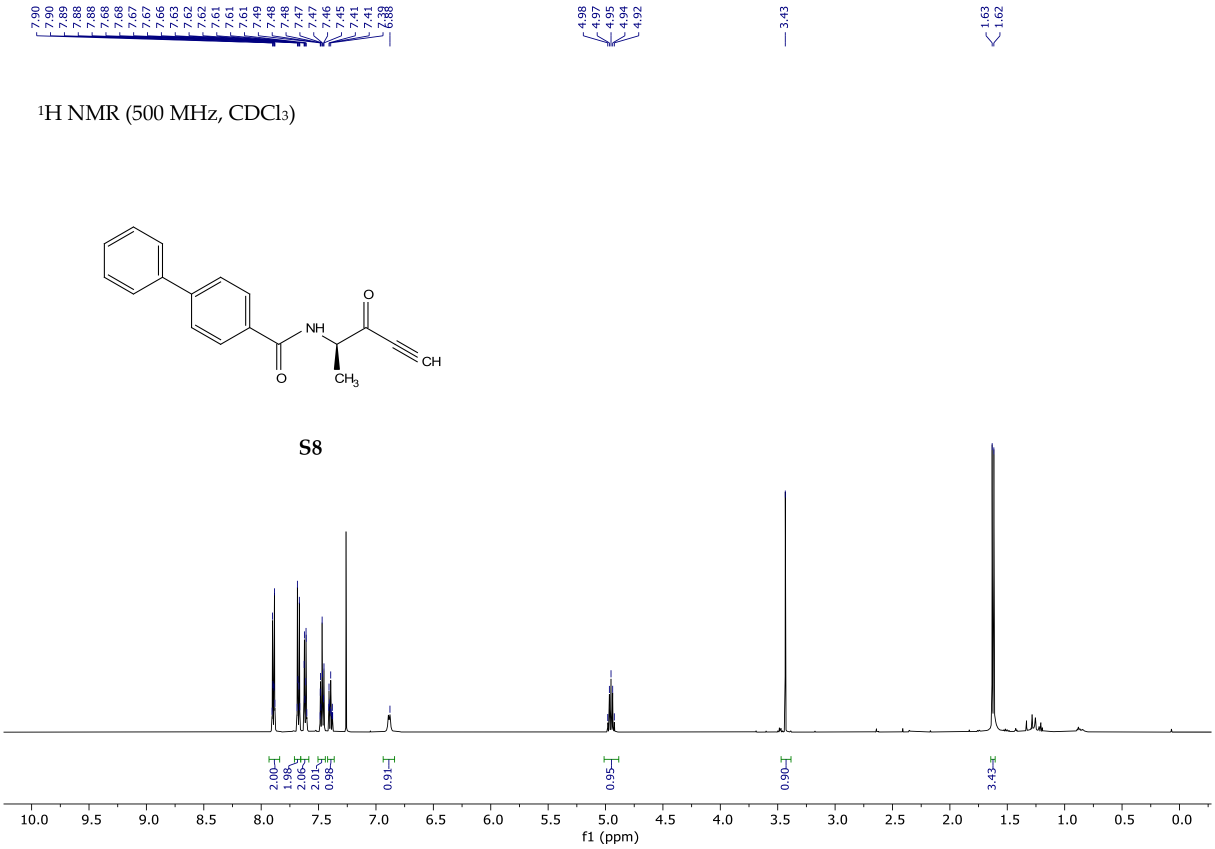


**S8**

$^{13}C$ {$^1H$} NMR (125 MHz, $CDCl_3$)

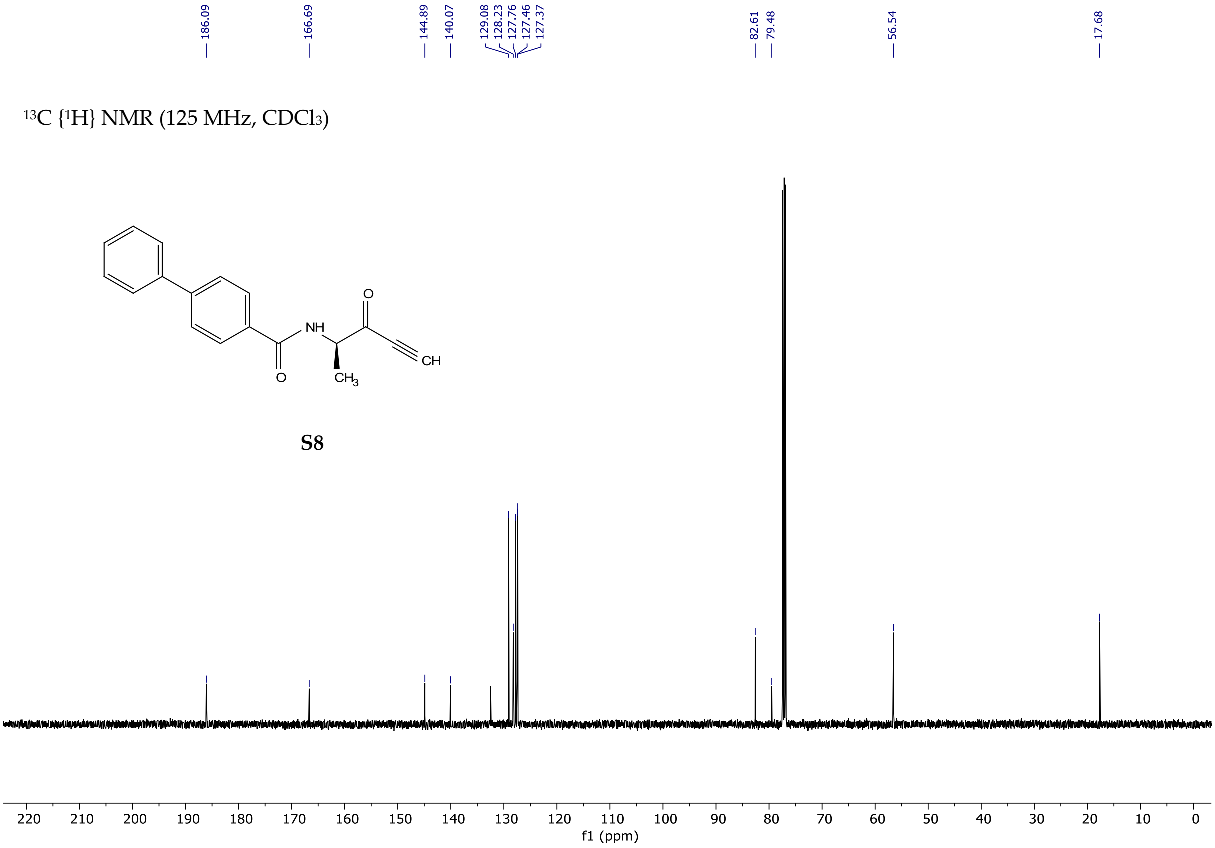


**S8**

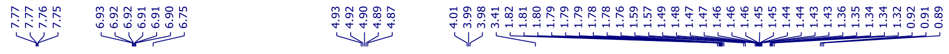

$^{1}$H NMR (500 MHz, $CDCl_3$)

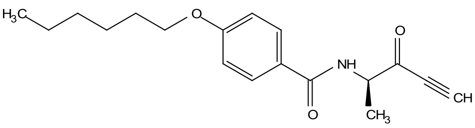

**S9**

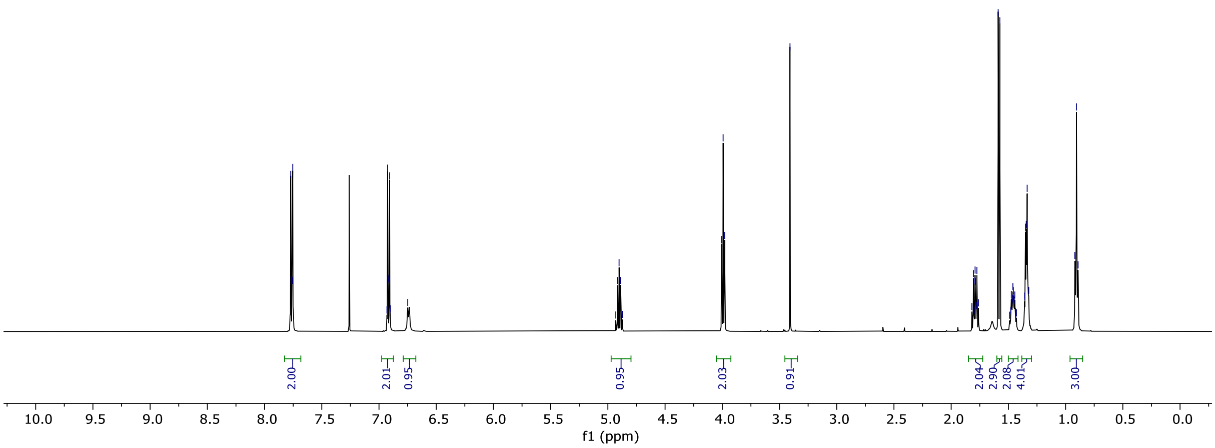

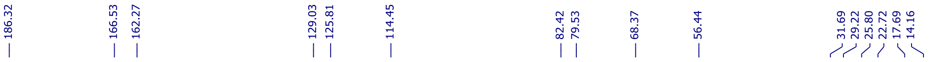

$^{13}$C {$^{1}$H} NMR (125 MHz, $CDCl_3$)

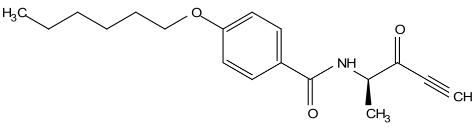

**S9**

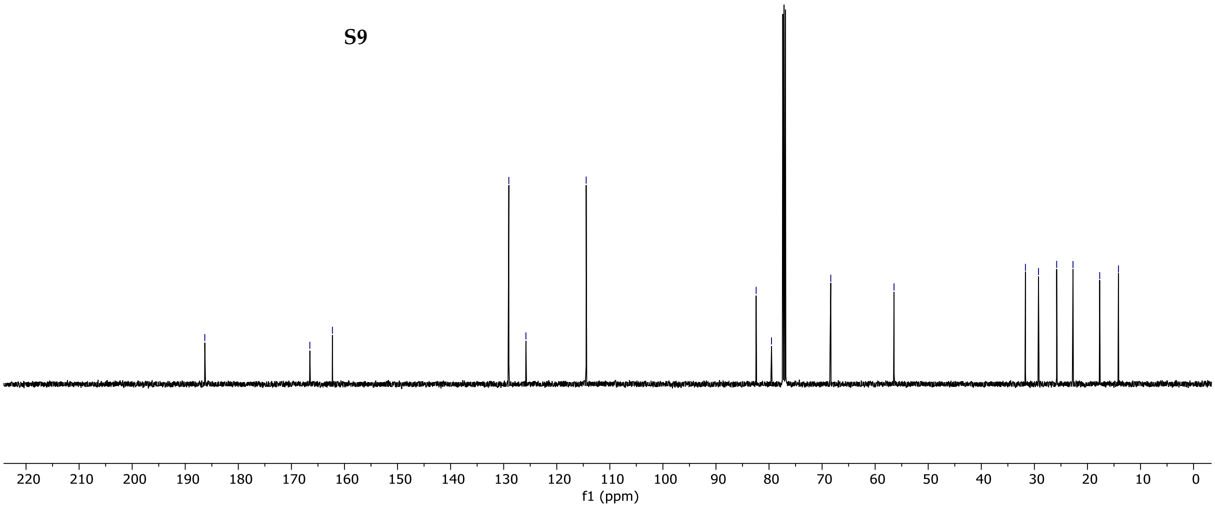

$^{1}H$ NMR (500 MHz, DMSO-d6)

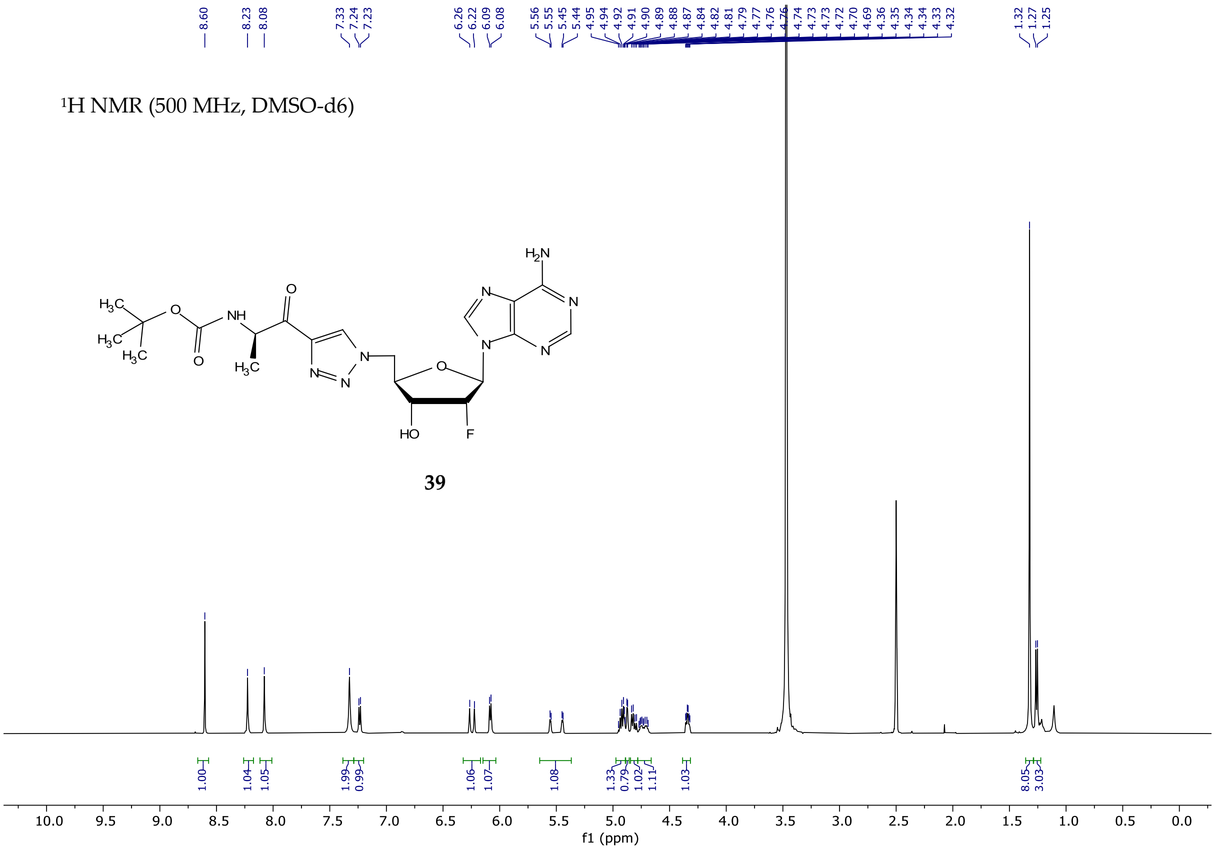


$^{19}F$ NMR (470 MHz, DMSO-d6)

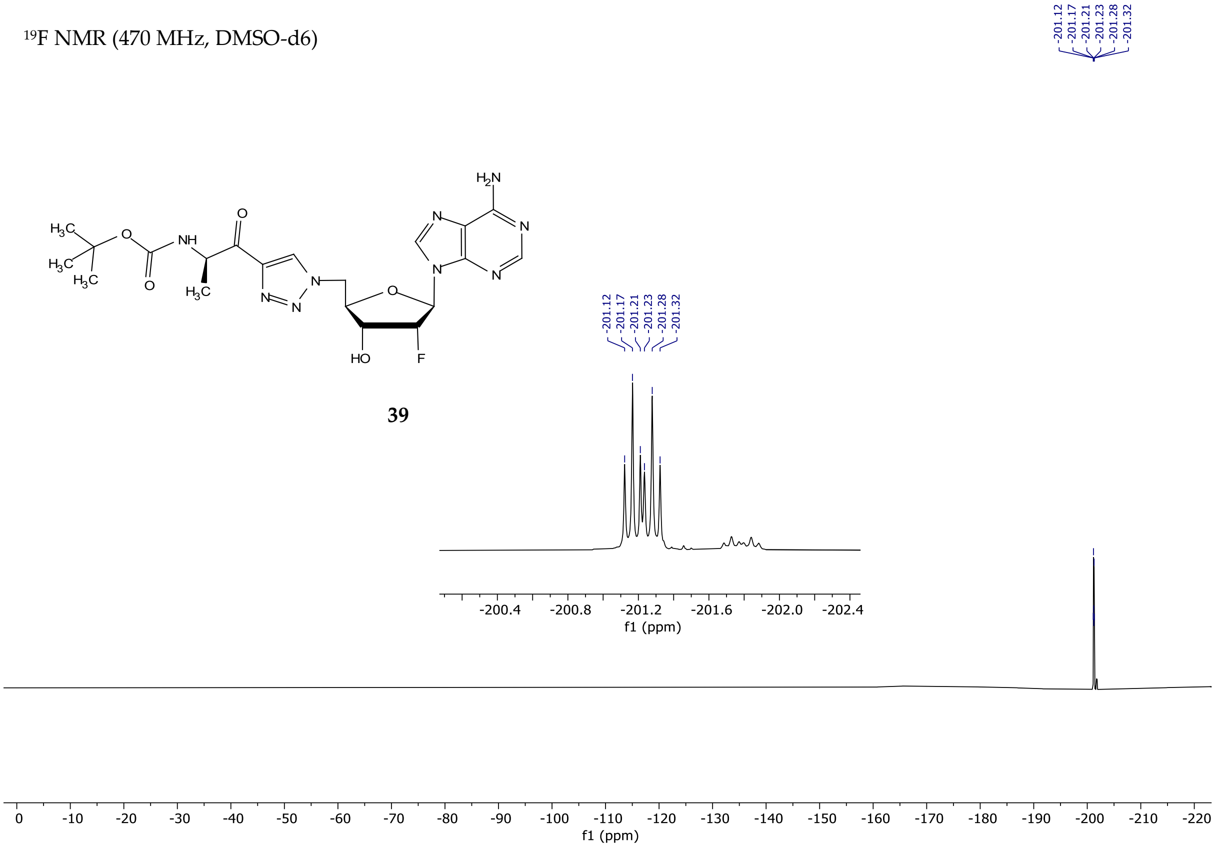

$^{13}C\ \{^{1}H\}$ NMR (125 MHz, DMSO-d6)

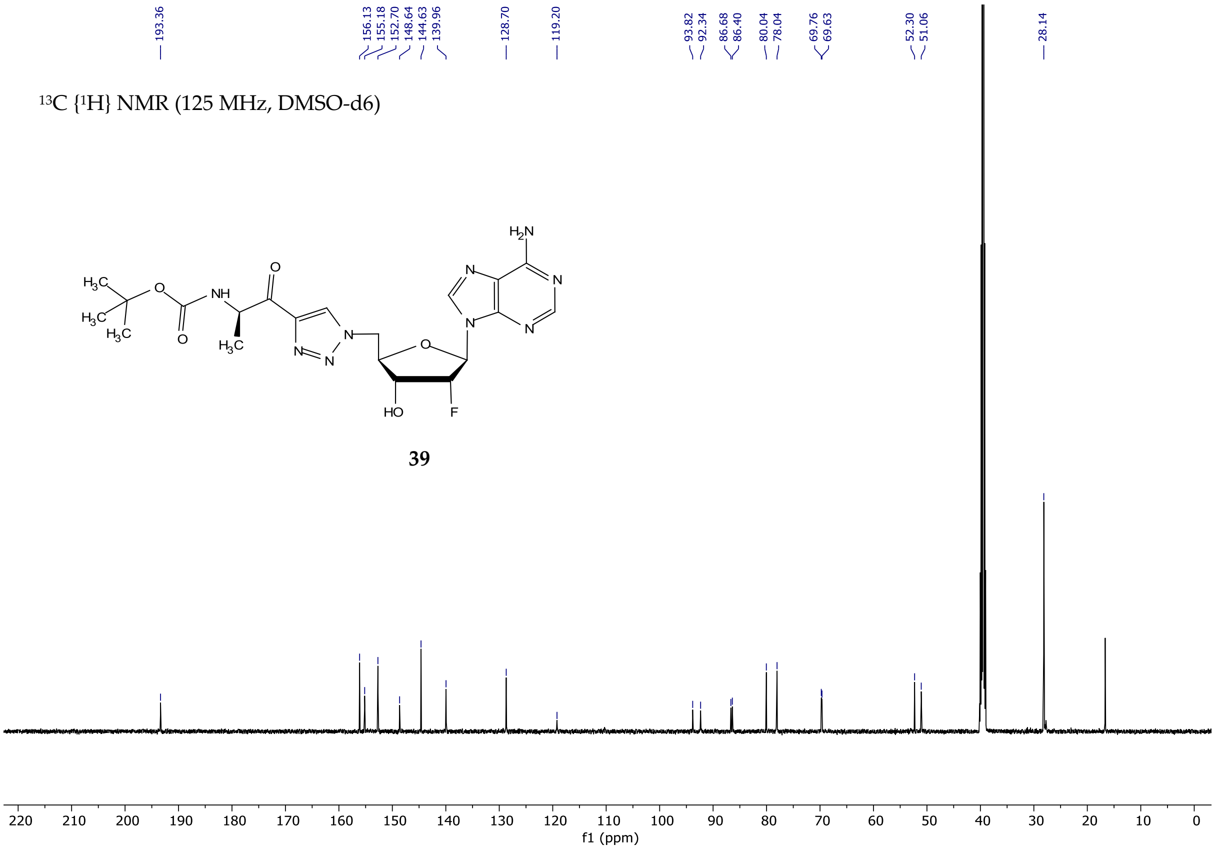


**39**

$^1H$ NMR (500 MHz, $D_2O$)

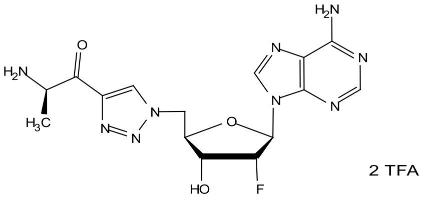

**44**

2 TFA

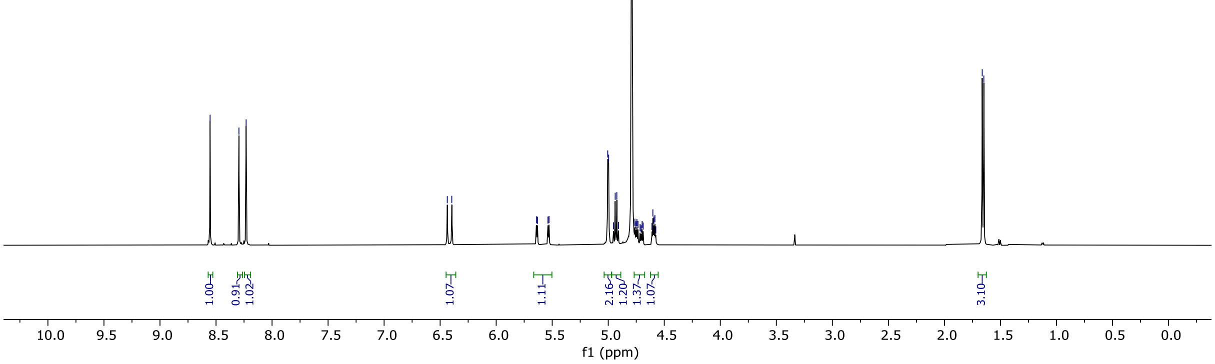


$^{19}F$ NMR (470 MHz, $D_2O$)

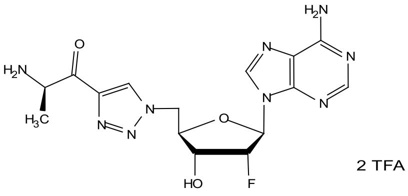

2 TFA

**44**

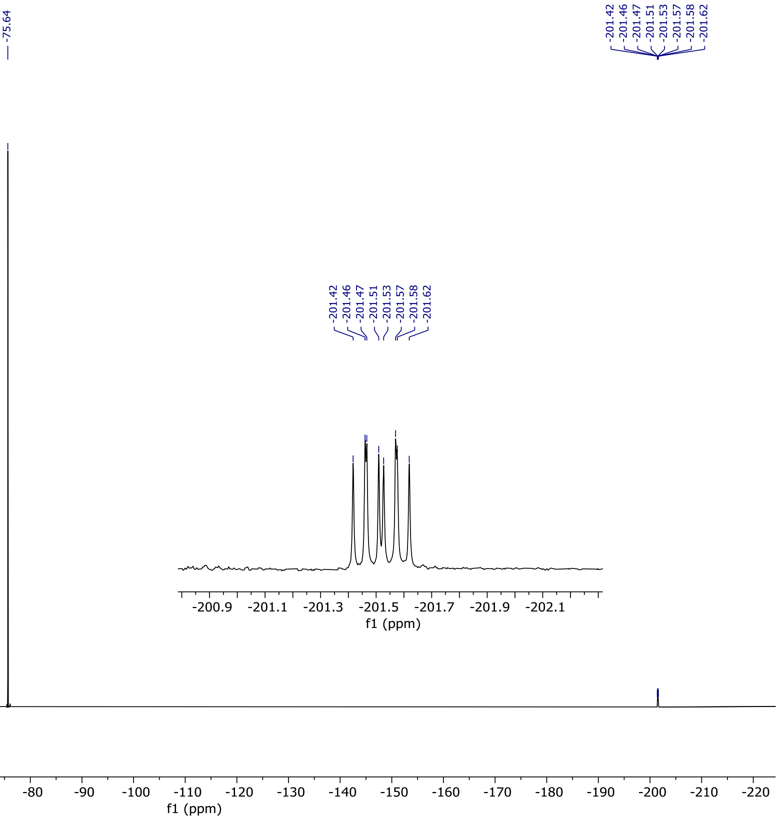

$^{13}C$ {$^{1}H$} NMR (150 MHz, $D_2O$)

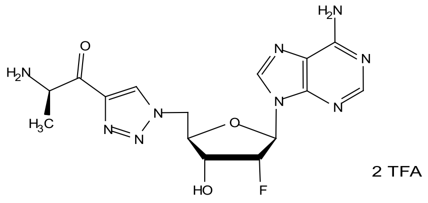


**44**

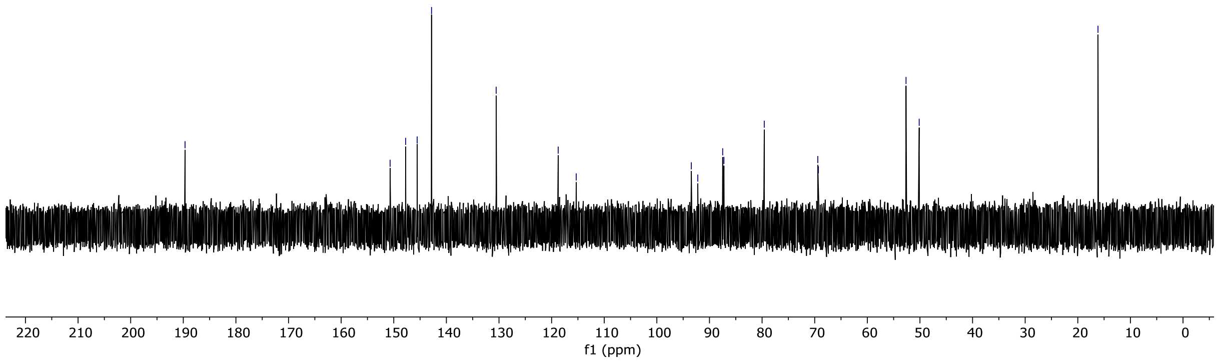

RAPPORT HPLC

Empower Node Lcmt-hplc3

# DL341A

Date Acquired 06/12/2021 11:59:56 CET

## Instrument Method: im PURETE HPLC

*Stored: 06/12/2021 11:01:47 CET*

*Revision I*

This method co

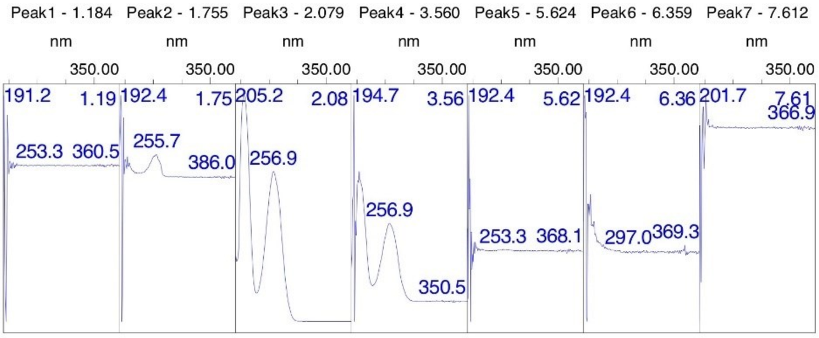


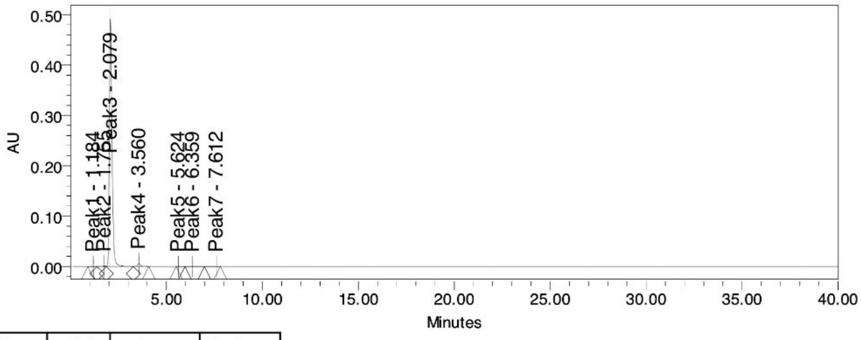


| | Peak Name | RT | Area | % Area |
|---|---|---|---|---|
| 1 | Peak1 | 1.184 | 1434 | 0.03 |
| 2 | Peak2 | 1.755 | 15083 | 0.31 |
| 3 | Peak3 | 2.079 | 4756124 | 98.26 |
| 4 | Peak4 | 3.560 | 62657 | 1.29 |

Reported by User: System
Report Method: RAPPORT HPLC
Report Method ID: 8150
Page: 1 of 2

Project Name: X-terra 2018
Date Printed:
16/05/2025
10:16:58 Europe/Paris

HPLC analysis for compound **44**

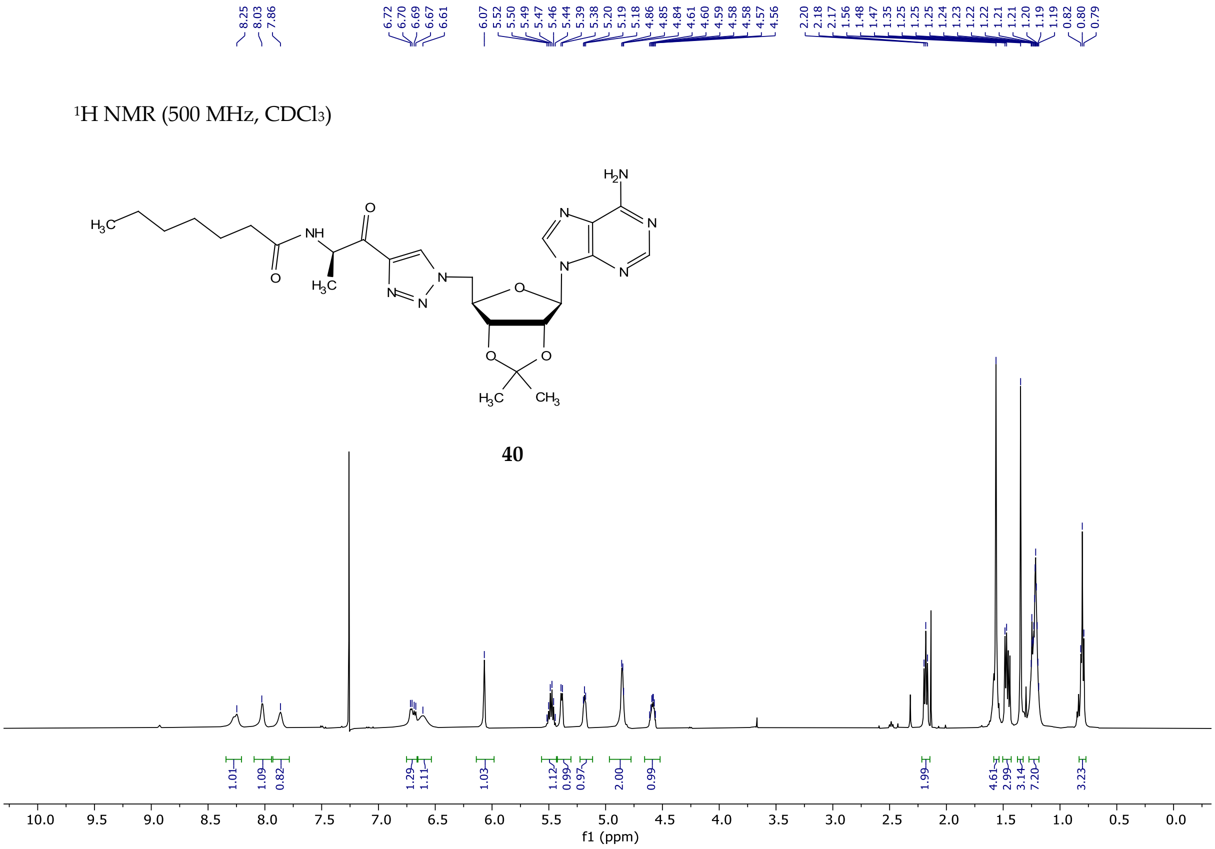
8.25 8.03 7.86 6.72 6.70 6.69 6.67 6.61 6.07 5.52 5.50 5.49 5.47 5.46 5.44 5.39 5.38 5.20 5.19 5.18 4.86 4.85 4.84 4.61 4.60 4.59 4.58 4.58 4.57 4.56 2.20 2.18 2.17 1.56 1.48 1.47 1.35 1.25 1.25 1.25 1.24 1.23 1.22 1.22 1.21 1.21 1.20 1.19 1.19 0.82 0.80 0.79
¹H NMR (500 MHz, CDCl₃)
40
1.01 1.09 0.82 1.29 1.11 1.03 1.12 0.99 0.97 2.00 0.99 1.99 4.61 2.99 3.14 7.20 3.23
f1 (ppm)

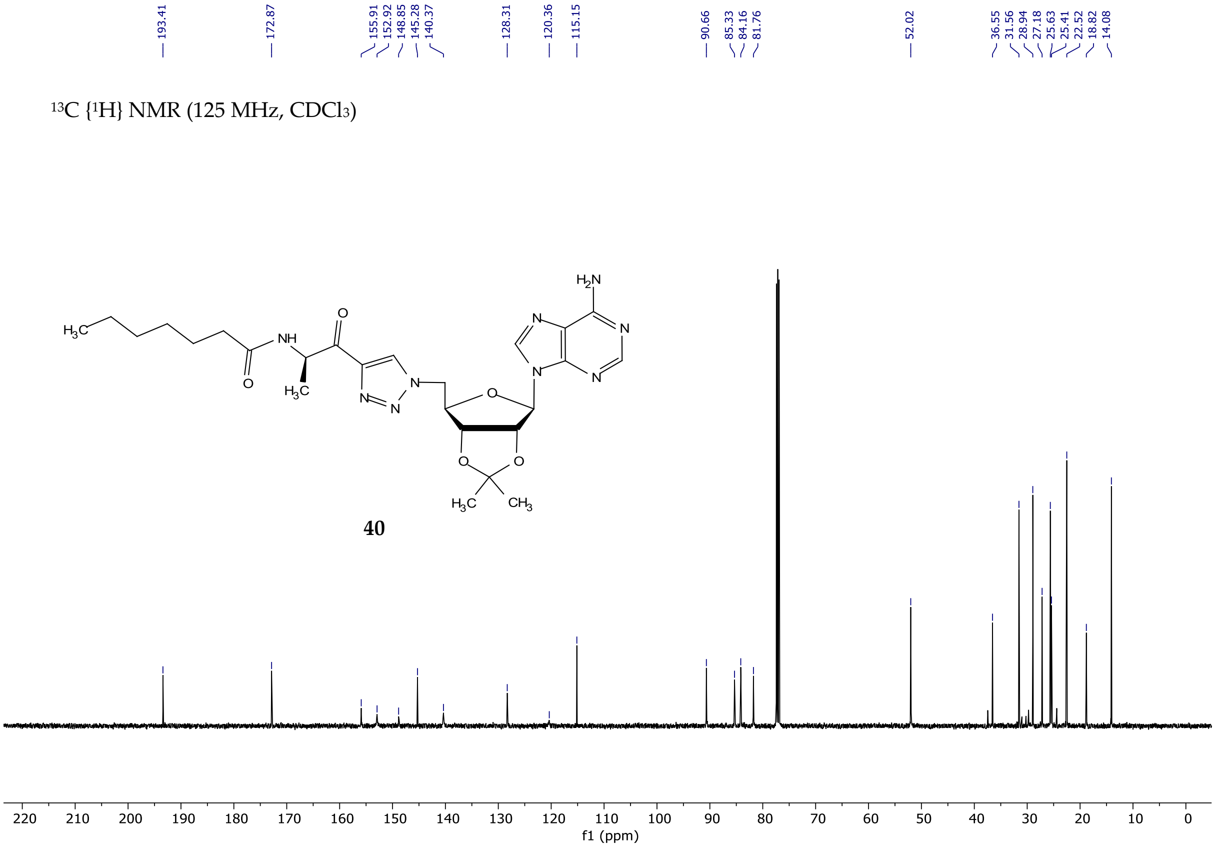
193.41 172.87 155.91 152.92 148.85 145.28 140.37 128.31 120.36 115.15 90.66 85.33 84.16 81.76 52.02 36.55 31.56 28.94 27.18 25.63 25.41 22.52 18.82 14.08
¹³C {¹H} NMR (125 MHz, CDCl₃)
40
f1 (ppm)

$^{1}$H NMR (500 MHz, DMSO-d6)

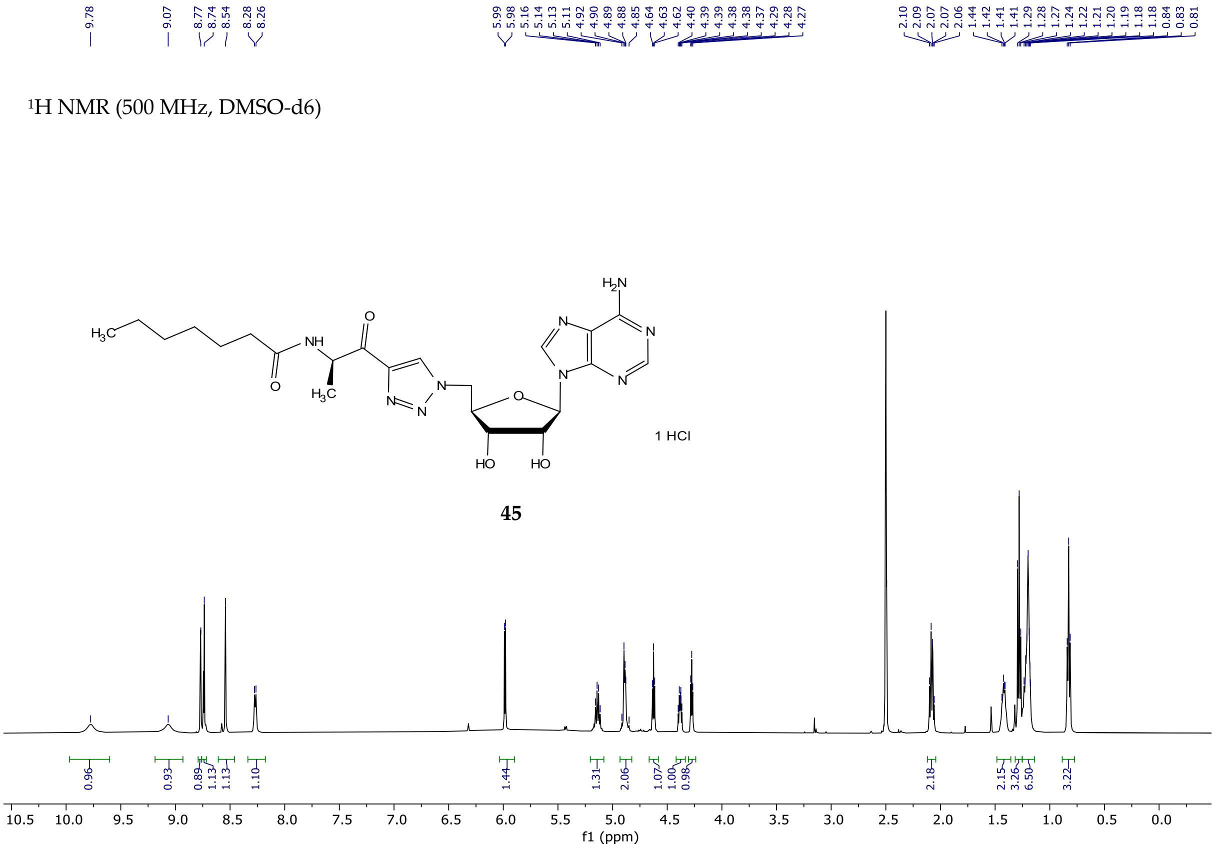


45

$^{13}$C {$^{1}$H} NMR (125 MHz, DMSO-d6)

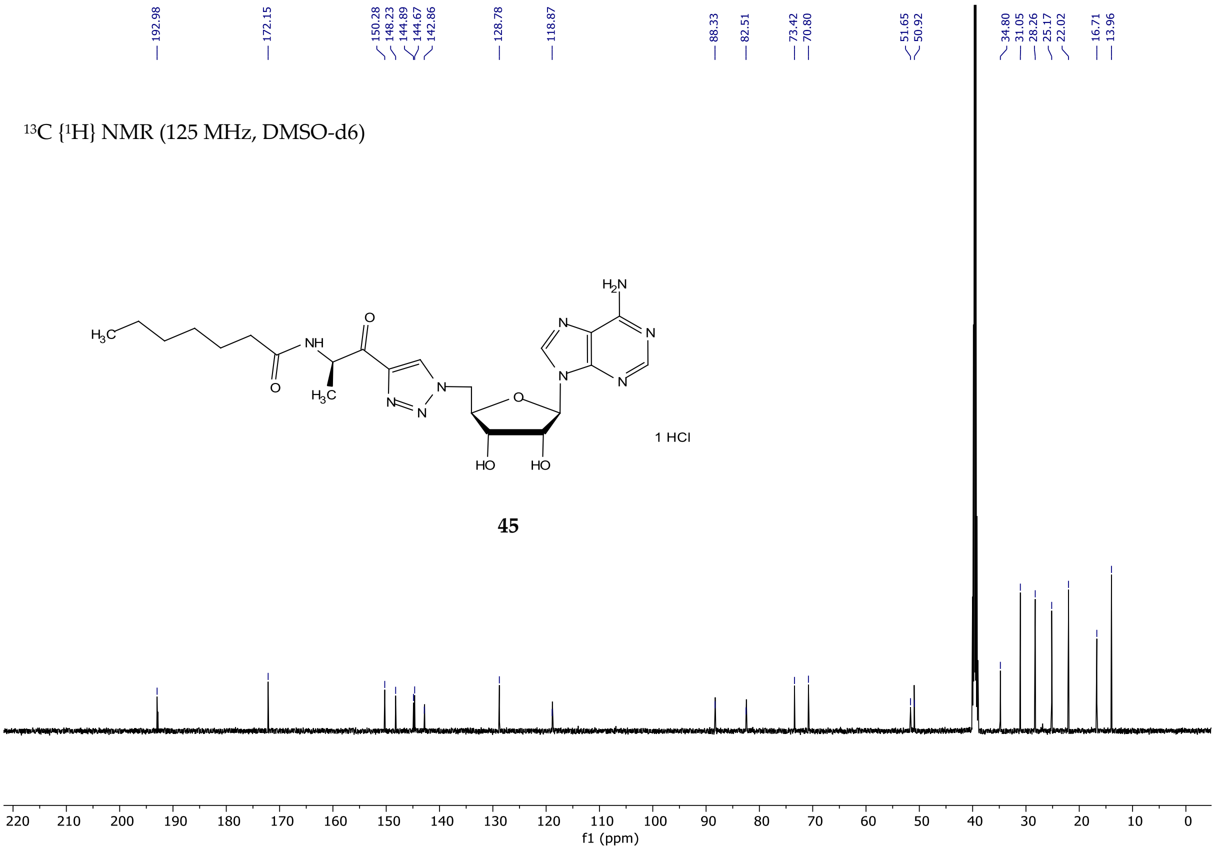


45

PDF Complete
Your complimentary use period has ended. Thank you for using PDF Complete.
Click Here to upgrade to Unlimited Pages and Expanded Features

rapport

## INFORMATION

| | | | |
|---|---|---|---|
| Sample Name: | DL493A | Acquired By: | System |
| Sample Type: | Unknown | Sample Set Name: | 3 |
| Vial: | 1 | Acq. Method Set: | Pureté HPLC |
| Injection #: | 1 | Processing Method: | 2 |
| Injection Volume: | 10,00 ul | Channel Name: | 254,0nm@3 |
| Run Time: | 30,0 Minutes | Proc. Chnl. Descr.: | PDA 254,0 nm |
| Date Acquired: | 01/09/2022 13:44:44 CET | | |
| Date Processed: | 01/09/2022 14:24:37 CET | | |

**Spectrum Index Plot**

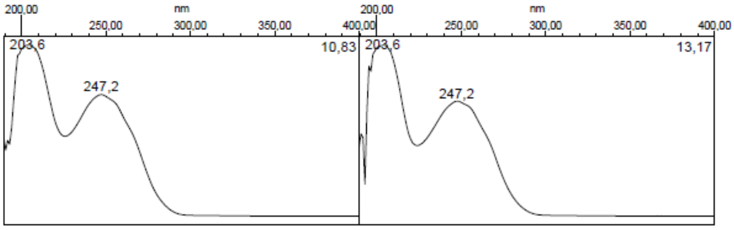


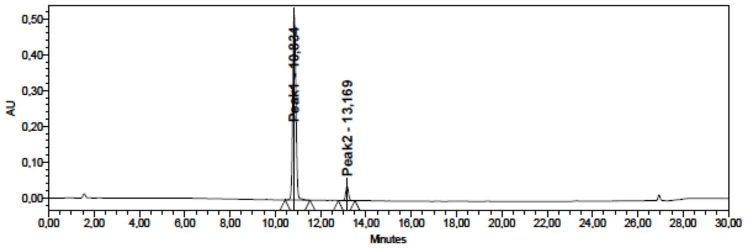


SampleName: DL493A; Vial: 1; Injection: 1; Date Acquired: 01/09/2022 13:44:44 CET

**Peak Results**

| | Name | RT | Area | % Area |
|---|---|---|---|---|
| 1 | Peak1 | 10,834 | 4999016 | 93,59 |
| 2 | Peak2 | 13,169 | 342491 | 6,41 |

Reported by User: System
Report Method: rapport
Report Method ID 5622
Page: 1 of 1

Project Name: IA-CELL1-AMY2
Date Printed:
01/09/2022
14:26:15 Europe/Paris

HPLC analysis for compound **45**

$^{1}H$ NMR (500 MHz, $CDCl_3$)

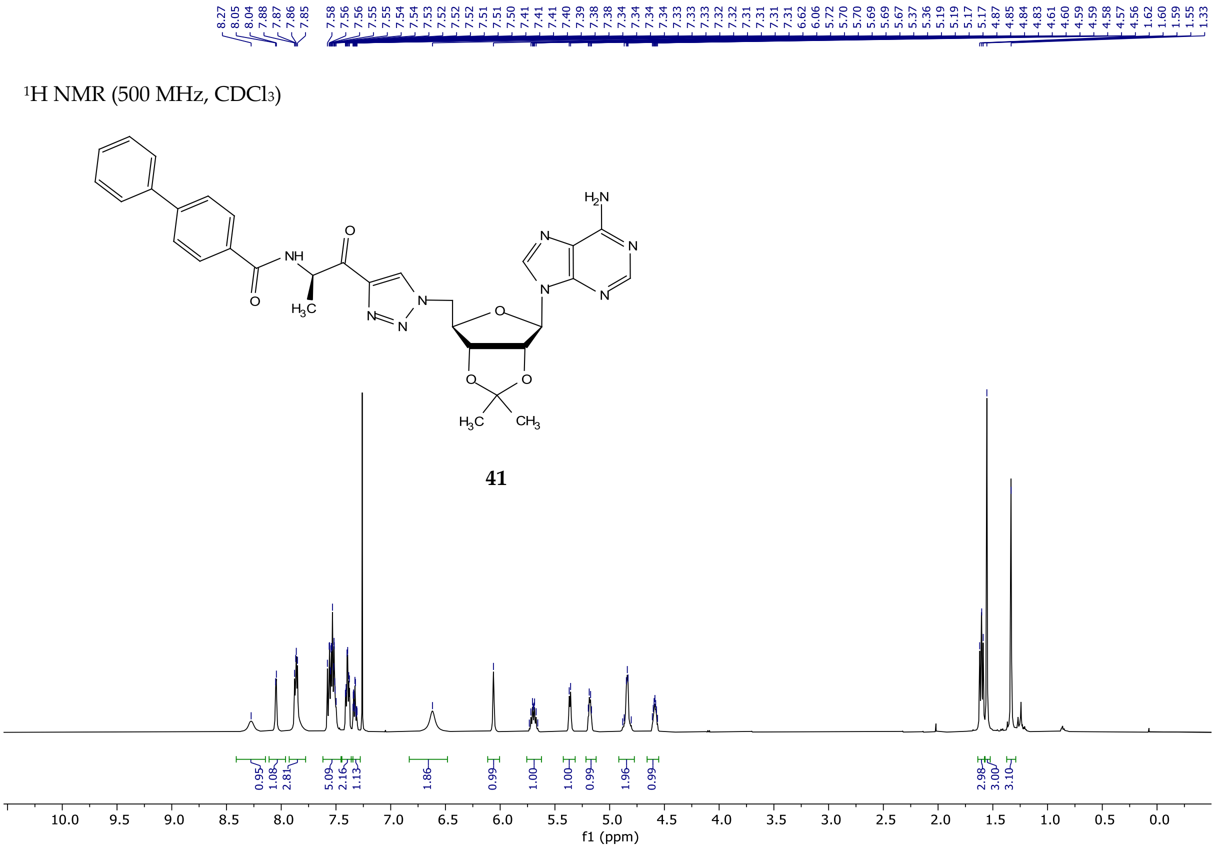


**41**

$^{13}C$ {$^{1}H$} NMR (125 MHz, $CDCl_3$)

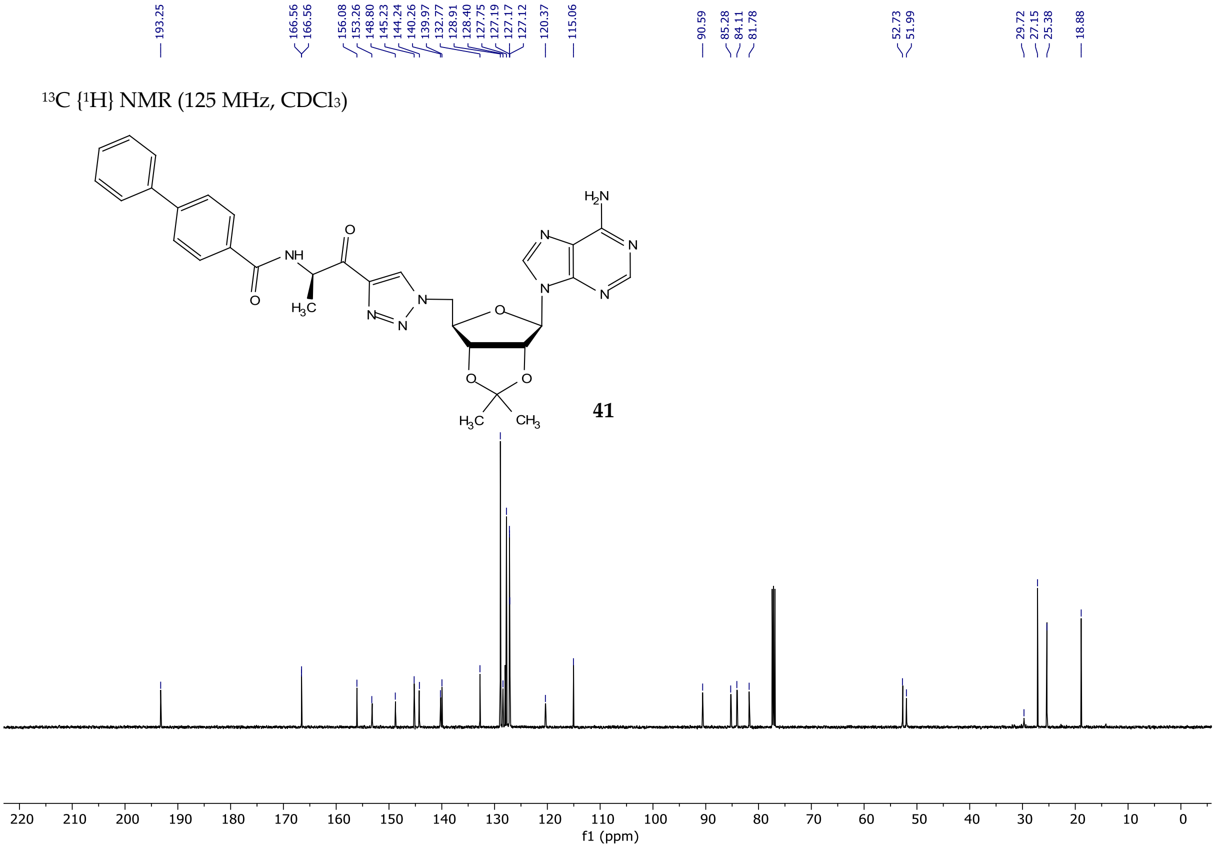


**41**

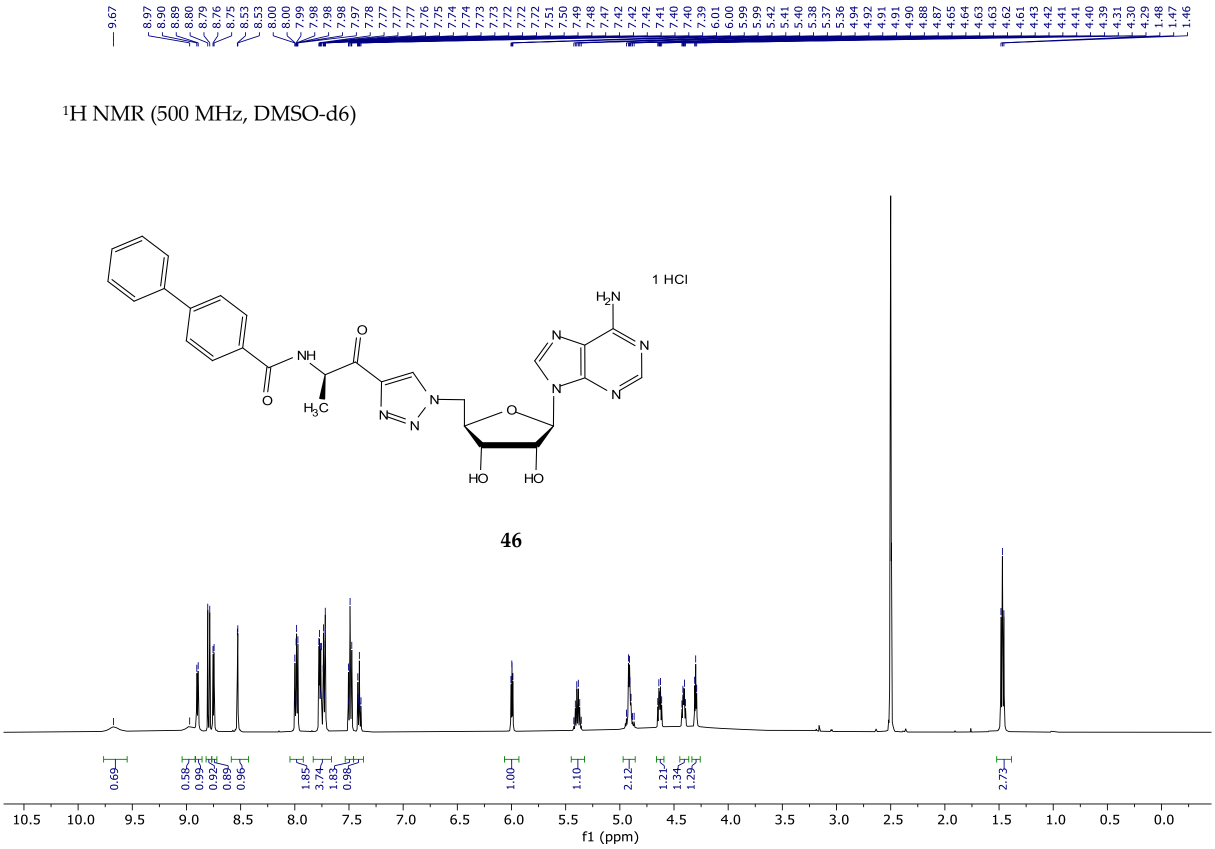

$^{1}$H NMR (500 MHz, DMSO-d6)
1 HCl
46
f1 (ppm)


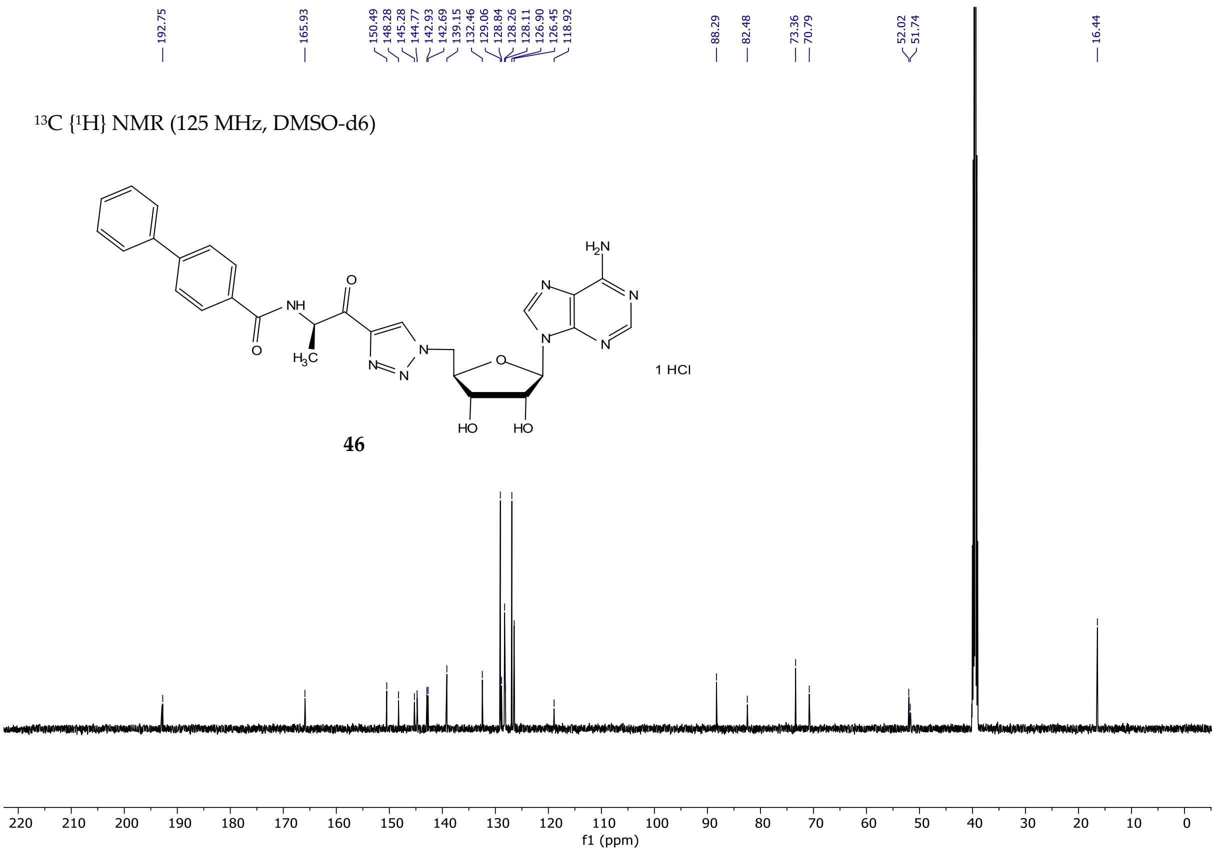

$^{13}$C {$^{1}$H} NMR (125 MHz, DMSO-d6)
1 HCl
46
f1 (ppm)

PDF Complete
Your complimentary use period has ended. Thank you for using PDF Complete.
Click Here to upgrade to Unlimited Pages and Expanded Features

rapport

INFORMATION

| | | | |
|---|---|---|---|
| Sample Name: | DL494A | Acquired By: | System |
| Sample Type: | Unknown | Sample Set Name: | F |
| Vial: | 4 | Acq. Method Set: | Pureté HPLC |
| Injection #: | 1 | Processing Method: | 2 |
| Injection Volume: | 10,00 ul | Channel Name: | 254,0nm |
| Run Time: | 30,0 Minutes | Proc. Chnl. Descr.: | PDA 254,0 nm |
| Date Acquired: | 28/07/2022 12:05:04 CET | | |
| Date Processed: | 29/07/2022 09:03:56 CET | | |

Spectrum Index Plot

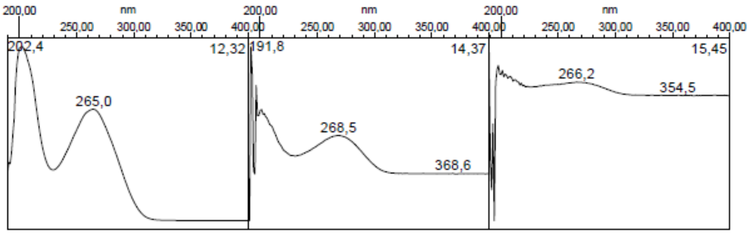


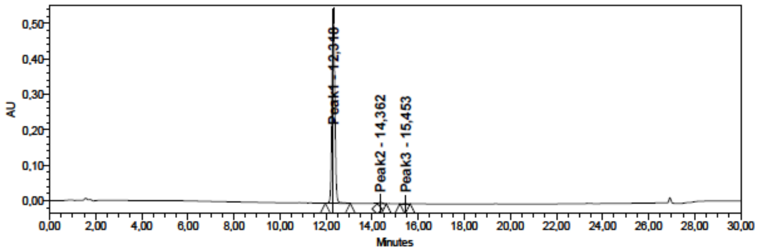


SampleName: DL494A; Vial: 4; Injection: 1; Date Acquired: 28/07/2022 12:05:04 CET

Peak Results

| | Name | RT | Area | % Area |
|---|---|---|---|---|
| 1 | Peak1 | 12,318 | 4687665 | 98,82 |
| 2 | Peak2 | 14,362 | 41626 | 0,88 |
| 3 | Peak3 | 15,453 | 14557 | 0,31 |

Reported by User: System
Report Method: rapport
Report Method ID 5622
Page: 1 of 1

Project Name: IA-CELL1-AMY2
Date Printed:
29/07/2022
09:07:08 Europe/Paris

HPLC analysis for compound **46**

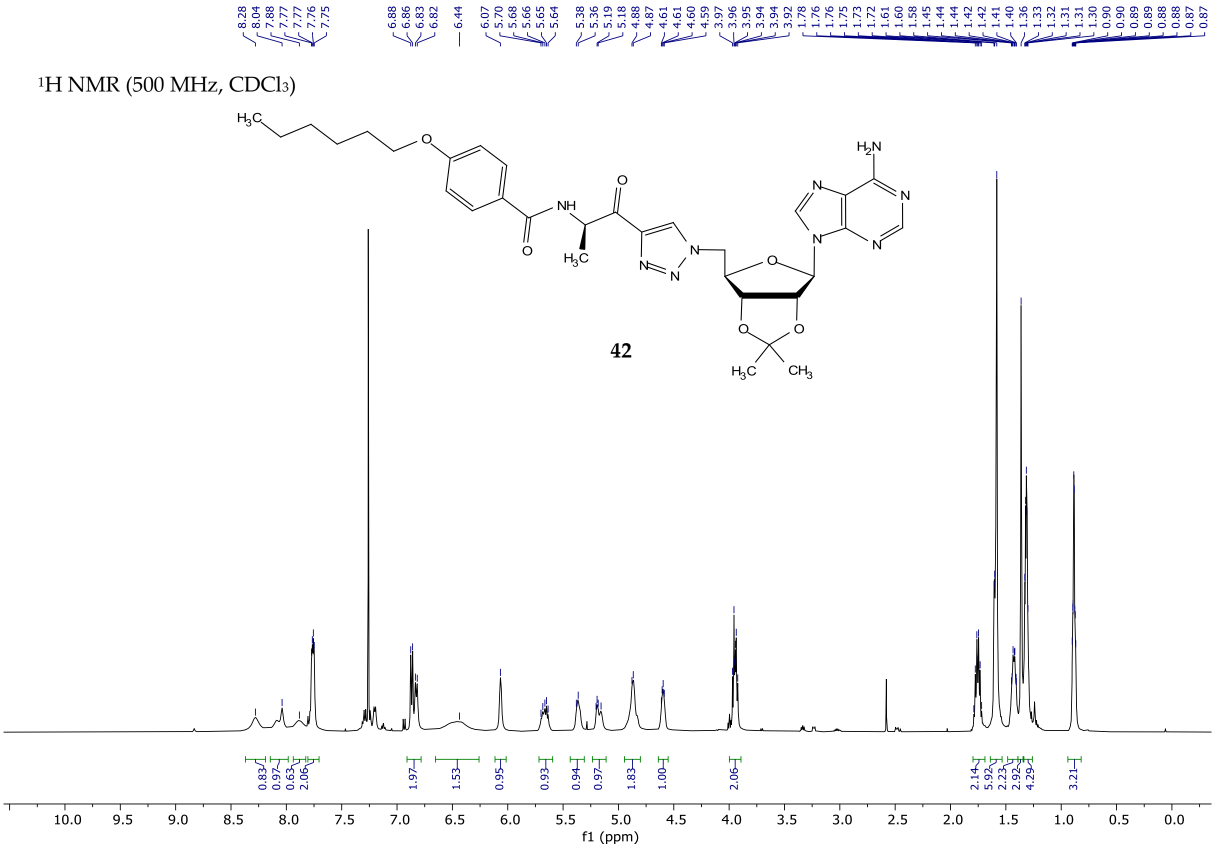
$^1$H NMR (500 MHz, $CDCl_3$)
42
f1 (ppm)

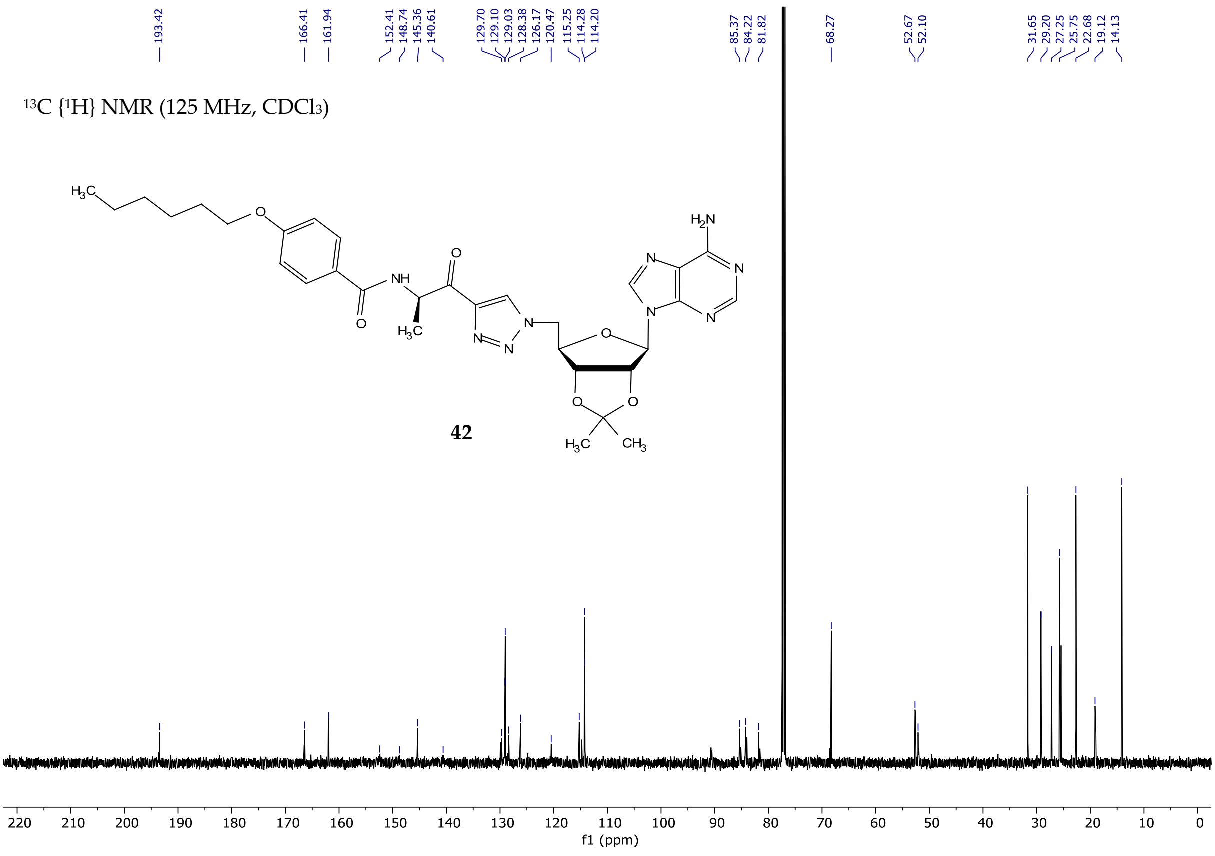
$^{13}$C {$^1$H} NMR (125 MHz, $CDCl_3$)
42
f1 (ppm)

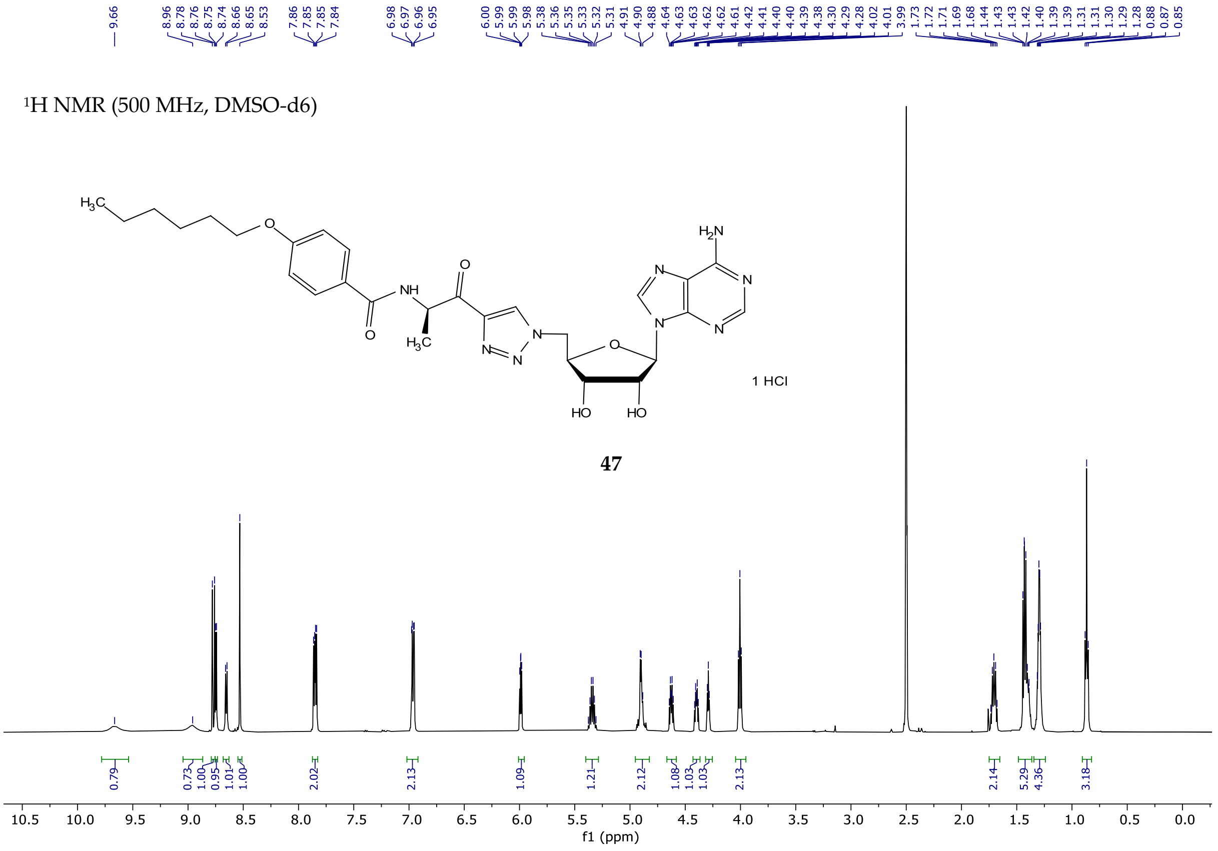
9.66 8.96 8.78 8.76 8.75 8.74 8.66 8.65 8.53 7.86 7.85 7.85 7.84 6.98 6.97 6.96 6.95 6.00 5.99 5.99 5.98 5.38 5.36 5.35 5.33 5.32 5.31 4.91 4.90 4.88 4.64 4.63 4.63 4.62 4.61 4.42 4.41 4.40 4.40 4.39 4.38 4.30 4.29 4.28 4.02 4.01 3.99 1.73 1.72 1.71 1.69 1.68 1.44 1.43 1.43 1.42 1.40 1.39 1.39 1.31 1.31 1.30 1.29 1.28 0.88 0.87 0.85
¹H NMR (500 MHz, DMSO-d6)
H3C
O
NH
O
H3C
O
N
N
N
O
HO
HO
H2N
N
N
N
N
1 HCl
47
0.79 0.73 1.00 0.95 1.01 1.00 2.02 2.13 1.09 1.21 2.12 1.08 1.03 1.03 2.13 2.14 5.29 4.36 3.18
10.5 10.0 9.5 9.0 8.5 8.0 7.5 7.0 6.5 6.0 5.5 5.0 4.5 4.0 3.5 3.0 2.5 2.0 1.5 1.0 0.5 0.0
f1 (ppm)

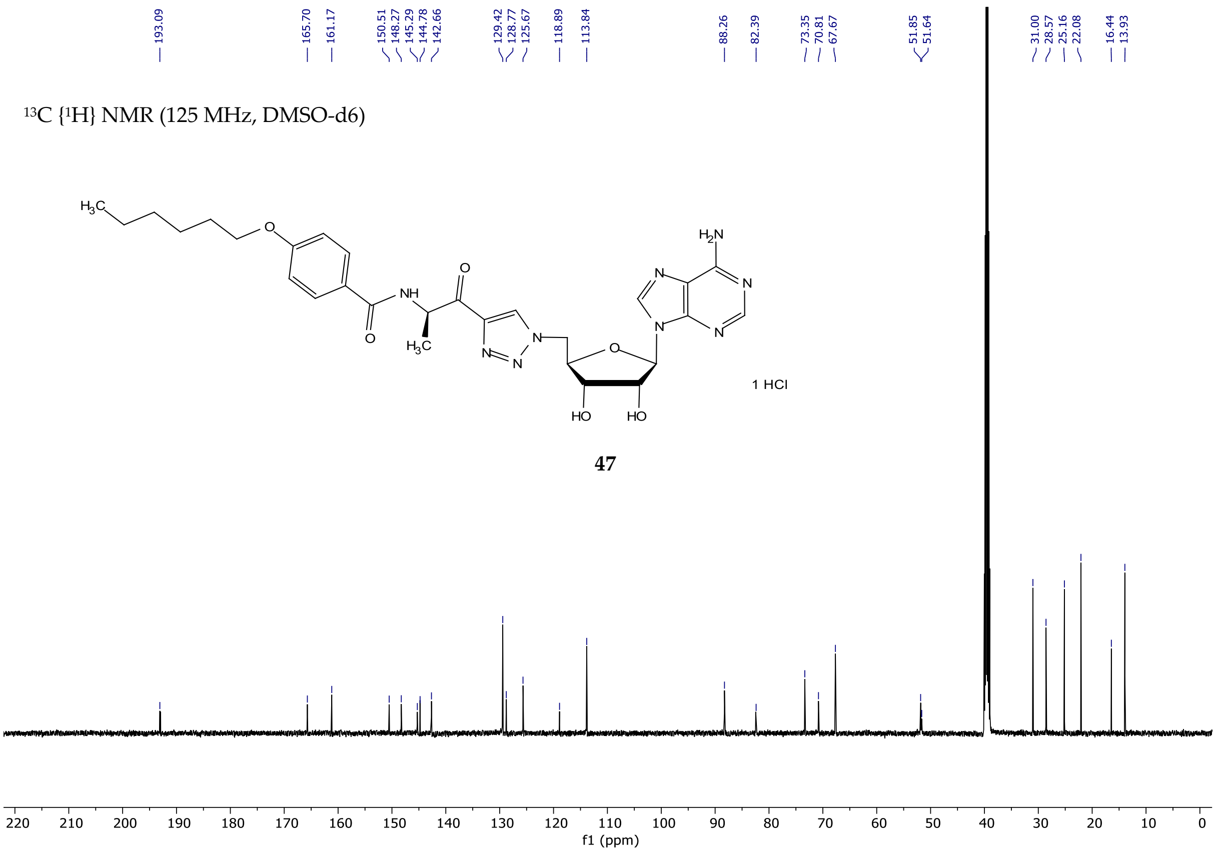
193.09 165.70 161.17 150.51 148.27 145.29 144.78 142.66 129.42 128.77 125.67 118.89 113.84 88.26 82.39 73.35 70.81 67.67 51.85 51.64 31.00 28.57 25.16 22.08 16.44 13.93
¹³C {¹H} NMR (125 MHz, DMSO-d6)
H3C
O
NH
O
H3C
O
N
N
N
O
HO
HO
H2N
N
N
N
N
1 HCl
47
220 210 200 190 180 170 160 150 140 130 120 110 100 90 80 70 60 50 40 30 20 10 0
f1 (ppm)

PDF Complete
Your complimentary use period has ended. Thank you for using PDF Complete.
Click Here to upgrade to Unlimited Pages and Expanded Features

rapport

## INFORMATION

| | | | |
|---|---|---|---|
| Sample Name: | DL536A | Acquired By: | System |
| Sample Type: | Unknown | Sample Set Name: | TR |
| Vial: | 32 | Acq. Method Set: | Pureté HPLC |
| Injection #: | 1 | Processing Method: | 2 |
| Injection Volume: | 10,00 ul | Channel Name: | 254,0nm@1 |
| Run Time: | 30,0 Minutes | Proc. Chnl. Descr.: | PDA 254,0 nm |
| Date Acquired: | 05/10/2022 13:59:19 CET | | |
| Date Processed: | 05/10/2022 14:55:09 CET | | |

Spectrum Index Plot

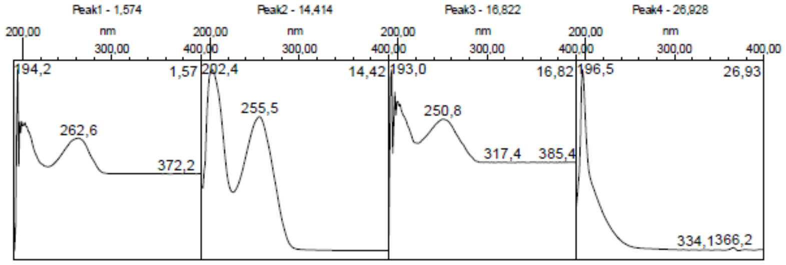


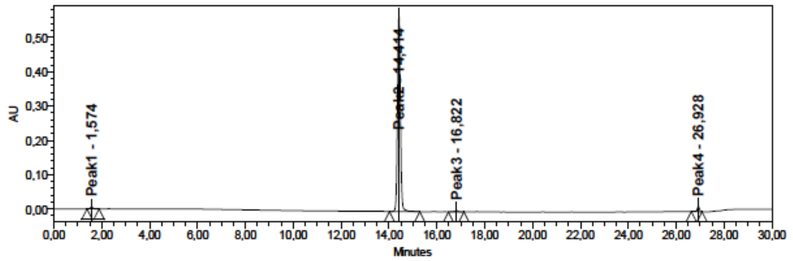


SampleName: DL536A; Vial: 32; Injection: 1; Date Acquired: 05/10/2022 13:59:19 CET

Peak Results

| | Name | RT | Area | % Area |
|---|---|---|---|---|
| 1 | Peak1 | 1,574 | 42952 | 0,85 |
| 2 | Peak2 | 14,414 | 4876539 | 96,51 |
| 3 | Peak3 | 16,822 | 38738 | 0,77 |
| 4 | Peak4 | 26,928 | 94586 | 1,87 |

Reported by User: System
Report Method: rapport
Report Method ID 5622
Page: 1 of 1

Project Name: IA-CELL1-AMY2
Date Printed:
05/10/2022
14:55:27 Europe/Paris

HPLC analysis for compound **47**

$^{1}H$ NMR (500 MHz, $CDCl_3$)

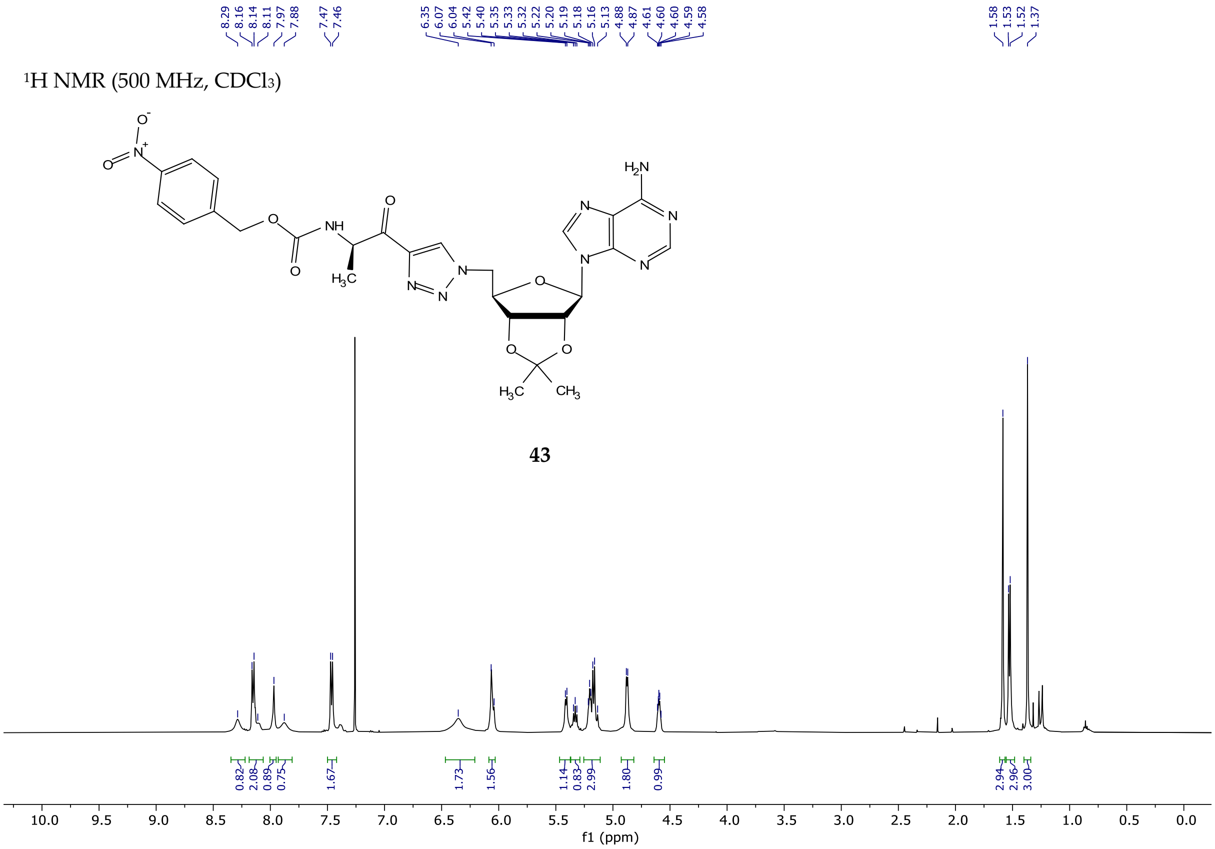


$^{13}C$ {$^{1}H$} NMR (125 MHz, $CDCl_3$)

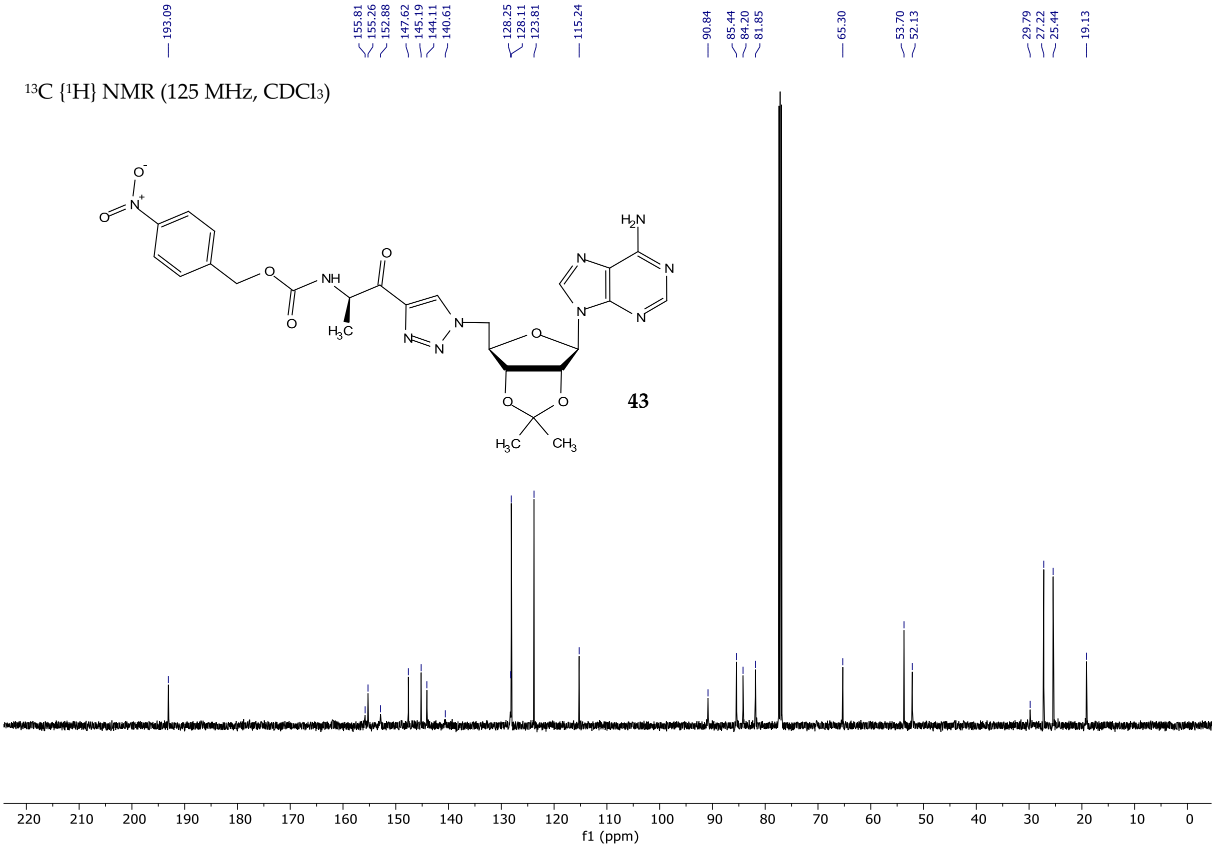

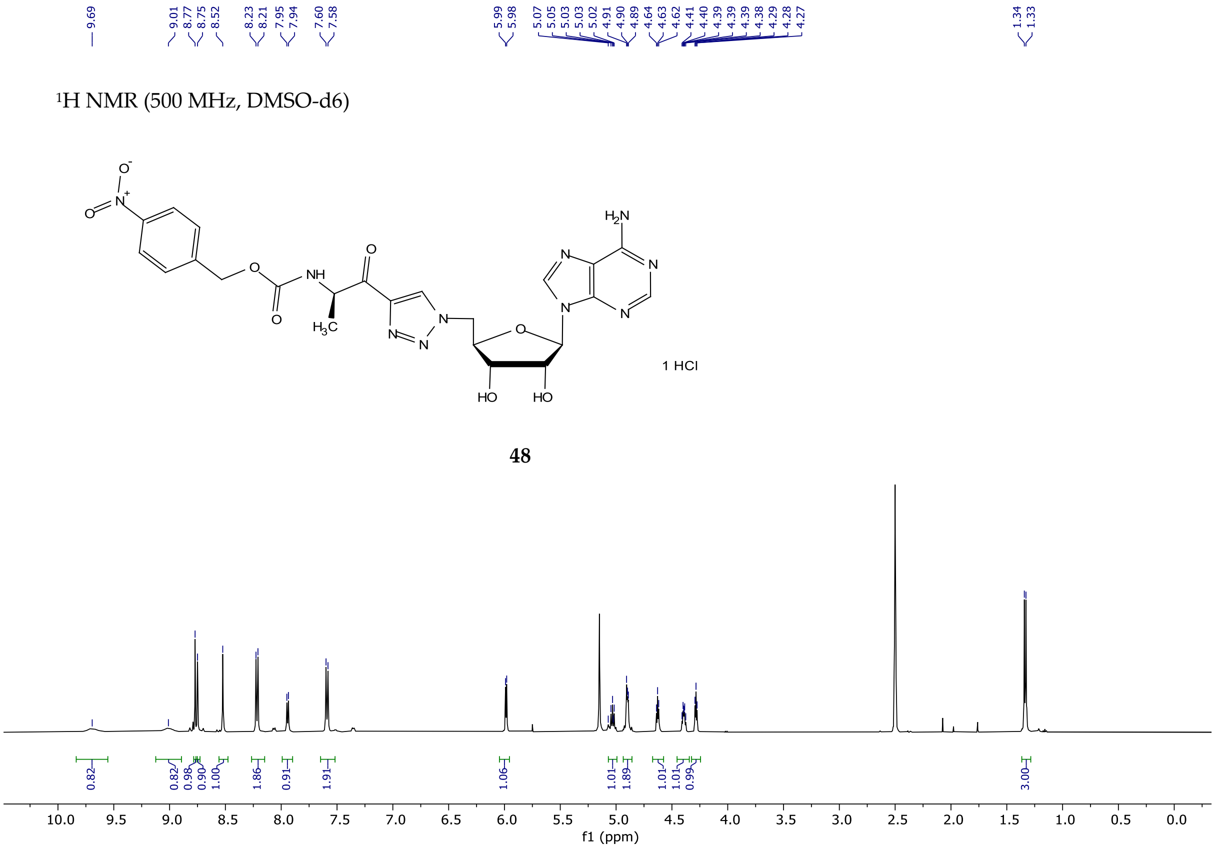

9.69
9.01
8.77
8.75
8.52
8.23
8.21
7.95
7.94
7.60
7.58
5.99
5.98
5.07
5.05
5.03
5.03
5.02
4.91
4.90
4.89
4.64
4.63
4.62
4.41
4.40
4.39
4.39
4.39
4.38
4.29
4.28
4.27
1.34
1.33
¹H NMR (500 MHz, DMSO-d6)
1 HCl
48
0.82
0.82
0.98
0.90
1.00
1.86
0.91
1.91
1.06
1.01
1.89
1.01
1.01
0.99
3.00
f1 (ppm)


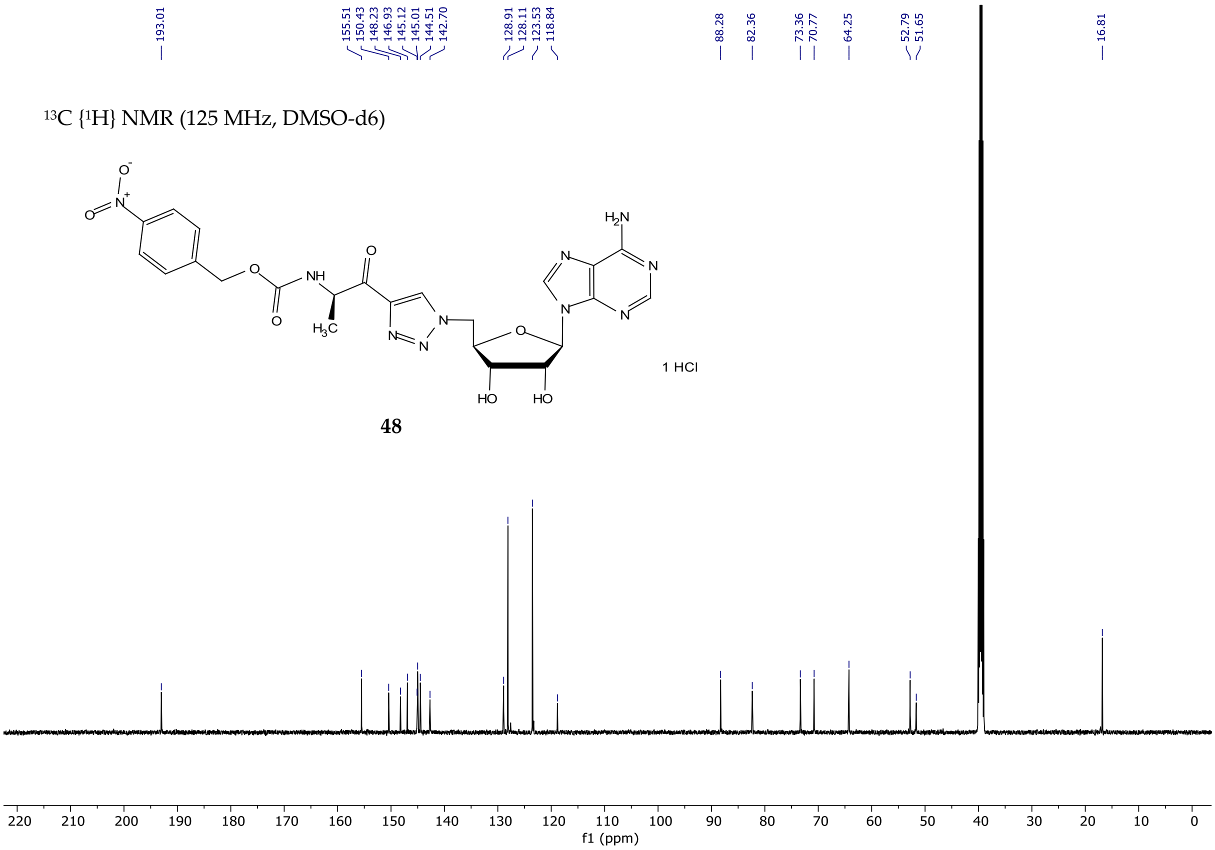

193.01
155.51
150.43
148.23
146.93
145.12
145.01
144.51
142.70
128.91
128.11
123.53
118.84
88.28
82.36
73.36
70.77
64.25
52.79
51.65
16.81
¹³C {¹H} NMR (125 MHz, DMSO-d6)
1 HCl
48
f1 (ppm)

PDF Complete
Your complimentary use period has ended. Thank you for using PDF Complete.
Click Here to upgrade to Unlimited Pages and Expanded Features

rapport

## INFORMATION

| | | | |
|---|---|---|---|
| Sample Name: | DL498A | Acquired By: | System |
| Sample Type: | Unknown | Sample Set Name: | F |
| Vial: | 5 | Acq. Method Set: | Pureté HPLC |
| Injection #: | 1 | Processing Method: | 2 |
| Injection Volume: | 10,00 ul | Channel Name: | 254,0nm |
| Run Time: | 30,0 Minutes | Proc. Chnl. Descr.: | PDA 254,0 nm |
| Date Acquired: | 28/07/2022 12:35:53 CET | | |
| Date Processed: | 29/07/2022 09:04:32 CET | | |

**Spectrum Index Plot**

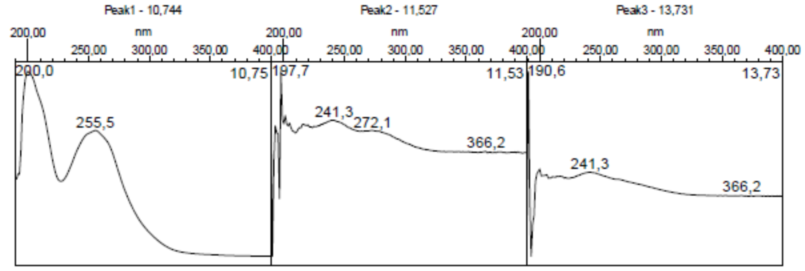


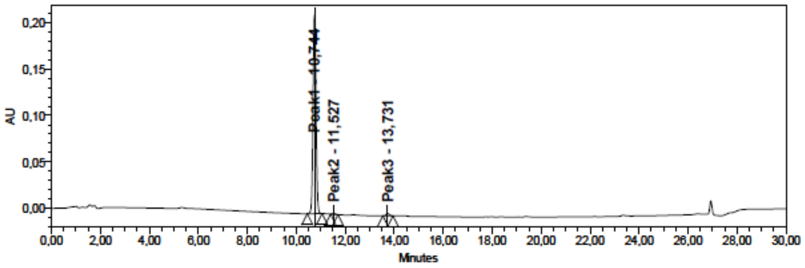


SampleName: DL498A; Vial: 5; Injection: 1; Date Acquired: 28/07/2022 12:35:53 CET

**Peak Results**

| | Name | RT | Area | % Area |
|---|---|---|---|---|
| 1 | Peak1 | 10,744 | 1788202 | 98,26 |
| 2 | Peak2 | 11,527 | 8847 | 0,49 |
| 3 | Peak3 | 13,731 | 22833 | 1,25 |

Reported by User: System
Report Method: rapport
Report Method ID 5622
Page: 1 of 1

Project Name: IA-CELL1-AMY2
Date Printed:
29/07/2022
09:06:51 Europe/Paris

HPLC analysis for compound **48**
